# Effects of Non-statin Lipid-Modifying Agents on Cardiovascular Morbidity and Mortality Among Statin-Treated Patients: A Systematic Review and Network Meta-Analysis

**DOI:** 10.3389/fphar.2019.00547

**Published:** 2019-05-22

**Authors:** Thanaputt Chaiyasothi, Surakit Nathisuwan, Piyameth Dilokthornsakul, Prin Vathesatogkit, Ammarin Thakkinstian, Christopher Reid, Wanwarang Wongcharoen, Nathorn Chaiyakunapruk

**Affiliations:** ^1^Department of Pharmacy, Faculty of Pharmacy, Mahidol University, Bangkok, Thailand; ^2^Department of Clinical Pharmacy, Faculty of Pharmacy, Srinakharinwirot University, Nakhon Nayok, Thailand; ^3^Department of Pharmacy Practice, Faculty of Pharmaceutical Sciences, Center of Pharmaceutical Outcomes Research, Naresuan University, Phitsanulok, Thailand; ^4^Department of Medicine, Faculty of Medicine, Ramathibodi Hospital, Mahidol University, Bangkok, Thailand; ^5^Section for Clinical Epidemiology and Biostatistics, Faculty of Medicine, Ramathibodi Hospital, Mahidol University, Bangkok, Thailand; ^6^School of Epidemiology and Preventive Medicine, Monash University, Melbourne, VIC, Australia; ^7^School of Public Health, Curtin University, Perth, WA, Australia; ^8^Division of Cardiology, Department of Internal Medicine, Faculty of Medicine, Chiang Mai University, Chiang Mai, Thailand; ^9^School of Pharmacy, Monash University Malaysia, Bandar Sunway, Selangor, Malaysia; ^10^School of Pharmacy, University of Wisconsin, Madison, WI, United States; ^11^Asian Centre for Evidence Synthesis in Population, Implementation and Clinical Outcomes, Health and Well-being Cluster, Global Asia in the 21st Century (GA21) Platform, Monash University Malaysia, Bandar Sunway, Malaysia

**Keywords:** non-statin lipid-modifying agent, statin-treated patient, cardiovascular morbidity, mortality, network meta-analysis

## Abstract

**Background:** Currently, there is a lack of information on the comparative efficacy and safety of non-statin lipid-lowering agents (NST) in cardiovascular (CV) disease risk reduction when added to background statin therapy (ST). This study determine the relative treatment effects of NST on fatal and non-fatal CV events among statin-treated patients.

**Methods:** A network meta-analysis based on a systematic review of randomized controlled trials (RCTs) comparing non-statin lipid-modifying agents among statin-treated patients was performed. PubMed, EMBASE, CENTRAL, and Clinicaltrial.gov were searched up to April 10, 2018. The primary outcomes were CV and all-cause mortalities. Secondary CV outcomes were coronary heart disease (CHD) death, non-fatal myocardial infarction (MI), any stroke, and coronary revascularization. Risks of discontinuations were secondary safety outcomes.

**Results:** Sixty-seven RCTs including 259,429 participants with eight interventions were analyzed. No intervention had significant effects on the primary outcomes (CV mortality and all-cause mortality). For secondary endpoints, proprotein convertase subtilisin/kexin type 9 inhibitor (PCSK) plus statin (PCSK/ST) significantly reduced the risk of non-fatal MI (RR 0.82, 95% CI 0.72–0.93, *p* = 0.003), stroke (RR 0.74, 95% CI 0.65–0.85, *p* < 0.001), coronary revascularization (RR 0.84, 95% CI 0.75–0.94, *p* = 0.003) compared to ST. Combinations of ST and all NST except PCSK and ezetimibe showed higher rate of discontinuation due to adverse events compared to ST.

**Conclusions:** None of NST significantly reduced CV or all-cause death when added to ST. PCSKs and to a lesser extent, ezetimibe may help reduce cardiovascular events with acceptable tolerability profile among broad range of patients.

## Introduction

Statins or 3-hydroxy-3-methyl-glutaryl-coenzyme A (HMG-CoA) reductase inhibitors are the cornerstone of atherosclerotic cardiovascular disease (ASCVD) risk reduction therapy for both primary and secondary preventions (Baigent et al., 2005; Taylor et al., 2013; Fulcher et al., 2015). Nevertheless, a significant number of patients do not achieve optimal lipid level or still experience cardiovascular (CV) events despite receiving statin therapy (Fruchart et al., 2008). The concept of adding non-statin lipid-lowering agents (NST) on top of statins has therefore been implemented to achieve the lipid goal with the hope that it may reduce hard clinical outcomes. Despite their lipid modifying effects, when tested in large-scale clinical trials, these agents did not uniformly lead to a reduction in CV events when added to statin therapy. Some agents were shown to have neutral effects (Barter et al., 2007a; Ginsberg et al., 2010; Kromhout et al., 2010; Boden et al., 2011; Schwartz et al., 2012; Landray et al., 2014) while some agents were shown to reduce some forms of cardiovascular outcomes (Yokoyama et al., 2007; Cannon et al., 2015a; Robinson et al., 2015; Sabatine et al., 2015). Up to now, most CV outcome studies involving a combination of lipid-modifying therapies were a comparison of a non-statin lipid-modifying agent plus statin therapy vs. statin monotherapy. There remains insufficient data regarding the comparative efficacy and safety of various non-statin agents among statin-treated patients. As a result, current practice guidelines are making recommendation based on an inferential interpretation without data from direct comparison (Catapano et al., 2016; Lloyd-Jones et al., 2017). Since most trials evaluating NST used statin as a comparator, indirect comparisons across trials based on a common comparator is therefore possible through a network meta-analysis (Mills et al., 2012; Cipriani et al., 2013). Therefore, we conducted a systematic review and a network meta-analysis to evaluate the relative treatment effects and safety of NST on cardiovascular morbidity and mortality among statin users.

## Methods

### Study Design

This study was performed in accordance to the Preferred Reporting Items for Systematic Reviews and Meta-Analyses (PRISMA) extension statement for network meta-analysis (Hutton et al., 2015). The study protocol was registered in PROSPERO with the number of registration of CRD42016052839. Additionally, this study protocol was approved by the Institutional Review Board of Mahidol University (COE.No. MU-DT/PY-IRB 2017/PY055).

#### Data Sources and Search Strategy

The following databases were used to search for original research articles from inception to April 2018: PubMed, Embase, Cochrane Central Register of Control Trials (CENTRAL), and ClinicalTrials.gov. Combinations of terms of medical subject headings (MeSH) and keywords were used in the search strategy. The MeSH and keywords contain Ezetimibe, “Omega-3 fatty acid,” Fibrate, Niacin, “Bile acid sequestrant,” “Proprotein convertase subtilisin/kexin,” “Cholesteryl ester transfer protein,” Lomitapide, Mipomersen, Phytosterol, Non-statin, statin, name of statin (atorvastatin, simvastatin, pravastatin, fluvastatin, rosuvastatin, pitavastatin, lovastatin), cardiovascular, death, mortality, “myocardial infarction” stroke, and synonymous words. References of papers derived for full text review were screened to identify potential studies not indexed in the above databases. No language restriction was applied (Appendix 1).

#### Study Selection

We included only randomized controlled trials (RCTs) if they met the inclusion criteria including (1) studied in adults (age ≥18 years), (2) comparing NSTs among statin-treated patients, where statin was used either as monotherapy or as a part of combination therapy, (3) reported any outcome of interest including CV mortality, all-cause mortality, individual (not composite) events of coronary heart disease (CHD) mortality or non-fatal myocardial infarction (MI), any stroke, or coronary revascularization (4) with the entire follow-up duration of ≥24 weeks.

#### Data Extraction and Quality Assessment

Two reviewers (TC and PD) independently screened the titles and abstracts of retrieved citations to identify potentially relevant studies. Relevant data were abstracted using a standardized extraction form including study characteristics, patient characteristics, interventions, outcomes, and other relevant findings. The Revised Cochrane Risk of Bias Tool for randomized trials (RoB 2.0) was used to assess risk of bias among the included studies (Higgins et al., 2016). The quality assessment was undertaken by two reviewers (T.C. and P.D.) independently. Disagreements were resolved by consensus, or with consultation of a third party.

#### Interventions

NSTs were bile acid sequestrants (BAS), cholesteryl ester transfer protein inhibitors (CETP), ezetimibe (EZT), fibrates (FBT), microsomal transfer protein inhibitors (MTP), niacin (NIA), omega-3 fatty acids (OMG3), proprotein convertase subtilisin/kexin-9 inhibitors (PCSK), or miscellaneous agents following 2017 ACC-AHA (Lloyd-Jones et al., 2017) and 2016 ESC guidelines of dyslipidemias (Catapano et al., 2016). Combinations of NST were also evaluated. ST was used as the reference for network meta-analysis.

#### Outcomes of Interest

The primary outcomes were cardiovascular death and all-cause mortality. Secondary cardiovascular endpoints were (1) CHD mortality, (2) non-fatal MI, (3) any stroke, and (4) coronary revascularization. Although composite CV outcome is the common endpoint in CV outcome trials (e.g., any major vascular event (MVE) or any major adverse cardiovascular event (MACE), we did not consider a composite CV outcome because of non-mutually exclusive patients with events and varied definitions of MVE or MACE across studies. For secondary safety endpoints, risks of all-cause discontinuation (acceptability) and discontinuation due to adverse events (tolerability) were also investigated.

#### Quality of Evidence

Evaluation of evidence quality from both direct and network meta-analysis was performed using GRADEpro® GDT software online version (http://www.guidelinedevelopment.org/ [access April 2018]). There were 4 levels of quality of evidence including, very low, low, moderate, and high (Balshem et al., 2011; Puhan et al., 2014). Grading of evidence for each outcome was performed based on 5 domains including risk of bias, inconsistency, indirectness, imprecision, and publication bias. Two independent reviewers (T.C and P.D) assessed the quality of evidence. When discrepancy cannot be resolved by discussion, the third reviewer was consulted to make a final decision.

#### Data Synthesis and Statistical Analysis

The relative treatment effects of all outcomes of interest among treatment interventions were estimated using the risk ratio (RR). A direct meta-analysis was applied for pooling RRs across studies using a random-effects model (Dersimonian and Laird, 1986). Cochran Q test and the I-squared statistics were deployed to assess heterogeneity (Higgins et al., 2003). Heterogeneity was present if the Cochrane Q test was significant (*P* < 0.10) or *I*^2^ ≥ 50%.

A network meta-analysis with consistency model was constructed to compare all interventions using ST as the common comparator. This approach assumes “consistency” of treatment effects across all included trials—that is, the direct and indirect estimates are consistent (Lu and Ades, 2004; Caldwell et al., 2005). Global inconsistency test by fitting design-by-treatment in the inconsistency model was used for examining the assumption of inconsistency in the entire network (Dias et al., 2010). Additionally, transitivity was explored by assessing the distribution of clinical and methodological variables that might affect the outcome of interests. These data also were available across treatment comparisons (Cipriani et al., 2013). The rankograms, surface under the cumulative ranking (SUCRA) curves (Salanti et al., 2011), and mean ranks were calculated to rank all interventions in the network meta-analysis model. Comparison-adjusted funnel plot was finally used to evaluate publication bias (Chaimani et al., 2013).

Pre-specified subgroup analyses were performed by several clinical factors including indication of treatment (primary, secondary, or mixed indication), intensity of statin therapy (low/moderate, moderate, or moderate/ high) based on the ACC/AHA 2013 definition (Stone et al., 2014), requirement of statin prior to starting NST (optimal LDL-C level/maximally tolerated dose vs. no optimal LDL-C target/maximally tolerated dose), level of cardiovascular risk (non-high CV risk vs. high CV risk) adapted from the ESC 2016 definition (Catapano et al., 2016), age (<65 vs. ≥65 years), percentage of familial hypercholesterolemia (FH) (≥80 vs. <80%) and baseline lipid level (LDL-C, non-HDL-C, HDL, and TG). Additionally, we conducted a sensitivity analysis by excluding the following conditions of studies; studies with high risk of bias, non-adjudicated CV events, follow-up duration <1 year, and small sample size study (<25 percentile) (Dechartres et al., 2014). All analyses were performed in STATA® version 14.2 (StataCorp, College Station, Texas, USA). A *p*-value < 0.05 was considered statistically significant.

## Results

### Study Selection

A total of 20,508 potential studies were identified by searching strategies (eTable 1.1), 68 studies including 259,537 adults were eligible for the qualitative review. However, only 67 studies with 259,429 participants were included for network meta-analysis except for one study reported composite CV outcome but not for individual CV events. The searching results and the PRISMA flowchart were shown in eFigure 1.1.

### Study Characteristics

Six different classes of NST including CETP, EZT, FBT, NIA, OMG3, and PCSK were used among 67 included studies. Among these trials, there were 8 interventions including ST, CETP/ST, EZT/ST, FBT/ST, NIA/ST, OMG3/ST, PCSK/ST, and NIA + EZT/ST. Most studies (65 studies) were with 2-arm comparison while the two trials (Bays et al., 2015; Farnier et al., 2016) were with multiple comparisons. ST was mostly used as the comparator (64 in 67) while NST plus ST was used as the comparator in 3 trials (Guyton et al., 2008; Taylor et al., 2009; Cannon et al., 2015b). For trial design, the majority (74%) were double-blind RCT. Studied population in these trials were mostly high risk patients under the age of 65 who were receiving moderate to high intensity statin with mean age ranged from 45.9 to 84.1 years. It is important to note that 40% of the trial used moderate intensity of statin while another 40% used moderate to high intensity of statin. Proportion of male patients ranged from 31.5 to 93.7%. Most trials were secondary prevention or mixed prevention trials with small contribution (9%) of primary prevention trials. Two thirds of the trials were with a follow-up period of ≥1 year with a range of 6–72 (0.5–6 years) months of treatment duration. Summary of all comparisons are shown in Appendix 2 while key characteristics of these trials are shown in Table 1. Additional details of included studies such as number of patients, type of study population and interventions were provided in Appendix 3.

**Table 1:** Characteristics of the 68 included studies.

**Study group/first author**	**Published** **year**	**Treatment**	**Study** **size (N)**	**Male** **(%)**	**Age** **(year)**	**Target population**	Intensity of statin^*^	**Follow-up** **duration (month)**
IMPROVE-IT (Cannon et al., 2015a)	2015	EZT/ST vs. ST	18,144	75.7	≥50	ACS	Moderate	72
ENHANCE (Kastelein et al., 2008)	2008	EZT/ST vs. ST	720	51.4	30–75	FH	High	24
ARBIRTER2 (Taylor et al., 2004)	2004	NIA/ST vs. ST	167	91.0	≥30	CHD	Moderate	12
AIM-HIGH (Boden et al., 2011)	2011	NIA/ST vs. ST	3,414	85.2	≥45	ASCVD	Moderate/high	36
HPS2-THRIVE (Landray et al., 2014)	2014	NIA/ST vs. ST	25,673	82.7	50–80	ASCVD	Moderate	47
ACCORD (Ginsberg et al., 2010)	2010	FBT/ST vs. ST	5,518	69.3	40–79	DM	Moderate	56
JELIS (Yokoyama et al., 2007)	2007	OMG3/ST vs. ST	18,645	31.5	≥40	HC	Low	55
ILLUSTRATE (Nissen et al., 2007)	2007	CETP/ST vs. ST	1,188	70.5	18–75	Coronary stenosis by angiography	Moderate	24
ILLUMINATE (Barter P. J. et al., 2007)	2007	CETP/ST vs. ST	15,067	77.8	45–75	ASCVD or DM	Unclassified	18
dal-OUTCOME (Schwartz et al., 2012)	2012	CETP/ST vs. ST	15,871	80.5	≥45	Recent ACS	Unclassified	31
ODYSSEY LONG TERM (Robinson et al., 2015)	2015	PCSK/ST vs. ST	2,341	61.8	≥18	HeFH or CHD or high risk CHD	Moderate/high	20
OSLER (Sabatine et al., 2015)	2015	PCSK/ST vs. ST	4,465	50.8	≥18	Hyperlipidemia	Moderate/high	11
ARBIRTER6 (Taylor et al., 2009)	2009	NIA/ST vs. EZT/ST	363	80.2	≥30	CHD or CHD risk equivalents	Moderate/high	14
(Guyton et al., 2008)	2008	NIA + EST/ST vs. EZT/ST	1,220	50.1	18–79	IIa or IIb hyperlipidemia	Moderate	6
ELIMIT (Brunner et al., 2013)	2013	NIA + EZT/ST vs. ST	95	93.7	Not specified	PAD	Moderate	24
SEACOAST I (Ballantyne et al., 2008a)	2008	NIA/ST vs. ST	314	50.6	≥21	Mixed hyperlipidemia	Moderate	6
(Wang et al., 2016)	2016	EZT/ST vs. ST	106	72.5	any	CHD	Moderate	12
PRECISE-IVUS (Tsujita et al., 2015)	2015	EZT/ST vs. ST	246	78.0	30–85	ACS or SA	Low/moderate	10
(Masuda et al., 2015)	2015	EZT/ST vs. ST	51	87.5	20–80	SAP with PCI	Moderate	6
(Luo et al., 2014)	2014	EZT/ST vs. ST	84	52.3	Not specified	HC	Moderate	12
OMEGA (Rauch et al., 2010)	2010	OMG3/ST vs. ST	3,851	74.4	≥18	Acute MI	Unclassified	12
ODYSSEY OPTIONS II (Farnier et al., 2016)	2016	PCSK/ST vs. EZT/ST vs. ST	305	61.3	≥ 18	HC with high or very high CV risk	Moderate/high	6
ODYSSEY COMBO II (Cannon et al., 2015b)	2015	PCSK/ST vs. EZT/ST	720	73.6	≥18	CHD or CHD risk equivalents	Moderate/high	12
DESCARTES (Blom et al., 2014)	2014	PCSK/ST vs. ST	901	47.7	18–75	HC	Moderate/high	12
(West et al., 2011)	2011	EZT/ST vs. ST	44	62.5	30–85	PAD	Moderate	24
(Arimura et al., 2012)	2012	EZT/ST vs. ST	44	70.5	Not specified	SA with stent	Moderate	8
RADIANCE-2 (Bots et al., 2007)	2007	CETP/ST vs. ST	752	64.0	18–70	Mixed dyslipidemia	Moderate	20
REALIZE (Kastelein et al., 2015a)	2015	CETP/ST vs. ST	306	54.0	18–80	HeFH	Moderate/high	12
DEFINE (Cannon et al., 2010)	2010	CETP/ST vs. ST	1,623	77.4	18–80	CHD or CHD risk equivalents	Unclassified	19
FIRST (Davidson et al., 2014)	2014	FBT/ST vs. ST	682	68.0	≥45	Dyslipidemia CHD or CHD risk equivalents	Moderate/high	26
RADIANCE-1 (Kastelein et al., 2007)	2007	CETP/ST vs. ST	904	49.4	18–70	HeFH	High	24
(Derosa et al., 2004)	2004	FBT/ST vs. ST	48	50.1	18–80	DM with CHD	Moderate	12
(Durrington et al., 2001)	2001	OMG3/ST vs. ST	59	72.9	≤75	CHD with high TG	Moderate	6
ODYSSEY FH I& II (Kastelein et al., 2015b)	2015	PCSK/ST vs. ST	735	55.1	≥18	HeFH	Moderate/high	20
ODYSSEY COMBO I (Kereiakes et al., 2015)	2015	PCSK/ST vs. ST	316	65.8	≥18	CHD or CHD risk equivalent	Moderate/high	12
(Nishio et al., 2014)	2014	OMG3/ST vs. ST	31	86.7	≥18	PCI with SA/ACS	Low/moderate	9
ODYSSEY JAPAN (Teramoto et al., 2016)	2016	PCSK/ST vs. ST	216	60.6	≥20	HeFH/HC with CHD/CHD risk equivalents	Unclassified	12
(Stein et al., 2010)	2010	CETP/ST vs. ST	135	78.5	18–75	CHD or CHD risk equivalents	Moderate/high	12
ODYSSEY OPTIONS I (Bays et al., 2015)	2015	PCSK/ST vs. EZT/ST vs. ST	355	65.1	≥18	HC with high or very high CV risk	Moderate/high	8
dal-PLAUQE (Fayad et al., 2011)	2011	CETP/ST vs. ST	130	81.5	18–75	CHD or CHD risk equivalents	Unclassified	24
dal-VESSEL (Luscher et al., 2012)	2012	CETP/ST vs. ST	476	90.5	18–75	CHD or CHD risk equivalents	Unclassified	9
SEACOAST II (Ballantyne et al., 2008b)	2008	NIA/ST vs. ST	343	54.5	≥21	Dyslipidemia (non-HDL-C)	Moderate	6
GLAGOV (Nicholls et al., 2016)	2016	PCSK/ST vs. ST	970	72.2	≥18	Coronary stenosis by angiography with CVD risk	Moderate/high	20
UK-HARP-II (Landray et al., 2006)	2006	EZT/ST vs. ST	203	69.5	≥18	CKD	Moderate	6
(Shaw et al., 2009)	2009	EZT/ST vs. ST	68	84.5	Not specified	Cardiac transplant treated with cyclosporine	Low/moderate	6
(Kouvelos et al., 2013)	2013	EZT/ST vs. ST	262	89.7	Not specified	Patient with vascular surgery	Moderate	12
ODYSSEY HIGH FH (Ginsberg et al., 2016)	2016	PCSK/ST vs. ST	107	53.3	≥18	HeFH	Moderate/high	20
(Ballantyne et al., 2017a)	2017	CETP/ST vs. ST	459	67.7	18–80	Hypercholesterolemia	Moderate/high	6
ALPHA OMEGA (Kromhout et al., 2010)	2010	OMG3/ST vs. ST	4,837	78.2	60–80	History of MI	Unclassified	41
FOURIER (Sabatine et al., 2017)	2017	PCSK/ST vs. ST	27,564	75.4	40–85	ASCVD	Moderate/high	26
SPIRE-1 and−2 (Ridker et al., 2017a)	2017	PCSK/ST vs. ST	27,438	70.4	≥18	ASCVD or high CV risk	Moderate/high	7 and 12
(Ridker et al., 2017b)	2017	PCSK/ST vs. ST	4,449	58.3	≥18	Hyperlipidemia	Moderate/high	Up to 12
(Luo et al., 2016)	2016	EZT/ST vs. ST	148	56.8	Not specified	CHD	Moderate	12
(Liu et al., 2017)	2017	EZT/ST vs. ST	230	51.7	80–90	ACS	Moderate	12
(Nosaka et al., 2017)	2017	OMG3/ST vs. ST	241	76.0	Not specified	ACS with PCI	Moderate	12
(Lincoff et al., 2017)	2017	CETP/ST vs. ST	12,092	77.0	≥18	ASCVD	Moderate/high	28
(Bowman et al., 2017)	2017	CETP/ST vs. ST	30,449	83.9	≥50	ASCVD	Moderate/high	49.2
(Hagiwara et al., 2017)	2017	EZT/ST vs. ST	1,734	75.5	≥20	ACS	Moderate	46.3
(Hibi et al., 2018)	2018	EZT/ST vs. ST	128	80.0	Not specified	ACS	Moderate	10
(Miyoshi et al., 2018)	2018	OMG3/ST vs. ST	198	55.0	>20	Hypercholesterolemia	Moderate	12
(Watanabe et al., 2017)	2017	OMG3/ST vs. ST	241	82.0	≥20	CAD (SA or ACS) with PCI and hypercholesterolemia	Moderate	8
(Koh et al., 2018)	2018	PCSK/ST vs. ST	199	82.5	≥18	High CV risk	Moderate/high	6
(Leiter et al., 2017)	2017	PCSK/ST vs. ST	517	55.1	≥18	T1DM or T2DM (treated with insulin) with ASCVD and/or CV risk factor(s)	Moderate/high	6
(Teramoto et al., 2017)	2017	CETP/ST vs. ST	307	67.8	18–80	Dyslipidemia	Unclassified	6
(Ballantyne et al., 2017b)	2017	CETP/ST vs. ST	583	72.7	18–80	Dyslipidemia	Unclassified	6
(Ray et al., 2018)	2018	PCSK/ST vs. ST	413	52.3	≥18	T2DM and mixed dyslipidemia	Moderate/high	6
(Schwartz et al., 2018)	2018	PCSK/ST vs. ST	18,924	74.8	≥40	ACS	Moderate/high	34
(Sang et al., 2009)^¶^	2009	NIA/ST vs. ST	108	61.1	Not specified	CAD	Moderate	12

* *Adapted from 2013 ACC/AHA guideline (Stone et al., 2014), High intensity: atorvastatin (≥40 mg), rosuvastatin (≥20 mg), simvastatin (≥80 mg); Moderate intensity: atorvastatin (10–20 mg), rosuvastatin (5–10 mg), simvastatin (20–40 mg), pravastatin (40–80 mg), lovastatin (≥40 mg), fluvastatin (80 mg), pitavastatin (2–4 mg); Low intensity: atorvastatin (<10 mg), rosuvastatin (<5 mg), simvastatin (<20 mg), pravastatin (<40 mg), lovastatin (<40 mg), fluvastatin (<80 mg) pitavastatin (<2 mg)*.

¶ *The study was not included in the network meta-analysis*.

*CETP/ST, cholesteryl ester transfer protein inhibitor + statin; EZT/ST, ezetimibe + statin; FBT/ST, fibrate + statin; NIA/ST, niacin + statin; OMG_3_/ST, omega-3 fatty acids + statin; PCSK/ST, proprotein convertase subtilisin/kexin type 9 inhibitor + statin; NIA+EZT/ST, niacin + ezetimibe + statin; ST, statin monotherapy; ACS, Acute coronary syndrome; ASCVD, Atherosclerotic cardiovascular disease; CV, cardiovascular; CHD, Coronary heart disease; DM, diabetes mellitus; FH, Familial hypercholesterolemia; HeFH, Heterozygous familial hypercholesterolemia; HC, Hypercholesterolemia; MI, myocardial infarction; PCI, percutaneous coronary intervention; PAD, Peripheral artery disease; SA, stable angina; SAP, stable angina pectoris*.

### Risk of Bias

Based on the Revised Cochrane Risk of Bias Tool for randomized trials (RoB 2.0) (Higgins et al., 2016), 31, 40, and 29% of studies were considered as at low risk, some concerns, and high risk of bias, respectively (Appendix 4, eFigure 4.1). Among five domains evaluated, inadequate description of allocation concealment and blinding process along with missing outcome data were the three most common reasons for potential bias. For trials with high risk of bias (20 trials with 10,812 patients which represented about 4% of total population), the majority were relatively small trials with <1,000 patients in each trial. Additional details for the assessment of risk of bias were provided in Appendix 4, eTable 4.1.

### Effects of Non-statin Therapy on Primary and Secondary Outcomes

Pair-wise meta-analyses were performed for eight outcomes (see Appendix 5), all pooling were with low heterogeneity except six pair-wise comparisons (1 for coronary revascularization, 3 for any discontinuation and 2 for discontinuation from adverse events) in which the *I*^2^ ranged from 61.9 to 84%. We explored but could not identify the source of heterogeneity.

**Figure 1:**
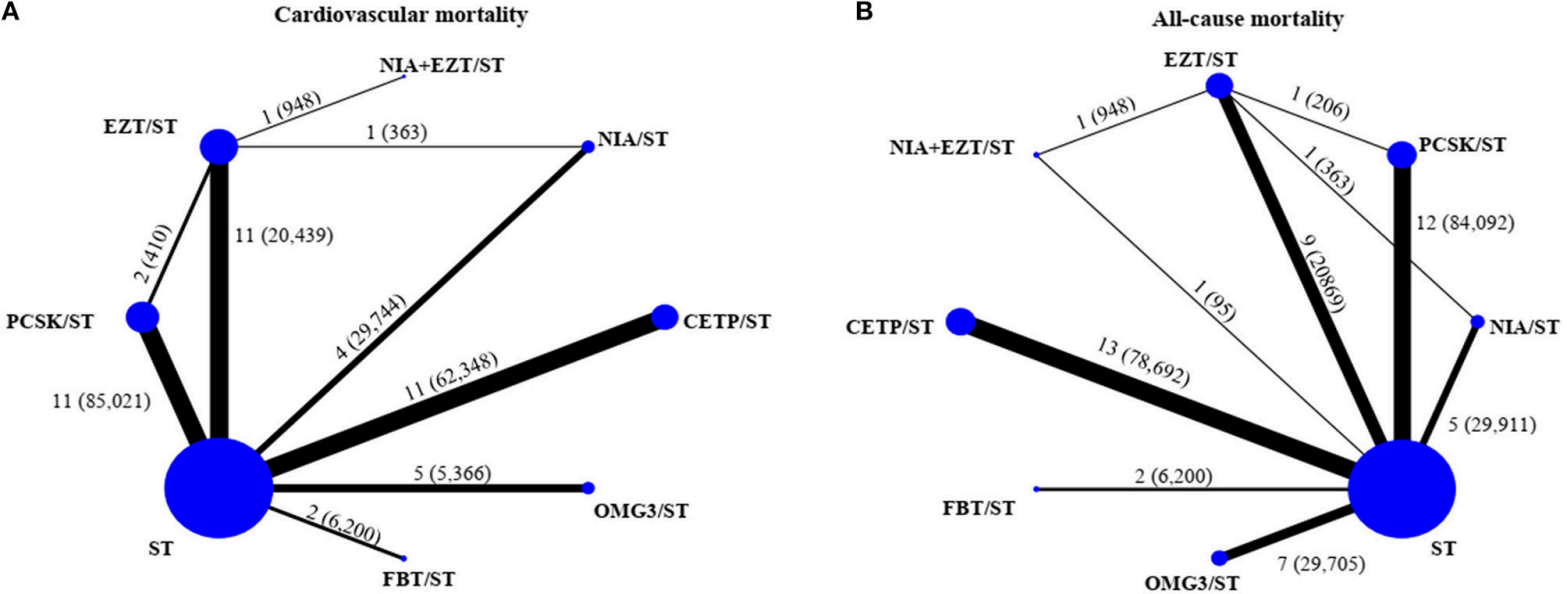
Network of eligible comparisons for primary outcomes [**(A)** cardiovascular mortality and **(B)** all-cause mortality]. The size of the node corresponds to the number of individual studies that studied the interventions. The directly compared interventions are linked with a line, the thickness of which corresponds to the number of studies that assess respective comparison. CETP/ST, cholesteryl ester transfer protein inhibitor + statin; EZT/ST, ezetimibe + statin; FBT/ST, fibrate + statin; NIA/ST, niacin + statin; OMG3/ST, omega-3 fatty acids + statin; PCSK/ST, proprotein convertase subtilisin/kexin type 9 inhibitor + statin; NIA+EZT/ST, niacin + ezetimibe + statin; ST, statin monotherapy.

Network of eligible comparisons for primary and secondary outcomes were provided in Figure 1 and Appendix 6. Global inconsistency test was performed and found no evidence of inconsistency of treatment effects for the outcomes (Appendix 7). In addition, transitivity was explored by comparing distributions of age, duration of treatments, intensity of statin, and indication of treatment. These indicated no evidence of intransitivity, see Appendix 8. Comparisons among all treatment interventions for the outcomes were demonstrated in Appendix 9. SUCRAs are provided in Appendix 10. The lists of included studies for the network meta-analysis of primary and secondary outcomes were presented in Appendix 11.

### Primary Outcomes

**Figure 2:**
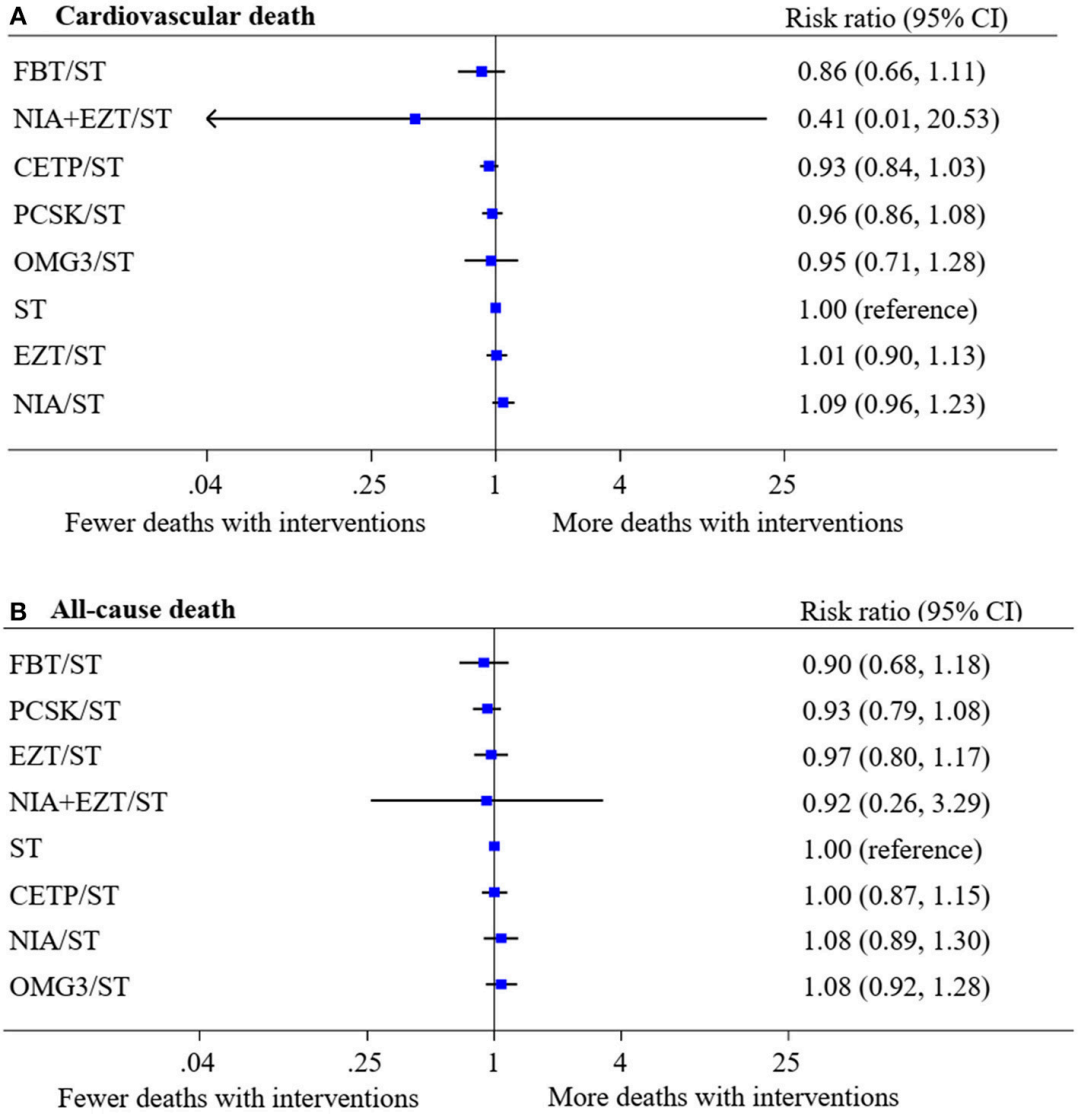
Network meta-analysis of treatment interventions compared with statin monotherapy for primary outcomes. Summary estimate represents risk ratio of **(A)** cardiovascular death and **(B)** all-cause death. Interventions were ranked by Surface under the cumulative ranking (SUCRA) values. CI, confidence interval; CETP/ST, cholesteryl ester transfer protein inhibitor + statin; EZT/ST, ezetimibe + statin; FBT/ST, fibrate + statin; NIA/ST, niacin + statin; OMG3/ST, omega-3 fatty acids + statin; PCSK/ST, proprotein convertase subtilisin/kexin type 9 inhibitor + statin; NIA+EZT/ST, niacin + ezetimibe + statin; ST, statin monotherapy.

A total of 44 studies (210,179 participants, 5,052 cases with events) and 50 studies (249,196 participants, 11,112 cases with events) were analyzed for the risk of CV death and all-cause death, respectively. Networks of eight treatment interventions for CV and all-cause mortality were mapped as shown in Figure 1. Overall, there were no statistically significant differences in both primary outcomes among various NST compared to ST (Figure 2). Additionally, no significant difference on estimated effects was seen among non-statin therapies for both primary outcomes (eTables 9.1, 9.2). Results of SUCRA rank on both outcomes were shown in eTables 10.1, 10.2.

### Secondary Outcomes

Treatment interventions were mapped for CHD mortality, non-fatal MI, stroke, and coronary revascularization using data from 43, 37, 41, and 36 studies, respectively (see eFigures 6.1–6.4).

**Figure 3:**
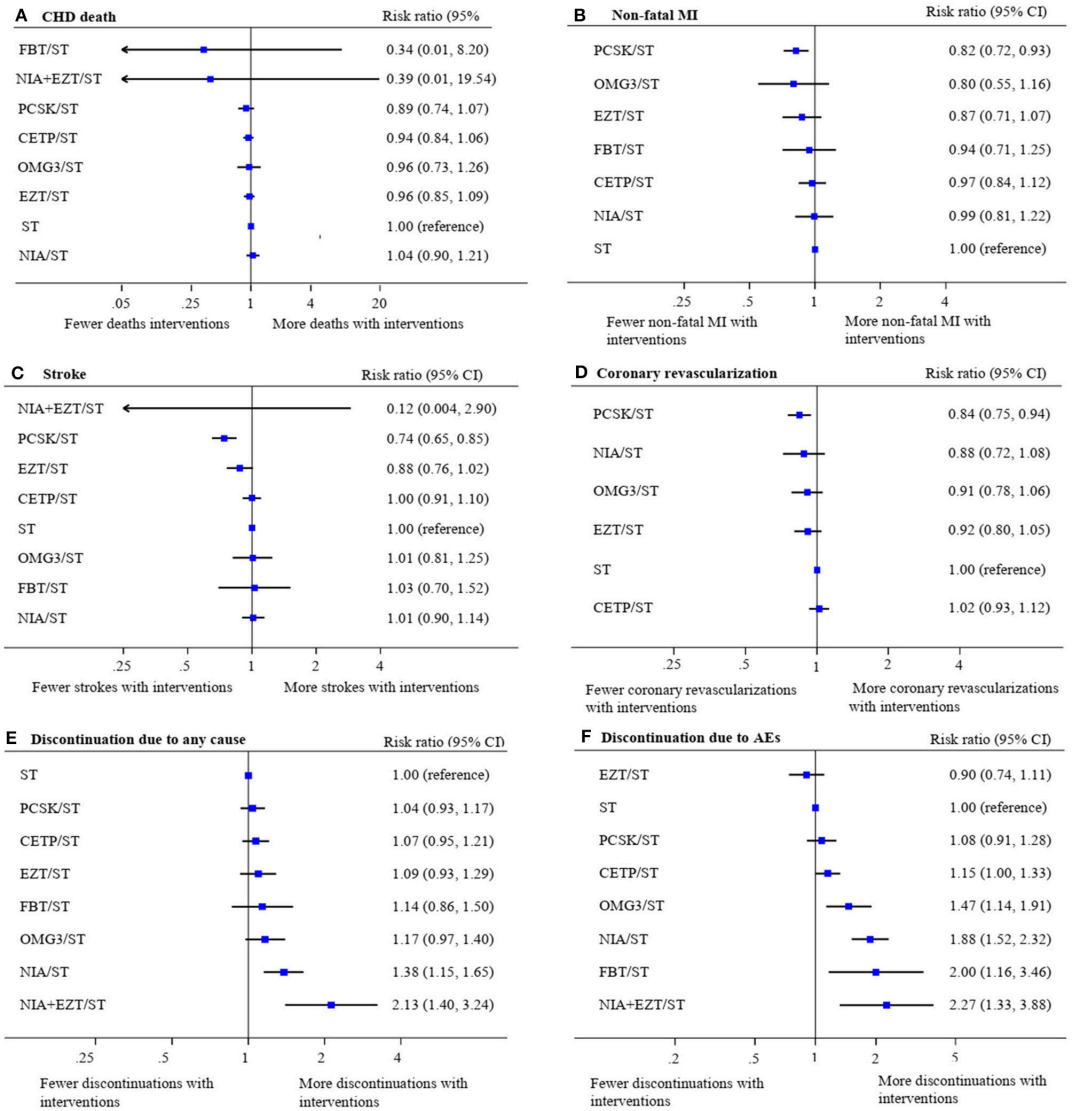
Network meta-analysis of treatment interventions compared with statin monotherapy for secondary cardiovascular endpoints and discontinuations. Summary estimate represents risk ratio of **(A)** coronary heart disease death, **(B)** non-fatal myocardial infarction, **(C)** stroke, **(D)** coronary revascularization, **(E)** discontinuation due to any cause, and **(F)** discontinuation due to adverse events. Interventions were ranked by SUCRA values. AE, adverse event; CHD, coronary heart disease; CI, confidence interval; CETP/ST, cholesteryl ester transfer protein inhibitor + statin; EZT/ST, ezetimibe + statin; FBT/ST, fibrate + statin; MI, myocardial infarction; NIA/ST, niacin + statin; OMG3/ST, omega-3 fatty acids + statin; PCSK/ST, proprotein convertase subtilisin/kexin type 9 inhibitor + statin; NIA+EZT/ST, niacin + ezetimibe + statin; ST, statin monotherapy.

The treatment effects for these outcomes compared with ST were estimated (Figure 3). Overall, there were no differences in the risk of CHD mortality among all treatment comparisons. However, PCSK/ST was significantly reduced the risks of non-fatal MI (RR 0.82, 95% CI 0.72–0.93, *p* = 0.003), stroke (RR 0.74, 95% CI 0.65–0.85, *p* < 0.001) and coronary revascularization (RR 0.84, 95% CI 0.75–0.94, *p* = 0.003). Additionally, PCSK/ST significantly reduced the risks of stroke when compared to CETP/ST, OMG3/ST, and NIA/ST (RR 0.74 with 95% CI 0.63–0.88, RR 0.74 with 95% CI 0.57–0.95, and RR 0.73 with 95% CI 0.61–0.87, respectively). Also, PCSK/ST was superior to CETP/ST in reducing the risk of coronary revascularization (RR 0.83 with 95% CI 0.71–0.96), see eTables 9.3–9.6. Results of SUCRA rank of these outcomes are listed in eTables 10.3–10.6. Based on these results along with SUCRA rank, PCSK/ST appeared to be the most efficacious regimen to reduce non-fatal MI and coronary revascularization compared to other NST.

For safety endpoints, the network maps were presented in eFigures 6.5, 6.6. The effects of treatments on all-cause discontinuation (58 studies, 236,043 participants) and discontinuation from any adverse event (56 studies, 209,532 participants) compared with ST were demonstrated in Figure 3. Only NIA/ST and NIA + EZT/ST showed a significant increase in the risk of all-cause discontinuation. Most NST significantly increased the risk of treatment discontinuations due to adverse events except PCSK/ST and EZT/ST compared with ST. Details of network estimates for safety endpoints of all treatment comparisons were presented in eTables 9.7, 9.8. A three-drug combination of NIA + EZT/ST was ranked the lowest for both safety endpoints (see eTables 10.7–10.8).

### Subgroup Analyses

We performed subgroup analyses in primary and secondary outcomes with regards to indication of treatment, intensity of statin therapy, requirement of statin prior to starting NST, level of cardiovascular risk, elderly, familial hypercholesterolemia (FH) and lipid level at baseline. Most effect estimates among subgroup analyses on the outcomes were relatively consistent with results in the main analyses (Appendix 12).

### Sensitivity Analyses and Publication Bias

We also performed sensitivity analyses by excluding studies with high risk of bias, non-adjudicated CV events, follow-up duration <1 year, and sample size <25 percentile. The effect estimates were generally robust among sensitivity analyses (Appendix 13). Comparison-adjusted funnel plots for all outcomes showed no evidence of asymmetry (Appendix 14). We also identified 7 studies registered in ClinicalTrials.gov but no published reports or results of those studies are available (Appendix 15). However, these trials were mostly small in size in comparison to the total study population. As a result, the chance for these trials to affect the main analysis is very low.

### Quality of Evidence

The quality of direct evidence for all outcomes was generally rated as moderate to high quality. When applying GRADE to network meta-analysis evidence, most comparison of interventions were rated as moderate quality for primary and secondary outcomes except safety endpoints as low quality. In addition, a better rating of quality of evidence for non-fatal MI was found. More details of their quality of evidence are presented in Appendix 16.

## Discussion

This network meta-analysis offers a single and comprehensive framework for comparison of efficacy and safety outcomes among various NST when added on to statin therapy in a broad range of patient populations. The results showed that none of these agents reduced the risk of CV death or all-cause death when compared with ST. Our findings suggested that PCSKs were the most efficacious agents when added on to statin therapy based on their ability to significantly reduce cardiovascular events including non-fatal MI, stroke and coronary revascularization. Such findings were robust and remain significant in various sensitivity and subgroup analyses. For safety aspects, the tolerability profile of PCSK/ST was similar to ST; therefore, such regimen appears to have a well-balanced efficacy and safety profile.

The reason of why NST did not reduce the risk of CV death and all-cause mortality may derive from several aspects including differences in mechanism of lipid-lowering actions, magnitude of LDL-C lowering effects along with trial design. Previously, a meta-analysis has shown that NST whose mechanisms of action relates to the upregulation of LDL-C receptor reduce CV events while those without this action did not (Silverman et al., 2016). As a result, mechanism of action may play a role in translating biochemical modification into clinical benefit. Trial design may partly explain the lack of mortality benefit of PCSK. A recent meta-analysis of 24 RCTs (Navarese et al., 2015) showed that PCSKs significantly reduced all-cause mortality. However, some of the included studies in the meta-analysis were without background statin therapy which is different from our study. Based on statin trials that demonstrated reduction in risk of mortality, the data showed that event curves started to diverge after 1.5–2.0 years [Scandinavian Simvastatin Survival Study Group, 1994; The Long-Term Intervention with Pravastatin in Ischaemic Disease (Lipid) Study Group, 1998]. Therefore, the duration of followed-up time might be an important factor. For CV outcome trials of PCSK9 inhibitors including FOURIER and ODESSEY Outcomes, the median follow-up time was 2.2 and 2.8 years, respectively (Sabatine et al., 2017; Schwartz et al., 2018). These may explain why the lack of reduction was seen in the trials of PCSK9 inhibitors in spite of dramatic reduction in LDL-C level compared with placebo (Sabatine et al., 2017; Schwartz et al., 2018). Of note, The ODYSSEY Outcomes trial, which had a longer follow-up, demonstrated significant reduction in mortality; however, it was a secondary endpoint of the trial (Schwartz et al., 2018).

For ezetimibe, we did not find significant effects of ezetimibe on clinical outcome in the overall analysis. Nevertheless, the results from the IMPROVE-IT trial showed that ezetimibe reduced non-fatal MI and ischemic stroke in ACS patients during the mean follow-up of 7 years (Cannon et al., 2015a). This may indicate that cardiovascular benefits of ezetimibe require a long period of exposure, potentially due to its modest LDL-C reduction effects. Since our analysis included studies of ezetimibe that were mostly run for no more than 2 years, inclusion of those trials therefore may dilute the effect of ezetimibe in our analysis. However, based on the subgroup and sensitivity analyses, ezetimibe reduced the risk of non-fatal MI and coronary revascularization in patients receiving moderate-intensity statins. Favorable tolerability profile, ease of use and affordability may make ezetimibe a viable option compared to PCSKs. Overall, this study lends a strong support toward the current clinical practice guideline that PCSKs and ezetimibe should be considered when patients failed to reach lipid goals or desired percentage reduction after maximally tolerated statin therapy has been deployed (Lloyd-Jones et al., 2017).

Similar to the results of previous RCTs of niacin and CETPs (Barter et al., 2007b; Boden et al., 2011; Schwartz et al., 2012; Landray et al., 2014; Lincoff et al., 2017), our analysis did not find any benefit of these agents on all CV outcomes of interest. Although previous epidemiological data have shown the association between low HDL-C and increased risk of cardiovascular disease (Barter et al., 2007b), a recent observational cohort study demonstrated that high level of HDL-C have not been associated with lowered risk of CV death (Ko et al., 2016). As a result, the hypothesis of using therapeutic agents to raise HDL-C may need to be carefully reexamined. Recently, anacetrapib, a CETP inhibitor, has been shown in HPS3/TIMI55–REVEAL trial to significantly reduce CV events. However, the effect was modest (Bowman et al., 2017) despite a doubling increase in HDL-C level. With a modest effect coupled with safety concern including blood pressure increase, reduced renal function along with prolonged accumulation of the drug in adipose tissue, this agent is later dropped from entering the market. For safety endpoints, both NIA/ST and CETP/ST were associated with higher risks of discontinuations compared with ST. In summary, these interventions did not demonstrate any benefit yet were associated with increased risk of adverse events, making it very difficult to justify their uses.

Fibrates and OMG3 are NST with predominant triglyceride-lowering effects. Based on our analysis, neither agent has demonstrable effects on clinical outcomes. Based on our inclusion criteria, all trials for fibrate included in our analysis used fenofibrate. The lack of effect in our analysis is consistent with findings from the ACCORD trial (Ginsberg et al., 2010). For OMG3, available evidence from 3 large RCTs are conflicting; with one positive and two neutral trials (Yokoyama et al., 2007; Kromhout et al., 2010; Rauch et al., 2010). Our main analysis showed that OMG3/ST was not superior to any NST or ST. Combination of these agents with statin was also associated with higher risks of discontinuations compared with ST. As a result, justification for use of these agents is quite limited.

The clinical benefit seen with PCSK and the lack of benefit among other therapies may partly be explained by two potential reasons including the magnitude of additional LDL-C lowering effects and the mechanism of LDL-lowering effect (Silverman et al., 2016). A recent two meta-analyses suggested that the risk of CV events was reduced by 19–23% per 1-mmol/L reduction in LDL-C level among ST and NST that reduced LDL-C via the upregulation of LDL-C receptor expression (including PCSK and ezetimibe) (Silverman et al., 2016; Koskinas et al., 2018). Our finding is consistent with their findings except ezetimibe where the inclusion of short-term trials may dilute the effect of ezetimibe as mentioned above.

While our study can be considered as the most comprehensive evaluation for NST, the heterogeneity of trials that came with data gathered for this analysis should be clearly declared and noted. Despite our best attempt with statistical analysis, conclusion drawn from our analysis is still far from being definitive. This stems from the fact that approximately one third of included trials were at high risk of bias while quality of evidence among included data were considered moderate. We therefore caution reader to consider this limitation when interpreting our results.

In addition to the key limitation mentioned above, several other limitations should be noted. First, bile acid sequestrant, mipomersen, lomitapide, or phytosterol were not included in the network meta-analysis. None of clinical studies of these agents met our inclusion criteria due to short follow-up duration, lack of background statin therapy or no reporting of outcomes of interest. Second, since we did not have access to individual patient data, we therefore were unable to perform analysis on composite endpoints such as the standard MACEs. Third, our subgroup analyses were based on aggregated data; consequently, contamination of each subgroup is possible. For certain subgroup, we were unable to compare all 8 interventions due to the lack of data of some interventions on certain subgroups. In addition, we were unable to perform an analysis on diabetes subgroup due to incomplete information for data extraction. Fourth, most studies included in the analysis did not use CV events as primary outcome and follow-up duration of these studies were generally not as long as large-scale clinical studies. Certain therapies may require very long duration of treatment before any effects can be seen. Lastly, although PCSK/ST showed acceptable tolerability in our analysis, this was derived from mostly short-term studies. As a result, long-term safety of this combination needs to be evaluated further. Despite these limitations, our analysis offers a useful comparative data on both efficacy and safety of various NST among statin-treated patients. Such information may be useful to guide clinical decision or formulate clinical practice guideline for dyslipidemia.

## Conclusion

In summary, our network meta-analysis suggested that none of NST significantly reduce the risk of CV death and all-cause death when added to moderate to high intensity statin therapy. However, PCSKs and to a lesser extent, ezetimibe may help reduce cardiovascular events with acceptable tolerability profile among broad range of patients. Fibrate, CETPs, niacin, and OMG3 did not show any positive effects on CV outcomes in broad range of high risk patients. Moreover, these agents when combined with statin were associated with higher incidence of adverse reactions. Further research into the risk-benefit along with cost-effectiveness analysis of these therapeutic options should be warranted.

## Transparency

The lead authors (TC, SN, NC) affirm that the manuscript is an honest, accurate, and transparent account of the study being reported; that no important aspects of the study have been omitted; and that any discrepancies from the study as planned have been explained.

## Author Contributions

TC, SN, and NC conceived and designed the study. TC wrote the protocol and the first draft. SN, NC, AT, CR, WW, and PV contributed to the writing of the manuscript. TC and PD screened, extracted the data, and performed the quality assessment and the quality of evidence. SN and NC had access to all the data in the study, analyzed the data and take responsibility for the integrity of the data and the accuracy of the data analysis.

### Conflict of Interest Statement

The authors declare that the research was conducted in the absence of any commercial or financial relationships that could be construed as a potential conflict of interest.
