# Supplementary material for: Effects of Non-statin Lipid-Modifying Agents on Cardiovascular Morbidity and Mortality Among Statin-Treated Patients: A Systematic Review and Network Meta-Analysis
Source: Front Pharmacol. 2019 May 22;10:547. doi: 10.3389/fphar.2019.00547 (PMC6540916; doi:10.3389/fphar.2019.00547)
Supplement: Supplementary file 1 [file Table_1.DOCX]

**Supplementary appendix**

**Effects of non-statin lipid-modifying agents on cardiovascular morbidity and mortality among statin-treated patients: A systematic review & network meta-analysis.**

Thanaputt Chaiyasothi, Surakit Nathisuwan, Piyameth Dilokthornsakul, Prin Vathesatogkit, Ammarin Thakkinstian, Christopher Reid, Wanwarang Wongcharoen, Nathorn Chaiyakunapruk

**Online Supplementary Content**

[Appendix 1 Search strategies 5](#_Toc6754787)

[eTable 1.1 search algorithm 5](#_Toc6754788)

[eFigure 1.1 Flow diagram and references of included studies 8](#_Toc6754789)

[Appendix 2 General characteristics of treatment options 9](#_Toc6754790)

[eTable 2.1 General characteristics of treatment options among included studies 9](#_Toc6754791)

[eTable 2.2 Intervention comparisons among included studies 9](#_Toc6754792)

[Appendix 3 Characteristic of included studies 10](#_Toc6754793)

[eTable 3.1 Description of included studies 10](#_Toc6754794)

[eTable 3.2 Description of participants of included studies 15](#_Toc6754795)

[eTable 3.3 Description of interventions of included studies 21](#_Toc6754796)

[Appendix 4 Risk of bias assessment 27](#_Toc6754797)

[eFigure 4.1 Risk of bias graph 27](#_Toc6754798)

[eTable 4.1 Risk of bias summary: judgements about each bias item for each study 28](#_Toc6754799)

[Appendix 5 Results of meta-analyses of direct comparisons of treatment options 31](#_Toc6754800)

[eTable 5.1 Pairwise meta-analyses risk ratio (and 95% CI) for all dichotomous outcomes 31](#_Toc6754801)

[Appendix 6 Network of comparisons 42](#_Toc6754802)

[eFigure 6.1 Networks map of treatment comparisons for Coronary heart disease mortality 42](#_Toc6754803)

[eFigure 6.2 Networks map of treatment comparisons for non-fatal myocardial infarction 42](#_Toc6754804)

[eFigure 6.3 Networks map of treatment comparisons for any stroke 43](#_Toc6754805)

[eFigure 6.4 Networks map of treatment comparisons for Coronary revascularization 43](#_Toc6754806)

[eFigure 6.5 Networks map of treatment comparisons for discontinuation due to any cause 44](#_Toc6754807)

[eFigure 6.6 Networks map of treatment comparisons for discontinuation due to adverse events 44](#_Toc6754808)

[Appendix 7 Assessment of inconsistency for each outcome network 45](#_Toc6754809)

[eTable 7.1 Evaluation of the global inconsistency in networks using the ‘design-by-treatment’ interaction model for each outcome. 45](#_Toc6754810)

[Appendix 8 Transitivity 46](#_Toc6754811)

[eTable 8.1 Explore distribution of covariables among treatment comparisons 46](#_Toc6754812)

[Appendix 9 Result of network meta-analysis 47](#_Toc6754813)

[eTable 9.1 Network estimated risk ratio (95% confidence intervals) of treatment options on cardiovascular mortality 47](#_Toc6754814)

[eTable 9.2 Network estimated risk ratio (95% confidence intervals) of treatment options on all-cause mortality 48](#_Toc6754815)

[eTable 9.3 Network estimated risk ratio (95% confidence intervals) of treatment options on coronary heart disease mortality 49](#_Toc6754816)

[eTable 9.4 Network estimated risk ratio (95% confidence intervals) of treatment options on non-fatal myocardial infarction 50](#_Toc6754817)

[eTable 9.5 Network estimated risk ratio (95% confidence intervals) of treatment options on any stroke 51](#_Toc6754818)

[eTable 9.6 Network estimated risk ratio (95% confidence intervals) of treatment options on coronary revascularization 52](#_Toc6754819)

[eTable 9.7 Network estimated risk ratio (95% confidence intervals) of treatment options on Discontinuation due to any cause 53](#_Toc6754820)

[eTable 9.8 Network estimated risk ratio (95% confidence intervals) of treatment options on Discontinuation due to any adverse event 54](#_Toc6754821)

[Appendix 10 Treatment ranking and surface under the cumulative ranking curves (SUCRA) for each outcome 55](#_Toc6754822)

[eFigure 10.1 SUCRA ranking curve for cardiovascular mortality 55](#_Toc6754823)

[eTable 10.1 SUCRA ranking for cardiovascular mortality 55](#_Toc6754824)

[eFigure 10.2 SUCRA ranking curve for all-cause mortality 56](#_Toc6754825)

[eTable 10.2 SUCRA ranking for cardiovascular mortality 56](#_Toc6754826)

[eFigure 10.3 SUCRA ranking curve for coronary heart disease mortality 57](#_Toc6754827)

[eTable 10.3 SUCRA ranking for coronary heart disease mortality 57](#_Toc6754828)

[eFigure 10.4 SUCRA ranking curve for non-fatal myocardial infarction 58](#_Toc6754829)

[eTable 10.4 SUCRA ranking for non-fatal myocardial infarction 58](#_Toc6754830)

[eFigure 10.5 SUCRA ranking curve for any stroke 59](#_Toc6754831)

[eTable 10.5 SUCRA ranking for any stroke 59](#_Toc6754832)

[eFigure 10.6 SUCRA ranking curve for coronary revascularization 60](#_Toc6754833)

[eTable 10.6 SUCRA ranking for coronary revascularization 60](#_Toc6754834)

[eFigure 10.7 SUCRA ranking curve for discontinuation due to any cause 61](#_Toc6754835)

[eTable 10.7 SUCRA ranking for discontinuation due to any cause 61](#_Toc6754836)

[eFigure 10.8 SUCRA ranking curve for discontinuation due to adverse events 62](#_Toc6754837)

[eTable 10.8 SUCRA ranking for discontinuation due to adverse events 62](#_Toc6754838)

[Appendix 11 The lists of included studies for the network meta-analysis of primary and secondary outcomes 63](#_Toc6754839)

[Appendix 12 Subgroup analyses 66](#_Toc6754840)

[12.1 Subgroup analyses of indication of therapy with treatment options 66](#_Toc6754841)

[eTable 12.1.1 the risk of cardiovascular mortality 66](#_Toc6754842)

[eTable 12.1.2 The risk of all-cause mortality 67](#_Toc6754843)

[eTable 12.1.3 The risk of coronary heart disease mortality 68](#_Toc6754844)

[eTable 12.1.4 The risk of non-fatal myocardial infarction 69](#_Toc6754845)

[eTable 12.1.5 The risk of any stroke 70](#_Toc6754846)

[eTable 12.1.6 The risk of coronary revascularization 71](#_Toc6754847)

[12.2 Subgroup analyses of intensity of statin with treatment options 72](#_Toc6754848)

[eTable 12.2.1 The risk of cardiovascular mortality 72](#_Toc6754849)

[eTable 12.2.2 The risk of all-cause mortality 73](#_Toc6754850)

[eTable 12.2.3 The risk of coronary heart disease mortality 74](#_Toc6754851)

[eTable 12.2.4 The risk of non-fatal myocardial infarction 75](#_Toc6754852)

[eTable 12.2.5 The risk of any stroke 76](#_Toc6754853)

[eTable 12.2.6 The risk of coronary revascularization 77](#_Toc6754854)

[12.3 Subgroup analyses of requirement of statin prior to starting non-statin lipid-lowering agent(s) with treatment options 78](#_Toc6754855)

[eTable 12.3.1 The risk of cardiovascular mortality 78](#_Toc6754856)

[eTable 12.3.2 The risk of all-cause mortality 79](#_Toc6754857)

[eTable 12.3.3 The risk of coronary heart disease mortality 80](#_Toc6754858)

[eTable 12.3.4 The risk of non-fatal myocardial infarction 81](#_Toc6754859)

[eTable 12.3.5 The risk of any stroke 82](#_Toc6754860)

[eTable 12.3.6 The risk of coronary revascularization 83](#_Toc6754861)

[12.4 Subgroup analyses of cardiovascular risk (CV) stratification with treatment options 84](#_Toc6754862)

[eTable 12.4.1 The risk of cardiovascular mortality 84](#_Toc6754863)

[eTable 12.4.2 The risk of all-cause mortality 85](#_Toc6754864)

[eTable 12.4.3 The risk of coronary heart disease mortality 86](#_Toc6754865)

[eTable 12.4.4 The risk of non-fatal myocardial infarction 87](#_Toc6754866)

[eTable 12.4.5 The risk of any stroke 88](#_Toc6754867)

[eTable 12.4.6 The risk of coronary revascularization 89](#_Toc6754868)

[12.5 Subgroup analyses of average age group with treatment options 90](#_Toc6754869)

[eTable 12.5.1 The risk of cardiovascular mortality 90](#_Toc6754870)

[eTable 12.5.2 The risk of All-cause mortality 91](#_Toc6754871)

[eTable 12.5.3 The risk of coronary heart disease mortality 92](#_Toc6754872)

[eTable 12.5.4 The risk of non-fatal myocardial infarction 93](#_Toc6754873)

[eTable 12.5.5 The risk of any stroke 94](#_Toc6754874)

[eTable 12.5.6 The risk of coronary revascularization 95](#_Toc6754875)

[12.6 Subgroup analyses of Familial hypercholesterolemia (FH) groups with treatment options 96](#_Toc6754876)

[eTable 12.6.1 The risk of cardiovascular mortality 96](#_Toc6754877)

[eTable 12.6.2 The risk of All-cause mortality 97](#_Toc6754878)

[eTable 12.6.3 The risk of coronary heart disease mortality 98](#_Toc6754879)

[eTable 12.6.4 The risk of non-fatal myocardial infarction 99](#_Toc6754880)

[eTable 12.6.5 The risk of any stroke 100](#_Toc6754881)

[eTable 12.6.6 The risk of coronary revascularization 101](#_Toc6754882)

[12.7 Subgroup analyses of baseline lipid level (LDL-C and Non-HDL-C level) 102](#_Toc6754883)

[eTable 12.7.1 The risk of cardiovascular mortality 102](#_Toc6754884)

[eTable 12.7.2 The risk of All-cause mortality 103](#_Toc6754885)

[eTable 12.7.3 The risk of coronary heart disease mortality 104](#_Toc6754886)

[eTable 12.7.4 The risk of non-fatal myocardial infarction 105](#_Toc6754887)

[eTable 12.7.5 The risk of any stroke 106](#_Toc6754888)

[eTable 12.7.6 The risk of coronary revascularization 107](#_Toc6754889)

[12.8 Subgroup analyses of baseline lipid level (HDL-C and Triglyceride level) 108](#_Toc6754890)

[eTable 12.8.1 the risk of cardiovascular mortality 108](#_Toc6754891)

[eTable 12.8.2 The risk of all-cause mortality 109](#_Toc6754892)

[eTable 12.8.3 The risk of coronary heart disease mortality 110](#_Toc6754893)

[eTable 12.8.4 The risk of non-fatal myocardial infarction 111](#_Toc6754894)

[eTable 12.8.5 The risk of any stroke 112](#_Toc6754895)

[eTable 12.8.6 The risk of coronary revascularization 113](#_Toc6754896)

[Appendix 13 Sensitivity analyses 114](#_Toc6754897)

[eTable 13.1 Sensitivity analyses for the risk of cardiovascular mortality with treatment options 114](#_Toc6754898)

[eTable 13.2 Sensitivity analyses for the risk of all-cause mortality with treatment options 115](#_Toc6754899)

[eTable 13.3 Sensitivity analyses for the risk of coronary heart disease mortality with treatment options 116](#_Toc6754900)

[eTable 13.4 Sensitivity analyses for the risk of non-fatal myocardial infarction with treatment options 117](#_Toc6754901)

[eTable 13.5 Sensitivity analyses for the risk of any stoke with treatment options 118](#_Toc6754902)

[eTable 13.6 Sensitivity analyses for the risk of coronary revascularization with treatment options 119](#_Toc6754903)

[Appendix 14 Comparison-adjusted funnel plot for each outcome form the network meta-analyses 120](#_Toc6754904)

[eFigure 14.1 Comparison-adjusted funnel plot for the network of cardiovascular mortality 120](#_Toc6754905)

[eFigure 14.2 Comparison-adjusted funnel plot for the network of all-cause mortality 120](#_Toc6754906)

[eFigure 14.3 Comparison-adjusted funnel plot for the network of coronary heart disease mortality 121](#_Toc6754907)

[eFigure 14.4 Comparison-adjusted funnel plot for the network of non-fatal myocardial infarction 121](#_Toc6754908)

[eFigure 14.5 Comparison-adjusted funnel plot for the network of any stroke 122](#_Toc6754909)

[eFigure 14.6 Comparison-adjusted funnel plot for the network of coronary revascularization 122](#_Toc6754910)

[eFigure 14.7 Comparison-adjusted funnel plot for the network of discontinuation due to any cause 123](#_Toc6754911)

[eFigure 14.8 Comparison-adjusted funnel plot for the network of discontinuation due to adverse events 123](#_Toc6754912)

[Appendix 15 Unpublished studies registered in ClinicalTrials.gov 124](#_Toc6754913)

[Appendix 16 Grading 125](#_Toc6754914)

[Appendix 17 eReferences 133](#_Toc6754915)

Appendix 1 Search strategies

eTable 1.1 search algorithm

| **Database** | **Step** | **Keyword** | **Item found** |
| --- | --- | --- | --- |
| PubMed | #1 | Ezetimibe OR "cholesterol absorption" OR “Niemann-Pick C1-like 1” OR NPC1L1 | 4,726 |
|  | #2 | Omega-3 OR "fish oil" OR "Omega-3 fatty acid" OR "n-3 fatty acid" OR "Alpha-Linolenic acid" OR "eicosapentaenoic acid" OR "docosahexaenoic acid" | 35,496 |
|  | #3 | Fibrate OR “fibric acid” OR Fenofibrate OR Gemfibrozil OR Bezafibrate OR Ciprofibrate OR Clofibrate OR Clinofibrate | 11,714 |
|  | #4 | “Nicotinic acid” OR niacin OR acipimox | 16,575 |
|  | #5 | "Bile acid sequestrant" OR resin OR Cholestyramine OR Colestipol OR Colesevelam | 67,547 |
|  | #6 | “Proprotein convertase subtilisin/kexin” OR “Proprotein convertase subtilisin kexin” OR “Proprotein convertase subtilisin-kexin” OR PCSK9 OR alirocumab OR evolocumab | 2,658 |
|  | #7 | "Cholesteryl ester transfer protein" OR CETP OR Torcetrapib OR Dalcetrapib OR Anacetrapib OR Evacetrapib | 3,548 |
|  | #8 | “Microsomal triglyceride transfer protein” OR “microsomal transfer protein” OR MTP OR Lomitapide | 3,475 |
|  | #9 | “Antisense oligonucleotide” OR “Apoprotein B-100” OR “apo B-100” OR Mipomersen | 5,038 |
|  | #10 | Phytosterol OR "plant sterol" OR monacolin OR "red yeast rice" OR "dietary fiber" OR “soy protein” OR policosanol OR berberine | 25,567 |
|  | #11 | Non-statin OR nonstatin OR "non statin" | 623 |
|  | #12 | (#1 OR #2 OR #3 OR #4 OR #5 OR #6 OR #7 OR #8 OR #9 OR #10 OR #11) | 109,484 |
|  | #13 | Statin OR "3-hydroxy-3-methylglutaryl coenzyme-A" OR "HMG-CoA" OR atorvastatin OR simvastatin OR pravastatin OR fluvastatin OR rosuvastatin OR pitavastatin OR lovastatin | 50,944 |
|  | #14 | Cardiovascular OR cerebrovascular OR cardiac OR coronary OR heart OR vascular OR “myocardial infarction” OR “unstable angina” OR stroke OR death OR mortality OR fatal OR arterial OR artery OR “peripheral artery” OR “peripheral arterial” OR event | 4,753,872 |
|  | #15 | (#12 AND #13 AND #14) | 4,634 |
| Embase | #1 | Ezetimibe OR "cholesterol absorption" OR “Niemann-Pick C1-like 1” OR NPC1L1 | 12,040 |
|  | #2 | Omega-3 OR "fish oil" OR "Omega-3 fatty acid" OR "n-3 fatty acid" OR "Alpha-Linolenic acid" OR "eicosapentaenoic acid" OR "docosahexaenoic acid" | 58,336 |
|  | #3 | Fibrate OR “fibric acid” OR Fenofibrate OR Gemfibrozil OR Bezafibrate OR Ciprofibrate OR Clofibrate OR Clinofibrate | 32,763 |
|  | #4 | “Nicotinic acid” OR niacin OR acipimox | 30,661 |
|  | #5 | "Bile acid sequestrant" OR resin OR Cholestyramine OR Colestipol OR Colesevelam | 93,651 |
|  | #6 | “Proprotein convertase subtilisin/kexin” OR “Proprotein convertase subtilisin kexin” OR “Proprotein convertase subtilisin-kexin” OR PCSK9 OR alirocumab OR evolocumab | 4,573 |
|  | #7 | "Cholesteryl ester transfer protein" OR CETP OR Torcetrapib OR Dalcetrapib OR Anacetrapib OR Evacetrapib | 5,387 |
|  | #8 | “Microsomal triglyceride transfer protein” OR “microsomal transfer protein” OR MTP OR Lomitapide | 5,586 |
|  | #9 | “Antisense oligonucleotide” OR “Apoprotein B-100” OR “apo B-100” OR Mipomersen | 19,875 |
|  | #10 | Phytosterol OR "plant sterol" OR monacolin OR "red yeast rice" OR "dietary fiber" OR “soy protein” OR policosanol OR berberine | 39,626 |
|  | #11 | Non-statin OR nonstatin OR "non statin" | 989 |
|  | #12 | #1 OR #2 OR #3 OR #4 OR #5 OR #6 OR #7 OR #8 OR #9 OR #10 OR #11 | 15,921 |
|  | #13 | Statin OR "3-hydroxy-3-methylglutaryl coenzyme-A" OR "HMG-CoA" OR atorvastatin OR simvastatin OR pravastatin OR fluvastatin OR rosuvastatin OR pitavastatin OR lovastatin | 98,608 |
|  | #14 | Cardiovascular OR cerebrovascular OR cardiac OR coronary OR heart OR vascular OR “myocardial infarction” OR “unstable angina” OR stroke OR death OR mortality OR fatal OR arterial OR artery OR “peripheral artery” OR “peripheral arterial” OR event | 6,069,281 |
|  | #15 | #12 AND #13 AND #14 | 13,960 |
| CENTRAL | #1 | Ezetimibe OR "cholesterol absorption" OR “Niemann-Pick C1-like 1” OR NPC1L1 | 937 |
|  | #2 | Omega-3 OR "fish oil" OR "Omega-3 fatty acid" OR "n-3 fatty acid" OR "Alpha-Linolenic acid" OR "eicosapentaenoic acid" OR "docosahexaenoic acid" | 5,399 |
|  | #3 | Fibrate OR “fibric acid” OR Fenofibrate OR Gemfibrozil OR Bezafibrate OR Ciprofibrate OR Clofibrate OR Clinofibrate | 1,927 |
|  | #4 | “Nicotinic acid” OR niacin OR acipimox | 1,477 |
|  | #5 | "Bile acid sequestrant" OR resin OR Cholestyramine OR Colestipol OR Colesevelam | 4,802 |
|  | #6 | “Proprotein convertase subtilisin/kexin” OR “Proprotein convertase subtilisin kexin” OR “Proprotein convertase subtilisin-kexin” OR PCSK9 OR alirocumab OR evolocumab | 208 |
|  | #7 | "Cholesteryl ester transfer protein" OR CETP OR Torcetrapib OR Dalcetrapib OR Anacetrapib OR Evacetrapib | 282 |
|  | #8 | “Microsomal triglyceride transfer protein” OR “microsomal transfer protein” OR MTP OR Lomitapide | 180 |
|  | #9 | “Antisense oligonucleotide” OR “Apoprotein B-100” OR “apo B-100” OR Mipomersen | 180 |
|  | #10 | Phytosterol OR "plant sterol" OR monacolin OR "red yeast rice" OR "dietary fiber" OR “soy protein” OR policosanol OR berberine | 3,244 |
|  | #11 | Non-statin OR nonstatin OR "non statin" | 86 |
|  | #12 | #1 OR #2 OR #3 OR #4 OR #5 OR #6 OR #7 OR #8 OR #9 OR #10 OR #11 | 17,642 |
|  | #13 | Statin OR "3-hydroxy-3-methylglutaryl coenzyme-A" OR "HMG-CoA" OR atorvastatin OR simvastatin OR pravastatin OR fluvastatin OR rosuvastatin OR pitavastatin OR lovastatin | 9,688 |
|  | #14 | Cardiovascular OR cerebrovascular OR cardiac OR coronary OR heart OR vascular OR “myocardial infarction” OR “unstable angina” OR stroke OR death OR mortality OR fatal OR arterial OR artery OR “peripheral artery” OR “peripheral arterial” OR event | 298,357 |
|  | #15 | #12 AND #13 AND #14 | 1,538 |
| Clinicaltrial.gov | 1 | Ezetimibe OR "cholesterol absorption" OR “Niemann-Pick C1-like 1” OR NPC1L1 | 343 |
|  | 2 | Omega-3 OR "fish oil" OR "Omega-3 fatty acid" OR "n-3 fatty acid" OR "Alpha-Linolenic acid" OR "eicosapentaenoic acid" OR "docosahexaenoic acid" | 1,442 |
|  | 3 | Fibrate OR “fibric acid” OR Fenofibrate OR Gemfibrozil OR Bezafibrate OR Ciprofibrate OR Clofibrate OR Clinofibrate | 258 |
|  | 4 | “Nicotinic acid” OR niacin OR acipimox | 989 |
|  | 5 | "Bile acid sequestrant" OR resin OR Cholestyramine OR Colestipol OR Colesevelam | 362 |
|  | 6 | “Proprotein convertase subtilisin/kexin” OR “Proprotein convertase subtilisin kexin” OR “Proprotein convertase subtilisin-kexin” OR PCSK9 OR alirocumab OR evolocumab | 163 |
|  | 7 | "Cholesteryl ester transfer protein" OR CETP OR Torcetrapib OR Dalcetrapib OR Anacetrapib OR Evacetrapib | 117 |
|  | 8 | “Microsomal triglyceride transfer protein” OR “microsomal transfer protein” OR MTP OR Lomitapide | 104 |
|  | 9 | “Antisense oligonucleotide” OR “Apoprotein B-100” OR “apo B-100” OR Mipomersen | 159 |
|  | 10 | Phytosterol OR "plant sterol" OR monacolin OR "red yeast rice" OR "dietary fiber" OR “soy protein” OR policosanol OR berberine | 2,689 |
|  | 11 | Non-statin OR nonstatin OR "non statin" | 16 |
|  | 12 | #1 OR #2 OR #3 OR #4 OR #5 OR #6 OR #7 OR #8 OR #9 OR #10 OR #11 | 6,684 |
|  | #13 | Statin OR "3-hydroxy-3-methylglutaryl coenzyme-A" OR "HMG-CoA" OR atorvastatin OR simvastatin OR pravastatin OR fluvastatin OR rosuvastatin OR pitavastatin OR lovastatin | 2,461 |
|  | #14 | Cardiovascular OR cerebrovascular OR cardiac OR coronary OR heart OR vascular OR “myocardial infarction” OR “unstable angina” OR stroke OR death OR mortality OR fatal OR arterial OR artery OR “peripheral artery” OR “peripheral arterial” OR event | 107,898 |
|  | #15 | #12 AND #13 AND #14 | 376 |

eFigure 1.1 Flow diagram and references of included studies

**394 full-text articles** assessed for eligibility

**5,091 Duplicate records** removed

**15,417 records** screened by title and abstract

**15,030 records excluded**

5,148 unrelated study

2,781 letter/editorial/conference abstract

/commentary

5,991 review/systemic review/meta-

analysis/case report/pooled analysis

631 no statin background/same non-

statin/single arm

300 follow-up duration < 24 weeks

197 non-randomized studies

**326 articles excluded**

33 no statin background/no non-statin/

Single arm/same non-statin

193 no outcomes of interest

5 non-randomized studies

92 sub-analysis/substudy of the included trials

3 follow-up duration < 24 weeks

**Screening**

**Eligibility**

**7 additional studies** from reference lists

**67 studies** included in network meta-analysis

(259,429 participants)

**68 studies** included in qualitative review

**1 article excluded**

- reported only a composite of outcomes of

interest but no data for individual outcomes

**Included**

**20,508 records** identified through database searching in April 2018

PubMed 4,634

Embase 13,960

Cochrane 1,538

Clinicaltrial.gov 376

**Identification**

Appendix 2 General characteristics of treatment options

eTable 2.1 General characteristics of treatment options among included studies

| **Treatment abbreviation** | **General characteristics** |
| --- | --- |
| ST | Statin monotherapy   - atorvastatin (ATV), rosuvastatin (RSV), simvastatin (SMV), pravastatin (PRV), fluvastatin (FLV), and pitavastatin (PTV) |
| CETP/ST | Cholesteryl ester transfer protein inhibitor plus statin   - CETP including torcetrapib (TORC), dalcetrapib (DALC), evacetrapib (EVAC, and ancetrapib (ANAC) |
| EZT/ST | Ezetimibe plus statin |
| FBT/ST | Fibrate + statin   - Fibrates including fenofibrate (FENO) or fibric acid (FA) |
| NIA/ST | Niacin plus statin |
| OMG3/ST | Omega-3 fatty acids plus statin   - OMG3 including eicosapentaenoic acid (EPA) and docosahexaenoic acid (DHA) |
| PCSK/ST | Proprotein convertase subtilisin/kexin type 9 inhibitor plus statin   - PCSK including alirocumab, (ALI) evolocumab (EVO), and Bococizumab (BOC) |
| NIA+EZT/ST | Niacin plus ezetimibe plus statin |

eTable 2.2 Intervention comparisons among included studies

| **No. of study** | **Intervention comparisons** | | |
| --- | --- | --- | --- |
|  | Intervention 1 | Intervention 2 | Intervention 3 |
| 15 | CETP/ST | vs ST | - |
| 15 | EZT/ST | vs ST | - |
| 3 | FBT/ST | vs ST | - |
| 6 | NIA/ST | vs ST | - |
| 8 | OMG3/ST | vs ST | - |
| 15 | PCSK/ST | vs ST | - |
| 1 | NIA+EZT/ST | vs ST | - |
| 1 | NIA/ST | vs EZT/ST | - |
| 1 | PCSK/ST | vs EZT/ST | - |
| 1 | NIA+EZT/ST | vs EZT/ST | - |
| 2 | PCSK/ST | vs EZT/ST | vs ST |

Appendix 3 Characteristic of included studies

eTable 3.1 Description of included studies

| Author | Published  Year | Study name | Treatment | N | Male (%) | **Target population** | Indication of treatment | Follow-up Duration (month) | Blinding | **Adjudicated CV event** |
| --- | --- | --- | --- | --- | --- | --- | --- | --- | --- | --- |
| Cannon [1] | 2015 | IMPROVE-IT | SMV (40 mg/d) EZT(10 mg/d) + SMV (40 mg/d) | 18,144 | 75.7 | ACS | Secondary | 72 | Double-blind | Yes |
| Kastelein [2] | 2008 | ENHANCE | SMV (80 mg/d)  EZT (10 mg/d) + SMV (80 mg/d) | 720 | 51.4 | FH | Mixed | 24 | Double-blind | No |
| Taylor [3] | 2004 | ARBIRTER2 | Any ST NIA (1000 mg/d) + any ST | 167 | 91.0 | CHD | Secondary | 12 | Double-blind | NR |
| Boden [4] | 2011 | AIM-HIGH | SMV (40-80 mg/d) NIA (1500-2000 mg/d) + SMV (40-80 mg/d) | 3,414 | 85.2 | ASCVD | Secondary | 36 | Double-blind | Yes |
| Landray [5] | 2014 | HPS2-THRIVE | SMV (40 mg) NIA/LRP (2000/40 mg/d) + SMV (40 mg/d) | 25,673 | 82.7 | ASCVD | Secondary | 46.8 | Double-blind | Yes |
| Ginsberg [6] | 2010 | ACCORD | SMV (20-40 mg/d) FENO (160 mg/d) + SMV (20-40 mg/d) | 5,518 | 69.3 | DM | Mixed | 56.4 | Double-blind | Yes |
| Yokoyama [7] | 2007 | JELIS | PRV (10-20 mg/d) or SMV (5-10 mg/d) EPA (1800 mg/d) + PRV (10-20 mg/d) or SMV (5-10 mg/d) | 18,645 | 31.5 | HC | Mixed | 55.2 | Open-label | Yes |
| Nissen [8] | 2007 | ILLUSTRATE | Any ATV TORC (60 mg/d) + any ATV | 1,188 | 70.5 | Coronary stenosis by angiography | Mixed | 24 | Double-blind | Yes |
| Barter [9] | 2007 | ILLUMINATE | Any ATV TORC (60 mg/d) + any ATV | 15,067 | 77.8 | ASCVD or DM | Mixed | 18.3 | Double-blind | Yes |
| Schwartz [10] | 2012 | dal-OUTCOME | Any ST DALC (600 mg/d) + any ST | 15,871 | 80.5 | Recent ACS | Secondary | 31 | Double-blind | Yes |
| Robinson [11] | 2015 | ODYSSEY- LONG TERM | Any ST ALI (150 mg SC q 2 wk) + any ST | 2,341 | 61.8 | HeFH or CHD or high risk CHD | Mixed | 18 | Double-blind | Yes |
| Sabatine [12] | 2015 | OSLER | Any ST EVO (140 mg SC q 2 wk or 420 mg SC q 1 mo) + any ST | 4,465 | 50.8 | Hyperlipidemia | Mixed | 11.1 | Open-label | Yes |
| Taylor [13] | 2009 | ARBIRTER6 | EZT (10 mg/d) + any ST NIA (2000 mg/d) + any ST | 363 | 80.2 | CHD or CHD risk equivalents | Mixed | 14 | Open-label | Yes |
| Guyton [14] | 2008 | No | NIA (2000 mg/d) EZT (10 mg/d) + SMV (20 mg/d) NIA (2000 mg/d) +EZT(10 mg/d) + SMV (20 mg/d) | 1,220 | 50.1 | IIa or IIb hyperlipidemia | Mixed | 6 | Double-blind | No |
| Brunner [15] | 2013 | ELIMIT | SMV (40 mg/d) NIA (1500 mg/d) + EZT (10 mg/d) + SMV (40 mg/d) | 95 | 93.7 | PAD | Secondary | 24 | Double-blind | No |
| Ballantyne [16] | 2008 | SEACOAST I | SMV (20 mg/d) NIA (1000 mg/d) + SMV (20 mg/d) NIA (2000 mg/d) + SMV (20 mg/d) | 314 | 50.6 | Mixed hyperlipidemia | Mixed | 6 | Double-blind | NR |
| Wang [17] | 2016 | No | RSV (10 mg/d) EZT (10 mg/d) + RSV (10 mg/d) | 106 | 72.5 | CHD | Secondary | 12 | NR | NR |
| Tsujita [18] | 2015 | PRECISE-IVUS | Any ATV EZT (10 mg/d) + any ATV | 246 | 78.0 | ACS or SA | Secondary | 9.9 | Open-label | NR |
| Masuda [19] | 2015 | No | RSV (5 mg/d) EZT (10 mg/d) + RSV (5 mg/d) | 51 | 87.5 | SAP with PCI | Secondary | 6 | Open-label | NR |
| Luo [20] | 2014 | No | ATV (20 mg/d) EZT (10 mg/d + ATV (20 mg/d) | 84 | 52.3 | HC | Mixed | 12 | NR | NR |
| Rauch [21] | 2010 | OMEGA | Any ST EPA-DHA (460/380 mg/d) + any ST | 3,851 | 74.4 | Acute MI | Secondary | 12 | Double-blind | Yes |
| Farnier [22] | 2016 | ODYSSEY OPTIONS II | RSV (40 mg/d) EZT (10 mg/d) + RSV (20 mg/d) ALI (75-150 mg SC q 2 wk) + RSV (20 mg/d) RSV (20 mg/d) EZT (10) + RSV(10 mg/d) ALI (75-150 SC mg q 2 wk) + RSV (10 mg/d) | 305 | 61.3 | HC with high or very high CV risk | Mixed | 6 | Double-blind | Yes |
| Cannon [23] | 2015 | ODYSSEY COMBO II | EZT(10 mg/d) + any ST ALI(75-150 mg SC q 2 wk) + any statin | 720 | 73.6 | CHD or CHD risk equivalents | Mixed | 12 | Double-blind | Yes |
| Blom [24] | 2014 | DESCARTES | ATV (10 or 80 mg/d) EVO (420 mg SC q 1 mo) + ATV (10 or 80 mg/d) | 901 | 47.7 | HC | Mixed | 12 | Double-blind | Yes |
| West [25] | 2011 | No | SMV (40 mg/d) EZT (10 mg/d) + SMV (40 mg/d) | 44 | 62.5 | PAD | Secondary | 24 | Double-blind | NR |
| Arimura [26] | 2012 | No | ATV (10 mg/d) EZT (10 mg/d) + ATV (10 mg/d) | 44 | 70.5 | SA with stent | Secondary | 8.4 | NR | NR |
| Bots [27] | 2007 | RADIANCE 2 | Any ATV TORC (60 mg/d) + any ATV | 752 | 64.0 | Mixed dyslipidemia | Primary | 20 | Double-blind | No |
| Kastelein [28] | 2015 | REALIZE | Any ST ANAC (100 mg/d) + any ST | 306 | 54.0 | HeFH | Primary | 12 | Double-blind | Yes |
| Cannon [29] | 2010 | DEFINE | Any ST ANAC (100 mg/d) + any ST | 1,623 | 77.4 | CHD or CHD risk equivalents | Mixed | 17.5 | Double-blind | Yes |
| Davidson [30] | 2014 | FIRST | Any ATV (10-40 mg/d) FA (135 mg/d) +any ATV (10-40 mg/d) | 682 | 68.0 | Dyslipidemia CHD or CHD risk equivalents | Mixed | 24 | Double-blind | Yes |
| Kastelein [31] | 2007 | RADIANCE 1 | Any ATV TORC (60 mg/d) + any ATV | 904 | 49.4 | HeFH | Primary | 24 | Double-blind | No |
| Derosa [32] | 2004 | No | FLV (80 mg/d) FENO (200 mg/d) + FLV (80 mg/d) | 48 | 50.1 | DM with CHD | Secondary | 12 | Double-blind | NR |
| Durrington [33] | 2001 | No | SMV (10-40 mg/d) EPA-DHA (1760/1440 mg/d) + SMV (10-40 mg/d) | 59 | 72.9 | CHD with high TG | Secondary | 6 | Double-blind | NR |
| Kastelein [34] | 2015 | ODYSSEY FH I & II | Any ST ALI (75-150 mg SC q 2 wk) + any statin | 735 | 55.1 | HeFH | Mixed | 18 | Double-blind | Yes |
| Kereiakes [35] | 2015 | ODYSSEY COMBO I | Any statin ALI (75-150 mg SC q 2 wk) + any statin | 316 | 65.8 | CHD or CHD risk equivalent | Mixed | 12 | Double-blind | Yes |
| Nishio [36] | 2014 | No | Any RSV EPA (1800 mg/d) + any RSV | 31 | 86.7 | PCI with SA/ACS | Secondary | 9 | Open-label | NR |
| Teramoto [37] | 2016 | ODYSSEY JAPAN | Any ST ALI (75-150 mg SC q 2 wk) + any statin | 216 | 60.6 | HeFH/HC with CHD/CHD risk equivalents | Mixed | 12 | Double-blind | Yes |
| Stein [38] | 2010 | No | Any ATV DALC (900 mg/d) + any ATV | 135 | 78.5 | CHD or CHD risk equivalents | Mixed | 12 | Double-blind | NR |
| Bays [39] | 2015 | ODYSSEY OPTIONS I | RSV (40 mg/d) ATV (80 mg/d) EZT (10 mg/d) + ATV (40 mg/d) ALI (75-150 mg SC q 2 wk) + ATV (40 mg/d) ATV (40 mg/d) EZT (10 mg/d) + ATV (20 mg/d) ALI (75-150 mg SC q 2 wk) + ATV (20 mg/d) | 355 | 65.1 | HC with high or very high CV risk | Mixed | 8 | Double-blind | Yes |
| Fayad [40] | 2011 | dal-PLAUQE | Any ST DALC (600 mg/d) + any ST | 130 | 81.5 | CHD or CHD risk equivalents | Mixed | 24 | Double-blind | Yes |
| Luscher [41] | 2012 | dal-VESSEL | Any ST DALC (600 mg/d) + any ST | 476 | 90.5 | CHD or CHD risk equivalents | Mixed | 8.3 | Double-blind | Yes |
| Ballantyne [42] | 2008 | SEACOAST II | SMV (80 mg/d) NIA (1000 mg/d) + SMV (40) NIA (2000 mg/d) + SMV (40) | 343 | 54.5 | Dyslipidemia (non-HDL-C) | Mixed | 6 | Double-blind | NR |
| Nicholls [43] | 2016 | GLAGOV | Any ST EVO (420 mg SC q 1 mo) + any ST | 970 | 72.2 | Coronary stenosis by angiography with CVD risk | Mixed | 18 | Double-blind | Yes |
| Landray [44] | 2006 | UK-HARP-II | SMV (20 mg/d) EZT (10 mg/d) + SMV (20 mg/d) | 203 | 69.5 | CKD | Mixed | 6 | Double-blind | NR |
| Shaw [45] | 2009 | No | Any ST EZT (10 mg/d) + any ST | 68 | 84.5 | Cardiac transplant treated with cyclosporine | Primary | 6 | Double-blind | Yes |
| Kouvelos [46] | 2013 | No | RSV (10 mg/d) EZT (10 mg/d) + any ST | 262 | 89.7 | Patient with vascular surgery | Secondary | 12 | Open-label | NR |
| Ginsberg [47] | 2016 | ODYSSEY HIGH FH | Any ST ALI (150 mg SC q 2 wk) + any ST | 107 | 53.3 | HeFH | Mixed | 18 | Double-blind | Yes |
| Ballantyne [48] | 2017 | No | Any ST ANAC (100 mg/d) + any ST ANAC (25 mg/d) + any ST | 459 | 67.7 | Hypercholesterolemia | Mixed | 6 | Double-blind | Yes |
| Kromhout [49] | 2010 | ALPHA OMEGA | Any ST + ALA(2,000 mg) EPA-DHA (400 mg/d) +any ST+ ALA (2,000 mg) | 4,837 | 78.2 | History of MI | Secondary | 40.8 | Double-blind | Yes |
| Sabatine [50] | 2017 | FOURIER | Any ST  EVO (140 mg SC q 2wk or 420 mg SC q 1 mo) + any ST | 27,564 | 75.4 | ASCVD | Secondary | 26 | Double-blind | Yes |
| Ridker [51] | 2017 | SPIRE-1 | Any ST  BOC (150 mg SC q 2 wk) + any statin | 16,817 | 70.4 | ASCVD or high CV risk | Mixed | 7 | Double-blind | Yes |
|  |  | SPIRE-2 | Any ST  BOC (150 mg SC q 2 wk) + any statin | 10,621 | 73.6 | ASCVD or high CV risk | Mixed | 12 | Double-blind | Yes |
| Ridker [52] | 2017 | SPIRE-HR | Any ST  BOC (150 mg SC q 2 wk) + any statin | 711 | 61.3 | ASCVD or CHD risk equivalents | Mixed | 12 | Double-blind | Yes |
|  |  | SPRIE-LDL | Any ST  BOC (150 mg SC q 2 wk) + any statin | 2,139 | 62 | ASCVD or CHD risk equivalents or multiple CV risk factors | Mixed | 12 | Double-blind | Yes |
|  |  | SPIRE-FH | Any ST  BOC (150 mg SC q 2 wk) + any statin | 370 | 56.1 | HeFH | Mixed | 12 | Double-blind | Yes |
|  |  | SPIRE-LL | Any ST  BOC (150 mg SC q 2 wk) + any statin | 746 | 61.6 | ASCVD or CHD risk equivalents or multiple CV risk factors | Mixed | 12 | Double-blind | Yes |
|  |  | SPIRE-SI | Placebo  ATV (40)  BOC (150 mg SC q 2 wk) | 184 | 63.9 | Statin intolerant | Mixed | 6 | Double-blind | Yes |
|  |  | SPIRE-AI | Any ST  BOC (75 mg SC q 2 wk) (autoinjection) + any ST  Any ST  BOC (150 mg SC q 2 wk) (autoinjection) + any ST | 299 | 60 | Hyperlipidemia | Mixed | 3 | Double-blind | Yes |
| Luo [53] | 2016 | No | ATV (20 mg/d)  EZT (10 mg/d) + ATV(20 mg/d) | 148 | 56.8 | CHD | Secondary | 12 | Double-blind | NR |
| Liu [54] | 2017 | No | ATV (20 mg/d)  EZT (10 mg/d) + ATV(10 mg/d) | 230 | 51.7 | ACS | Secondary | 12 | NR | NR |
| Nosaka [55] | 2017 | No | PTV (2 mg/d)  EPA(1800 mg/d) + PTV(2 mg/d) | 241 | 75.5 | ACS with PCI | Secondary | 12 | Open-label | Yes |
| Lincoff [56] | 2017 | ACCELERATE | Any ST  EVAC (130 mg/d) + any ST | 12,092 | 77 | ASCVD | Secondary | 26 | Double-blind | Yes |
| Bowman [57] | 2017 | REVEAL | Any ATV  ANAC (100 mg/d) + any ATV | 30,449 | 83.9 | ASCVD | Secondary | 49.2 | Double-blind | Yes |
| Hagiwara [58] | 2017 | HIJ-PROPER | PTV (2 mg/d)  EZT (10 mg/d) + PTV(2 mg/d) | 1,734 | 75.5 | ACS | Secondary | 46.3 | Open-label | Yes |
| Hibi [59] | 2018 | No | PTV (2 mg/d)  EZT (10 mg/d) + PTV(2 mg/d) | 128 | 80 | ACS | Secondary | 10 | Open-label | NR |
| Miyoshi [60] | 2018 | No | PTV (2 mg/d)  PTV (4 mg/d)  EPA (1,800 mg/d) + PTV(2 mg/d) | 198 | 55 | Hypercholesterolemia | Primary | 12 | Open-label | Yes |
| Watanabe [61] | 2017 | CHERRY | PTV (4 mg/d)  EPA (1,800 mg/d) + PTV(4 mg/d) | 241 | 82 | CAD (SA or ACS) with PCI and hypercholesterolemia | Secondary | 8 | Double-blind | Yes |
| Koh [62] | 2018 | ODYSSEY KT | Any ST  ALI (75-150 mg SC q 2 wk) + any ST | 199 | 82.5 | High CV risk | Combined | 6 | Double-blind | Yes |
| Leiter [63] | 2017 | ODYSSEY DM-INSULIN | Any ST  ALI (75-150 mg SC q 2 wk) + any ST | 517 | 55.1 | T1DM or T2DM (treated with insulin) with ASCVD and/or CV risk factor(s) | Combined | 6 | Double-blind | NR |
| Teramoto [64] | 2017 | No | Any ST  ANAC (100 mg/d) + any ST | 307 | 67.8 | Dyslipidemia | Combined | 6 | Double-blind | Yes |
| Ballantyne [65] | 2017 | No | Any ST  ANAC (100 mg/d) + any ST | 583 | 72.7 | Dyslipidemia | Combined | 6 | Double-blind | Yes |
| Ray [66] | 2018 | ODYSSEY DM-DYSLIPIDEMIA | Any ST (± LLT)  ALI (75-150 mg SC q 2 wk) + any ST | 413 | 52.3 | T2DM and mixed dyslipidemia | Combined | 6 | Open-label | NR |
| Schwartz [67] | 2018 | ODYSSEY Outcomes | Any ST  ALI (75-150 mg SC q 2 wk) + any ST | 18,924 | 74.8 | ACS | Secondary | 33.6 | Double-blind | Yes |
| Sang^a^ [68] | 2009 | No | ATV (10 mg/d) NIA (1000 mg/d) + ATV(10 mg/d) | 108 | 61.1 | CAD | Secondary | 12 | NR | NR |

^a^The study did not included in network meta-analysis

**Abbreviation:** ACS=Acute coronary syndrome; ASCVD=Atherosclerotic cardiovascular disease; CAD=coronary artery disease; CHD=coronary heart disease; CKD=chronic kidney disease; DM=diabetes mellitus; FH=Familial hypercholesterolemia; HeFH=Heterozygous familial hypercholesterolemia; HC=Hypercholesterolemia; MI=myocardial infarction; NR = no report; PAD=Peripheral artery disease; PCI=percutaneous coronary intervention SA=stable angina; SAP=stable angina pectoris; LLT=lipid-lowering therapy; mg=milligram; SC=subcutaneous

eTable 3.2 Description of participants of included studies

| Author | Published  year | Treatment | (N) | Age (SD or percentile) | Previous CHD (%) | Previous MI (%) | Previous stroke (%) | DM (%) | HTN (%) | FH (%) | Smoking (%) | BMI (SD or percentile) (kg/m^2^) | LDL (mg/dl) | HDL (mg/dl) | Non-HDL (mg/dl) | TG (mg/dl) |
| --- | --- | --- | --- | --- | --- | --- | --- | --- | --- | --- | --- | --- | --- | --- | --- | --- |
| Cannon [1] | 2015 | ST | 9,077 | 63.6 (9.8) | NR | 20.7 | NR | 27.3 | 61.3 | NR | 33.5 | 28.3 (5.2) | 93.8 | 42.2 | 120.5 | 137.5 |
|  |  | EZT/ST | 9,067 | 63.6 (9.7) | NR | 21.3 | NR | 27.1 | 61.6 | NR | 32.5 | 28.3 (5.2) | 93.8 | 42.1 | 120.5 | 137.6 |
| Kastelein [2] | 2008 | ST | 363 | 45.7 (10) | NR | 7.2 | NR | 1.4 | 14 | 100 | 28.7 | 26.7 (4.4) | 317.8 | 47.4 | 352.6 | 160 |
|  |  | EZT/ST | 357 | 46.1 (9) | NR | 3.9 | NR | 2.2 | 18 | 100 | 28.6 | 27.4 (4.6) | 319 | 46.7 | 353.3 | 157 |
| Taylor [3] | 2004 | ST | 80 | 68 (10) | NR | 52.5 | NR | 27.5 | 76.3 | NR | 6.3 | NR | 91 | 40 | 121 | 172 |
|  |  | NIA/ST | 87 | 67 (10) | NR | 49.4 | NR | 27.6 | 73.6 | NR | 13.8 | NR | 87 | 39 | 115 | 154 |
| Boden [4] | 2011 | ST | 1,696 | 63.7 (8.7) | NR | 56.3 | 21.3 | 33.6 | 70.1 | NR | NR | NR | 74 | 34.9 | 110.3 | 163 |
|  |  | NIA/ST | 1,718 | 63.7 (8.8) | NR | 56.3 | 20.8 | 34.2 | 72.8 | NR | NR | NR | 74.2 | 34.5 | 110.8 | 167.5 |
| Landray [5] | 2014 | ST | 12,835 | 64.9 (7.5) | NR | 67.5 | 32 | 32.5 | 62.4 | NR | 18.1 | NR | 63 | 44 | 84 | 105 |
|  |  | NIA/ST | 12,838 | 64.9 (7.5) | NR | 67.7 | 31.6 | 32.2 | 62.5 | NR | 17.9 | NR | 64 | 43.9 | 84.1 | 100 |
| Ginsberg [6] | 2010 | ST | 2,753 | 62.3 (6.9) | NR | NR | NR | 100 | NR | NR | 14.3 | 32.4 (5.4) | 101.1 | 38.2 | 137.5 | 160 |
|  |  | FBT/ST | 2,765 | 62.2 (6.7) | NR | NR | NR | 100 | NR | NR | 14.8 | 32.2 (5.4) | 100 | 38 | 136.7 | 164 |
| Yokoyama [7] | 2007 | ST | 9,319 | 61 (9) | NR | 5 | NR | 16 | 35 | NR | 18 | 24 (3) | 181.7 | 58.4 | 216.5 | 154.1 |
|  |  | OMG3/ST | 9,326 | 61 (8) | NR | 6 | NR | 16 | 36 | NR | 20 | 24 (3) | 181.4 | 58.8 | 216.1 | 153.2 |
| Nissen [8] | 2007 | ST | 597 | 57 (9.2) | NR | NR | NR | 22.3 | 77.6 | NR | 18.8 | 30.3 (5.2) | 84.3 | 45.2 | 112.3 | 123.9 |
|  |  | CETP/ST | 591 | 56.9 (9.1) | NR | NR | NR | 22.1 | 74.5 | NR | 17.3 | 30.6 (6.2) | 83.1 | 46 | 111.7 | 122 |
| Barter [9] | 2007 | ST | 7,534 | 61.3 (7.6) | NR | 45.3 | 5.5 | 45.2 | 73.9 | NR | 13.9 | 30.2 (5.6) | 79.9 | 48.5 | 108.8 | 128 |
|  |  | CETP/ST | 7,533 | 61.3 (7.6) | NR | 46.2 | 5.2 | 43.5 | 72.3 | NR | 13.4 | 30.1 (5.7) | 79.7 | 48.6 | 108.2 | 127 |
| Schwartz [10] | 2012 | ST | 7,933 | 60.1 (9.1) | NR | 15 | 3 | 25 | 68 | NR | 21 | 28.6 (5.1) | 75.8 | 42.2 | 102.4 | 134.2 |
|  |  | CETP/ST | 7,938 | 60.3 (9.1) | NR | 16 | 3 | 24 | 67 | NR | 21 | 28.6 (5) | 76.4 | 42.5 | 103.2 | 133 |
| Robinson [11] | 2015 | ST | 788 | 60.6 (10.4) | 70.1 | NR | NR | 33.9 | NR | 17.6 | 20.2 | 30.5 (5.5) | 121.9 | 50 | 152 | 135 |
|  |  | PCSK/ST | 1,553 | 60.4 (10.4) | 67.9 | NR | NR | 34.9 | NR | 17.8 | 20.9 | 30.2 (5.7) | 122.7 | 49.8 | 152.6 | 132 |
| Sabatine [12] | 2015 | ST | 1,489 | 58.2 (10.9) | 20.6 | 9.5 | 2.5 | 14.6 | 52.2 | 10.1 | 14.9 | NR | 121 | 51 | 154 | 119 |
|  |  | PCSK/ST | 2,976 | 57.8 (11) | 19.8 | 9.3 | 2.7 | 12.8 | 51.9 | 9.7 | 15.6 | NR | 120 | 51 | 151 | 120 |
| Taylor [13] | 2009 | EZT/ST | 176 | 65 (11) | NR | 33.5 | NR | 40.9 | 87.5 | NR | 5.1 | 30.8 (5.6) | 83.4 | 43.3 | 103.3 | 122 |
|  |  | NIA/ST | 187 | 65 (10) | NR | 28.9 | NR | 39 | 87.2 | NR | 7 | 31.3 (6.4) | 80.5 | 42.5 | 103.1 | 126 |
| Guyton [14] | 2008 | NIA | 272 | 56.4 (10.6) | 5.9 | NR | NR | 14.7 | NR | NR | NR | 30.1 (5.9) | 157.1 | 50.5 | 191 | 148 |
|  |  | EZT/ST | 272 | 57.5 (10.3) | 8.1 | NR | NR | 15.8 | NR | NR | NR | 30.3 (6) | 155.4 | 49.9 | 190.8 | 158 |
|  |  | NIA+EZT/ST | 676 | 56.9 (10.9) | 8.9 | NR | NR | 15.5 | NR | NR | NR | 29.8 (5.5) | 156.9 | 50.5 | 190.9 | 159 |
| Brunner [15] | 2013 | ST | 48 | 63.9 (7.1) | NR | NR | NR | 39.6 | 85.4 | NR | 35.4 | 31.4 (7.3) | 96 | 41.5 | 125.5 | 146.5 |
|  |  | NIA+EZT/ST | 47 | 62.1 7.8) | NR | NR | NR | 42.6 | 80.9 | NR | 42.6 | 30.9 (8) | 88 | 39 | 120 | 134 |
| Ballantyne [16] | 2008 | ST | 121 | 58 (32-76) | NR | NR | NR | 19 | 69 | NR | 19.4 | 28-28.7 | 114.8 | 43 | 156.5 | 208.5 |
|  |  | NIA/ST | 127 | 56 (30-81) | NR | NR | NR | 22.3 | 67.9 | NR | 19.4 | 28.1 | 117.5 | 41.3 | 164.5 | 209.5 |
|  |  | NIA/ST | 66 | 55 (34-75) | NR | NR | NR | 22.3 | 67.9 | NR | 19.4 | 28.1 | 111.5 | 40.9 | 159.8 | 214.3 |
| Wang [17] | 2016 | ST | 51 | 65 (12) | 100 | NR | NR | 35 | 48 | NR | 60 | NR | 134.6 | 43.7 | 172.1 | 168.3 |
|  |  | EZT/ST | 55 | 63 (10) | 100 | NR | NR | 36 | 50 | NR | 62 | NR | 140 | 43.7 | 174.8 | 174.5 |
| Tsujita [18] | 2015 | ST | 124 | 67 (10) | NR | 13 | 1 | 30 | 66 | NR | 32 | 24.9 (3.1) | 108.3 | 40 | 132.7 | 116 |
|  |  | EZT/ST | 122 | 66 (10) | NR | 15 | 10 | 29 | 75 | NR | 20 | 24.8 (3.4) | 109.8 | 41.1 | 136.2 | 114 |
| Masuda [19] | 2015 | ST | 25 | 70.2 (7.6 | NR | 21.1 | NR | 42.1 | 89.5 | NR | 21.1 | 23.8 (2) | 123 | 47.1 | 146.2 | 144.9 |
|  |  | EZT/ST | 26 | 64 (7.9) | NR | 23.8 | NR | 52.4 | 61.9 | NR | 42.9 | 24.7 (4.3) | 131.8 | 53.1 | 151.4 | 129.7 |
| Luo [20] | 2014 | ST | 44 | 66.3 (5.8) | 77 | NR | NR | 36.3 | NR | NR | NR | 24.7 (4.4) | 128 | 45.6 | 181.8 | 208.1 |
|  |  | EZT/ST | 40 | 67.2 (6.4) | 90 | NR | NR | 30 | NR | NR | NR | 24.4 (4.6) | 126.5 | 45.2 | 177.2 | 201.9 |
| Rauch [21] | 2010 | ST | 1,911 | 64 (54-72) | NR | 13.5 | 5.1 | 26.7 | 66.1 | NR | 37.5 | NR | NR | NR | NR | NR |
|  |  | OMG3/ST | 1,940 | 64 (54-72) | NR | 15.3 | 5.8 | 27.6 | 66.9 | NR | 35.9 | NR | NR | NR | NR | NR |
| Farnier [22] | 2016 | ST | 48 | 60.6 (10.1) | 67.9 | NR | NR | 32.1 | 79.2 | 6.7 | NR | 31.5 (6.7) | 108.6 | 46.9 | 143.6 | 143 |
|  |  | EZT/ST | 48 | 63.1 (10.2) | 60.4 | NR | NR | 39.6 | 67.9 | 5.4 | NR | 30.2 (5.4) | 115.2 | 52.2 | 149 | 143 |
|  |  | PCSK/ST | 49 | 57.9 (8.9) | 59.3 | NR | NR | 33.3 | 74.1 | 11.1 | NR | 30.2 (6.0) | 114.1 | 51.8 | 145.8 | 116 |
|  |  | ST | 53 | 61.5 (11.1) | 52.1 | NR | NR | 58.3 | 70.8 | 6.2 | NR | 32 (6.2) | 100.2 | 48.5 | 136.9 | 130.5 |
|  |  | EZT/ST | 53 | 60.4 (10.4) | 60.4 | NR | NR | 47.9 | 68.8 | 7.3 | NR | 32.1 (7.3) | 94.7 | 51 | 131.7 | 127 |
|  |  | PCSK/ST | 54 | 62.2 (10) | 46.9 | NR | NR | 38.8 | 73.5 | 16.3 | NR | 31.8 (7.7) | 106 | 49.4 | 138 | 116 |
| Cannon [23] | 2015 | EZT/ST | 241 | 61.3 (9.2) | 88 | 57.7 | 8.3 | 31.5 | NR | NR | NR | 30.3 (5.1) | 104.4 | 46.4 | 135.3 | 141.7 |
|  |  | PCSK/ST | 479 | 61.7 (9.4) | 91.2 | 57.8 | 8.4 | 30.7 | NR | NR | NR | 30 (5.4) | 108.3 | 46.4 | 139.2 | 132.9 |
| Blom [24] | 2014 | ST | 302 | 56.7 (10.1) | 13.9 | NR | NR | 13.9 | 49.3 | NR | 15.9 | 30.5 (5.9) | 100.2 | 53.5 | 125.6 | 110.3 |
|  |  | PCSK/ST | 599 | 55.9 (10.8) | 15.7 | NR | NR | 10.4 | 48.2 | NR | 14.5 | 29.9 (6.1) | 100.4 | 52.6 | 124.2 | 105 |
| West [25] | 2011 | ST | 22 | 59 (10) | 50 | NR | NR | 31 | 81 | NR | 50 | 30 (7) | 118 | 45 | 149 | 227 |
|  |  | EZT/ST | 22 | 62 (8) | 56 | NR | NR | 28 | 78 | NR | 72 | 28 (6) | 118 | 48 | 141 | 130 |
| Arimura [26] | 2012 | ST | 22 | 69 (8) | 100 | 27 | NR | 27 | 68 | NR | 59 | 22.5 (2.9) | 105 | 51 | 130 | 125 |
|  |  | EZT/ST | 22 | 69 (9) | 100 | 27 | NR | 32 | 82 | NR | 59 | 23.6 (2.4) | 105 | 50 | 130 | 130 |
| Bots [27] | 2007 | ST | 375 | 56.5 (8.2) | NR | NR | NR | 25 | 49 | NR | 15 | 30 (4.4) | 100.5 | 47.6 | 136.5 | 165.6 |
|  |  | CETP/ST | 377 | 57.9 (8.1) | NR | NR | NR | 18 | 51 | NR | 17 | 30 (4.3) | 100.5 | 47.6 | 137.2 | 167.4 |
| Kastelein [28] | 2015 | ST | 102 | 55.7 (11.9) | 12 | 17 | NR | 6 | 39 | 100 | NR | 27.9 (4.7) | 135.3 | 54 | 154.4 | 106.3 |
|  |  | CETP/ST | 204 | 55 (11.8) | 12 | 12 | NR | 5 | 30 | 100 | NR | 28.2 (4.2) | 131.5 | 54 | 150.6 | 97.4 |
| Cannon [29] | 2010 | ST | 812 | 62.5 (8.7) | 54.3 | 22.4 | NR | 53 | 66.6 | NR | NR | 30.1 (5.2) | 82.2 | 40.4 | 111.1 | 128 |
|  |  | CETP/ST | 811 | 62.9 (9) | 55.1 | 22.8 | NR | 53.2 | 69.1 | NR | NR | 30.4 (5.5) | 81.2 | 40.5 | 109.7 | 127 |
| Davidson [30] | 2014 | ST | 342 | 61 (NR) | 26 | 12 | 7 | 50 | 81 | NR | 25 | NR | 84.5 | 39.6 | 118.2 | 193 |
|  |  | FBT/ST | 340 | 61 (NR) | 19 | 10 | 6 | 50 | 77 | NR | 21 | NR | 84 | 40.1 | 117.8 | 205 |
| Kastelein [31] | 2007 | ST | 450 | 45.2 (12.9) | NR | NR | NR | 4.2 | 25.1 | 100 | 20.9 | 26.7 (4.4) | 138.9 | 51.8 | 161.7 | 97.4 |
|  |  | CETP/ST | 454 | 46.8 (12) | NR | NR | NR | 2.7 | 24.4 | 100 | 19.1 | 26.7 (4.3) | 138.4 | 52.9 | 160.1 | 97.4 |
| Derosa [32] | 2004 | ST | 23 | 59 (6) | 100 | 100 | NR | 100 | NR | NR | 21.7 | 25.3 (1.8) | 186 | 42 | 218 | 150 |
|  |  | FBT/ST | 25 | 61 (5) | 100 | 100 | NR | 100 | NR | NR | 28 | 25.2 (1.7) | 191 | 41 | 224 | 161 |
| Durrington [33] | 2001 | ST | 29 | 54.8 (10.2) | 100 | NR | NR | 24.1 | NR | NR | NR | 28.4 (4.2) | 162.4 | 42.5 | 197.3 | 336.6 |
|  |  | OMG3/ST | 30 | 55.2 (10.2) | 100 | NR | NR | 26.7 | NR | NR | NR | 28.8 (2.8) | 135.3 | 42.5 | 174.1 | 407.4 |
| Kastelein [34] | 2015 | ST | 82 | 53.2 (12.5) | 37.8 | 17.1 | 1.2 | 3.7 | 29.3 | 100 | 15.9 | 27.7 (4.7) | 140.9 | 50.1 | 165.6 | 123.4 |
|  | (ODYSSEY FH I ) | PCSK/ST | 167 | 53.2 (12.9) | 34.7 | 16.2 | 3 | 4.2 | 34.1 | 100 | 21.6 | 28.6 (4.6) | 141.3 | 51.4 | 166.5 | 126.7 |
|  | 2015 | ST | 163 | 51.7 (12.3) | 47.9 | 26.4 | 1.8 | 15.3 | 43.6 | 100 | 18.4 | 30 (5.4) | 140.9 | 50.1 | 165.6 | 123.4 |
|  | (ODYSSEY FH II) | PCSK/ST | 323 | 52.1 (12.3) | 45.5 | 22 | 4 | 9.9 | 43 | 100 | 12.1 | 29 (4.6) | 141.3 | 51.4 | 166.5 | 126.7 |
| Kereiakes [35] | 2015 | ST | 107 | 63.0 (8.8) | 77.6 | NR | NR | 39.3 | NR | NR | NR | 32.03 (7.1) | 100.2 | 48.8 | 133.4 | 123 |
|  |  | PCSK/ST | 209 | 63.0 (9.5) | 78.5 | NR | NR | 45 | NR | NR | NR | 32.62 (6.3) | 94.8 | 48.3 | 130 | 130 |
| Nishio [36] | 2014 | ST | 15 | 63.8 (9.5) | NR | NR | NR | 13.3 | 66.7 | NR | 60 | 24.6 (3.5) | 130.3 | 41.5 | 154.8 | 146.8 |
|  |  | OMG3/ST | 16 | 61 (12.6) | NR | NR | NR | 33.3 | 73.3 | NR | 80 | 26.4 (3.6) | 138 | 40.9 | 166.4 | 161.4 |
| Teramoto [37] | 2016 | ST | 72 | 61.8 (9) | NR | 5.6 | 1.4 | 59.7 | NR | 19.4 | NR | 25.4 (3.2) | 143.1 | 54.1 | 169.9 | 132.9 |
|  | 2016 | PCSK/ST | 144 | 60.3 (9.7) | NR | 8.3 | 1.4 | 72.9 | NR | 18.8 | NR | 25.6 (4.3) | 143.1 | 54.1 | 169.9 | 124 |
| Stein [38] | 2010 | ST | 46 | 60.2 (7.5) | 41 | NR | NR | 54 | 65 | NR | 9 | 30.1 (5.6) | 76.9 | 41 | 103.2 | 131.7 |
|  | 2010 | CETP/ST | 89 | 61.2 (7.76) | 55 | NR | NR | 54 | 73 | NR | 16 | 30.5 (4.7) | 76.9 | 41.4 | 105.9 | 150.2 |
| Bays [39] | 2015 | ST | 45 | 57.5 (10) | 48.9 | NR | NR | 40 | 73.3 | NR | NR | 30.8 (6.9) | 106.2 | 50 | 140 | 116 |
|  |  | ST | 47 | 63.2 (10.9) | 66 | NR | NR | 53.5 | 78.7 | NR | NR | 30.2 (6) | 104.4 | 47.9 | 136.2 | 122 |
|  |  | EZT/ST | 47 | 63.9 (10.3) | 74.5 | NR | NR | 34 | 78.7 | NR | NR | 30.8 (5.9) | 94.7 | 46.5 | 124.5 | 113 |
|  |  | PCSK/ST | 47 | 64.2 (10.4) | 70.2 | NR | NR | 53.2 | 76.6 | NR | NR | 29.8 (5.4) | 110.5 | 49.3 | 144.5 | 120 |
|  |  | ST | 57 | 63 (9.9) | 50.9 | NR | NR | 54.4 | 80.7 | NR | NR | 31.4 (6.8) | 98 | 51.2 | 129.7 | 126 |
|  |  | EZT/ST | 55 | 65.7 (9) | 50.9 | NR | NR | 52.7 | 81.8 | NR | NR | 31.6 (6) | 96.7 | 48.9 | 128.6 | 124 |
|  |  | PCSK/ST | 57 | 62.2 (10) | 38.6 | NR | NR | 57.9 | 77.2 | NR | NR | 32.2 (7.7) | 101.3 | 47.2 | 131.4 | 134 |
| Fayad [40] | 2011 | ST | 66 | 64.6 (7.8) | 82 | NR | NR | 30 | 73 | NR | 12 | 29.8 (6.2) | 73.5 | 46.4 | 100.5 | 132.9 |
|  |  | CETP/ST | 64 | 62.6 (8.2) | 89 | NR | NR | 30 | 73 | NR | 14 | 29.6 (4.8) | 73.5 | 42.5 | 100.6 | 124 |
| Luscher [41] | 2012 | ST | 237 | 61.9 (7.92) | 66 | NR | 8 | 44 | 75 | NR | 24 | 28.7 (4.4) | 79.3 | 38.5 | 108.5 | 146.5 |
|  |  | CETP/ST | 239 | 62.3 (7.05) | 63 | NR | 7 | 47 | 74 | NR | 28 | 29.6 (4.8) | 81.5 | 39.2 | 113.4 | 161.1 |
| Ballantyne [42] | 2008 | ST | 123 | 60 (36-86) | NR | NR | NR | 34.1 | 64.2 | NR | 16.3 | 28.6  (26-33) | 98.5 | 46.5 | 130.5 | 140.5 |
|  |  | NIA/ST | 118 | 62 (32-83) | NR | NR | NR | 39.8 | 63.6 | NR | 13.6 | 29.4  (26.8-33.8) | 105 | 44.3 | 135.5 | 147.3 |
|  |  | NIA/ST | 102 | 59 (42-82) | NR | NR | NR | 35.3 | 67.6 | NR | 15.7 | 29.4  (26.3-32.9) | 109 | 46.3 | 143 | 155.5 |
| Nicholls [43] | 2016 | ST | 486 | 59.8 (8.8) | NR | 35.5 | NR | 21.5 | 83.7 | NR | 23.3 | 29.5 (5) | 92.4 | 45.2 | 120.8 | 124.5 |
|  |  | PCSK/ST | 484 | 59.8 (9.6) | NR | 34.9 | NR | 20.2 | 82.2 | NR | 25.6 | 29.4 (5) | 92.2 | 46.7 | 119.4 | 117 |
| Landray [44] | 2006 | ST | 101 | 60 (15) | NR | NR | NR | 10 | NR | NR | NR | 27.5 (5.5) | 117 | 40 | 155 | 188 |
|  |  | EZT/ST | 102 | 60 (14) | NR | NR | NR | 12 | NR | NR | NR | 27.1 (6.4) | 121 | 40 | 158 | 167 |
| Shaw [45] | 2009 | ST | 34 | 57 (10) | NR | NR | NR | NR | NR | NR | NR | NR | 108.2 | 65.7 | 143.1 | 186 |
|  |  | EZT/ST | 34 | 52 (14) | NR | NR | NR | NR | NR | NR | NR | NR | 112.1 | 58 | 146.9 | 194.8 |
| Kouvelos [46] | 2013 | ST | 136 | 72 (46-88) | 49.3 | NR | NR | 43.4 | 80.9 | NR | NR | NR | 143 | 41.3 | 198 | 160.2 |
|  | 2013 | EZT/ST | 126 | 70 (41-89) | 49.2 | NR | NR | 42.1 | 81.7 | NR | NR | NR | 148 | 40.9 | 202.4 | 155 |
| Ginsberg [47] | 2016 | ST | 35 | 52.1 (11.2) | 62.9 | NR | NR | 17.1 | 60 | 100 | 25.7 | 28.9 (4.2) | 201 | 44.9 | 231.5 | 122 |
|  |  | PCSK/ST | 72 | 49.8 (14.2) | 43.1 | NR | NR | 12.5 | 55.6 | 100 | 16.7 | 28.8 (5.2) | 196.3 | 49.6 | 223.9 | 131.5 |
| Ballantyne [48] | 2017 | ST | 154 | 60.3 (8.9) | 11 | 15.6 | NR | 50.6 | 74 | No | NR | NR | 93 | 47.7 | 124 | 155 |
|  |  | CETP/ST | 153 | 58.7 (9.6) | 17 | 22.9 | NR | 48.4 | 71.9 | No | NR | NR | 95.7 | 46.2 | 128.7 | 165 |
|  |  | CETP/ST | 152 | 61.9 (9.1) | 21.7 | 17.8 | NR | 58.6 | 70.4 | No | NR | NR | 96.9 | 45.3 | 128.4 | 157.5 |
| Kromhout [49] | 2010 | ST | 2,433 | 69 (5.6) | 100 | 100 | 7 | 20.9 | NR | NR | 17.7 | 27.8 (3.9) | 100 | 49.5 | 134.2 | 149.7 |
|  |  | OMG3/ST | 2,404 | 69.1 (5.6) | 100 | 100 | 7.3 | 21.1 | NR | NR | 15.9 | 27.8 (3.8) | 101.2 | 49.9 | 133 | 144.4 |
| Sabatine [50] | 2017 | ST | 13,780 | 62.5 (8.9) | NR | 81.3 | 19.2 | 36.5 | 80.1 | NR | 28.5 | NR | 92 | 44 | 124 | 133 |
|  |  | PCSK/ST | 13,784 | 62.5 (9.1) | NR | 80.9 | 19.5 | 36.7 | 80.1 | NR | 28 | NR | 92 | 44 | 124 | 134 |
| Ridker [51] | 2017 | ST | 8,409 | 63.3 (9.2) | NR | NR | NR | 47.4 | 80.9 | 1.8 | 23 | NR | 93.7 | 47.4 | 114 | 124.8 |
|  | (SPIRE-1) | PCSK/ST | 8,408 | 63.3 (9.1) | NR | NR | NR | 48.3 | 81.2 | 1.7 | 22.8 | NR | 93.8 | 47.1 | 114.3 | 124.3 |
|  | 2017 | ST | 5,309 | 62.6 (9.5) | NR | NR | NR | 46.1 | 79.6 | 7.6 | 26.6 | NR | 133.4 | 47.4 | 160.2 | 154 |
|  | (SPIRE-2) | PCSK/ST | 5,312 | 62.2 (9.6) | NR | NR | NR | 47.8 | 81.3 | 7 | 27.7 | NR | 133.9 | 47.3 | 160.5 | 157.1 |
| Ridker [52] | 2017 | ST | 354 | 61.5 (NR) | NR | NR | NR | 50 | 82.8 | 6.2 | 17.2 | NR | 114.3 | 48.8 | 137.2 | 138 |
|  | (SPIRE-HR) | PCSK/ST | 357 | 61.1 (NR) | NR | NR | NR | 48.7 | 85.2 | 8.1 | 15.1 | NR | 115.5 | 48.9 | 140.1 | 136.3 |
|  | 2017 | ST | 1,071 | 62.2 (NR) | NR | NR | NR | 62.9 | 82.4 | 2.4 | 19.7 | NR | 113.5 | 48.3 | 138 | 147.4 |
|  | (SPRIE-LDL) | PCSK/ST | 1,068 | 61.8 (NR) | NR | NR | NR | 62.9 | 82.9 | 1.3 | 18.4 | NR | 110.9 | 47.8 | 135.3 | 147.5 |
|  | 2017 | ST | 185 | 55.7 (NR) | NR | NR | NR | 17.8 | 54.6 | 100 | 19.5 | NR | 150 | 48.6 | 178.6 | 131.9 |
|  | (SPIRE-FH) | PCSK/ST | 185 | 56.5 (NR) | NR | NR | NR | 22.7 | 49.2 | 100 | 17.8 | NR | 144.2 | 50.3 | 170.4 | 118.5 |
|  | 2017 | ST | 247 | 61.7 (NR) | NR | NR | NR | 59.5 | 77.3 | 7.7 | 18.6 | NR | 134.7 | 49.2 | 160.2 | 159.5 |
|  | (SPIRE-LL) | PCSK/ST | 499 | 61.5 (NR) | NR | NR | NR | 54.9 | 77.6 | 6.6 | 23.8 | NR | 135.9 | 48.2 | 162.1 | 170.5 |
|  | 2017 | Placebo | 73 | 63.2 (NR) | NR | NR | NR | 21.9 | 63 | 9.6 | 12.3 | NR | 182.1 | 51.1 | 210.7 | 172 |
|  | (SPIRE-SI) | ST | 37 | 65.1 (NR) | NR | NR | NR | 21.6 | 70.3 | 18.9 | 2.7 | NR | 169.9 | 55.7 | 200.5 | 142 |
|  |  | PCSK | 74 | 64 (NR) | NR | NR | NR | 28.4 | 77 | 8.1 | 5.4 | NR | 168.8 | 50.9 | 199.2 | 166 |
|  | 2017 | ST | 49 | 61 (NR) | NR | NR | NR | 46.9 | 71.4 | 0 | 24.5 | NR | 116.1 | 53.4 | 127 | 119 |
|  | (SPIRE-AI) | ST | 50 | 61.5 (NR) | NR | NR | NR | 44 | 80 | 2 | 8 | NR | 107.9 | 49.8 | 134.4 | 123.5 |
|  |  | PCSK/ST | 100 | 59.9 (NR) | NR | NR | NR | 45 | 78 | 1 | 14 | NR | 111.4 | 47.4 | 128.2 | 121.8 |
|  |  | PCSK/ST | 100 | 58.9 (NR) | NR | NR | NR | 42 | 69 | 2 | 17 | NR | 112.3 | 47.5 | 130.3 | 118.8 |
| Luo [53] | 2017 | ST | 74 | 61.6 (9.7) | 100 | NR | 16.2 | 40.5 | 51.4 | NR | 40.5 | 24.7 (5.4) | 136.1 | 46 | 185.2 | 226.7 |
|  |  | EZT/ST | 74 | 60.8 (11.6) | 100 | NR | 18.9 | 45.9 | 48.6 | NR | 35.1 | 25.2 (4.8) | 138.1 | 45.2 | 182.2 | 219.7 |
| Liu [54] | 2017 | ST | 114 | 84 (1.8) | NR | 14.7 | 13.8 | 36.2 | 69 | NR | 13.8 | 25.4 (3.9) | 88.9 | 50.3 | NR | 141.7 |
|  | 2017 | EZT/ST | 116 | 84.2 (2.9) | NR | 19.3 | 12.3 | 40.4 | 71.1 | NR | 11.4 | 25.6 (3.5) | 85.1 | 46.4 | NR | 132.9 |
| Nosaka [55] | 2017 | ST | 121 | 71(12) | NR | 6 | 13 | 34 | 69 | NR | 32 | 24 (3.6) | 118 | 43 | 139 | 105 |
|  |  | OMG3/ST | 120 | 70 (11) | 19.3 | 12 | 7 | 43 | 71 | NR | 28 | 23.8 (3.9) | 116 | 43 | 142 | 117 |
| Lincoff [56] | 2017 | ST | 6,054 | 65.0 (9.5) | NR | 60.1 | 11.7 | 67.9 | 87.6 | NR | 15.7 | NR | 81.1 | 45.3 | 106.7 | 128 |
|  |  | CETP/ST | 6,038 | 64.8 (9.4) | NR | 60 | 12.1 | 68.4 | 87.3 | NR | 16.6 | NR | 81.6 | 45.3 | 107.2 | 128 |
| Bowman [57] | 2017 | ST | 15,225 | 67 (8) | 87.7 | NR | 22.3 | 37.2 | NR | NR | NR | 28.6 (5.1) | 61 | 40 | 92 | NR |
|  |  | CETP/ST | 15,224 | 67 (8) | 87.5 | NR | 22.2 | 37.1 | NR | NR | NR | 28.6 (5.0) | 61 | 40 | 92 | NR |
| Hagiwara [58] | 2017 | ST | 865 | 65.5 (11.9) | NR | 7.9 | 5.7 | 30.3 | 67.2 | 0 | 35 | 24.3 (3.6) | 135.6 | 48.3 | 162.5 | 132.5 |
|  |  | EZT/ST | 869 | 65.7 (11.7) | NR | 7.2 | 6.5 | 30.1 | 69.3 | 0 | 34 | 24.3 (3.5) | 134.8 | 49 | 161 | 129.1 |
| Hibi [59] | 2018 | ST | 63 | 63 (12) | NR | NR | 38 | 21 | 64 | NR | 38 | NR | 126 | 46 | 150 | 112 |
|  |  | EZT/ST | 65 | 63 (10) | NR | NR | 44 | 20 | 46 | NR | 44 | NR | 123 | 45 | 146 | 109 |
| Miyoshi [60] | 2018 | ST | 64 | 67 (9) | NR | NR | NR | 29 | 95 | 0 | 15 | 25 (4) | NR | NR | NR | NR |
|  |  | ST | 66 | 66 (10) | NR | NR | NR | 28 | 83 | 0 | 20 | 26 (5) | NR | NR | NR | NR |
|  |  | OMG3/ST | 68 | 68 (9) | NR | NR | NR | 25 | 68 | 0 | 18 | 25 (3) | NR | NR | NR | NR |
| Watanabe [61] | 2017 | ST | 119 | 68 (10) | NR | NR | NR | 35 | 65 | 0 | 21 | 23.9 (2.9) | 98.6 | 47.5 | 118.1 | 105 |
|  |  | OMG3/ST | 122 | 67 (10) | NR | NR | NR | 36 | 68 | 0 | 32 | 23.7 (3.1) | 107.1 | 49.8 | 125.1 | 111 |
| Koh [62] | 2018 | ST | 102 | 60.1 (9.1) | 93.1 | NR | NR | 37.3 | NR | NR | NR | 26.6 (3.8) | 99.3 | 46.1 | 128.4 | 136.5 |
|  |  | PCSK/ST | 97 | 61.2 (10.4) | 99 | NR | NR | 33 | NR | NR | NR | 26.3 (4.0) | 97 | 45.5 | 123.9 | 116 |
| Leiter [63] | 2017 | ST | 172 | 63.2 (8.6) | 31.4 | NR | NR | 100 | NR | NR | NR | 32.1 (5.1) | 109.7 | 47.7 | 142.7 | 153 |
|  |  | PCSK/ST | 345 | 62.3 (9.5) | 32.2 | NR | NR | 100 | NR | NR | NR | 32.3 (5.5) | 113.1 | 46.9 | 145.5 | 146 |
| Teramoto [64] | 2017 | ST | 103 | 60.5 (9.9) | 27.2 | NR | NR | 43.7 | NR | NR | NR | 25.4 (4.4) | 132.3 | 56 | 159.8 | 121 |
|  |  | CETP/ST | 204 | 60.9 (9.6) | 28.9 | NR | NR | 34.3 | NR | NR | NR | 25.2 (3.5) | 130.4 | 53.9 | 158.3 | 125 |
| Ballantyne [65] | 2017 | ST | 293 | 60.9 (8.9) | NR | NR | NR | 5.5 | 72 | NR | NR | 27.8 (4.2) | 91.3 | 43.6 | 125.2 | 153 |
|  |  | CETP/ST | 290 | 60.3 (9.1) | NR | NR | NR | 9 | 63.8 | NR | NR | 27.7 (4.3) | 88.4 | 43.5 | 121 | 141 |
| Ray [66] | 2018 | ST | 137 | 64.1 (8.8) | NR | NR | NR | 100 | 89.8 | NR | 16.8 | 33.2 (4.9) | 117.3 | 41.1 | 161.5 | 212 |
|  |  | PCSK/ST | 276 | 62.8 (9.3) | NR | NR | NR | 100 | 87.3 | NR | 13.8 | 32.7 (5.4) | 110.4 | 40.3 | 155.1 | 214.5 |
| Schwartz [67] | 2018 | ST | 9,462 | 58 (52-65) | NR | 19.5 | NR | 29.1 | 63.9 | NR | 24.1 | NR | 87 | 42 | 115 | 129 |
|  | 2018 | PCSK/ST | 9,462 | 58 (52-65) | NR | 18.9 | NR | 28.5 | 65.6 | NR | 24.1 | NR | 87 | 43 | 115 | 129 |
| Sang^a^ [68] | 2009 | ST | 56 | 68.8 (10.1) | NR | 36 | NR | 16 | 69 | NR | NR | NR | 103 | 51 | 135 | 158 |
|  |  | NIA/ST | 52 | 72.9 (6.8) | NR | 9.6 | NR | 54 | 63 | NR | NR | NR | 106 | 49 | 131 | 135 |

Red color indicated the intervention not included in network analysis because of no statin treatment

^a^The study not included in network meta-analysis

**Abbreviation:** CETP/ST=cholesteryl ester transfer protein inhibitor + statin; EZT/ST=ezetimibe + statin; FBT/ST=fibrate + statin; NIA/ST=niacin + statin; OMG3/ST=omega-3 fatty acids + statin; PCSK/ST=proprotein convertase subtilisin/kexin type 9 inhibitor + statin; NIA+EZT/ST= niacin + ezetimibe + statin; ST=statin monotherapy. NR= not reported

eTable 3.3 Description of interventions of included studies

| Author | Published year | **Treatment** | N | Requirement of statin use before random | **Intensity of statin^a^** | **Statin Users after randomization (%)** | **Any Discontinuation (%)** | **Discontinuation from AE (%)** |
| --- | --- | --- | --- | --- | --- | --- | --- | --- |
| Cannon [1] | 2015 | SMV (40 mg/d) | 9,077 | No | Moderate | 100 | 43.4 | 10.1 |
|  |  | EZT (10 mg/d) + SMV (40 mg/d) | 9,067 | No | Moderate | 100 | 42 | 10.6 |
| Kastelein [2] | 2008 | SMV (80 mg/d) | 363 | No | High | 100 | 15 | 9.4 |
|  |  | EZT (10 mg/d) + SMV (80 mg/d) | 357 | No | High | 100 | 23 | 8.1 |
| Taylor [3] | 2004 | Any ST | 80 | Stable dose | Moderate | 100 | 11.2 | 7.5 |
|  |  | NIA (1000 mg/d) + any ST | 87 | Stable dose | Moderate | 100 | 10.3 | 2.3 |
| Boden [4] | 2011 | SMV (40-80 mg/d) | 1,696 | Stable dose | Moderate/High | 100 | 20.1 | 9.9 |
|  |  | NIA (1500-2000 mg/d) + SMV (40-80 mg/d) | 1,718 | Stable dose | Moderate/High | 100 | 25.4 | 15.2 |
| Landray [5] | 2014 | SMV (40 mg/d) | 12,835 | Stable dose | Moderate | 100 | 16.6 | 7.9 |
|  |  | NIA/LRP (2000/40 mg/d) + SMV (40 mg/d) | 12,838 | Stable dose | Moderate | 100 | 25.4 | 16.4 |
| Ginsberg [6] | 2010 | SMV (20-40 mg/d) | 2,753 | Stable dose | Moderate | 100 | 2.8 | NR |
|  |  | FENO (160 mg/d) + SMV (20-40 mg/d) | 2,765 | Stable dose | Moderate | 100 | 3.2 | NR |
| Yokoyama [7] | 2007 | PRV (10-20 mg/d) or SMV (5-10 mg/d) | 9,319 | Stable dose | Low | 100 | 15.7 | 7.2 |
|  |  | EPA (1800) + PRV (10-20 mg/d) or SMV (5-10 mg/d) | 9,326 | Stable dose | Low | 100 | 21.1 | 11.7 |
| Nissen [8] | 2007 | Any ATV | 597 | Optimal lipid target | Moderate | 100 | 23.4 | 10.7 |
|  |  | TORC (60 mg/d) + any ATV | 591 | Optimal lipid target | Moderate | 100 | 22.8 | 11.2 |
| Barter [9] | 2007 | Any ATV | 7,534 | Optimal lipid target | Unclassified | 100 | 11 | 5.7 |
|  |  | TORC (60 mg/d) + any ATV | 7,533 | Optimal lipid target | Unclassified | 100 | 13.4 | 9.3 |
| Schwartz [10] | 2012 | Any ST | 7,933 | Optimal lipid target/ maximal tolerated | Unclassified | 98 | 19 | NR |
|  |  | DALC (600 mg/d) + any ST | 7,938 | Optimal lipid target/ maximal tolerated | Unclassified | 97 | 21 | NR |
| Robinson [11] | 2015 | Any ST | 788 | Maximal tolerated | Moderate/High | 99.9 | 24.5 | 6 |
|  |  | ALI (150 mg SC q 2 wk) + any ST | 1,553 | Maximal tolerated | Moderate/High | 99.9 | 28.2 | 7.2 |
| Sabatine [12] | 2015 | Any ST | 1,489 | Stable dose | Moderate/High | 69.7 | 4 | NR |
|  |  | EVO (140 mg SC q 2 wk or 420 mg SC q 1 mo) + any ST | 2,976 | Stable dose | Moderate/High | 70.9 | 3.4 | 2.3 |
| Taylor [13] | 2009 | EZT (10 mg/d) + any ST | 176 | Optimal lipid target | Moderate/High | 100 | 9 | 1.7 |
|  |  | NIA (2000 mg/d) + any ST | 187 | Optimal lipid target | Moderate/High | 100 | 15 | 9.1 |
| Guyton [14] | 2008 | NIA (2000 mg/d) | 272 | No | No | No | 39.0 | 25 |
|  |  | EZT (10 mg/d) + SMV (20 mg/d) | 272 | No | Moderate | 100 | 21.7 | 9.2 |
|  |  | NIA (2000 mg/d) +EZT (10 mg/d) + SMV (20 mg/d) | 676 | No | Moderate | 100 | 42 | 23.1 |
| Brunner [15] | 2013 | SMV (40 mg/d) | 48 | No | Moderate | 100 | NR | NR |
|  |  | NIA (1500 mg/d) + EZT (10 mg/d) + SMV (40 mg/d) | 47 | No | Moderate | 100 | NR | NR |
| Ballantyne [16] | 2008 | SMV (20 mg/d) | 121 | Optimal lipid target | Moderate | 100 | 19.8 | 5.3 |
|  |  | NIA (1000 mg/d) + SMV (20 mg/d) | 127 | Optimal lipid target | Moderate | 100 | 34.1 | 13.4 |
|  |  | NIA (2000 mg/d) + SMV (20 mg/d) | 66 | Optimal lipid target | Moderate | 100 | 34.1 | 13.4 |
| Wang [17] | 2016 | RSV (10 mg/d) | 51 | No | Moderate | 100 | 5.9 | 2 |
|  |  | EZT (10 mg/d) + RSV (10 mg/d) | 55 | No | Moderate | 100 | 9.1 | 3.6 |
| Tsujita [18] | 2015 | Any ATV | 124 | No | Low/Moderate | 100 | 17.8 | 2.4 |
|  |  | EZT (10 mg/d) + any ATV | 122 | No | Low/Moderate | 100 | 18 | 3.2 |
| Masuda [19] | 2015 | RSV (5 mg/d) | 25 | No | Moderate | 100 | 24 | 4.2 |
|  |  | EZT (10 mg/d) + RSV (5 mg/d) | 26 | No | Moderate | 100 | 19.2 | 7.6 |
| Luo [20] | 2014 | ATV (20 mg/d) | 44 | Stable dose | Moderate | 100 | NR | NR |
|  |  | EZT (10 mg/d) + ATV (20 mg/d) | 40 | Stable dose | Moderate | 100 | NR | NR |
| Rauch [21] | 2010 | Any ST | 1,911 | No | Unclassified | 93.8 | 14.6 | NR |
|  |  | EPA-DHA (460/380 mg/d) + any ST | 1,940 | No | Unclassified | 94.6 | 14.8 | NR |
| Farnier [22] | 2016 | RSV (40 mg/d) | 48 | Stable dose | High | 100 | 15.1 | 5.7 |
|  |  | EZT (10 mg/d) + RSV (20 mg/d) | 48 | Stable dose | High | 100 | 17 | 3.8 |
|  |  | ALI (75-150 mg SC q 2 wk) + RSV (20 mg/d) | 49 | Stable dose | High | 100 | 15.1 | 3.7 |
|  |  | RSV (20 mg/d) | 53 | Stable dose | High | 100 | 10.4 | 4.2 |
|  |  | EZT (10 mg/d) + RSV (10 mg/d) | 53 | Stable dose | Moderate | 100 | 29.2 | 12.5 |
|  |  | ALI (75-150 mg SC q 2 wk) + RSV (10 mg/d) | 54 | Stable dose | Moderate | 100 | 22.4 | 6.1 |
| Cannon [23] | 2015 | EZT (10 mg/d) + any ST | 241 | Maximal tolerated | Moderate/High | 100 | 14.5 | 5.4 |
|  |  | ALI (75-150 mg SC q 2 wk) + any ST | 479 | Maximal tolerated | Moderate/High | 100 | 13.1 | 7.5 |
| Blom [24] | 2014 | ATV (10 or 80 mg/d) | 302 | Optimal lipid target /maximal tolerated | Moderate/High | 88.8 | 9.3 | 1.3 |
|  |  | EVO (420 mg SC q 1 mo) + ATV (10 or 80 mg/d) | 599 | Optimal lipid target/ maximal tolerated | Moderate/High | 88.6 | 12.2 | 2.0 |
| West [25] | 2011 | SMV (40 mg/d) | 22 | No | Moderate | 100 | 27.3 | 4.5 |
|  |  | EZT (10 mg/d) + SMV (40 mg/d) | 22 | No | Moderate | 100 | 18.2 | 0 |
| Arimura [26] | 2012 | ATV (10 mg/d) | 22 | No | Moderate | 100 | 13.6 | 9.1 |
|  |  | EZT (10 mg/d) + ATV (10 mg/d) | 22 | No | Moderate | 100 | 13.6 | 0 |
| Bots [27] | 2007 | Any ATV | 375 | Optimal lipid target | Moderate | 100 | 8.3 | 4.3 |
|  |  | TORC (60 mg/d) + any ATV | 377 | Optimal lipid target | Moderate | 100 | 10.1 | 5.8 |
| Kastelein [28] | 2015 | Any ST | 102 | Stable dose | Moderate/High | 100 | 13.7 | 4.9 |
|  |  | ANAC (100 mg/d) + any ST | 204 | Stable dose | Moderate/High | 100 | 14.7 | 5.9 |
| Cannon [29] | 2010 | Any ST | 812 | Stable dose | Unclassified | 99.1 | 17.4 | 5.7 |
|  |  | ANAC (100 mg/d) + any ST | 811 | Stable dose | Unclassified | 99.5 | 32.1 | 5.7 |
| Davidson [30] | 2014 | Any ATV (10-40 mg/d) | 342 | Optimal lipid target | Moderate/High | 100 | 28.9 | 6.4 |
|  |  | FA (135 mg/d) + any ATV (10-40 mg/d) | 340 | Optimal lipid target | Moderate/High | 100 | 32.9 | 13.8 |
| Kastelein [31] | 2007 | Any ATV | 450 | Optimal lipid target/  maximal tolerated | High | 100 | 14.0 | 6.0 |
|  |  | TORC (60 mg/d) + any ATV | 454 | Optimal lipid target/  maximal tolerated | High | 100 | 13.9 | 8.8 |
| Derosa [32] | 2004 | FLV (80 mg/d) | 23 | No | Moderate | 100 | 8.7 | 8.7 |
|  |  | FENO (200 mg/d) + FLV (80) | 25 | No | Moderate | 100 | 12.0 | 8.0 |
| Durrington [33] | 2001 | SMV (10-40 mg/d) | 29 | Optimal lipid target/  maximal tolerated | Moderate | 100 | 10.3 | 6.9 |
|  |  | EPA-DHA (1760/1440 mg/d) + SMV (10-40 mg/d) | 30 | Optimal lipid target/  maximal tolerated | Moderate | 100 | 3.3 | 3.3 |
| Kastelein [34] | 2015 | Any ST | 82 | Maximal tolerated | Moderate/High | 100 | 12.2 | 1.2 |
|  | (ODYSSEY FH I) | ALI (75-150 mg SC q 2 wk) + any statin | 167 | Maximal tolerated | Moderate/High | 100 | 10.2 | 3.6 |
|  | 2015 | Any ST | 163 | Maximal tolerated | Moderate/High | 100 | 19.8 | 6.1 |
|  | (ODYSSEY FH I I) | ALI (75-150 mg SC q 2 wk) + any statin | 323 | Maximal tolerated | Moderate/High | 100 | 23.5 | 4.0 |
| Kereiakes [35] | 2015 | Any statin | 107 | Maximal tolerated | Moderate/High | 100 | 29.9 | 7.5 |
|  | 2015 | ALI (75-150 mg SC q 2 wk) + any statin | 209 | Maximal tolerated | Moderate/High | 99.5 | 24.4 | 6.2 |
| Nishio [36] | 2014 | Any RSV | 15 | No | Low/Moderate | 100 | 0 | 0 |
|  |  | EPA (1800 mg/d) + any RSV | 16 | No | Low/Moderate | 100 | 6.7 | 0 |
| Teramoto [37] | 2016 | Any ST | 72 | Stable dose | Unclassified | 100 | 8.3 | 5.6 |
|  |  | ALI (75-150 mg SC q 2 wk) + any statin | 144 | Stable dose | Unclassified | 100 | 7.6 | 4.9 |
| Stein [38] | 2010 | Any ATV | 46 | Optimal lipid target | Moderate/High | 100 | 23.9 | 8.7 |
|  | 2010 | DALC (900 mg/d) + any ATV | 89 | Optimal lipid target | Moderate/High | 100 | 22.5 | 12.4 |
| Bays [39] | 2015 | RSV (40 mg/d) | 45 | Stable dose | High | 100 | 13.3 | 2.2 |
|  |  | ATV (80 mg/d) | 47 | Stable dose | High | 100 | 17.0 | 6.4 |
|  |  | EZT (10) + ATV (40 mg/d) | 47 | Stable dose | High | 100 | 12.8 | 2.1 |
|  |  | ALI (75-150 mg SC q 2 wk) + ATV (40 mg/d) | 47 | Stable dose | High | 100 | 19.1 | 4.3 |
|  |  | ATV (40 mg/d) | 57 | Stable dose | High | 100 | 22.8 | 7.0 |
|  |  | EZT (10 mg/d) + ATV (20 mg/d) | 55 | Stable dose | Moderate | 100 | 27.3 | 5.5 |
|  |  | ALI (75-150 mg SC q 2 wk) + ATV (20 mg/d) | 57 | Stable dose | Moderate | 100 | 19.3 | 8.8 |
| Fayad [40] | 2011 | Any ST | 66 | Optimal lipid target/  maximal tolerated | Unclassified | 92 | 22.7 | 7.6 |
|  |  | DALC (600 mg/d) + any ST | 64 | Optimal lipid target/  maximal tolerated | Unclassified | 81 | 17.2 | 6.3 |
| Luscher [41] | 2012 | Any ST | 237 | Optimal lipid target/  maximal tolerated | Unclassified | 97 | 9.7 | 4.2 |
|  |  | DALC (600 mg/d) + any ST | 239 | Optimal lipid target/  maximal tolerated | Unclassified | 94 | 10.1 | 5.5 |
| Ballantyne [42] | 2008 | SMV (80 mg/d) | 123 | Stable dose | High | 100 | 22.0 | 4.1 |
|  |  | NIA (1000 mg/d) + SMV (40 mg/d) | 118 | Stable dose | Moderate | 100 | 24.6 | 11.0 |
|  |  | NIA (2000 mg/d) + SMV (40 mg/d) | 102 | Stable dose | Moderate | 100 | 21.6 | 11.8 |
| Nicholls [43] | 2016 | Any ST | 486 | Maximal tolerated | Moderate/High | 98.1 | 20.2 | 2.3 |
|  |  | EVO (420 mg SC q 1 mo) + any ST | 484 | Maximal tolerated | Moderate/High | 98.4 | 20.5 | 2.5 |
| Landray [44] | 2006 | SMV (20 mg/d) | 101 | No | Moderate | 100 | 7.8 | 1.0 |
|  |  | EZT (10 mg/d) + SMV (20 mg/d) | 102 | No | Moderate | 100 | 8.9 | 0.0 |
| Shaw [45] | 2009 | Any ST | 34 | Stable dose | Low/Moderate | 97.1 | 17.6 | 8.8 |
|  |  | EZT (10 mg/d) + any ST | 34 | Stable dose | Low/Moderate | 85.3 | 8.8 | 8.8 |
| Kouvelos [46] | 2013 | RSV (10 mg/d) | 136 | No | Moderate | 100 | NR | 1.5 |
|  |  | EZT (10 mg/d) + any ST | 126 | No | Moderate | 100 | NR | 1.6 |
| Ginsberg [47] | 2016 | Any ST | 35 | Maximal tolerated | Moderate/High | 100 | 25.7 | 5.7 |
|  |  | ALI (150 mg SC q 2wk) + any ST | 72 | Maximal tolerated | Moderate/High | 100 | 40.3 | 4.2 |
| Ballantyne [48] | 2017 | Any ST | 154 | Stable dose | Moderate/High | 0.5 | 9.1 | 5.8 |
|  |  | ANAC (100 mg/d) + any ST | 153 | Stable dose | Moderate/High | 0.5 | 8.5 | 2.6 |
|  |  | ANAC (25 mg/d) + any ST | 152 | Stable dose | Moderate/High | 0.5 | 10.5 | 3.3 |
| Kromhout [49] | 2010 | Any ST +/-ALA (2000 mg/d) | 2,433 | Stable dose | Unclassified | 86 | 8.1 | 1.8 |
|  |  | EPA-DHA (400 mg/d) + any ST +/-ALA (2000 mg/d) | 2,404 | Stable dose | Unclassified | 86 | 10.1 | 2.2 |
| Sabatine [50] | 2017 | Any ST | 13780 | Maximal tolerated | Moderate/High | 100 | 12.7 | 4.2 |
|  |  | EVO (140 mg SC q 2wk or 420 mg SC q 1 mo) + any ST | 13784 | Maximal tolerated | Moderate/High | 100 | 12.2 | 4.6 |
| Ridker [51] | 2017 | Any ST | 8409 | Maximal tolerated | Moderate/High | 91.4 | 2.8 | 0.1 |
|  | (SPIRE-1) | BOC (150 mg SC q 2 wk) + any ST | 8408 | Maximal tolerated | Moderate/High | 91.7 | 2.7 | 0.1 |
|  | 2017 | Any ST | 5309 | Maximal tolerated | Moderate/High | 73.5 | 4.8 | 0.3 |
|  | (SPIRE-2) | BOC (150 mg SC q 2 wk) + any ST | 5312 | Maximal tolerated | Moderate/High | 73.3 | 4.8 | 0.2 |
| Ridker [52] | 2017 | Any ST | 354 | Maximal tolerated | Moderate/High | 100 | NR | NR |
|  | (SPIRE-HR) | BOC (150 mg SC q 2 wk) + any ST | 357 | Maximal tolerated | Moderate/High | 100 | NR | NR |
|  | 2017 | Any ST | 1071 | Maximal tolerated | Moderate/High | 99.8 | NR | NR |
|  | (SPRIE-LDL) | BOC (150 mg SC q 2 wk) + any ST | 1068 | Maximal tolerated | Moderate/High | 99.5 | NR | NR |
|  | 2017 | Any ST | 185 | Maximal tolerated | Moderate/High | 100 | NR | NR |
|  | (SPIRE-FH) | BOC (150 mg SC q 2 wk) + any ST | 185 | Maximal tolerated | Moderate/High | 98.9 | NR | NR |
|  | 2017 | Any ST | 247 | Stable dose | Moderate/High | 96.6 | NR | NR |
|  | (SPIRE-LL) | BOC (150 mg SC q 2 wk) + any ST | 499 | Stable dose | Moderate/High | 100 | NR | NR |
|  | 2017 | Placebo | 73 | Statin intolerant | No | 0 | NR | NR |
|  | (SPIRE-SI) | ATV(40) | 37 | Statin intolerant | High | 100 | NR | NR |
|  |  | BOC (150 mg SC q 2 wk) + any ST | 74 | Statin intolerant | No | 0 | NR | NR |
|  | 2017 | Any ST | 49 | Stable dose | Moderate/High | 100 | NR | NR |
|  | (SPIRE-AI) | Any ST | 50 | Stable dose | Moderate/High | 100 | NR | NR |
|  |  | BOC(75 mg SC q 2 wk) (autoinjection) + any ST | 100 | Stable dose | Moderate/High | 100 | NR | NR |
|  |  | BOC(150 mg SC q 2 wk) (autoinjection) + any ST | 100 | Stable dose | Moderate/High | 100 | NR | NR |
| Luo [53] | 2017 | ATV (20 mg/d) | 74 | Stable dose | Moderate | 100 | NR | NR |
|  |  | EZT (10 mg/d) + ATV (20 mg/d) | 74 | Stable dose | Moderate | 100 | NR | NR |
| Liu [54] | 2017 | ATV (20 mg/d) | 114 | No | Moderate | 100 | NR | NR |
|  |  | EZT(10 mg/d) + ATV (10 mg/d) | 116 | No | Moderate | 100 | NR | NR |
| Nosaka [55] | 2017 | PTV (2 mg/d) | 121 | No | Moderate | 100 | NR | NR |
|  |  | EPA (1800 mg/d) + PTV (2 mg/d) | 120 | No | Moderate | 100 | NR | NR |
| Lincoff [56] | 2017 | Any ST | 6054 | Optimal lipid target/ maximal tolerated | Moderate/High | 2.3 | 18.8 | 6.7 |
|  |  | EVAC (130 mg/d) + any ST | 6038 | Optimal lipid target/ maximal tolerated | Moderate/High | 2.3 | 17.0 | 7.0 |
| Bowman [57] | 2017 | Any ATV | 15225 | Optimal lipid target | Moderate/High | 100 | 13.0 | 5.1 |
|  |  | ANAC (100 mg/d) + any ATV | 15224 | Optimal lipid target | Moderate/High | 100 | 13 | 5.1 |
| Hagiwara [58] | 2017 | PTV (2 mg/d) | 865 | No | Moderate | 100 | NR | 8.6 |
|  |  | EZT (10 mg/d) + PTV(2 mg/d) | 869 | No | Moderate | 100 | NR | 6.4 |
| Hibi [59] | 2018 | PTV (2 mg/d) | 63 | No | Moderate | 100 | 15.9 | 0.0 |
|  |  | EZT (10 mg/d) + PTV(2 mg/d) | 65 | No | Moderate | 100 | 23.1 | 0.0 |
| Miyoshi [60] | 2018 | PTV (2 mg/d) | 64 | Stable dose | Moderate | 100 | 14.1 | NR |
|  |  | PTV (4 mg/d) | 66 | Stable dose | Moderate | 100 | 30.3 | NR |
|  |  | EPA (1,800 mg/d) + PTV(2 mg/d) | 68 | Stable dose | Moderate | 100 | 17.6 | NR |
| Watanabe [61] | 2017 | PTV (4 mg/d) | 119 | No | Moderate | 100 | 8.4 | 2.6 |
|  |  | EPA (1,800 mg/d) + PTV(4 mg/d) | 122 | No | Moderate | 100 | 10.7 | 5.0 |
| Koh [62] | 2018 | Any ST | 102 | Maximal tolerated | Moderate/High | 100 | 4.9 | 1.0 |
|  |  | ALI (75-150 mg SC q 2 wk) + any ST | 97 | Maximal tolerated | Moderate/High | 100 | 10.3 | 2.1 |
| Leiter [63] | 2017 | Any ST | 172 | Maximal tolerated | Moderate/High | 75 | 7.6 | 2.3 |
|  |  | ALI (75-150 mg SC q 2 wk) + any ST | 345 | Maximal tolerated | Moderate/High | 74.8 | 9.3 | 4.9 |
| Teramoto [64] | 2017 | Any ST | 103 | Stable dose | Unclassified | 100 | 3.9 | 1.9 |
|  |  | ANAC (100 mg/d) + any ST | 204 | Stable dose | Unclassified | 100 | 6.9 | 5.9 |
| Ballantyne [65] | 2017 | Any ST | 293 | Stable dose | Unclassified | 100 | 11.9 | 3.8 |
|  | 2017 | ANAC (100 mg/d) + any ST | 290 | Stable dose | Unclassified | 100 | 5.5 | 2.4 |
| Ray [66] | 2018 | Any ST (± LLT) | 137 | Maximal tolerated | Moderate/High | 76 | 5.8 | 2.9 |
|  |  | ALI (75-150 mg SC q 2 wk) + any ST | 276 | Maximal tolerated | Moderate/High | 84 | 10.9 | 2.9 |
| Schwartz [67] | 2018 | Any ST | 9462 | Maximal tolerated | Moderate/High | 99 | 15.8 | NR |
|  |  | ALI (75-150 mg SC q 2 wk) + any ST | 9462 | Maximal tolerated | Moderate/High | 99.1 | 14.2 | NR |
| Sang^b^ [68] | 2009 | ATV (10 mg/d) | 56 | No | Moderate | 100 | NR | NR |
|  |  | NIA (1000 mg/d) + ATV (10 mg/d) | 52 | No | Moderate | 100 | NR | NR |

Red color indicated the intervention not included in network analysis because of no statin treatment

^a^Adapted from 2013 ACC/AHA,^57^ High intensity: atorvastatin (≥40 mg), rosuvastatin (≥20 mg), simvastatin (≥80 mg); Moderate intensity: atorvastatin (10-40 mg), rosuvastatin (5-20 mg), simvastatin (20-80 mg), pravastatin (≥40 mg), lovastatin (≥40 mg), fluvastatin (80 mg), pitavastatin (≥2 mg); Low intensity: atorvastatin (<10 mg), rosuvastatin (<5 mg), simvastatin (<20 mg), pravastatin (<40 mg), lovastatin (<40 mg)

^b^The study not included in network meta-analysis

Appendix 4 Risk of bias assessment

eFigure 4.1 Risk of bias graph

Rview authors' judgements (Low, Some concerns and High) about each risk of bias item presented as percentages across all included studies.

Low risk of bias Some concerns High risk of bias

eTable 4.1 Risk of bias summary: judgements about each bias item for each study

We followed the recommended approach for assessing risk of bias in studies included in Cochrane reviews. It addresses six specific domains (namely the randomization process, intended intervention, missing outcome data, measurement of outcome, reported results, and overall risk of bias). Each domain includes one or more specific entries in a ‘Risk of bias’ table. The tool involves assigning a judgment relating to the risk of bias for that entry. This is achieved by answering a pre-specified question about the adequacy of the study in relation to the entry, such that a judgment of low risk of bias, some concerns and high risk of bias.

|  | **1. The randomization process** | **2. Intended intervention** | **3. Missing outcome data** | **4. Measurement of outcome** | **5. Reported results** | **6. Overall risk of bias** |
| --- | --- | --- | --- | --- | --- | --- |
| Arimura, 2012 [26] | 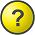 | 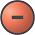 | 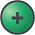 | 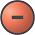 | 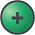 | 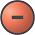 |
| Ballantyne, 2008 [16] | 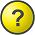 | 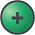 | 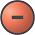 | 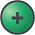 | 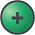 | 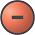 |
| Ballantyne, 2008 [42] | 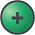 | 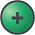 | 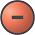 | 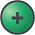 | 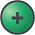 | 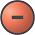 |
| Ballantyne, 2017 [48] | 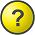 | 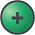 | 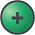 | 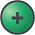 | 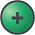 | 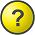 |
| Ballantyne, 2017 [65] | 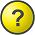 | 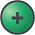 | 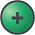 | 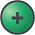 | 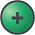 | 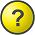 |
| Barter, 2007 [9] | 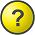 | 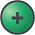 | 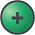 | 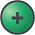 | 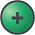 | 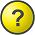 |
| Bays, 2015 [39] | 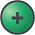 | 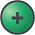 | 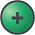 | 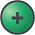 | 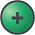 | 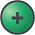 |
| Blom, 2014 [24] | 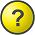 | 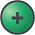 | 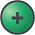 | 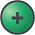 | 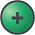 | 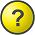 |
| Boden, 2011 [4] | 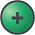 | 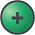 | 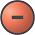 | 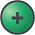 | 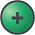 | 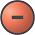 |
| Bots, 2007 [27] | 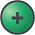 | 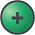 | 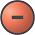 | 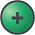 | 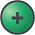 | 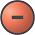 |
| Bowman, 2017 [57] | 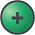 | 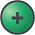 | 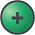 | 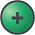 | 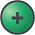 | 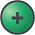 |
| Brunner, 2013 [15] | 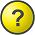 | 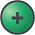 | 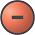 | 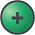 | 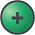 | 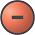 |
| Cannon, 2010 [29] | 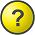 | 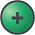 | 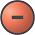 | 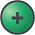 | 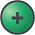 | 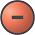 |
| Cannon, 2015 [1] | 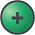 | 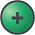 | 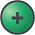 | 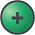 | 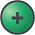 | 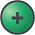 |
| Cannon, 2015 [23] | 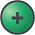 | 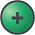 | 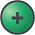 | 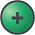 | 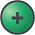 | 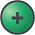 |
| Davidson, 2014 [30] | 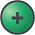 | 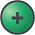 | 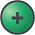 | 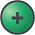 | 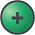 | 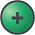 |
| Derosa, 2004 [32] | 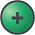 | 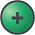 | 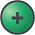 | 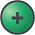 |  |  |
| Durrington, 2001 [33] |  |  |  |  |  |  |
| Farnier, 2016 [22] |  |  |  |  |  |  |
| Fayad, 2011 [40] |  |  |  |  |  |  |
| Gingberg, 2010 [6] |  |  |  |  |  |  |
| Gingberg, 2016 [47] |  |  |  |  |  |  |
| Guyton, 2008 [14] |  |  |  |  |  |  |
| Hagiwara, 2017 [58] |  |  |  |  |  |  |
| Hibi, 2018 [59] |  |  |  |  |  |  |
| Kastelein, 2007 [31] |  |  |  |  |  |  |
| Kastelein, 2008 [2] |  |  |  |  |  |  |
| Kastelein, 2015 [28] |  |  |  |  |  |  |
| Kastelein, 2015 [34] |  |  |  |  |  |  |
| Kereiakes, 2015 [35] |  |  |  |  |  |  |
| Koh, 2018 [62] |  |  |  |  |  |  |
| Kouvelos, 2013 [46] |  |  |  |  |  |  |
| Kromhout, 2010 [49] |  |  |  |  |  |  |
| Landray, 2006 [44] |  |  |  |  |  |  |
| Landray, 2014 [5] |  |  |  |  |  |  |
| Leiter, 2017 [63] |  |  |  |  |  |  |
| Lincoff, 2017 [56] |  |  |  |  |  |  |
| Liu, 2017 [54] |  |  |  |  |  |  |
| Luo, 2014 [20] |  |  |  |  |  |  |
| Luo, 2016 [53] |  |  |  |  |  |  |
| Luscher, 2012 [41] |  |  |  |  |  |  |
| Masuda, 2015 [19] |  |  |  |  |  |  |
| Miyoshi, 2018 [60] |  |  |  |  |  |  |
| Nicholls, 2016 [43] |  |  |  |  |  |  |
| Nishio, 2014 [36] |  |  |  |  |  |  |
| Nissen, 2007 [8] |  |  |  |  |  |  |
| Nosaka, 2017 [55] |  |  |  |  |  |  |
| Rauch, 2010 [21] |  |  |  |  |  |  |
| Ray, 2018 [66] |  |  |  |  |  |  |
| Ridker, 2017 [51] |  |  |  |  |  |  |
| Ridker, 2017 [52] |  |  |  |  |  |  |
| Robinson, 2015 [11] |  |  |  |  |  |  |
| Sabatine, 2015 [12] |  |  |  |  |  |  |
| Sabatine, 2017 [50] |  |  |  |  |  |  |
| Sang, 2009 [68] |  |  |  |  |  |  |
| Schwartz, 2012 [10] |  |  |  |  |  |  |
| Schwartz, 2018 [67] |  |  |  |  |  |  |
| Shaw, 2009 [45] |  |  |  |  |  |  |
| Stein, 2010 [38] |  |  |  |  |  |  |
| Taylor, 2004 [3] |  |  |  |  |  |  |
| Taylor, 2009 [13] |  |  |  |  |  |  |
| Teramoto, 2016 [37] |  |  |  |  |  |  |
| Teramoto, 2017 [64] |  |  |  |  |  |  |
| Tsujita, 2015 [18] |  |  |  |  |  |  |
| Wang, 2016 [17] |  |  |  |  |  |  |
| Watanabe, 2017 [61] |  |  |  |  |  |  |
| West, 2011 [25] |  |  |  |  |  |  |
| Yokoyama, 2007 [7] |  |  |  |  |  |  |
| Low risk Some concerns High risk | | | | | | |

Appendix 5 Results of meta-analyses of direct comparisons of treatment options

eTable 5.1 Pairwise meta-analyses risk ratio (and 95% CI) for all dichotomous outcomes

| First author, year | | No. of events/total | | Weight (%) | Risk ratio [95% CI] |
| --- | --- | --- | --- | --- | --- |
|  |  | Intervention | Comparator |  |  |
| **Primary outcome** |  |  |  |  |  |
| **Cardiovascular death** | | | | | |
| **CETP+ST vs ST (n = 11)** | | | | | |
| Barter, 2007 [9] | | 49/7533 | 35/7,534 | 5.36 | 1.40 (0.91, 2.16) |
| Bots, 2007 [27] | | 0/377 | 0/375 | 0.07 | 0.99 (0.02, 50.00) |
| Kastelein, 2007 [31] | | 0/454 | 1/450 | 0.1 | 0.33 (0.01, 8.09) |
| Cannon, 2010 29] | | 4/811 | 1/812 | 0.21 | 4.00 (0.45, 35.75) |
| Stein, 2010 [38] | | 0/89 | 0/46 | 0.07 | 0.52 (0.01, 25.90) |
| Fayad, 2011 [40] | | 0/64 | 2/66 | 0.11 | 0.21 (0.01, 4.21) |
| Kastelein, 2015 [28] | | 0/204 | 0/102 | 0.07 | 0.50 (0.01, 25.14) |
| Ballantyne, 2017 [65] | | 0/290 | 0/293 | 0.07 | 1.01 (0.02, 50.75) |
| Bowman, 2017 [57] | | 520/15224 | 564/15,225 | 73.3 | 0.92 (0.82, 1.04) |
| Lincoff, 2017 [56] | | 143/6038 | 166/6,054 | 20.6 | 0.86 (0.69, 1.08) |
| Teramoto, 2017 [64] | | 0/204 | 0/103 | 0.07 | 0.51 (0.01, 25.39) |
| Subtotal (I-squared = 0.0%, p = 0.703) | | 716/31,288 | 769/31,060 | 100.00 | 0.93 (0.84, 1.03) |
| **EZT+ST vs. ST (n = 11 )** | | | | | |
| Landray, 2006 [44] | | 2/102 | 0/101 | 0.15 | 4.95 (0.24, 101.87) |
| Kastelein, 2008 [2] | | 2/357 | 1/363 | 0.23 | 2.03 (0.19, 22.33) |
| Kouvelos, 2013 [46] | | 0/126 | 5/136 | 0.16 | 0.10 (0.01, 1.76) |
| Luo, 2014 [20] | | 0/40 | 0/44 | 0.09 | 1.10 (0.02, 54.06) |
| Bays, 2015 [39] | | 1/102 | 0/149 | 0.13 | 4.37 (0.18, 106.20) |
| Cannon, 2015 [1] | | 537/9,067 | 538/9,077 | 8.72 | 1.00 (0.89, 1.12) |
| Masuda, 2015 [19] | | 0/26 | 0/25 | 0.09 | 0.96 (0.02, 46.76) |
| Tsujita, 2015 [18] | | 0/122 | 0/124 | 0.09 | 1.02 (0.02, 50.81) |
| Farnier, 2016 [22] | | 1/101 | 0/101 | 0.13 | 3.00 (0.12, 72.78) |
| Luo, 2016 [53] | | 0/74 | 0/74 | 0.09 | 1.00 (0.02, 49.74) |
| Hibi, 2018 [59] | | 1/65 | 0/63 | 0.13 | 2.91 (0.12, 70.10) |
| Subtotal (I-squared = 0.0%, p = 0.847) | | 544/10,182 | 544/10,257 | 100 | 1.00 (0.90, 1.13) |
| **FBT+ST vs. ST (n = 2)** | | | | | |
| Ginsberg, 2010 [6] | | 99/2,765 | 114/2,753 | 99.25 | 0.86 (0.66, 1.13) |
| Davidson, 2014 [30] | | 0/340 | 2/342 | 0.75 | 0.20 (0.01, 4.17) |
| Subtotal (I-squared = 0.0%, p = 0.348) | | 99/3,105 | 116/3,095 | 100.00 | 0.86 (0.66, 1.11) |
| **NIA+ST vs. ST (n = 4)** | |  |  |  | 1.10 (0.97, 1.24) |
| Ballantyne, 2008 [16] | | 0/193 | 0/121 | 0.10 | 0.63 (0.01, 31.49) |
| Ballantyne, 2008 [42] | | 0/220 | 0/123 | 0.10 | 0.56 (0.01, 28.10) |
| Boden, 2011 [4] | | 45/1,718 | 38/1,696 | 8.67 | 1.17 (0.76, 1.79) |
| Landray, 2014 [5] | | 448/12,838 | 411/12,835 | 91.12 | 1.09 (0.96, 1.24) |
| Subtotal (I-squared = 0.0%, p = 0.963) | | 493/14,969 | 449/14,775 | 100.00 | 1.10 (0.97, 1.24) |
| **OMG3+ST vs. ST (n = 5)** | | | | | |
| Durrington, 2001[33] | | 0/30 | 1/29 | 0.88 | 0.32 (0.01, 7.61) |
| Kromhout, 2010 [49] | | 80/2,404 | 82/2,433 | 96.02 | 0.99 (0.73, 1.34) |
| Nishio, 2014 [36] | | 0/16 | 0/15 | 0.59 | 0.94 (0.02, 44.67) |
| Nosaka, 2017 [55] | | 1/120 | 5/121 | 1.94 | 0.20 (0.02, 1.70) |
| Miyoshi, 2018 [60] | | 0/68 | 0/130 | 0.58 | 1.90 (0.04, 94.65) |
| Subtotal (I-squared = 0.0%, p = 0.616) | | 81/2,638 | 88/2,728 | 100 | 0.95 (0.71, 1.28) |
| **PCSK+ST vs. ST (n = 11)** | | | | | |
| Blom, 2014 [24] | | 2/599 | 0/302 | 0.14 | 2.53 (0.12, 52.43) |
| Bays, 2015 [39] | | 0/104 | 0/149 | 0.09 | 1.43 (0.03, 71.43) |
| Sabatine, 2015 [12] | | 4/2,976 | 3/1,489 | 0.59 | 0.67 (0.15, 2.98) |
| Farnier, 2016 [22] | | 0/103 | 0/101 | 0.09 | 0.98 (0.02, 48.96) |
| Ginsberg, 2016 [47] | | 0/72 | 0/35 | 0.09 | 0.49 (0.01, 24.35) |
| Leiter, 2017 [63] | | 0/345 | 1/172 | 0.13 | 0.17 (0.01, 4.07) |
| Ridker, 2017 [51] | | 65/13,720 | 64/13,718 | 11.06 | 1.02 (0.72, 1.43) |
| Ridker, 2017 [52] | | 4/2,383 | 2/2,066 | 0.46 | 1.73 (0.32, 9.46) |
| Sabatine, 2017 [50] | | 251/13,784 | 240/13,780 | 42.64 | 1.05 (0.88, 1.25) |
| Koh, 2018 [62] | | 0/97 | 0/102 | 0.09 | 1.05 (0.02, 52.45) |
| Schwartz, 2018 [67] | | 240/9,462 | 271/9,462 | 44.65 | 0.89 (0.75, 1.05) |
| Subtotal (I-squared = 0.0%, p = 0.936) | | 566/43,645 | 581/41,376 | 100.00 | 0.97 (0.86, 1.08) |
| **NIA+ST vs. EZT+ST (n = 1)** | | | | | |
| Taylor, 2009 [13] | | 1/187 | 5/176 | 100.00 | 0.19 (0.02, 1.60) |
| Subtotal (I-squared = N/A, p = N/A) | | 1/187 | 5/176 | 100.00 | 0.19 (0.02, 1.60) |
| **NIA+EZT+ST vs. EZT+ST (n = 1)** | | | | | |
| Guyton, 2008 [14] | | 0/676 | 0/272 | 100.00 | 0.40 (0.01, 20.27) |
| Subtotal (I-squared = N/A, p = N/A) | | 0/676 | 0/272 | 100.00 | 0.40 (0.01, 20.27) |
| **PCSK+ST vs. EZT+ST (n=2)** | | | | | |
| Bays, 2015 [39] | | 0/104 | 1/102 | 50 | 0.33 (0.01, 7.93) |
| Farnier, 2016 [22] | | 0/103 | 1/101 | 50 | 0.33 (0.01, 7.93) |
| Subtotal (I-squared = 0.0%, p = 1.000) | | 0/207 | 2/203 | 100 | 0.33 (0.03, 3.12) |
| **All cause death** |  |  |  |  |  |
| **CETP+ST vs. ST (n = 13)** | | | | | |
| Barter, 2007 [9] | | 93/7,533 | 59/7,534 | 7.79 | 1.58 (1.14, 2.18) |
| Bots, 2007 [27] | | 1/377 | 1/375 | 0.12 | 0.99 (0.06, 15.84) |
| Nissen, 2007 [8] | | 8/591 | 6/597 | 0.82 | 1.35 (0.47, 3.86) |
| Cannon, 2010 [29] | | 11/811 | 8/812 | 1.11 | 1.38 (0.56, 3.40) |
| Stein, 2010 [38] | | 0/89 | 0/46 | 0.06 | 0.52 (0.01, 25.90) |
| Fayad, 2011 [40] | | 1/64 | 2/66 | 0.16 | 0.52 (0.05, 5.55) |
| Schwartz, 2012 [10] | | 226/7,938 | 229/7,933 | 20.10 | 0.99 (0.82, 1.18) |
| Kastelein, 2015 [28] | | 0/204 | 0/102 | 0.06 | 0.50 (0.01, 25.14) |
| Ballantyne, 2017 [48] | | 2/305 | 0/154 | 0.10 | 2.53 (0.12, 52.43) |
| Ballantyne, 2017 [65] | | 1/290 | 2/293 | 0.16 | 0.51 (0.05, 5.54) |
| Bowman, 2017 [57] | | 1,122/15,224 | 1,155/15,225 | 47.67 | 0.97 (0.90, 1.05) |
| Lincoff, 2017 [56] | | 231/6,038 | 276/6,054 | 21.78 | 0.84 (0.71, 1.00) |
| Teramoto, 2017 [64] | | 0/204 | 0/103 | 0.06 | 0.51 (0.01, 25.39) |
| Subtotal (I-squared = 11.6%, p = 0.329) | | 1,696/39,668 | 1,738/39,294 | 100.00 | 0.98 (0.89, 1.08) |
| **EZT+ST vs. ST (n = 9)** | | | | | |
| Landray, 2006 [44] | | 3/102 | 0/101 | 0.18 | 6.93 (0.36, 132.51) |
| Shaw, 2009 [45] | | 0/34 | 1/34 | 0.16 | 0.33 (0.01, 7.91) |
| West, 2011 [25] | | 4/22 | 3/22 | 0.83 | 1.33 (0.34, 5.28) |
| Bays, 2015 [39] | | 2/102 | 0/149 | 0.17 | 7.28 (0.35, 150.10) |
| Cannon, 2015 [1] | | 1,215/9,067 | 1,231/9,077 | 88.37 | 0.99 (0.92, 1.06) |
| Masuda, 2015 [19] | | 0/26 | 0/25 | 0.10 | 0.96 (0.02, 46.76) |
| Tsujita, 2015 [18] | | 0/122 | 0/124 | 0.10 | 1.02 (0.02, 50.81) |
| Hagiwara, 2017 [58] | | 42/869 | 60/865 | 9.91 | 0.70 (0.48, 1.02) |
| Hibi, 2018 [59] | | 2/65 | 0/63 | 0.17 | 4.85 (0.24, 99.04) |
| Subtotal (I-squared = 2.3%, p = 0.415) | | 1,268/10,409 | 1,295/10,460 | 100.00 | 0.96 (0.85, 1.09) |
| **FBT+ST vs. ST (n = 2)** | | | | | |
| Ginsberg, 2010 [6] | | 203/2,765 | 221/2,753 | 87.05 | 0.91 (0.76, 1.10) |
| Davidson, 2014 [30] | | 1/340 | 4/342 | 12.95 | 0.25 (0.03, 2.24) |
| Subtotal (I-squared = 24.8%, p = 0.249) | | 204/3,105 | 225/3,095 | 100.00 | 0.77 (0.33, 1.81) |
| **NIA+ST vs. ST (n = 5)** | | | | | |
| Taylor, 2004 [3] | | 1/87 | 2/80 | 0.15 | 0.46 (0.04, 4.97) |
| Ballantyne, 2008 [16] | | 0/193 | 1/121 | 0.08 | 0.21 (0.01, 5.10) |
| Ballantyne, 2008 [42] | | 0/220 | 0/123 | 0.06 | 0.56 (0.01, 28.10) |
| Boden, 2011 [4] | | 96/1,718 | 82/1,696 | 10.28 | 1.16 (0.87, 1.54) |
| Landray, 2014 [5] | | 798/12,838 | 732/12,835 | 89.44 | 1.09 (0.99, 1.20) |
| Subtotal (I-squared = 0.0%, p = 0.773) | | 895/15,056 | 817/14,855 | 100.00 | 1.09 (1.00, 1.20) |
| **OMG3+ST vs. ST (n = 7)** | | | | | |
| Durrington, 2001 [33] | | 0/30 | 1/29 | 0.15 | 0.32 (0.01, 7.61) |
| Yokoyama, 2007 [7] | | 286/9,326 | 256/9,319 | 47.98 | 1.12 (0.95, 1.32) |
| Kromhout, 2010 [49] | | 186/2,404 | 184/2,433 | 35.65 | 1.02 (0.84, 1.24) |
| Rauch, 2010 [21] | | 88/1,940 | 70/1,911 | 15.21 | 1.24 (0.91, 1.68) |
| Nishio, 2014 [36] | | 0/16 | 0/15 | 0.10 | 0.94 (0.02, 44.67) |
| Nosaka, 2017 [55] | | 2/120 | 9/121 | 0.65 | 0.22 (0.05, 1.02) |
| Watanabe, 2017 [61] | | 2/122 | 1/119 | 0.26 | 1.95 (0.18, 21.23) |
| Subtotal (I-squared = 24.8%, p = 0.249) | | 564/13,958 | 521/13,947 | 100.00 | 1.09 (0.96, 1.23) |
| **PCSK+ST vs. ST (n = 12)** | | | | | |
| Blom, 2014 [24] | | 2/599 | 0/302 | 0.17 | 2.53 (0.12, 52.43) |
| Bays, 2015 [39] | | 0/104 | 0/149 | 0.10 | 1.43 (0.03, 71.43) |
| Robinson, 2015 [11] | | 8/1,553 | 10/788 | 82.00 | 0.41 (0.16, 1.02) |
| Sabatine, 2015 [12] | | 4/2,976 | 6/1,489 | 0.99 | 0.33 (0.09, 1.18) |
| Ginsberg, 2016 [47] | | 0/72 | 0/35 | 0.11 | 0.49 (0.01, 24.35) |
| Nicholls, 2016 [43] | | 3/484 | 4/486 | 0.71 | 0.75 (0.17, 3.35) |
| Leiter, 2017 [63] | | 0/345 | 1/172 | 0.16 | 0.17 (0.01, 4.07) |
| Ridker, 2017 [51] | | 120/13,720 | 117/13,718 | 18.40 | 1.03 (0.80, 1.32) |
| Sabatine, 2017 [50] | | 444/13,784 | 426/13,780 | 40.18 | 1.04 (0.91, 1.19) |
| Koh, 2018 [62] | | 1/97 | 0/102 | 0.16 | 3.15 (0.13, 76.48) |
| Ray, 2018 [66] | | 1/276 | 0/137 | 0.16 | 1.49 (0.06, 36.45) |
| Schwartz, 2018 [67] | | 334/9,462 | 392/9,462 | 37.04 | 0.85 (0.74, 0.98) |
| Subtotal (I-squared = 13.8%, p = 0.309) | | 917/43,472 | 956/40,620 | 100.00 | 0.94 (0.82, 1.06) |
| **NIA+EZT+ST vs. ST (n = 1)** | | | | | |
| Brunner, 2013 [15] | | 4/47 | 4/48 | 100.00 | 1.02 (0.27, 3.85) |
| Subtotal (I-squared = N/A, p = N/A) | | 4/47 | 4/48 | 100.00 | 1.02 (0.27, 3.85) |
| **NIA+ST vs. EZT+ST (n = 1)** | | | | | |
| Taylor, 2009 [13] | | 7/176 | 1/187 | 100.00 | 0.13 (0.02, 1.08) |
| Subtotal (I-squared = N/A, p = N/A) | | 7/176 | 1/187 | 100.00 | 0.13 (0.02, 1.08) |
| **NIA+EZT+ST vs. EZT+ST (n = 1)** | | | | | |
| Guyton, 2008 [14] | | 0/676 | 0/272 | 100.00 | 0.40 (0.01, 20.27) |
| Subtotal (I-squared = N/A, p = N/A) | | 0/676 | 0/272 | 100.00 | 0.40 (0.01, 20.27) |
| **PCSK+ST vs. EZT+ST (n = 1)** | | | | | |
| Bays, 2015 [39] | | 0/104 | 2/102 | 100.00 | 0.20 (0.01, 4.04) |
| Subtotal (I-squared = 0.0%, p = 0.520) | | 0/104 | 2/102 | 100.00 | 0.20 (0.01, 4.04) |
| **Secondary outcome** | | | | | |
| **CHD death** |  |  |  |  |  |
| **CETP+ST vs. ST (n = 11)** | | | | | |
| Barter, 2007 [9] | | 40/7,533 | 33/7534 | 6.28 | 1.21 (0.77, 1.92) |
| Bots, 2007 [27] | | 0/377 | 0/375 | 0.09 | 0.99 (0.02, 50.00) |
| Nissen, 2007 [8] | | 1/591 | 1/597 | 0.17 | 1.01 (0.06, 16.11) |
| Stein, 2010 [38] | | 0/89 | 0/46 | 0.09 | 0.52 (0.01, 25.90) |
| Fayad, 2011 [40] | | 0/64 | 1/66 | 0.13 | 0.34 (0.01, 8.28) |
| Luscher, 2012 [41] | | 0/239 | 1/237 | 0.13 | 0.33 (0.01, 8.07) |
| Schwartz, 2012 [10] | | 118/7,938 | 125/7933 | 21.29 | 0.94 (0.73, 1.21) |
| Kastelein, 2015 [28] | | 0/204 | 0/102 | 0.09 | 0.50 (0.01, 25.14) |
| Ballantyne, 2017 [65] | | 0/290 | 0/293 | 0.09 | 1.01 (0.02, 50.75) |
| Bowman, 2017 [57] | | 388/15,224 | 420/15,225 | 71.56 | 0.92 (0.81, 1.06) |
| Teramoto, 2017 [64] | | 0/204 | 0/103 | 0.09 | 0.51 (0.01, 25.39) |
| Subtotal (I-squared = 0.0%, p = 0.993) | | 547/32,753 | 581/32,511 | 100.00 | 0.94 (0.84, 1.06) |
| **EZT+ST vs. ST (n = 11)** | | | | | |
| Arimura, 2012 [26] | | 1/22 | 0/22 | 0.16 | 3.00 (0.13, 69.87) |
| Kouvelos, 2013 [46] | | 0/126 | 3/136 | 0.18 | 0.15 (0.01, 2.95) |
| Bays, 2015 [39] | | 1/102 | 0/149 | 0.16 | 4.37 (0.18, 106.20) |
| Cannon, 2015 [1] | | 440/9,067 | 461/9,077 | 97.75 | 0.96 (0.84, 1.09) |
| Masuda, 2015 [19] | | 0/26 | 0/25 | 0.11 | 0.96 (0.02, 46.76) |
| Tsujita, 2015 [18] | | 0/122 | 0/124 | 0.10 | 1.02 (0.02, 50.81) |
| Farnier, 2016 [22] | | 0/101 | 0/101 | 0.10 | 1.00 (0.02, 49.91) |
| Luo, 2016 [53] | | 0/74 | 0/74 | 0.10 | 1.00 (0.02, 49.74) |
| Wang, 2016 [17] | | 0/55 | 0/51 | 0.10 | 0.93 (0.02, 45.95) |
| Liu, 2017 [54] | | 5/114 | 5/116 | 1.08 | 1.02 (0.30, 3.42) |
| Hibi, 2018 [59] | | 1/65 | 0/63 | 0.16 | 2.91 (0.12, 70.10) |
| Subtotal (I-squared = 0.0%, p = 0.973) | | 448/9,874 | 469/9,938 | 100.00 | 0.96 (0.85, 1.09) |
| **FBT+ST vs. ST (n = 1)** | | | | | |
| Davidson, 2014 [30] | | 0/340 | 1/342 | 100.00 | 0.34 (0.01, 8.20) |
| Subtotal (I-squared = N/A, p = N/A) | | 0/340 | 1/342 | 100.00 | 0.34 (0.01, 8.20) |
| **NIA+ST vs. ST (n = 4)** | | | | | |
| Ballantyne, 2008 [16] | | 0/193 | 0/121 | 0.15 | 0.63 (0.01, 31.49) |
| Ballantyne, 2008 [42] | | 0/220 | 0/123 | 0.15 | 0.56 (0.01, 28.10) |
| Boden, 2011 [4] | | 38/1,718 | 34/1,696 | 10.75 | 1.10 (0.70, 1.74) |
| Landray, 2014 [5] | | 302/12,838 | 291/12,835 | 88.96 | 1.04 (0.88, 1.22) |
| Subtotal (I-squared = 0.0%, p = 0.974) | | 340/14,969 | 325/14,775 | 100.00 | 1.04 (0.90, 1.21) |
| **OMG3+ST vs. ST (n = 6)** | | | | | |
| Durrington, 2001 [33] | | 0/30 | 1/29 | 0.74 | 0.32 (0.01, 7.61) |
| Yokoyama, 2007 [7] | | 29/9,326 | 31/9,319 | 9.00 | 0.93 (0.56, 1.55) |
| Kromhout, 2010 [49] | | 67/2,404 | 71/2,433 | 68.46 | 0.96 (0.69, 1.33) |
| Nishio, 2014 [36] | | 0/16 | 0/15 | 0.50 | 0.94 (0.02, 44.67) |
| Watanabe, 2017 [61] | | 2/122 | 0/119 | 0.81 | 4.88 (0.24, 100.55) |
| Miyoshi, 2018 [60] | | 0/68 | 0/130 | 0.49 | 1.90 (0.04, 94.65) |
| Subtotal (I-squared = 0.0%, p = 0.890) | | 98/11,966 | 103/12,045 | 100.00 | 0.96 (0.73, 1.26) |
| **PCSK+ST vs. ST (n = 10)** | | | | | |
| Blom, 2014 [24] | | 1/599 | 0/302 | 0.33 | 1.51 (0.06, 37.08) |
| Bays, 2015 [39] | | 0/104 | 0/149 | 0.22 | 1.43 (0.03, 71.43) |
| Kastelein, 2015 [34] | | 3/490 | 0/245 | 0.38 | 3.51 (0.18, 67.63) |
| Kereiakes, 2015 [35] | | 1/209 | 1/107 | 0.44 | 0.51 (0.03, 8.11) |
| Robinson, 2015 [11] | | 4/1,553 | 7/788 | 0.24 | 0.29 (0.09, 0.99) |
| Farnier, 2016 [22] | | 0/103 | 0/101 | 0.22 | 0.98 (0.02, 48.96) |
| Ginsberg, 2016 [47] | | 0/72 | 0/35 | 0.22 | 0.49 (0.01, 24.35) |
| Leiter, 2017 [63] | | 0/345 | 1/172 | 0.33 | 0.17 (0.01, 4.07) |
| Koh, 2018 [62] | | 0/97 | 0/102 | 0.22 | 1.05 (0.02, 52.45) |
| Schwartz, 2018 [67] | | 205/9,462 | 222/9,462 | 95.40 | 0.92 (0.77, 1.11) |
| Subtotal (I-squared = 0.0%, p = 0.774) | | 214/13,034 | 231/11,463 | 100.00 | 0.90 (0.75, 1.08) |
| **NIA+EZT+ST vs. EZT+ST (n = 1)** | | | | | |
| Guyton, 2008 [14] | | 0/676 | 0/272 | 100.00 | 0.40 (0.01, 20.27) |
| Subtotal (I-squared = N/A, p = N/A) | | 0/676 | 0/272 | 100.00 | 0.40 (0.01, 20.27) |
| **PCSK+ST vs. EZT+ST (n = 3)** | | | | | |
| Bays, 2015 [39] | | 0/104 | 1/102 | 23.10 | 0.33 (0.01, 7.93) |
| Cannon, 2015 [23] | | 2/479 | 2/241 | 61.54 | 0.50 (0.07, 3.55) |
| Farnier, 2016 [22] | | 0/103 | 0/101 | 5.36 | 0.98 (0.02, 48.96) |
| Subtotal (I-squared = 0.0%, p = 0.913) | | 2/686 | 3/444 | 100.00 | 0.50 (0.11, 2.34) |
| **Non-fatal myocardial infarction** | | | | | |
| **CETP+ST vs. ST (n = 7)** | | | | | |
| Barter, 2007 [9] | | 142/7,533 | 118/7,534 | 18.12 | 1.20 (0.94, 1.53) |
| Nissen, 2007 [8] | | 13/591 | 16/597 | 2.72 | 0.82 (0.40, 1.69) |
| Cannon, 2010 [29] | | 6/811 | 9/812 | 1.37 | 0.67 (0.24, 1.87) |
| Schwartz, 2012 [10] | | 414/7,938 | 407/7,933 | 36.20 | 1.02 (0.89, 1.16) |
| Ballantyne, 2017 [65] | | 0/290 | 1/293 | 0.14 | 0.34 (0.01, 8.23) |
| Bowman, 2017 [57] | | 546/15,224 | 628/15,225 | 41.35 | 0.87 (0.78, 0.97) |
| Teramoto, 2017 [64] | | 0/204 | 0/103 | 0.10 | 0.51 (0.01, 25.39) |
| Subtotal (I-squared = 26.4%, p = 0.227) | | 1,121/32,591 | 1,179/32,497 | 100.00 | 0.97 (0.86, 1.09) |
| **EZT+ST vs. ST (n = 8)** | | | | | |
| Kouvelos, 2013 [46] | | 0/126 | 1/136 | 0.07 | 0.36 (0.01, 8.75) |
| Luo, 2014 [20] | | 0/40 | 0/44 | 0.04 | 1.10 (0.02, 54.06) |
| Cannon, 2015 [1] | | 945/9,067 | 1,083/9,077 | 8.73 | 0.87 (0.80, 0.95) |
| Masuda, 2015 [19] | | 0/26 | 0/25 | 0.04 | 0.96 (0.02, 46.76) |
| Tsujita, 2015 [18] | | 1/122 | 1/124 | 0.09 | 1.02 (0.06, 16.07) |
| Farnier, 2016 [22] | | 1/101 | 0/101 | 0.07 | 3.00 (0.12, 72.78) |
| Luo, 2016 [53] | | 0/74 | 0/74 | 0.04 | 1.00 (0.02, 49.74) |
| Hagiwara, 2017 [58] | | 11/869 | 10/865 | 0.92 | 1.09 (0.47, 2.56) |
| Subtotal (I-squared = 0.0%, p = 0.992) | | 958/10,425 | 1,095/10,446 | 100.00 | 0.88 (0.81, 0.95) |
| **FBT+ST vs. ST (n = 3)** | | | | | |
| Derosa, 2004 [32] | | 0/25 | 0/23 | 0.26 | 0.92 (0.02, 44.72) |
| Ginsberg, 2010 [6] | | 173/2,765 | 186/2,753 | 98.96 | 0.93 (0.76, 1.13) |
| Davidson, 2014 [30] | | 3/340 | 1/342 | 0.78 | 3.02 (0.32, 28.87) |
| Subtotal (I-squared = 0.0%, p = 0.594) | | 176/3,130 | 187/3,118 | 100.00 | 0.93 (0.77, 1.14) |
| **NIA+ST vs. ST (n = 3)** | | | | | |
| Boden, 2011 [4] | | 92/1,718 | 80/1,696 | 27.34 | 1.14 (0.85, 1.52) |
| Landray, 2014 [5] | | 402/12,838 | 431/12,835 | 72.66 | 0.93 (0.82, 1.07) |
| Subtotal (I-squared = 30.6%, p = 0.230) | | 494/14,556 | 511/14,531 | 100.00 | 0.98 (0.83, 1.17) |
| **OMG3+ST vs. ST (n = 5)** | | | | | |
| Yokoyama, 2007 [7] | | 62/9,326 | 83/9,319 | 95.60 | 0.75 (0.54, 1.04) |
| Nishio, 2014 [36] | | 0/16 | 0/15 | 0.69 | 0.94 (0.02, 44.67) |
| Nosaka, 2017 [55] | | 1/120 | 0/121 | 1.01 | 3.02 (0.12, 73.52) |
| Watanabe, 2017 [61] | | 3/122 | 1/119 | 2.03 | 2.93 (0.31, 27.74) |
| Miyoshi, 2018 [60] | | 0/68 | 0/130 | 0.67 | 1.90 (0.04, 94.65) |
| Subtotal (I-squared = 0.0%, p = 0.681) | | 66/9,652 | 84/9,704 | 100.00 | 0.78 (0.57, 1.08) |
| **PCSK+ST vs. ST (n = 12)** | | | | | |
| Kastelein, 2015 [34] | | 1/490 | 2/245 | 0.28 | 0.25 (0.02, 2.74) |
| Kereiakes, 2015 [35] | | 1/209 | 1/107 | 0.21 | 0.51 (0.03, 8.11) |
| Robinson, 2015 [11] | | 14/1,553 | 18/788 | 3.12 | 0.39 (0.20, 0.79) |
| Ginsberg, 2016 [47] | | 0/103 | 0/101 | 0.11 | 0.98 (0.02, 48.96) |
| Farnier, 2016 [22] | | 4/72 | 0/35 | 0.19 | 4.44 (0.25, 80.21) |
| Nicholls, 2016 [43] | | 10/484 | 14/486 | 2.38 | 0.72 (0.32, 1.60) |
| Teramoto, 2016 [37] | | 1/144 | 1/72 | 0.21 | 0.50 (0.03, 7.88) |
| Ridker, 2017 [51] | | 192/13,720 | 210/13,718 | 22.07 | 0.91 (0.75, 1.11) |
| Ridker, 2017 [52] | | 24/2,383 | 16/2,066 | 3.73 | 1.30 (0.69, 2.44) |
| Sabatine, 2017 [50] | | 443/13,784 | 609/13,780 | 32.44 | 0.73 (0.64, 0.82) |
| Koh, 2018 [62] | | 0/97 | 1/102 | 0.16 | 0.35 (0.01, 8.50) |
| Schwartz, 2018 [67] | | 626/9,462 | 722/9,462 | 35.11 | 0.87 (0.78, 0.96) |
| Subtotal (I-squared = 28.4%, p = 0.167) | | 1,316/42,501 | 1,594/40,962 | 100.00 | 0.81 (0.72, 0.92) |
| **PCSK+ST vs. EZT+ST (n = 2)** | | | | | |
| Cannon, 2015 [23] | | 12/479 | 3/241 | 83.86 | 2.01 (0.57, 7.06) |
| Farnier, 2016 [22] | | 0/103 | 1/101 | 16.14 | 0.33 (0.01, 7.93) |
| Subtotal (I-squared = 7.4%, p = 0.299) | | 12/582 | 4/342 | 100.00 | 1.50 (0.40, 5.57) |
| **Any stroke** |  |  |  |  |  |
| **CETP+ST vs. ST (n = 10)** | | | | | |
| Barter, 2007 [9] | | 43/7,533 | 40/7,534 | 5.20 | 1.08 (0.70, 1.65) |
| Bots, 2007 [27] | | 1/377 | 0/375 | 0.09 | 2.98 (0.12, 73.02) |
| Nissen, 2007 [8] | | 1/454 | 1/450 | 0.12 | 0.99 (0.06, 15.80) |
| Kastelein, 2007 [31] | | 2/591 | 8/597 | 0.40 | 0.25 (0.05, 1.18) |
| Luscher, 2012 [41] | | 0/239 | 0/237 | 0.06 | 0.99 (0.02, 49.77) |
| Schwartz, 2012 [10] | | 91/7,938 | 73/7,933 | 10.21 | 1.25 (0.92, 1.69) |
| Ballantyne, 2017 [65] | | 0/290 | 2/293 | 0.10 | 0.20 (0.01, 4.19) |
| Bowman, 2017 [57] | | 547/15,224 | 559/15,225 | 71.55 | 0.98 (0.87, 1.10) |
| Lincoff, 2017 [56] | | 94/6,038 | 98/6,054 | 12.16 | 0.96 (0.73, 1.27) |
| Teramoto, 2017 [64] | | 1/204 | 0/103 | 0.09 | 1.52 (0.06, 37.04) |
| Subtotal (I-squared = 0.0%, p = 0.645) | | 780/38,888 | 781/38,801 | 100.00 | 1.00 (0.91, 1.10) |
| **EZT+ST vs. ST (n = 9)** | | | | | |
| West, 2011 [25] | | 3/22 | 2/22 | 0.72 | 1.50 (0.28, 8.12) |
| Arimura, 2012 [26] | | 0/22 | 1/22 | 0.21 | 0.33 (0.01, 7.76) |
| Cannon, 2015 [1] | | 296/9,067 | 345/9,077 | 8.65 | 0.86 (0.74, 1.00) |
| Masuda, 2015 [19] | | 0/26 | 0/25 | 0.14 | 0.96 (0.02, 46.76) |
| Farnier, 2016 [22] | | 0/101 | 1/101 | 0.20 | 0.33 (0.01, 8.09) |
| Luo, 2016 [53] | | 6/74 | 5/74 | 1.58 | 1.20 (0.38, 3.76) |
| Wang, 2016 [17] | | 0/55 | 0/51 | 0.14 | 0.93 (0.02, 45.95) |
| Hagiwara, 2017 [58] | | 17/869 | 18/865 | 4.79 | 0.94 (0.49, 1.81) |
| Liu, 2017 [54] | | 13/114 | 11/116 | 3.57 | 1.20 (0.56, 2.57) |
| Subtotal (I-squared = 0.0%, p = 0.975) | | 335/10,350 | 383/10,353 | 100.00 | 0.88 (0.76, 1.01) |
| **FBT+ST vs. ST (n = 2)** | | | | | |
| Ginsberg, 2010 [6] | | 51/2,765 | 48/2,753 | 92.73 | 1.06 (0.72, 1.56) |
| Davidson, 2014 [30] | | 0/340 | 2/342 | 7.27 | 0.20 (0.01, 4.17) |
| Subtotal (I-squared = 11.7%, p = 0.287) | | 51/3,105 | 50/3,095 | 100.00 | 0.94 (0.40, 2.18) |
| **NIA+ST vs. ST (n = 4)** | | | | | |
| Taylor, 2004 [3] | | 0/87 | 1/80 | 2.02 | 0.31 (0.01, 7.42) |
| Ballantyne, 2008 [16] | | 0/193 | 1/121 | 2.01 | 0.21 (0.01, 5.10) |
| Boden, 2011 [4] | | 27/1,718 | 15/1,696 | 30.17 | 1.78 (0.95, 3.33) |
| Landray, 2014 [5] | | 498/12,838 | 499/12,835 | 65.80 | 1.00 (0.88, 1.13) |
| Subtotal (I-squared = 35.0%, p = 0.202) | | 525/14,836 | 516/14,732 | 100.00 | 1.12 (0.71, 1.78) |
| **OMG3+ST vs. ST (n = 4)** | | | | | |
| Yokoyama, 2007 [7] | | 166/9,326 | 162/9,319 | 98.72 | 1.02 (0.83, 1.27) |
| Nosaka, 2017 [55] | | 0/120 | 4/121 | 0.54 | 0.11 (0.01, 2.06) |
| Watanabe, 2017 [61] | | 0/122 | 1/119 | 0.45 | 0.33 (0.01, 7.90) |
| Miyoshi, 2018 [60] | | 0/68 | 0/130 | 0.30 | 1.90 (0.04, 94.65) |
| Subtotal (I-squared = 0.0%, p = 0.425) | | 166/9,636 | 167/9,689 | 100.00 | 1.01 (0.81, 1.25) |
| **PCSK+ST vs. ST (n = 11)** | | | | | |
| Kastelein, 2015 [34] | | 1/490 | 0/245 | 0.17 | 1.50 (0.06, 36.76) |
| Kereiakes, 2015 [35] | | 2/209 | 0/107 | 0.19 | 2.57 (0.12, 53.09) |
| Robinson, 2015 [11] | | 9/1,553 | 2/788 | 0.75 | 2.28 (0.49, 10.54) |
| Sabatine, 2015 [12] | | 2/2,976 | 3/1,489 | 0.55 | 0.33 (0.06, 1.99) |
| Farnier, 2016 [22] | | 0/103 | 1/101 | 0.17 | 0.33 (0.01, 7.93) |
| Nicholls, 2016 [43] | | 2/484 | 3/486 | 0.55 | 0.67 (0.11, 3.99) |
| Ridker, 2017 [51] | | 45/13,720 | 75/13,718 | 12.83 | 0.60 (0.41, 0.87) |
| Ridker, 2017 [52] | | 6/2,383 | 9/2,066 | 1.64 | 0.58 (0.21, 1.62) |
| Sabatine, 2017 [50] | | 207/13,784 | 262/13,780 | 53.44 | 0.79 (0.66, 0.95) |
| Koh, 2018 [62] | | 0/97 | 1/102 | 0.17 | 0.35 (0.01, 8.50) |
| Schwartz, 2018 [67] | | 111/9,462 | 152/9,462 | 29.55 | 0.73 (0.57, 0.93) |
| Subtotal (I-squared = 0.0%, p = 0.804) | | 385/45,261 | 508/42,344 | 100.00 | 0.74 (0.65, 0.85) |
| **PCSK+ST vs. EZT+ST (n = 2)** | | | | | |
| Cannon, 2015 [23] | | 1/479 | 1/241 | 66.63 | 0.50 (0.03, 8.01) |
| Farnier, 2016 [22] | | 0/103 | 0/101 | 33.37 | 0.98 (0.02, 48.96) |
| Subtotal (I-squared = 0.0%, p = 0.785) | | 1/582 | 1/342 | 100.00 | 0.63 (0.07, 6.02) |
| **NIA+EZT+ST vs. EZT+ST (n = 1)** | | | | | |
| Guyton, 2008 [14] | | 0/676 | 1/272 | 100.00 | 0.13 (0.01, 3.29) |
| Subtotal (I-squared = N/A, p = N/A) | | 0/676 | 1/272 | 100.00 | 0.13 (0.01, 3.29) |
| **Coronary revascularization** | |  |  |  |  |
| **CETP+ST vs. ST (n = 6)** | | | | | |
| Barter, 2007 [9] | | 505/7,533 | 403/7,534 | 19.90 | 1.25 (1.10, 1.42) |
| Nissen, 2007 [8] | | 114/591 | 95/597 | 13.89 | 1.21 (0.95, 1.55) |
| Cannon, 2010 [29] | | 8/811 | 28/812 | 2.98 | 0.29 (0.13, 0.62) |
| Schwartz, 2012 [10] | | 674/7,938 | 672/7,933 | 21.04 | 1.00 (0.90, 1.11) |
| Bowman, 2017 [57] | | 1,081/15,224 | 1,201/15,225 | 21.98 | 0.90 (0.83, 0.97) |
| Lincoff, 2017 [56] | | 487/6,038 | 485/6,054 | 20.20 | 1.01 (0.89, 1.14) |
| Subtotal (I-squared = 84.0%, p = 0.000) | | 2,869/38,135 | 2,884/38,155 | 100.00 | 1.01 (0.88, 1.17) |
| **EZT+ST vs. ST (n = 10)** | | | | | |
| Arimura, 2012 [26] | | 2/22 | 3/22 | 0.11 | 0.67 (0.12, 3.61) |
| Bays, 2015 [39] | | 0/102 | 0/149 | 0.02 | 1.46 (0.03, 72.81) |
| Cannon, 2015 [1] | | 1,690/9,067 | 1,793/9,077 | 84.94 | 0.94 (0.89, 1.00) |
| Masuda, 2015 [19] | | 1/26 | 1/25 | 0.04 | 0.96 (0.06, 14.55) |
| Tsujita, 2015 [18] | | 22/122 | 23/124 | 1.09 | 0.97 (0.57, 1.65) |
| Farnier, 2016 [22] | | 0/101 | 0/101 | 0.02 | 1.00 (0.02, 49.91) |
| Luo, 2016 [53] | | 2/74 | 3/74 | 0.10 | 0.67 (0.11, 3.87) |
| Hagiwara, 2017 [58] | | 225/869 | 257/865 | 13.09 | 0.87 (0.75, 1.01) |
| Liu, 2017 [54] | | 10/114 | 6/116 | 0.32 | 1.70 (0.64, 4.51) |
| Hibi, 2018 [59] | | 6/65 | 7/63 | 0.28 | 0.83 (0.30, 2.34) |
| Subtotal (I-squared = 0.0%, p = 0.973) | | 1,952/10,497 | 2,086/10,553 | 100.00 | 0.93 (0.88, 0.99) |
| **NIA+ST vs. ST (n = 1)** | | | | | |
| Landray, 2014 [5] | | 591/12,838 | 664/12,835 | 100.00 | 0.89 (0.80, 0.99) |
| Subtotal (I-squared = N/A, p = N/A) | | 591/12,838 | 664/12,835 | 100.00 | 0.89 (0.80, 0.99) |
| OMG3+ST vs. ST **(n = 5)** | | 4 | 22,768 | 0.0% | 0.92 (0.84, 1.01) |
| Yokoyama, 2007 [7] | | 191/9,326 | 222/9,319 | 23.88 | 0.86 (0.71, 1.04) |
| Rauch, 2010 [21] | | 466/1,940 | 482/1,911 | 71.50 | 0.95 (0.85, 1.06) |
| Nishio, 2014 [36] | | 2/16 | 3/15 | 0.32 | 0.63 (0.12, 3.24) |
| Nosaka, 2017 [55] | | 9/120 | 15/121 | 1.41 | 0.61 (0.28, 1.33) |
| Watanabe, 2017 [61] | | 22/122 | 20/119 | 2.89 | 1.07 (0.62, 1.86) |
| Subtotal (I-squared = 0.0%, p = 0.655) | | 690/11,524 | 742/11,485 | 100.00 | 0.93 (0.84, 1.02) |
| **PCSK+ST vs. ST (n = 14)** | | | | | |
| Bays, 2015 [39] | | 1/104 | 0/149 | 0.04 | 4.29 (0.18, 104.18) |
| Kastelein, 2015 [34] | | 4/490 | 3/245 | 0.17 | 0.67 (0.15, 2.96) |
| Kereiakes, 2015 [35] | | 3/209 | 1/107 | 0.08 | 1.54 (0.16, 14.59) |
| Robinson, 2015 [11] | | 48/1,553 | 24/788 | 0.66 | 1.01 (0.63, 1.64) |
| Sabatine, 2015 [12] | | 15/2,976 | 17/1,489 | 0.81 | 0.44 (0.22, 0.88) |
| Farnier, 2016 [22] | | 0/103 | 0/101 | 0.03 | 0.98 (0.02, 48.96) |
| Ginsberg, 2016 [47] | | 5/72 | 0/35 | 0.05 | 5.42 (0.31, 95.43) |
| Nicholls, 2016 [43] | | 50/484 | 66/486 | 3.24 | 0.76 (0.54, 1.07) |
| Teramoto, 2016 [37] | | 2/144 | 1/72 | 0.07 | 1.00 (0.09, 10.85) |
| Ridker, 2017 [51] | | 67/13,720 | 75/13,718 | 3.58 | 0.89 (0.64, 1.24) |
| Ridker, 2017 [52] | | 40/2,383 | 38/2,066 | 2.00 | 0.91 (0.59, 1.42) |
| Sabatine, 2017 [50] | | 759/13,784 | 965/13,780 | 45.55 | 0.79 (0.72, 0.86) |
| Koh, 2018 [62] | | 3/97 | 4/102 | 0.18 | 0.79 (0.18, 3.43) |
| Schwartz, 2018 [67] | | 731/9,462 | 828/9,462 | 42.55 | 0.88 (0.80, 0.97) |
| Subtotal (I-squared = 0.0%, p = 0.654) | | 1,728/45,581 | 2,022/42,600 | 100.00 | 0.83 (0.78, 0.89) |
| **NIA+ST vs. EZT+ST (n = 1)** | | | | | |
| Taylor, 2009 [13] | | 0/187 | 3/176 | 100.00 | 0.13 (0.01, 2.59) |
| Subtotal (I-squared = N/A, p = N/A) | | 0/187 | 3/176 | 100.00 | 0.13 (0.01, 2.59) |
| **PCSK+ST vs. EZT+ST (n = 3)** | | | | | |
| Bays, 2015 [39] | | 1/104 | 0/102 | 9.70 | 2.94 (0.12, 71.41) |
| Cannon, 2015 [23] | | 16/479 | 4/241 | 83.85 | 2.01 (0.68, 5.95) |
| Farnier, 2016 [22] | | 0/103 | 0/101 | 6.45 | 0.98 (0.02, 48.96) |
| Subtotal (I-squared = 0.0%, p = 0.912) | | 17/686 | 4/444 | 100.00 | 1.99 (0.74, 5.38) |
| **Discontinuation due to any cause** | |  |  |  |  |
| **CETP+ST vs. ST (n = 14)** | | | | | |
| Barter, 2007 [9] | | 1,008/7,533 | 831/7,534 | 13.45 | 1.21 (1.11, 1.32) |
| Bots, 2007 [27] | | 38/377 | 31/375 | 5.50 | 1.22 (0.78, 1.92) |
| Kastelein, 2007 [31] | | 63/454 | 63/450 | 7.84 | 0.99 (0.72, 1.37) |
| Nissen, 2007 [8] | | 135/591 | 140/597 | 10.67 | 0.97 (0.79, 1.20) |
| Cannon, 2010 [29] | | 260/811 | 141/812 | 11.36 | 1.85 (1.54, 2.21) |
| Stein, 2010 [38] | | 20/89 | 11/46 | 3.38 | 0.94 (0.49, 1.79) |
| Fayad, 2011 [40] | | 11/64 | 15/66 | 2.98 | 0.76 (0.38, 1.52) |
| Luscher, 2012 [41] | | 24/239 | 23/237 | 4.33 | 1.03 (0.60, 1.78) |
| Kastelein, 2015 [28] | | 30/204 | 14/102 | 3.86 | 1.07 (0.60, 1.93) |
| Ballantyne, 2017 [48] | | 29/305 | 14/154 | 3.68 | 1.05 (0.57, 1.92) |
| Ballantyne, 2017 [65] | | 16/290 | 35/293 | 4.06 | 0.46 (0.26, 0.82) |
| Bowman, 2017 [57] | | 2,055/15,224 | 2,055/15,225 | 13.88 | 1.00 (0.94, 1.06) |
| Lincoff, 2017 [56] | | 1,025/6,038 | 1,139/6,054 | 13.61 | 0.90 (0.84, 0.97) |
| Teramoto, 2017 [64] | | 14/204 | 4/103 | 1.40 | 1.77 (0.60, 5.23) |
| Subtotal (I-squared = 83.1%, p = 0.000) | | 4,728/32,423 | 4,516/32,048 | 100.00 | 1.06 (0.93, 1.22) |
| **EZT+ST vs. ST (n = 12)** | | | | | |
| Landray, 2006 [44] | | 9/102 | 8/101 | 3.71 | 1.11 (0.45, 2.77) |
| Kastelein, 2008[2] | | 86/357 | 57/363 | 18.53 | 1.53 (1.14, 2.07) |
| Shaw, 2009 [45] | | 3/34 | 6/34 | 1.92 | 0.50 (0.14, 1.84) |
| West, 2011 [25] | | 4/22 | 6/22 | 2.55 | 0.67 (0.22, 2.04) |
| Arimura, 2012 [26] | | 3/22 | 3/22 | 1.49 | 1.00 (0.23, 4.42) |
| Bays, 2015 [39] | | 21/102 | 27/149 | 9.63 | 1.14 (0.68, 1.90) |
| Cannon, 2015 [1] | | 3,809/9,067 | 3,941/9,077 | 35.88 | 0.97 (0.94, 1.00) |
| Masuda, 2015 [19] | | 5/26 | 6/25 | 2.86 | 0.80 (0.28, 2.29) |
| Tsujita, 2015 [18] | | 22/122 | 22/124 | 9.00 | 1.02 (0.59, 1.74) |
| Farnier, 2016 [22] | | 23/101 | 13/101 | 7.14 | 1.77 (0.95, 3.29) |
| Wang, 2016 [17] | | 5/55 | 3/51 | 1.72 | 1.55 (0.39, 6.14) |
| Hibi, 2018 [59] | | 15/65 | 10/63 | 5.58 | 1.45 (0.71, 2.99) |
| Subtotal (I-squared = 31.5%, p = 0.139) | | 4,005/10,075 | 4,102/10,132 | 100.00 | 1.13 (0.94, 1.36) |
| **FBT+ST vs. ST (n = 3)** | | | | | |
| Derosa, 2004 [32] | | 3/25 | 2/23 | 1.11 | 1.38 (0.25, 7.53) |
| Ginsberg, 2010 [6] | | 88/2,765 | 78/2,753 | 35.55 | 1.12 (0.83, 1.52) |
| Davidson, 2014 [30] | | 112/340 | 99/342 | 63.34 | 1.14 (0.91, 1.42) |
| Subtotal (I-squared = 0.0%, p = 0.972) | | 203/3,130 | 179/3,118 | 100.00 | 1.14 (0.95, 1.36) |
| **NIA+ST vs. ST (n = 7)** | | | | | |
| Taylor, 2004 [3] | | 9/87 | 9/80 | 2.98 | 0.92 (0.38, 2.20) |
| Ballantyne, 2008 [16] | | 62/193 | 24/121 | 10.75 | 1.62 (1.07, 2.45) |
| Ballantyne, 2008 [42] | | 51/220 | 27/123 | 10.82 | 1.06 (0.70, 1.59) |
| Boden, 2011 [4] | | 436/1,718 | 341/1,696 | 33.91 | 1.26 (1.11, 1.43) |
| Landray, 2014 [5] | | 3,256/12,838 | 2,136/12,835 | 41.54 | 1.52 (1.45, 1.60) |
| Subtotal (I-squared = 65.4%, p = 0.021) | | 3,814/15,056 | 2,537/14,855 | 100.00 | **1.36 (1.17, 1.59)** |
| **OMG3+ST vs. ST (n = 7)** | | | | | |
| Durrington, 2001 [33] | | 1/30 | 3/29 | 0.55 | 0.32 (0.04, 2.92) |
| Yokoyama, 2007 [7] | | 1,970/9,326 | 1,464/9,319 | 35.15 | 1.34 (1.26, 1.43) |
| Kromhout, 2010 [49] | | 244/2,404 | 196/2,433 | 25.74 | 1.26 (1.05, 1.51) |
| Rauch, 2010 [21] | | 287/1,940 | 279/1,911 | 28.12 | 1.01 (0.87, 1.18) |
| Nishio, 2014 [36] | | 1/16 | 0/15 | 0.28 | 2.82 (0.12, 64.39) |
| Watanabe, 2017 [61] | | 13/122 | 10/119 | 3.96 | 1.27 (0.58, 2.78) |
| Miyoshi, 2018 [60] | | 12/68 | 29/130 | 6.20 | 0.79 (0.43, 1.45) |
| Subtotal (I-squared = 61.9%, p = 0.015) | | 2,528/13,906 | 1,981/13,956 | 100.00 | 1.17 (0.99, 1.38) |
| **PCSK+ST vs. ST (n = 16)** | | | | | |
| Blom, 2014 [24] | | 73/599 | 28/302 | 2.99 | 1.31 (0.87, 1.99) |
| Bays, 2015 [39] | | 20/104 | 27/149 | 1.97 | 1.06 (0.63, 1.79) |
| Kastelein, 2015 [34] | | 93/490 | 43/245 | 4.44 | 1.08 (0.78, 1.50) |
| Kereiakes, 2015 [35] | | 51/209 | 32/107 | 3.53 | 0.82 (0.56, 1.19) |
| Robinson, 2015 [11] | | 437/1,553 | 193/788 | 12.88 | 1.15 (0.99, 1.33) |
| Sabatine, 2015 [12] | | 101/2,976 | 59/1,489 | 4.73 | 0.86 (0.63, 1.17) |
| Farnier, 2016 [22] | | 24/103 | 13/101 | 1.44 | 1.81 (0.98, 3.35) |
| Ginsberg, 2016 [47] | | 29/72 | 9/35 | 1.39 | 1.57 (0.83, 2.94) |
| Nicholls, 2016 [43] | | 99/484 | 98/486 | 6.77 | 1.01 (0.79, 1.30) |
| Teramoto, 2016 [37] | | 11/144 | 6/72 | 0.62 | 0.92 (0.35, 2.38) |
| Leiter, 2017 [63] | | 32/345 | 13/172 | 1.43 | 1.23 (0.66, 2.28) |
| Ridker, 2017 [51] | | 481/13,720 | 491/13,718 | 14.89 | 0.98 (0.87, 1.11) |
| Sabatine, 2017 [50] | | 1,682/13,784 | 1,746/13,780 | 20.96 | 0.96 (0.90, 1.03) |
| Koh, 2018 [62] | | 10/97 | 5/102 | 0.53 | 2.10 (0.75, 5.93) |
| Ray, 2018 [66] | | 30/276 | 8/137 | 0.99 | 1.86 (0.88, 3.95) |
| Schwartz, 2018 [67] | | 1,343/9,462 | 1,496/9,462 | 20.45 | 0.90 (0.84, 0.96) |
| Subtotal (I-squared = 42.4%, p = 0.037) | | 4,516/44,418 | 4,267/41,145 | 100.00 | 1.01 (0.94, 1.09) |
| **NIA+ST vs. EZT+ST (n = 1)** | | 1 | 363 | NA | 1.65 (0.92, 2.94) |
| Taylor, 2009 [13] | | 28/187 | 16/176 | 100.00 | 1.65 (0.92, 2.94) |
| Subtotal (I-squared = N/A, p = N/A) | | 28/187 | 16/176 | 100.00 | 1.65 (0.92, 2.94) |
| **NIA+EZT+ST vs. EZT+ST (n = 1)** | | | | | |
| Guyton, 2008 [14] | | 285/676 | 59/272 | 100.00 | 1.94 (1.53, 2.48) |
| Subtotal (I-squared = N/A, p = N/A) | | 285/676 | 59/272 | 100.00 | **1.94 (1.53, 2.48)** |
| **PCSK+ST vs. EZT+ST (n = 4)** | | | | | |
| Bays, 2015 [39] | | 20/104 | 21/102 | 23.59 | 0.93 (0.54, 1.62) |
| Cannon, 2015 [23] | | 63479 | 35/241 | 48.27 | 0.91 (0.62, 1.33) |
| Farnier, 2016 [22] | | 24/103 | 23/101 | 28.14 | 1.02 (0.62, 1.69) |
| Subtotal (I-squared = 0.0%, p = 0.930) | | 107/686 | 79/444 | 100.00 | 0.94 (0.72, 1.23) |
| **Discontinuation due to adverse events** | | | | | |
| **CETP+ST vs. ST (n = 14)** | | | | | |
| Barter, 2007 [9] | | 698/7,533 | 432/7,534 | 15.61 | 1.62 (1.44, 1.81) |
| Bots, 2007 [27] | | 22/377 | 16/375 | 5.70 | 1.37 (0.73, 2.56) |
| Kastelein, 2007 [31] | | 40/454 | 27/450 | 8.01 | 1.47 (0.92, 2.35) |
| Nissen, 2007 [8] | | 66/591 | 64/597 | 10.99 | 1.04 (0.75, 1.44) |
| Cannon, 2010 [29] | | 46/811 | 46/812 | 9.41 | 1.00 (0.67, 1.49) |
| Stein, 2010 [38] | | 11/89 | 4/46 | 2.46 | 1.42 (0.48, 4.22) |
| Fayad, 2011 [40] | | 4/64 | 5/66 | 1.88 | 0.82 (0.23, 2.93) |
| Luscher, 2012 [41] | | 13/239 | 10/237 | 4.01 | 1.29 (0.58, 2.88) |
| Kastelein, 2015 [28] | | 12/204 | 5/102 | 2.76 | 1.20 (0.43, 3.31) |
| Ballantyne, 2017 [48] | | 9/305 | 9/154 | 3.34 | 0.50 (0.20, 1.25) |
| Ballantyne, 2017 [65] | | 7/290 | 11/293 | 3.17 | 0.64 (0.25, 1.64) |
| Bowman, 2017 [57] | | 774/15,224 | 779/15,225 | 15.90 | 0.99 (0.90, 1.09) |
| Lincoff, 2017 [56] | | 424/6,038 | 405/6,054 | 15.33 | 1.05 (0.92, 1.20) |
| Teramoto, 2017 [64] | | 12/204 | 2/103 | 1.43 | 3.03 (0.69, 13.28) |
| Subtotal (I-squared = 75.3%, p = 0.000) | | 2,138/32,423 | 1,815/32,048 | 100.00 | 1.14 (0.95, 1.38) |
| **EZT+ST vs. ST (n = 14)** | | | | | |
| Landray, 2006 [44] | | 0/102 | 1/101 | 0.07 | 0.33 (0.01, 8.01) |
| Kastelein, 2008 [2] | | 29/357 | 34/363 | 3.07 | 0.87 (0.54, 1.39) |
| Shaw, 2009 [45] | | 3/34 | 3/34 | 0.30 | 1.00 (0.22, 4.61) |
| West, 2011 [25] | | 0/22 | 1/22 | 0.07 | 0.33 (0.01, 7.76) |
| Arimura, 2012 [26] | | 0/22 | 2/22 | 0.08 | 0.20 (0.01, 3.94) |
| Kouvelos, 2013 [46] | | 2/126 | 2/136 | 0.18 | 1.08 (0.15, 7.55) |
| Bays, 2015 [39] | | 4/102 | 8/149 | 0.50 | 0.73 (0.23, 2.36) |
| Cannon, 2015 [1] | | 903/9,067 | 877/9,077 | 88.47 | 1.03 (0.94, 1.13) |
| Masuda, 2015 [19] | | 2/26 | 1/25 | 0.13 | 1.92 (0.19, 19.90) |
| Tsujita, 2015 [18] | | 4/122 | 3/124 | 0.32 | 1.36 (0.31, 5.93) |
| Farnier, 2016 [22] | | 8/101 | 5/101 | 0.59 | 1.60 (0.54, 4.72) |
| Wang, 2016 [17] | | 2/55 | 1/51 | 0.12 | 1.85 (0.17, 19.84) |
| Hagiwara, 2017 [58] | | 55/869 | 73/865 | 6.06 | 0.75 (0.54, 1.05) |
| Hibi, 2018 [59] | | 0/65 | 0/63 | 0.05 | 0.97 (0.02, 48.14) |
| Subtotal (I-squared = 0.0%, p = 0.883) | | 1,012/11,070 | 1,011/11,133 | 100.00 | 1.01 (0.93, 1.09) |
| **FBT+ST vs. ST (n = 2)** | | | | | |
| Derosa, 2004 [32] | | 2/25 | 2/23 | 6.23 | 0.92 (0.14, 6.01) |
| Davidson, 2014 [30] | | 47/340 | 22/342 | 93.77 | 2.15 (1.33, 3.49) |
| Subtotal (I-squared = 0.0%, p = 0.391) | | 49/365 | 24/365 | 100.00 | **2.04 (1.28, 3.26)** |
| **NIA+ST vs. ST (n = 5)** | | | | | |
| Taylor, 2004 [3] | | 2/87 | 6/80 | 3.27 | 0.31 (0.06, 1.48) |
| Ballantyne, 2008 [16] | | 25/193 | 6/121 | 9.24 | 2.61 (1.10, 6.18) |
| Ballantyne, 2008 [42] | | 25/220 | 5/123 | 8.12 | 2.80 (1.10, 7.12) |
| Boden, 2011 [4] | | 261/1,718 | 169/1,696 | 37.17 | 1.52 (1.27, 1.83) |
| Landray, 2014 [5] | | 2,107/12,838 | 1,020/12,835 | 42.20 | 2.07 (1.92, 2.22) |
| Subtotal (I-squared = 74.5%, p = 0.004) | | 2,420/15,056 | 1,206/14,855 | 100.00 | **1.82 (1.35, 2.44)** |
| **OMG3+ST vs. ST (n = 5)** | | | | | |
| Durrington, 2001 [33] | | 1/30 | 2/29 | 0.14 | 0.48 (0.05, 5.05) |
| Yokoyama, 2007 [7] | | 1,087/9,326 | 673/9,319 | 94.25 | 1.61 (1.47, 1.77) |
| Kromhout, 2010 [49] | | 53/2,404 | 45/2,433 | 5.13 | 1.19 (0.80, 1.77) |
| Nishio, 2014 [36] | | 0/16 | 0/15 | 0.05 | 0.94 (0.02, 44.67) |
| Watanabe, 2017 [61] | | 6/122 | 3/119 | 0.43 | 1.95 (0.50, 7.62) |
| Subtotal (I-squared = 0.0%, p = 0.507) | | 1,147/11,898 | 723/11,915 | 100.00 | **1.59 (1.45, 1.73)** |
| **PCSK+ST vs. ST (n = 15)** | | | | | |
| Blom, 2014 [24] | | 12/599 | 4/302 | 0.67 | 1.51 (0.49, 4.65) |
| Bays, 2015 [39] | | 7/104 | 8/149 | 0.87 | 1.25 (0.47, 3.35) |
| Kastelein, 2015 [34] | | 19/490 | 11/245 | 1.6 | 0.86 (0.42, 1.79) |
| Kereiakes, 2015 [35] | | 13/209 | 8/107 | 1.17 | 0.83 (0.36, 1.95) |
| Robinson, 2015 [11] | | 113/1,553 | 47/788 | 7.77 | 1.22 (0.88, 1.70) |
| Farnier, 2016 [22] | | 5/103 | 5/101 | 0.58 | 0.98 (0.29, 3.28) |
| Ginsberg, 2016 [47] | | 3/72 | 2/35 | 0.28 | 0.73 (0.13, 4.17) |
| Nicholls, 2016 [43] | | 12/484 | 11/486 | 1.29 | 1.10 (0.49, 2.46) |
| Teramoto, 2016 [37] | | 7/144 | 4/72 | 0.59 | 0.88 (0.26, 2.89) |
| Leiter, 2017 [63] | | 17/345 | 4/172 | 0.73 | 2.12 (0.72, 6.20) |
| Ridker, 2017 [51] | | 19/13,720 | 23/13,718 | 2.28 | 0.83 (0.45, 1.52) |
| Ridker, 2017 [52] | | 115/2,383 | 98/2,066 | 12.18 | 1.02 (0.78, 1.32) |
| Sabatine, 2017 [50] | | 628/13,784 | 581/13,780 | 69.2 | 1.08 (0.97, 1.21) |
| Koh, 2018 [62] | | 2/97 | 1/102 | 0.15 | 2.10 (0.19, 22.82) |
| Ray, 2018 [66] | | 10/276 | 4/137 | 0.65 | 1.24 (0.40, 3.89) |
| Subtotal (I-squared = 0.0%, p = 0.988) | | 982/34,363 | 811/32,260 | 100 | 1.08 (0.98, 1.18) |
| **NIA+ST vs. EZT+ST (n = 1)** | | | | | |
| Taylor, 2009 [^13]^ | | 17/187 | 3/176 | 100.00 | 5.33 (1.59, 17.89) |
| Subtotal (I-squared = N/A, p = N/A) | | 17/187 | 3/176 | 100.00 | **5.33 (1.59, 17.89)** |
| **NIA+EZT+ST vs. EZT+ST (n = 1)** | | | | | |
| Guyton, 2008 [^14]^ | | 156/676 | 25/272 | 100.00 | 2.51 (1.69, 3.74) |
| Subtotal (I-squared = N/A, p = N/A) | | 156/676 | 25/272 | 100.00 | **2.51 (1.69, 3.74)** |
| **PCSK+ST vs. EZT+ST (n = 4)** | | | | | |
| Bays, 2015 [39] | | 7/104 | 4/102 | 17.02 | 1.72 (0.52, 5.69) |
| Cannon, 2015 [23] | | 36/479 | 13/241 | 62.23 | 1.39 (0.75, 2.58) |
| Farnier, 2016 [22] | | 5/103 | 8/101 | 20.75 | 0.61 (0.21, 1.81) |
| Subtotal (I-squared = 2.1%, p = 0.360) | | 48/686 | 25/444 | 100.00 | 1.22 (0.74, 2.00) |

**Abbreviation:** CETP =cholesteryl ester transfer protein inhibito; EZT=ezetimibe; FBT=fibrate; N/A=not available NIA=niacin; OMG3=omega-3 fatty acids; PCSK=proprotein convertase subtilisin/kexin type 9 inhibitor; ST=statin monotherapy

Appendix 6 Network of comparisons

eFigure 6.1 Networks map of treatment comparisons for Coronary heart disease mortality

CETP/ST

EZT/ST

FBT/ST

NIA+EZT/ST

NIA/ST

OMG3/ST

PCSK/ST

ST

The size of each treatment nodes is related to the number of studies of the treatments. Each line represents the direct comparison between treatments, the thickness of the line corresponds is relate to the number of studies of the comparison. **Abbreviation:** CETP/ST=cholesteryl ester transfer protein inhibitor + statin; EZT/ST=ezetimibe + statin; FBT/ST=fibrate + statin; NIA/ST=niacin + statin; OMG3/ST=omega-3 fatty acids + statin; PCSK/ST=proprotein convertase subtilisin/kexin type 9 inhibitor + statin; NIA+EZT/ST= niacin + ezetimibe + statin; ST=statin monotherapy.

eFigure 6.2 Networks map of treatment comparisons for non-fatal myocardial infarction

CETP/ST

EZT/ST

FBT/ST

NIA/ST

OMG3/ST

PCSK/ST

ST

The size of each treatment nodes is related to the number of studies of the treatments. Each line represents the direct comparison between treatments, the thickness of the line corresponds is relate to the number of studies of the comparison. **Abbreviation:** CETP/ST=cholesteryl ester transfer protein inhibitor + statin; EZT/ST=ezetimibe + statin; FBT/ST=fibrate + statin; NIA/ST=niacin + statin; OMG3/ST=omega-3 fatty acids + statin; PCSK/ST=proprotein convertase subtilisin/kexin type 9 inhibitor + statin; ST=statin monotherapy.

eFigure 6.3 Networks map of treatment comparisons for any stroke

CETP/ST

EZT/ST

FBT/ST

NIA+EZT/ST

NIA/ST

OMG3/ST

PCSK/ST

ST

The size of each treatment nodes is related to the number of studies of the treatments. Each line represents the direct comparison between treatments, the thickness of the line corresponds is relate to the number of studies of the comparison. **Abbreviation:** CETP/ST=cholesteryl ester transfer protein inhibitor + statin; EZT/ST=ezetimibe + statin; FBT/ST=fibrate + statin; NIA/ST=niacin + statin; OMG3/ST=omega-3 fatty acids + statin; PCSK/ST=proprotein convertase subtilisin/kexin type 9 inhibitor + statin; NIA+EZT/ST= niacin + ezetimibe + statin; ST=statin monotherapy.

eFigure 6.4 Networks map of treatment comparisons for Coronary revascularization

CETP/ST

EZT/ST

NIA/ST

OMG3/ST

PCSK/ST

ST

The size of each treatment nodes is related to the number of studies of the treatments. Each line represents the direct comparison between treatments, the thickness of the line corresponds is relate to the number of studies of the comparison. **Abbreviation:** CETP/ST=cholesteryl ester transfer protein inhibitor + statin; EZT/ST=ezetimibe + statin; NIA/ST=niacin + statin; OMG3/ST=omega-3 fatty acids + statin; PCSK/ST=proprotein convertase subtilisin/kexin type 9 inhibitor + statin; ST=statin monotherapy.

eFigure 6.5 Networks map of treatment comparisons for discontinuation due to any cause

CETP/ST

EZT/ST

FBT/ST

NIA+EZT/ST

NIA/ST

OMG3/ST

PCSK/ST

ST

The size of each treatment nodes is related to the number of studies of the treatments. Each line represents the direct comparison between treatments, the thickness of the line corresponds is relate to the number of studies of the comparison. **Abbreviation:** CETP/ST=cholesteryl ester transfer protein inhibitor + statin; EZT/ST=ezetimibe + statin; FBT/ST=fibrate + statin; NIA/ST=niacin + statin; OMG3/ST=omega-3 fatty acids + statin; PCSK/ST=proprotein convertase subtilisin/kexin type 9 inhibitor + statin; NIA+EZT/ST= niacin + ezetimibe + statin; ST=statin monotherapy.

eFigure 6.6 Networks map of treatment comparisons for discontinuation due to adverse events

CETP/ST

EZT/ST

FBT/ST

NIA+EZT/ST

NIA/ST

OMG3/ST

PCSK/ST

ST

The size of each treatment nodes is related to the number of studies of the treatments. Each line represents the direct comparison between treatments, the thickness of the line corresponds is relate to the number of studies of the comparison. **Abbreviation:** CETP/ST=cholesteryl ester transfer protein inhibitor + statin; EZT/ST=ezetimibe + statin; FBT/ST=fibrate + statin; NIA/ST=niacin + statin; OMG3/ST=omega-3 fatty acids + statin; PCSK/ST=proprotein convertase subtilisin/kexin type 9 inhibitor + statin; NIA+EZT/ST= niacin + ezetimibe + statin; ST=statin monotherapy.

Appendix 7 Assessment of inconsistency for each outcome network

eTable 7.1 Evaluation of the global inconsistency in networks using the ‘design-by-treatment’ interaction model for each outcome.

| Network outcome | Chi-square | P value for test of global inconsistency |
| --- | --- | --- |
| **Primary outcomes** |  |  |
| Cardiovascular mortality | 4.28 | 0.2326 |
| All-cause mortality | 6.61 | 0.1579 |
| **Secondary outcomes** |  |  |
| Coronary heart disease mortality | 1.06 | 0.7870 |
| Non-fatal myocardial infarction | 2.20 | 0.5314 |
| Any stroke | 0.63 | 0.8904 |
| Coronary revascularization | 4.57 | 0.3347 |
| Discontinuation due to any cause | 2.00 | 0.7350 |
| Discontinuation due to any adverse event | 2.96 | 0.5653 |

Appendix 8 Transitivity

eTable 8.1 Explore distribution of covariables among treatment comparisons

| Intervention | | N | Mean age | Mean Follow-up duration (month) | Moderate-High intensity (%) | Primary/mixed prevention (%) |
| --- | --- | --- | --- | --- | --- | --- |
| CETP/ST | vs ST | 14 | 59.5 | 20.5 | 7 (50) | 11 (78.6) |
| EZT/ST | vs ST | 17 | 63.9 | 17.7 | 15 (88.2) | 6 (35.3) |
| FBT/ST | vs ST | 3 | 61.08 | 33 | 3 (100) | 2 (66.7) |
| NIA+EZT/ST | vs ST | 1 | 63 | 24 | 1 (100) | 0 (0) |
| NIA/ST | vs ST | 5 | 62.6 | 21.4 | 5 (100) | 2 (40) |
| OMG3/ST | vs ST | 8 | 64.6 | 20.5 | 4 (50) | 2 (25) |
| PCSK/ST | vs ST | 19 | 59.6 | 14.7 | 18 (94.7) | 17 (89.5) |
| NIA+EZT | vs EZT/ST | 1 | 57.2 | 6 | 1 (100) | 1 (100) |
| NIA/ST | vs EZT/ST | 1 | 65 | 14 | 1 (100) | 1 (100) |
| PCSK/ST | vs EZT/ST | 3 | 62.1 | 10.3 | 3 (100) | 3 (100) |

Mean age ranged from 57.2 to 65 years, in which they were not much different across all treatment comparisons. All comparisons had mean follow-up durations longer than 12 months, except two comparisons (i.e., NIA+EZT vs EZT/ST and PCSK/ST vs EZT/ST ) in which their mean durations were 6 and 10.3 months. Most comparisons had moderate to high intensity of statin except only two comparisons (i.e., CETP/ST vs ST and OMG3/ST vs ST) that had about 50%. About 6/10 treatment comparisons had indications as primary or mix of primary and secondary preventions, whereas the rest had indications as secondary preventions. Generally speaking, characteristics including age, duration of treatment, Intensity of stain, and indication of treatment were not much different across treatment interventions.

Note: Intensity of statin: combine moderate, moderate-high, and high; Indication of treatment: Primary and mix of primary and secondary preventions

Appendix 9 Result of network meta-analysis

eTable 9.1 Network estimated risk ratio (95% confidence intervals) of treatment options on cardiovascular mortality

| **FBT/ST** |  |  |  |  |  |  |  |
| --- | --- | --- | --- | --- | --- | --- | --- |
| 2.10  (0.04,106.56) | **NIA+EZT/ST** |  |  |  |  |  |  |
| 0.92  (0.69,1.22) | 0.44  (0.01,22.11) | **CETP/ST** |  |  |  |  |  |
| 0.89  (0.67,1.18) | 0.42  (0.01,21.34) | 0.96  (0.83,1.12) | **PCSK/ST** |  |  |  |  |
| 0.90  (0.60,1.34) | 0.43  (0.01,21.82) | 0.98  (0.71,1.34) | 1.01  (0.74,1.39) | **OMG3/ST** |  |  |  |
| 0.85  (0.63,1.13) | 0.40  (0.01,20.27) | 0.92  (0.79,1.07) | 0.95  (0.81,1.12) | 0.94  (0.68,1.29) | **EZT/ST** |  |  |
| 0.79  (0.59,1.05) | 0.37  (0.01,18.90) | 0.85  (0.73,1.00) | 0.89  (0.75,1.05) | 0.87  (0.63,1.21) | 0.93  (0.78,1.10) | **NIA/ST** |  |
| 0.86  (0.66,1.11) | 0.41  (0.01,20.53) | 0.93  (0.84,1.03) | 0.96  (0.86,1.08) | 0.95  (0.71,1.28) | 1.01  (0.90,1.13) | 1.09  (0.96,1.23) | **ST** |

Comparisons between treatments should be read from column to row for each outcome. Treatments are ordered by SUCRA rank (except the reference as the bottom row).

**Abbreviation:** CETP/ST=cholesteryl ester transfer protein inhibitor + statin; EZT/ST=ezetimibe + statin; FBT/ST=fibrate + statin; NIA/ST=niacin + statin; OMG3/ST=omega-3 fatty acids + statin; PCSK/ST=proprotein convertase subtilisin/kexin type 9 inhibitor + statin; NIA+EZT/ST= niacin + ezetimibe + statin; ST=statin monotherapy.

eTable 9.2 Network estimated risk ratio (95% confidence intervals) of treatment options on all-cause mortality

| **FBT/ST** |  |  |  |  |  |  |  |
| --- | --- | --- | --- | --- | --- | --- | --- |
| 0.97  (0.71,1.32) | **PCSK/ST** |  |  |  |  |  |  |
| 0.93  (0.67,1.29) | 0.95  (0.75,1.22) | **EZT/ST** |  |  |  |  |  |
| 0.97  (0.27,3.56) | 1.00  (0.28,3.60) | 1.05  (0.29,3.77) | **NIA+EZT/ST** |  |  |  |  |
| 0.89  (0.66,1.22) | 0.92  (0.74,1.15) | 0.97  (0.77,1.22) | 0.92  (0.26,3.30) | **CETP/ST** |  |  |  |
| 0.83  (0.60,1.16) | 0.86  (0.68,1.09) | 0.90  (0.69,1.17) | 0.86  (0.24,3.10) | 0.93  (0.73,1.19) | **NIA/ST** |  |  |
| 0.83  (0.60,1.14) | 0.85  (0.68,1.07) | 0.89  (0.70,1.15) | 0.85  (0.24,3.07) | 0.93  (0.74,1.15) | 0.99  (0.77,1.28) | **OMG3/ST** |  |
| 0.90  (0.68,1.18) | 0.93  (0.79,1.08) | 0.97  (0.80,1.17) | 0.92  (0.26,3.29) | 1.00  (0.87,1.15) | 1.08  (0.89,1.30) | 1.08  (0.92,1.28) | **ST** |

Comparisons between treatments should be read from column to row for each outcome. Treatments are ordered by SUCRA rank (except the reference as the bottom row).

**Abbreviation:** CETP/ST=cholesteryl ester transfer protein inhibitor + statin; EZT/ST=ezetimibe + statin; FBT/ST=fibrate + statin; NIA/ST=niacin + statin; OMG3/ST=omega-3 fatty acids + statin; PCSK/ST=proprotein convertase subtilisin/kexin type 9 inhibitor + statin; NIA+EZT/ST= niacin + ezetimibe + statin; ST=statin monotherapy.

eTable 9.3 Network estimated risk ratio (95% confidence intervals) of treatment options on coronary heart disease mortality

| **FBT/ST** |  |  |  |  |  |  |  |
| --- | --- | --- | --- | --- | --- | --- | --- |
| 0.86  (0.01,135.94) | **NIA+EZT/ST** |  |  |  |  |  |  |
| 0.38  (0.02,9.26) | 0.44  (0.01,22.03) | **PCSK/ST** |  |  |  |  |  |
| 0.36  (0.01,8.74) | 0.41  (0.01,20.82) | 0.95  (0.76,1.18) | **CETP/ST** |  |  |  |  |
| 0.35  (0.01,8.67) | 0.41  (0.01,20.61) | 0.93  (0.67,1.29) | 0.98  (0.73,1.32) | **OMG3/ST** |  |  |  |
| 0.35  (0.01,8.55) | 0.40  (0.01,20.27) | 0.93  (0.74,1.15) | 0.98  (0.82,1.16) | 0.99  (0.74,1.34) | **EZT/ST** |  |  |
| 0.32  (0.01,7.89) | 0.37  (0.01,18.79) | 0.85  (0.67,1.08) | 0.90  (0.75,1.09) | 0.92  (0.67,1.25) | 0.92  (0.76,1.12) | **NIA/ST** |  |
| 0.34  (0.01,8.20) | 0.39  (0.01,19.54) | 0.89  (0.74,1.07) | 0.94  (0.84,1.06) | 0.96  (0.73,1.26) | 0.96  (0.85,1.09) | 1.04  (0.90,1.21) | **ST** |

Comparisons between treatments should be read from column to row for each outcome. Treatments are ordered by SUCRA rank (except the reference as the bottom row).

**Abbreviation:** CETP/ST=cholesteryl ester transfer protein inhibitor + statin; EZT/ST=ezetimibe + statin; FBT/ST=fibrate + statin; NIA/ST=niacin + statin; OMG3/ST=omega-3 fatty acids + statin; PCSK/ST=proprotein convertase subtilisin/kexin type 9 inhibitor + statin; NIA+EZT/ST= niacin + ezetimibe + statin; ST=statin monotherapy.

eTable 9.4 Network estimated risk ratio (95% confidence intervals) of treatment options on non-fatal myocardial infarction

| **PCSK/ST** |  |  |  |  |  |  |
| --- | --- | --- | --- | --- | --- | --- |
| 1.03  (0.69,1.53) | **OMG3/ST** |  |  |  |  |  |
| 0.94  (0.74,1.20) | 0.92  (0.60,1.40) | **EZT/ST** |  |  |  |  |
| 0.87  (0.64,1.19) | 0.85  (0.53,1.35) | 0.92  (0.65,1.31) | **FBT/ST** |  |  |  |
| 0.84  (0.69,1.02) | 0.82  (0.55,1.23) | 0.90  (0.70,1.15) | 0.97  (0.71,1.33) | **CETP/ST** |  |  |
| 0.83  (0.65,1.05) | 0.80  (0.53,1.23) | 0.88  (0.66,1.17) | 0.95  (0.67,1.34) | 0.98  (0.77,1.25) | **NIA/ST** |  |
| **0.82**  **(0.72,0.93)^a^** | 0.80  (0.55,1.16) | 0.87  (0.71,1.07) | 0.94  (0.71,1.25) | 0.97  (0.84,1.12) | 0.99  (0.81,1.22) | **ST** |

Comparisons between treatments should be read from column to row for each outcome. Treatments are ordered by SUCRA rank (except the reference as the bottom row).

**Abbreviation:** CETP/ST=cholesteryl ester transfer protein inhibitor + statin; EZT/ST=ezetimibe + statin; FBT/ST=fibrate + statin; NIA/ST=niacin + statin; OMG3/ST=omega-3 fatty acids + statin; PCSK/ST=proprotein convertase subtilisin/kexin type 9 inhibitor + statin; ST=statin monotherapy.

^a^Significant results are in bold.

eTable 9.5 Network estimated risk ratio (95% confidence intervals) of treatment options on any stroke

| **NIA+EZT/ST** |  |  |  |  |  |  |  |
| --- | --- | --- | --- | --- | --- | --- | --- |
| 0.16  (0.01,3.92) | **PCSK/ST** |  |  |  |  |  |  |
| 0.13  (0.01,3.29) | 0.84  (0.69,1.03) | **EZT/ST** |  |  |  |  |  |
| 0.12  (0.00,2.91) | **0.74**  **(0.63,0.88)^a^** | 0.88  (0.74,1.05) | **CETP/ST** |  |  |  |  |
| 0.12  (0.00,2.90) | **0.74**  **(0.57,0.95) ^a^** | 0.87  (0.67,1.13) | 0.99  (0.78,1.25) | **OMG3/ST** |  |  |  |
| 0.11  (0.00,2.89) | 0.72  (0.48,1.09) | 0.85  (0.57,1.29) | 0.97  (0.65,1.45) | 0.98  (0.63,1.52) | **FBT/ST** |  |  |
| 0.12  (0.00,2.87) | **0.73**  **(0.61,0.87)^a^** | 0.87  (0.72,1.04) | 0.99  (0.84,1.15) | 0.99  (0.78,1.27) | 1.01  (0.68,1.52) | **NIA/ST** |  |
| 0.12  (0.00,2.90) | **0.74**  **(0.65,0.85)^a^** | 0.88  (0.76,1.02) | 1.00  (0.91,1.10) | 1.01  (0.81,1.25) | 1.03  (0.70,1.52) | 1.01  (0.90,1.14) | **ST** |

Comparisons between treatments should be read from column to row for each outcome. Treatments are ordered by SUCRA rank (except the reference as the bottom row).

**Abbreviation:** CETP/ST=cholesteryl ester transfer protein inhibitor + statin; EZT/ST=ezetimibe + statin; FBT/ST=fibrate + statin; NIA/ST=niacin + statin; OMG3/ST=omega-3 fatty acids + statin; PCSK/ST=proprotein convertase subtilisin/kexin type 9 inhibitor + statin; NIA+EZT/ST= niacin + ezetimibe + statin; ST=statin monotherapy.

^a^Significant results are in bold

eTable 9.6 Network estimated risk ratio (95% confidence intervals) of treatment options on coronary revascularization

| **PCSK/ST** |  |  |  |  |  |
| --- | --- | --- | --- | --- | --- |
| 0.96  (0.76,1.21) | **NIA/ST** |  |  |  |  |
| 0.93  (0.77,1.12) | 0.97  (0.75,1.25) | **OMG3/ST** |  |  |  |
| 0.92  (0.77,1.10) | 0.96  (0.76,1.23) | 0.99  (0.81,1.21) | **EZT/ST** |  |  |
| **0.83**  **(0.71,0.96)^a^** | 0.86  (0.69,1.08) | 0.89  (0.74,1.06) | 0.90  (0.76,1.06) | **CETP/ST** |  |
| **0.84**  **(0.75,0.94)^a^** | 0.88  (0.72,1.08) | 0.91  (0.78,1.06) | 0.92  (0.80,1.05) | 1.02  (0.93,1.12) | **ST** |

Comparisons between treatments should be read from column to row for each outcome. Treatments are ordered by SUCRA rank (except the reference as the bottom row).

**Abbreviation:** CETP/ST=cholesteryl ester transfer protein inhibitor + statin; EZT/ST=ezetimibe + statin; NIA/ST=niacin + statin; OMG3/ST=omega-3 fatty acids + statin; PCSK/ST=proprotein convertase subtilisin/kexin type 9 inhibitor + statin; ST=statin monotherapy.

^a^Significant results are in bold

eTable 9.7 Network estimated risk ratio (95% confidence intervals) of treatment options on Discontinuation due to any cause

| **PCSK/ST** |  |  |  |  |  |  |  |
| --- | --- | --- | --- | --- | --- | --- | --- |
| 0.97  (0.82,1.15) | **CETP/ST** |  |  |  |  |  |  |
| 0.95  (0.79,1.14) | 0.98  (0.80,1.20) | **EZT/ST** |  |  |  |  |  |
| 0.92  (0.68,1.24) | 0.94  (0.69,1.27) | 0.96  (0.70,1.33) | **FBT/ST** |  |  |  |  |
| 0.89  (0.72,1.11) | 0.92  (0.74,1.14) | 0.94  (0.74,1.20) | 0.98  (0.70,1.36) | **OMG3/ST** |  |  |  |
| **0.76**  **(0.61,0.93)^a^** | **0.78**  **(0.63,0.96)^a^** | 0.79  (0.63,1.00) | 0.83  (0.59,1.15) | 0.85  (0.66,1.09) | **NIA/ST** |  |  |
| **0.49**  **(0.32,0.75)^a^** | **0.50**  **(0.32,0.78)^a^** | **0.51**  **(0.35,0.76)^a^** | **0.53**  **(0.32,0.89)^a^** | **0.55**  **(0.35,0.87)^a^** | 0.65  (0.41,1.02) | **NIA+EZT/ST** |  |
| 1.04  (0.93,1.17) | 1.07  (0.95,1.21) | 1.09  (0.93,1.29) | 1.14  (0.86,1.50) | 1.17  (0.97,1.40) | **1.38**  **(1.15,1.65)^a^** | **2.13**  **(1.40,3.24)^a^** | **ST** |

Comparisons between treatments should be read from column to row for each outcome**.** Treatments are ordered by SUCRA rank (except the reference as the bottom row).

**Abbreviation:** CETP/ST=cholesteryl ester transfer protein inhibitor + statin; EZT/ST=ezetimibe + statin; FBT/ST=fibrate + statin; NIA/ST=niacin + statin; OMG3/ST=omega-3 fatty acids + statin; PCSK/ST=proprotein convertase subtilisin/kexin type 9 inhibitor + statin; NIA+EZT/ST= niacin + ezetimibe + statin; ST=statin monotherapy.

^a^Significant results are in bold

eTable 9.8 Network estimated risk ratio (95% confidence intervals) of treatment options on Discontinuation due to any adverse event

| **EZT/ST** |  |  |  |  |  |  |  |
| --- | --- | --- | --- | --- | --- | --- | --- |
| 0.84  (0.65,1.08) | **PCSK/ST** |  |  |  |  |  |  |
| 0.78  (0.61,1.00) | 0.93  (0.75,1.16) | **CETP/ST** |  |  |  |  |  |
| **0.61**  **(0.44,0.85)^a^** | 0.73  (0.54,1.00) | 0.78  (0.58,1.05) | **OMG3/ST** |  |  |  |  |
| **0.48**  **(0.36,0.64)^a^** | **0.57**  **(0.44,0.75)^a^** | **0.61**  **(0.48,0.79)^a^** | 0.78  (0.56,1.10) | **NIA/ST** |  |  |  |
| **0.45**  **(0.25,0.81)^a^** | **0.54**  **(0.30,0.95)^a^** | 0.58  (0.33,1.01) | 0.73  (0.40,1.34) | 0.94  (0.52,1.68) | **FBT/ST** |  |  |
| **0.40**  **(0.24,0.66)^a^** | **0.47**  **(0.27,0.83)^a^** | **0.51**  **(0.29,0.89)^a^** | 0.65  (0.36,1.18) | 0.83  (0.47,1.47) | 0.88  (0.41,1.90) | **NIA+EZT/ST** |  |
| 0.90  (0.74,1.11) | 1.08  (0.91,1.28) | 1.15  (1.00,1.33) | **1.47**  **(1.14,1.91)^a^** | **1.88**  **(1.52,2.32)^a^** | **2.00**  **(1.16,3.46)^a^** | **2.27**  **(1.33,3.88)^a^** | **ST** |

Comparisons between treatments should be read from column to row for each outcome**.** Treatments are ordered by SUCRA rank (except the reference as the bottom row).

**Abbreviation:** CETP/ST=cholesteryl ester transfer protein inhibitor + statin; EZT/ST=ezetimibe + statin; FBT/ST=fibrate + statin; NIA/ST=niacin + statin; OMG3/ST=omega-3 fatty acids + statin; PCSK/ST=proprotein convertase subtilisin/kexin type 9 inhibitor + statin; NIA+EZT/ST= niacin + ezetimibe + statin; ST=statin monotherapy.

^a^Significant results are in bold

Appendix 10 Treatment ranking and surface under the cumulative ranking curves (SUCRA) for each outcome

eFigure 10.1 SUCRA ranking curve for cardiovascular mortality

**Abbreviation:** CETP/ST=cholesteryl ester transfer protein inhibitor + statin; EZT/ST=ezetimibe + statin; FBT/ST=fibrate + statin; NIA/ST=niacin + statin; OMG3/ST=omega-3 fatty acids + statin; PCSK/ST=proprotein convertase subtilisin/kexin type 9 inhibitor + statin; NIA+EZT/ST= niacin + ezetimibe + statin; ST=statin monotherapy.

eTable 10.1 SUCRA ranking for cardiovascular mortality

| Rank^a^ | Treatment | Cumulative probability (%) |
| --- | --- | --- |
| 1 | FBT/ST | 74.9 |
| 2 | NIA+EZT/ST | 67.1 |
| 3 | CETP/ST | 66 |
| 4 | PCSK/ST | 53.4 |
| 5 | OMG3/ST | 51.9 |
| 6 | ST | 37.8 |
| 7 | EZT/ST | 35 |
| 8 | NIA/ST | 13.9 |

**Abbreviation:** CETP/ST=cholesteryl ester transfer protein inhibitor + statin; EZT/ST=ezetimibe + statin; FBT/ST=fibrate + statin; NIA/ST=niacin + statin; OMG3/ST=omega-3 fatty acids + statin; PCSK/ST=proprotein convertase subtilisin/kexin type 9 inhibitor + statin; NIA+EZT/ST= niacin + ezetimibe + statin; ST=statin monotherapy.

^a^Ranking SUCRA probabilities in order: being the best treatment, the second best, the third best, and so on, among the interventions.

eFigure 10.2 SUCRA ranking curve for all-cause mortality

**Abbreviation:** CETP/ST=cholesteryl ester transfer protein inhibitor + statin; EZT/ST=ezetimibe + statin; FBT/ST=fibrate + statin; NIA/ST=niacin + statin; OMG3/ST=omega-3 fatty acids + statin; PCSK/ST=proprotein convertase subtilisin/kexin type 9 inhibitor + statin; NIA+EZT/ST= niacin + ezetimibe + statin; ST=statin monotherapy.

eTable 10.2 SUCRA ranking for cardiovascular mortality

| Rank^a^ | Treatment | Cumulative probability (%) |
| --- | --- | --- |
| 1 | FBT/ST | 72.2 |
| 2 | PCSK/ST | 71.4 |
| 3 | EZT/ST | 56.9 |
| 4 | NIA+EZT/ST | 54.5 |
| 5 | ST | 47.5 |
| 6 | CETP/ST | 46.5 |
| 7 | NIA/ST | 27.1 |
| 8 | OMG3/ST | 24 |

**Abbreviation:** CETP/ST=cholesteryl ester transfer protein inhibitor + statin; EZT/ST=ezetimibe + statin; FBT/ST=fibrate + statin; NIA/ST=niacin + statin; OMG3/ST=omega-3 fatty acids + statin; PCSK/ST=proprotein convertase subtilisin/kexin type 9 inhibitor + statin; NIA+EZT/ST= niacin + ezetimibe + statin; ST=statin monotherapy.

^a^Ranking SUCRA probabilities in order: being the best treatment, the second best, the third best, and so on, among the interventions.

eFigure 10.3 SUCRA ranking curve for coronary heart disease mortality

**Abbreviation:** CETP/ST=cholesteryl ester transfer protein inhibitor + statin; EZT/ST=ezetimibe + statin; FBT/ST=fibrate + statin; NIA/ST=niacin + statin; OMG3/ST=omega-3 fatty acids + statin; PCSK/ST=proprotein convertase subtilisin/kexin type 9 inhibitor + statin; NIA+EZT/ST= niacin + ezetimibe + statin; ST=statin monotherapy.

eTable 10.3 SUCRA ranking for coronary heart disease mortality

| Rank^a^ | Treatment | Cumulative probability (%) |
| --- | --- | --- |
| 1 | FBT/ST | 71.5 |
| 2 | NIA+EZT/ST | 65.0 |
| 3 | PCSK/ST | 64.6 |
| 4 | CETP/ST | 53.4 |
| 5 | OMG3/ST | 46.0 |
| 6 | EZT/ST | 45.7 |
| 7 | ST | 31.0 |
| 8 | NIA/ST | 22.7 |

**Abbreviation:** CETP/ST=cholesteryl ester transfer protein inhibitor + statin; EZT/ST=ezetimibe + statin; FBT/ST=fibrate + statin; NIA/ST=niacin + statin; OMG3/ST=omega-3 fatty acids + statin; PCSK/ST=proprotein convertase subtilisin/kexin type 9 inhibitor + statin; NIA+EZT/ST= niacin + ezetimibe + statin; ST=statin monotherapy.

^a^Ranking SUCRA probabilities in order: being the best treatment, the second best, the third best, and so on, among the interventions.

eFigure 10.4 SUCRA ranking curve for non-fatal myocardial infarction

**Abbreviation:** CETP/ST=cholesteryl ester transfer protein inhibitor + statin; EZT/ST=ezetimibe + statin; FBT/ST=fibrate + statin; NIA/ST=niacin + statin; OMG3/ST=omega-3 fatty acids + statin; PCSK/ST=proprotein convertase subtilisin/kexin type 9 inhibitor + statin; ST=statin monotherapy.

eTable 10.4 SUCRA ranking for non-fatal myocardial infarction

| Rank^a^ | Treatment | Cumulative probability (%) |
| --- | --- | --- |
| 1 | PCSK/ST | 81 |
| 2 | OMG3/ST | 75.3 |
| 3 | EZT/ST | 64.1 |
| 4 | FBT/ST | 43.2 |
| 5 | CETP/ST | 33.6 |
| 6 | NIA/ST | 29.7 |
| 7 | ST | 23.2 |

**Abbreviation:** CETP/ST=cholesteryl ester transfer protein inhibitor + statin; EZT/ST=ezetimibe + statin; FBT/ST=fibrate + statin; NIA/ST=niacin + statin; OMG3/ST=omega-3 fatty acids + statin; PCSK/ST=proprotein convertase subtilisin/kexin type 9 inhibitor + statin; ST=statin monotherapy.

^a^Ranking SUCRA probabilities in order: being the best treatment, the second best, the third best, and so on, among the interventions.

eFigure 10.5 SUCRA ranking curve for any stroke

**Abbreviation:** CETP/ST=cholesteryl ester transfer protein inhibitor + statin; EZT/ST=ezetimibe + statin; FBT/ST=fibrate + statin; NIA/ST=niacin + statin; OMG3/ST=omega-3 fatty acids + statin; PCSK/ST=proprotein convertase subtilisin/kexin type 9 inhibitor + statin; NIA+EZT/ST= niacin + ezetimibe + statin; ST=statin monotherapy.

eTable 10.5 SUCRA ranking for any stroke

| Rank^a^ | Treatment | Cumulative probability (%) |
| --- | --- | --- |
| 1 | NIA+EZT/ST | 89.6 |
| 2 | PCSK/ST | 86 |
| 3 | EZT/ST | 65.8 |
| 4 | CETP/ST | 33.4 |
| 5 | ST | 33 |
| 6 | OMG3/ST | 32 |
| 7 | FBT/ST | 31.8 |
| 8 | NIA/ST | 28.5 |

**Abbreviation:** CETP/ST=cholesteryl ester transfer protein inhibitor + statin; EZT/ST=ezetimibe + statin; FBT/ST=fibrate + statin; NIA/ST=niacin + statin; OMG3/ST=omega-3 fatty acids + statin; PCSK/ST=proprotein convertase subtilisin/kexin type 9 inhibitor + statin; NIA+EZT/ST= niacin + ezetimibe + statin; ST=statin monotherapy.

^a^Ranking SUCRA probabilities in order: being the best treatment, the second best, the third best, and so on, among the interventions.

eFigure 10.6 SUCRA ranking curve for coronary revascularization

**Abbreviation:** CETP/ST=cholesteryl ester transfer protein inhibitor + statin; EZT/ST=ezetimibe + statin; NIA/ST=niacin + statin; OMG3/ST=omega-3 fatty acids + statin; PCSK/ST=proprotein convertase subtilisin/kexin type 9 inhibitor + statin; ST=statin monotherapy.

eTable 10.6 SUCRA ranking for coronary revascularization

| Rank^a^ | Treatment | Cumulative probability (%) |
| --- | --- | --- |
| 1 | PCSK/ST | 85 |
| 2 | NIA/ST | 67.5 |
| 3 | OMG3/ST | 58.4 |
| 4 | EZT/ST | 56.6 |
| 5 | ST | 19.9 |
| 6 | CETP/ST | 12.5 |

**Abbreviation:** CETP/ST=cholesteryl ester transfer protein inhibitor + statin; EZT/ST=ezetimibe + statin; OMG3/ST=omega-3 fatty acids + statin; PCSK/ST=proprotein convertase subtilisin/kexin type 9 inhibitor + statin; ST=statin monotherapy.

^a^Ranking SUCRA probabilities in order: being the best treatment, the second best, the third best, and so on, among the interventions.

eFigure 10.7 SUCRA ranking curve for discontinuation due to any cause

**Abbreviation:** CETP/ST=cholesteryl ester transfer protein inhibitor + statin; EZT/ST=ezetimibe + statin; FBT/ST=fibrate + statin; NIA/ST=niacin + statin; OMG3/ST=omega-3 fatty acids + statin; PCSK/ST=proprotein convertase subtilisin/kexin type 9 inhibitor + statin; NIA+EZT/ST= niacin + ezetimibe + statin; ST=statin monotherapy.

eTable 10.7 SUCRA ranking for discontinuation due to any cause

| Rank^a^ | Treatment | Cumulative probability (%) |
| --- | --- | --- |
| 1 | ST | 89 |
| 2 | PCSK/ST | 73.8 |
| 3 | CETP/ST | 64.9 |
| 4 | EZT/ST | 58.5 |
| 5 | FBT/ST | 52.1 |
| 6 | OMG3/ST | 43.5 |
| 7 | NIA/ST | 17.7 |
| 8 | NIA+EZT/ST | 0.6 |

**Abbreviation:** CETP/ST=cholesteryl ester transfer protein inhibitor + statin; EZT/ST=ezetimibe + statin; FBT/ST=fibrate + statin; NIA/ST=niacin + statin; OMG3/ST=omega-3 fatty acids + statin; PCSK/ST=proprotein convertase subtilisin/kexin type 9 inhibitor + statin; NIA+EZT/ST= niacin + ezetimibe + statin; ST=statin monotherapy.

^a^Ranking SUCRA probabilities in order: being the best treatment, the second best, the third best, and so on, among the interventions.

eFigure 10.8 SUCRA ranking curve for discontinuation due to adverse events

**Abbreviation:** CETP/ST=cholesteryl ester transfer protein inhibitor + statin; EZT/ST=ezetimibe + statin; FBT/ST=fibrate + statin; NIA/ST=niacin + statin; OMG3/ST=omega-3 fatty acids + statin; PCSK/ST=proprotein convertase subtilisin/kexin type 9 inhibitor + statin; NIA+EZT/ST= niacin + ezetimibe + statin; ST=statin monotherapy.

eTable 10.8 SUCRA ranking for discontinuation due to adverse events

| Rank^a^ | Treatment | Cumulative probability (%) |
| --- | --- | --- |
| 1 | EZT/ST | 96 |
| 2 | ST | 84.8 |
| 3 | PCSK/ST | 70.9 |
| 4 | CETP/ST | 60.5 |
| 5 | OMG3/ST | 39.6 |
| 6 | NIA/ST | 20 |
| 7 | FBT/ST | 17.8 |
| 8 | NIA+EZT/ST | 10.4 |

**Abbreviation:** CETP/ST=cholesteryl ester transfer protein inhibitor + statin; EZT/ST=ezetimibe + statin; FBT/ST=fibrate + statin; NIA/ST=niacin + statin; OMG3/ST=omega-3 fatty acids + statin; PCSK/ST=proprotein convertase subtilisin/kexin type 9 inhibitor + statin; NIA+EZT/ST= niacin + ezetimibe + statin; ST=statin monotherapy.

^a^Ranking SUCRA probabilities in order: being the best treatment, the second best, the third best, and so on, among the interventions.

Appendix 11 The lists of included studies for the network meta-analysis of primary and secondary outcomes

| **Author** | **Published**  **Year** | **Primary outcomes** | | **Secondary outcomes** | | | | | |
| --- | --- | --- | --- | --- | --- | --- | --- | --- | --- |
|  |  | **Cardiovascular mortality** | **All-cause mortality** | **CHD death** | **Nonfatal MI** | **Stroke** | **Coronary revascularization** | **All-cause discontinuation** | **Discontinuation from any adverse event** |
| Cannon [1] | 2015 | √ | √ | √ | √ | √ | √ | √ | √ |
| Kastelein [2] | 2008 | √ | N/A | N/A | N/A | N/A | N/A | √ | √ |
| Taylor [3] | 2004 | N/A | √ | N/A | N/A | √ | N/A | √ | √ |
| Boden [4] | 2011 | √ | √ | √ | √ | √ | N/A | √ | √ |
| Landray [5] | 2014 | √ | √ | √ | √ | √ | √ | √ | √ |
| Ginsberg [6] | 2010 | √ | √ | N/A | √ | √ | N/A | √ | N/A |
| Yokoyama [7] | 2007 | N/A | √ | √ | √ | √ | √ | √ | √ |
| Nissen [8] | 2007 | N/A | √ | √ | √ | √ | √ | √ | √ |
| Barter [9] | 2007 | √ | √ | √ | √ | √ | N/A | √ | √ |
| Schwartz [10] | 2012 | N/A | √ | √ | √ | √ | √ | N/A | N/A |
| Robinson [11] | 2015 | N/A | √ | √ | √ | √ | √ | √ | √ |
| Sabatine [12] | 2015 | √ | √ | N/A | N/A | √ | √ | √ | N/A |
| Taylor [13] | 2009 | √ | √ | N/A | N/A | N/A | √ | √ | √ |
| Guyton [14] | 2008 | √ | √ | √ | N/A | √ | N/A | √ | √ |
| Brunner [15] | 2013 | N/A | √ | N/A | N/A | N/A | N/A | N/A | N/A |
| Ballantyne [16] | 2008 | √ | √ | √ | N/A | √ | N/A | √ | √ |
| Wang [17] | 2016 | N/A | N/A | √ | N/A | √ | N/A | √ | √ |
| Tsujita [18] | 2015 | √ | √ | √ | √ | N/A | √ | √ | √ |
| Masuda [19] | 2015 | √ | √ | √ | √ | √ | √ | √ | √ |
| Luo [20] | 2014 | √ | N/A | N/A | √ | N/A | N/A | N/A | N/A |
| Rauch [21] | 2010 | N/A | √ | N/A | N/A | N/A | √ | √ | N/A |
| Farnier [22] | 2016 | √ | N/A | √ | √ | √ | √ | √ | √ |
| Cannon [23] | 2015 | N/A | N/A | √ | √ | √ | √ | √ | √ |
| Blom [24] | 2014 | √ | √ | √ | N/A | N/A | N/A | √ | √ |
| West [25] | 2011 | N/A | √ | N/A | N/A | √ | N/A | √ | √ |
| Arimura [26] | 2012 | N/A | N/A | √ | N/A | √ | √ | √ | √ |
| Bots [27] | 2007 | √ | √ | √ | N/A | √ | N/A | √ | √ |
| Kastelein [28] | 2015 | √ | √ | √ | N/A | N/A | N/A | √ | √ |
| Cannon [29] | 2010 | √ | √ | N/A | √ | N/A | √ | √ | √ |
| Davidson [30] | 2014 | √ | √ | √ | √ | √ | N/A | √ | √ |
| Kastelein [31] | 2007 | √ | N/A | N/A | N/A | √ | N/A | √ | √ |
| Derosa [32] | 2004 | N/A | N/A | N/A | √ | N/A | N/A | √ | √ |
| Durrington [33] | 2001 | √ | √ | √ | N/A | N/A | N/A | √ | √ |
| Kastelein [34] | 2015 | N/A | N/A | √ | √ | √ | √ | √ | √ |
| Kereiakes [35] | 2015 | N/A | N/A | √ | √ | √ | √ | √ | √ |
| Nishio [36] | 2014 | √ | √ | √ | √ | N/A | √ | √ | √ |
| Teramoto [37] | 2016 | N/A | N/A | N/A | √ | N/A | √ | √ | √ |
| Stein [38] | 2010 | √ | √ | √ | N/A | N/A | N/A | √ | √ |
| Bays [39] | 2015 | √ | √ | √ | N/A | N/A | √ | √ | √ |
| Fayad [40] | 2011 | √ | √ | √ | N/A | N/A | N/A | √ | √ |
| Luscher [41] | 2012 | N/A | N/A | √ | N/A | √ | N/A | √ | √ |
| Ballantyne [42] | 2008 | √ | √ | √ | N/A | N/A | N/A | √ | √ |
| Nicholls [43] | 2016 | N/A | √ | N/A | √ | √ | √ | √ | √ |
| Landray [44] | 2006 | √ | √ | N/A | N/A | N/A | N/A | √ | √ |
| Shaw [45] | 2009 | N/A | √ | N/A | N/A | N/A | N/A | √ | √ |
| Kouvelos [46] | 2013 | √ | N/A | √ | √ | N/A | N/A | N/A | √ |
| Ginsberg [47] | 2016 | √ | √ | √ | √ | N/A | √ | √ | √ |
| Ballantyne [48] | 2017 | N/A | √ | N/A | N/A | N/A | N/A | √ | √ |
| Kromhout [49] | 2010 | √ | √ | √ | N/A | N/A | N/A | √ | √ |
| Sabatine [50] | 2017 | √ | √ | N/A | √ | √ | √ | √ | √ |
| Ridker [51] | 2017 | √ | √ | N/A | √ | √ | √ | √ | √ |
| Ridker [52] | 2017 | √ | N/A | N/A | √ | √ | √ | N/A | √ |
| Luo [53] | 2016 | √ | N/A | √ | √ | √ | √ | N/A | N/A |
| Liu [54] | 2017 | N/A | N/A | √ | N/A | √ | √ | N/A | N/A |
| Nosaka [55] | 2017 | √ | √ | N/A | √ | √ | √ | N/A | N/A |
| Lincoff [56] | 2017 | N/A | √ | N/A | N/A | √ | √ | √ | √ |
| Bowman [57] | 2017 | √ | √ | √ | √ | √ | √ | √ | √ |
| Hagiwara [58] | 2017 | N/A | √ | N/A | √ | √ | √ | N/A | √ |
| Hibi [59] | 2018 | √ | √ | √ | N/A | N/A | √ | √ | √ |
| Miyoshi [60] | 2018 | √ | N/A | √ | √ | √ | N/A | √ | N/A |
| Watanabe [61] | 2017 | N/A | √ | √ | √ | √ | √ | √ | √ |
| Koh [62] | 2018 | √ | √ | √ | √ | √ | √ | √ | √ |
| Leiter [63] | 2017 | √ | √ | √ | N/A | N/A | N/A | √ | √ |
| Teramoto [64] | 2017 | √ | √ | √ | √ | √ | N/A | √ | √ |
| Ballantyne [65] | 2017 | √ | √ | √ | √ | √ | N/A | √ | √ |
| Ray [66] | 2018 | N/A | √ | N/A | N/A | N/A | N/A | √ | √ |
| Schwartz [67] | 2018 | √ | √ | √ | √ | √ | √ | √ | N/A |

**Abbreviation:** CHD=coronary heart disease; MI=myocardial infarction

Appendix 12 Subgroup analyses

12.1 Subgroup analyses of indication of therapy with treatment options

eTable 12.1.1 the risk of cardiovascular mortality

The following table shows the effect sizes (risk ratio) and the rank order (SUCRA ranks) compared stain monotherapy before (standard analysis) and after subgroup analyses.

| **Treatment** | **Standard analysis** | **SUCRA rank** | **Primary prevention** | **SUCRA rank** | **Secondary prevention** | **SUCRA rank** | **Mixed indication^a^** | **SUCRA rank** |
| --- | --- | --- | --- | --- | --- | --- | --- | --- |
| FBT/ST | 0.86  (0.66,1.11) | 1 | N/A | N/A | N/A | N/A | 0.86  (0.66,1.11) | 1 |
| NIA+EZT/ST | 0.41  (0.01,20.53) | 2 | N/A | N/A | N/A | N/A | 1.27  (0.02,79.13) | 5 |
| CETP/ST | 0.93  (0.84,1.03) | 3 | 0.51  (0.06,4.14) | 1 | 0.91  (0.82,1.01) | 1 | 1.37  (0.90,2.07) | 6 |
| PCSK/ST | 0.96  (0.86,1.08) | 4 | N/A | N/A | 0.96  (0.85,1.09) | 2 | 1.00  (0.73,1.38) | 3 |
| OMG3/ST | 0.95  (0.71,1.28) | 5 | 1.90  (0.04,94.65) | 2 | 0.95  (0.70,1.28) | 3 | N/A | N/A |
| ST (reference) | 1.00 | 6 | 1.00 | 3 | 1.00 | 5 | 1.00 | 4 |
| EZT/ST | 1.01  (0.90,1.13) | 7 | 2.03  (0.19,22.33) | 4 | 1.00  (0.89,1.12) | 4 | 3.14  (0.83,11.81) | 7 |
| NIA/ST | 1.09  (0.96,1.23) | 8 | N/A | N/A | 1.10  (0.97,1.24) | 6 | 0.59  (0.09,3.90) | 2 |
| Global inconsistency chi^2^ (P value) | 4.28  (P=0.2326) |  | 0.1  (P=0.7479) |  | 0.42  (P=0.5184) |  | 0.03  (P=0.9989) |  |
| Number of studies | 44 |  | 5 |  | 16 |  | 23 |  |

^a^Not classified in primary or secondary prevention

eTable 12.1.2 The risk of all-cause mortality

The following table shows the effect sizes (risk ratio) and the rank order (SUCRA ranks) compared stain monotherapy before (standard analysis) and after subgroup analyses.

| **Treatment** | **Standard analysis** | **SUCRA rank** | **Primary prevention** | **SUCRA rank** | **Secondary prevention** | **SUCRA rank** | **Mixed indication^a^** | **SUCRA rank** |
| --- | --- | --- | --- | --- | --- | --- | --- | --- |
| FBT/ST | 0.90  (0.68,1.18) | 1 | N/A | N/A | N/A | N/A | 0.86  (0.52,1.42 | 3 |
| PCSK/ST | 0.93  (0.79,1.08) | 2 | N/A | N/A | 0.95  (0.82,1.09) | 2 | 0.79  (0.48,1.32) | 2 |
| EZT/ST | 0.97  (0.80,1.17) | 3 | 0.33  (0.01,7.91) | 1 | 0.95  (0.81,1.12) | 3 | 4.75  (0.95,23.76) | 8 |
| NIA+EZT/ST | 0.92  (0.26,3.29) | 4 | N/A | N/A | 1.02  (0.27,3.88 | 4 | 1.91  (0.03,135.45) | 6 |
| ST (reference) | 1.00 | 5 | 1.00 | 3 | 1.00 | 5 | 1.00 | 4 |
| CETP/ST | 1.00  (0.87,1.15) | 6 | 0.79  (0.08,7.59) | 2 | 0.94  (0.84,1.05) | 1 | 1.38  (0.88,2.18) | 7 |
| NIA/ST | 1.08  (0.89,1.30) | 7 | N/A | N/A | 1.10  (0.95,1.28) | 7 | 0.47  (0.07,3.06) | 1 |
| OMG3/ST | 1.08  (0.92,1.28) | 8 | N/A | N/A | 1.06  (0.87,1.29) | 6 | 1.12  (0.69,1.80) | 5 |
| Global inconsistency chi^2^ (P value) | 6.61  (P=0.1579) |  | 0.46  (P=0.4965) |  | 0.59  (P=0.4406) |  | 0.35  (P=0.9512) |  |
| Number of studies | 50 |  | 3 |  | 21 |  | 26 |  |

^a^Not classified in primary or secondary prevention

eTable 12.1.3 The risk of coronary heart disease mortality

The following table shows the effect sizes (risk ratio) and the rank order (SUCRA ranks) compared stain monotherapy before (standard analysis) and after subgroup analyses.

| **Treatment** | **Standard analysis** | **SUCRA rank** | **Primary prevention** | **SUCRA rank** | **Secondary prevention** | **SUCRA rank** | **Mixed indication^*^** | **SUCRA rank** |
| --- | --- | --- | --- | --- | --- | --- | --- | --- |
| FBT/ST | 0.34  (0.01,8.20) | 1 | N/A | N/A | N/A | N/A | 0.34  (0.01,8.20) | 2 |
| NIA+EZT/ST | 0.39  (0.01,19.54) | 2 | N/A | N/A | N/A | N/A | 0.52  (0.01,35.39) | 3 |
| PCSK/ST | 0.89  (0.74,1.07) | 3 | N/A | N/A | 0.92  (0.77,1.11) | 2 | 0.52  (0.22,1.21) | 1 |
| CETP/ST | 0.94  (0.84,1.06) | 4 | 0.71  (0.04,11.26) | 1 | 0.93  (0.82,1.05) | 1 | 1.12  (0.73,1.74) | 7 |
| OMG3/ST | 0.96  (0.73,1.26) | 5 | 1.90  (0.04,94.65) | 3 | 0.96  (0.70,1.33) | 4 | 0.93  (0.56,1.55) | 5 |
| EZT/ST | 0.96  (0.85,1.09) | 6 | N/A | N/A | 0.96  (0.84,1.08) | 3 | 1.30  (0.28,6.10) | 8 |
| ST (reference) | 1.00 | 7 | 1.00 | 2 | 1.00 | 5 | 1.00 | 6 |
| NIA/ST | 1.04  (0.90,1.21) | 8 | N/A | N/A | 1.04  (0.90,1.21) | 6 | 0.59  (0.04,9.45) | 4 |
| Global inconsistency chi^2^ (P value) | 1.06  (P=0.787) |  | 0.1  (P=0.7479) |  | 0.69  (P=0.4055) |  | 0.51  (P=0.7753) |  |
| Number of studies | 43 |  | 3 |  | 18 |  | 22 |  |

^*^ Not classified in primary or secondary prevention

eTable 12.1.4 The risk of non-fatal myocardial infarction

The following table shows the effect sizes (risk ratio) and the rank order (SUCRA ranks) compared stain monotherapy before (standard analysis) and after subgroup analyses.

| **Treatment** | **Standard analysis** | **SUCRA rank** | **Primary prevention** | **SUCRA rank** | **Secondary prevention** | **SUCRA rank** | **Mixed indication^a^** | **SUCRA rank** |
| --- | --- | --- | --- | --- | --- | --- | --- | --- |
| PCSK/ST | **0.82**  **(0.72,0.93)^b^** | 1 | N/A | N/A | **0.80**  **(0.68,0.93)^b^** | 1 | 0.81  (0.56,1.16) | 3 |
| OMG3/ST | 0.80  (0.55,1.16) | 2 | N/A | N/A | 2.39  (0.45,12.63) | 7 | 0.75  (0.41,1.36) | 2 |
| EZT/ST | 0.87  (0.71,1.07) | 3 | N/A | N/A | 0.88  (0.72,1.07) | 2 | 0.64  (0.20,2.09) | 1 |
| FBT/ST | 0.94  (0.71,1.25) | 4 | N/A | N/A | 0.92  (0.02,44.92) | 4 | 0.98  (0.57,1.70) | 4 |
| CETP/ST | 0.97  (0.84,1.12) | 5 | N/A | N/A | 0.94  (0.80,1.10) | 3 | 0.98  (0.60,1.59) | 5 |
| NIA/ST | 0.99  (0.81,1.22) | 6 | N/A | N/A | 0.99  (0.82,1.20) | 5 | N/A | N/A |
| ST (reference) | 1.00 | 7 | N/A | N/A | 1.00 | 6 | 1.00 | 6 |
| Global inconsistency chi^2^ (P value) | 2.2  (P=0.5314) |  | N/A |  | 8.41  (P=0.0037) |  | 1.78  (P=0.6201) |  |
| Number of studies | 37 |  | 1 |  | 16 |  | 20 |  |

^a^Not classified in primary or secondary prevention

^b^Significant results are in bold

eTable 12.1.5 The risk of any stroke

The following table shows the effect sizes (risk ratio) and the rank order (SUCRA ranks) compared stain monotherapy before (standard analysis) and after subgroup analyses.

| **Treatment** | **Standard analysis** | **SUCRA rank** | **Primary prevention** | **SUCRA rank** | **Secondary prevention** | **SUCRA rank** | **Mixed indication^a^** | **SUCRA rank** |
| --- | --- | --- | --- | --- | --- | --- | --- | --- |
| NIA+EZT/ST | 0.12  (0.005,2.90) | 1 | N/A | N/A | N/A | N/A | 0.10  (0.00,4.53) | 1 |
| PCSK/ST | **0.74**  **(0.65,0.85)^b^** | 2 | N/A | N/A | **0.77**  **(0.66,0.89)^b^** | 2 | **0.63**  **(0.46,0.87)^b^** | 3 |
| EZT/ST | 0.88  (0.76,1.02) | 3 | N/A | N/A | 0.88  (0.76,1.02) | 3 | 0.75  (0.09,5.93) | 4 |
| CETP/ST | 1.00  (0.91,1.10) | 4 | 1.59  (0.20,12.89) | 2 | 1.00  (0.91,1.11) | 5 | 0.95  (0.63,1.42) | 5 |
| ST (reference) | 1.00 | 5 | 1.00 | 1 | 1.00 | 4 | 1.00 | 6 |
| OMG3/ST | 1.01  (0.81,1.25) | 6 | 1.90  (0.04,94.65) | 3 | 0.18  (0.02,1.56) | 1 | 1.02  (0.83,1.27) | 8 |
| FBT/ST | 1.03  (0.70,1.52) | 7 | N/A | N/A | N/A | N/A | 1.03  (0.70,1.52) | 7 |
| NIA/ST | 1.01  (0.90,1.14) | 8 | N/A | N/A | 1.02  (0.90,1.15) | 6 | 0.21  (0.01,5.10) | 2 |
| Global inconsistency chi^2^ (P value) | 0.63  (P=0.8904) |  | 0.1  (P=0.7479) |  | 12.71  (P=0.0004) |  | 0.46  (P=0.7932) |  |
| Number of studies | 41 |  | 3 |  | 18 |  | 20 |  |

^a^Not classified in primary or secondary prevention

^b^Significant results are in bold

eTable 12.1.6 The risk of coronary revascularization

The following table shows the effect sizes (risk ratio) and the rank order (SUCRA ranks) compared stain monotherapy before (standard analysis) and after subgroup analyses.

| **Treatment** | **Standard analysis** | **SUCRA rank** | **Primary prevention** | **SUCRA rank** | **Secondary prevention** | **SUCRA rank** | **Mixed indication^a^** | **SUCRA rank** |
| --- | --- | --- | --- | --- | --- | --- | --- | --- |
| PCSK/ST | **0.84**  **(0.75,0.94)^b^** | 1 | N/A | N/A | **0.83**  **(0.76,0.91)^b^** | 1 | 0.84  (0.62,1.15) | 3 |
| NIA/ST | 0.88  (0.72,1.08) | 2 | N/A | N/A | 0.89  (0.77,1.02) | 2 | 0.06  (0.00,1.53) | 1 |
| OMG3/ST | 0.91  (0.78,1.06) | 3 | N/A | N/A | 0.94  (0.82,1.08) | 4 | 0.86  (0.47,1.58) | 4 |
| EZT/ST | 0.92  (0.80,1.05) | 4 | N/A | N/A | 0.93  (0.85,1.01) | 3 | 0.46  (0.15,1.40) | 2 |
| ST (reference) | 1.00 | 5 | N/A | N/A | 1.00 | 6 | 1.00 | 6 |
| CETP/ST | 1.02  (0.93,1.12) | 6 | N/A | N/A | 0.96  (0.89,1.04) | 5 | 0.97  (0.60,1.57) | 5 |
| Global inconsistency chi^2^ (P value) | 4.57  (P=0.3347) |  | N/A |  | 15.38  (P=0.0001) |  | 0.8  (P=0.6701) |  |
| Number of studies | 36 |  | 0 |  | 18 |  | 18 |  |

^a^Not classified in primary or secondary prevention

^b^Significant results are in bold

12.2 Subgroup analyses of intensity of statin with treatment options

eTable 12.2.1 The risk of cardiovascular mortality

The following table shows the effect sizes (risk ratio) and the rank order (SUCRA ranks) compared stain monotherapy before (standard analysis) and after subgroup analyses.

| Treatment | Standard analysis | SUCRA rank | Moderate/high^a^ | SUCRA rank | Moderate^a^ | SUCRA rank | Low/moderate^a^ | SUCRA rank |
| --- | --- | --- | --- | --- | --- | --- | --- | --- |
| FBT/ST | 0.86  (0.66,1.11) | 1 | 0.20  (0.01,4.17) | 1 | 0.86  (0.66,1.13) | 3 | N/A | N/A |
| NIA+EZT/ST | 0.41  (0.01,20.53) | 2 | N/A | N/A | 0.40  (0.01,20.29) | 2 | N/A | N/A |
| CETP/ST | 0.93  (0.84,1.03) | 3 | 0.91  (0.82,1.01) | 2 | 0.99  (0.02,50.00) | 4 | N/A | N/A |
| PCSK/ST | 0.96  (0.86,1.08) | 4 | 0.97  (0.86,1.08) | 3 | N/A | N/A | N/A | N/A |
| OMG3/ST | 0.95  (0.71,1.28) | 5 | N/A | N/A | 0.33  (0.07,1.67) | 1 | N/A | N/A |
| ST (reference) | 1.00 | 6 | 1.00 | 4 | 1.00 | 5 | N/A | N/A |
| EZT/ST | 1.01  (0.90,1.13) | 7 | 3.52  (1.07,11.65) | 6 | 1.00  (0.89,1.12) | 6 | N/A | N/A |
| NIA/ST | 1.09  (0.96,1.23) | 8 | 1.14  (0.75,1.74) | 5 | 1.09  (0.95,1.24) | 7 | N/A | N/A |
| Global inconsistency chi^2^ (P value) | 4.28  (P=0.2326) |  | 0.49  (P=0.9212) |  | 1.79  (P=0.1813) |  | N/A |  |
| Number of studies | 44 |  | 20 |  | 16 |  | 2 |  |

^a^Adapted from 2013 ACC/AHA,^69^ High intensity: atorvastatin (≥40 mg), rosuvastatin (≥20 mg), simvastatin (≥80 mg); Moderate intensity: atorvastatin (10-20 mg), rosuvastatin (5-10 mg), simvastatin (20-40 mg), pravastatin (40-80 mg), lovastatin (≥40 mg), fluvastatin (80 mg), pitavastatin (2-4 mg); Low intensity: atorvastatin (<10 mg), rosuvastatin (<5 mg), simvastatin (<20 mg), pravastatin (<40 mg), lovastatin (<40 mg), fluvastatin (<80 mg) pitavastatin (<2 mg)

eTable 12.2.2 The risk of all-cause mortality

The following table shows the effect sizes (risk ratio) and the rank order (SUCRA ranks) compared stain monotherapy before (standard analysis) and after subgroup analyses.

| Treatment | Standard analysis | SUCRA rank | | Moderate/high^a^ | SUCRA rank | Moderate^a^ | | SUCRA rank | Low/moderate^a^ | SUCRA rank |
| --- | --- | --- | --- | --- | --- | --- | --- | --- | --- | --- |
| FBT/ST | 0.90  (0.68,1.18) | 1 | 0.25  (0.03,2.25) | | 1 | | 0.91  (0.64,1.31) | 2 | N/A | N/A |
| PCSK/ST | 0.93  (0.79,1.08) | 2 | 0.93  (0.81,1.08) | | 3 | | N/A | N/A | N/A | N/A |
| EZT/ST | 0.97  (0.80,1.17) | 3 | 7.23  (1.51,34.51) | | 6 | | 0.93  (0.72,1.21) | 3 | 0.52  (0.04,6.07) | 1 |
| NIA+EZT/ST | 0.92  (0.26,3.29) | 4 | N/A | | N/A | | 0.92  (0.25,3.32) | 4 | N/A | N/A |
| ST (reference) | 1.00 | 5 | 1.00 | | 4 | | 1.00 | 5 | 1.00 | 2 |
| CETP/ST | 1.00  (0.87,1.15) | 6 | 0.92  (0.79,1.07) | | 2 | | 1.29  (0.47,3.59) | 7 | N/A | N/A |
| NIA/ST | 1.08  (0.89,1.30) | 7 | 1.15  (0.82,1.61) | | 5 | | 1.05  (0.74,1.50) | 6 | N/A | N/A |
| OMG3/ST | 1.08  (0.92,1.28) | 8 | N/A | | N/A | | 0.41  (0.12,1.35) | 1 | 1.12  (0.95,1.32) | 3 |
| Global inconsistency chi^2^ (P value) | 6.61  (P=0.1579) |  | 0.1  (P=0.9502) | |  | | 0.22  (P=0.6375) |  | 1.68  (P=0.1951) |  |
| Number of studies | 50 |  | | 20 |  | 18 | |  | 4 |  |

^a^Adapted from 2013 ACC/AHA,^69^ High intensity: atorvastatin (≥40 mg), rosuvastatin (≥20 mg), simvastatin (≥80 mg); Moderate intensity: atorvastatin (10-20 mg), rosuvastatin (5-10 mg), simvastatin (20-40 mg), pravastatin (40-80 mg), lovastatin (≥40 mg), fluvastatin (80 mg), pitavastatin (2-4 mg); Low intensity: atorvastatin (<10 mg), rosuvastatin (<5 mg), simvastatin (<20 mg), pravastatin (<40 mg), lovastatin (<40 mg), fluvastatin (<80 mg) pitavastatin (<2 mg)

eTable 12.2.3 The risk of coronary heart disease mortality

The following table shows the effect sizes (risk ratio) and the rank order (SUCRA ranks) compared stain monotherapy before (standard analysis) and after subgroup analyses.

| Treatment | Standard analysis | SUCRA rank | | Moderate/high^a^ | SUCRA rank | Moderate^a^ | | SUCRA rank | Low/moderate^a^ | SUCRA rank | |
| --- | --- | --- | --- | --- | --- | --- | --- | --- | --- | --- | --- |
| FBT/ST | 0.34  (0.01,8.20) | 1 | 0.34  (0.01,8.51) | | 1 | | N/A | N/A | N/A | N/A |  |
| NIA+EZT/ST | 0.39  (0.01,19.54) | 2 | N/A | | N/A | | 0.39  (0.01,19.43) | 1 | N/A | N/A |  |
| PCSK/ST | 0.89  (0.74,1.07) | 3 | 0.80  (0.42,1.53) | | 2 | | N/A | N/A | N/A | N/A |  |
| CETP/ST | 0.94  (0.84,1.06) | 4 | 0.91  (0.55,1.50) | | 3 | | 1.00  (0.10,9.64) | 3 | N/A | N/A |  |
| OMG3/ST | 0.96  (0.73,1.26) | 5 | N/A | | N/A | | 1.45  (0.21,9.76) | 6 | 0.93  (0.57,1.54) | 1 |  |
| EZT/ST | 0.96  (0.85,1.09) | 6 | 1.81  (0.40,8.23) | | 6 | | 0.96  (0.84,1.08) | 2 | 1.02  (0.02,50.81) | 2 |  |
| ST (reference) | 1.00 | 7 | 1.00 | | 4 | | 1.00 | 4 | 1.00 | 3 |  |
| NIA/ST | 1.04  (0.90,1.21) | 8 | 1.10  (0.57,2.15) | | 5 | | 1.04  (0.88,1.21) | 5 | N/A | N/A |  |
| Global inconsistency chi^2^ (P value) | 1.06  (P=0.787) |  | 0.13  (P=0.939) | |  | | 0.14  (P=0.7035) |  | 0.07  (P=0.7923) |  |  |
| Number of studies | 43 |  | | 16 |  | 17 | |  | 3 |  | |

^a^Adapted from 2013 ACC/AHA,^69^ High intensity: atorvastatin (≥40 mg), rosuvastatin (≥20 mg), simvastatin (≥80 mg); Moderate intensity: atorvastatin (10-20 mg), rosuvastatin (5-10 mg), simvastatin (20-40 mg), pravastatin (40-80 mg), lovastatin (≥40 mg), fluvastatin (80 mg), pitavastatin (2-4 mg); Low intensity: atorvastatin (<10 mg), rosuvastatin (<5 mg), simvastatin (<20 mg), pravastatin (<40 mg), lovastatin (<40 mg), fluvastatin (<80 mg) pitavastatin (<2 mg)

eTable 12.2.4 The risk of non-fatal myocardial infarction

The following table shows the effect sizes (risk ratio) and the rank order (SUCRA ranks) compared stain monotherapy before (standard analysis) and after subgroup analyses.

| Treatment | Standard analysis | SUCRA rank | | Moderate/high^a^ | SUCRA rank | Moderate^a^ | | SUCRA rank | Low/moderate^a^ | | SUCRA rank | |
| --- | --- | --- | --- | --- | --- | --- | --- | --- | --- | --- | --- | --- |
| PCSK/ST | **0.82**  **(0.72,0.93)^b^** | 1 | **0.82**  **(0.71,0.94)^b^** | | 2 | | N/A | N/A | N/A | N/A | |  |
| OMG3/ST | 0.80  (0.55,1.16) | 2 | N/A | | N/A | | 2.73  (0.52,14.41) | 6 | 0.75  (0.54,1.04) | 1 | |  |
| EZT/ST | 0.87  (0.71,1.07) | 3 | 0.60  (0.19,1.87) | | 1 | | **0.88**  **(0.81,0.95)^b^** | 1 | 1.02  (0.06,16.07) | 2 | |  |
| FBT/ST | 0.94  (0.71,1.25) | 4 | 3.02  (0.31,29.15) | | 6 | | 0.93  (0.76,1.13) | 3 | N/A | N/A | |  |
| CETP/ST | 0.97  (0.84,1.12) | 5 | 0.87  (0.68,1.10) | | 3 | | 0.82  (0.40,1.69) | 2 | N/A | N/A | |  |
| NIA/ST | 0.99  (0.81,1.22) | 6 | 1.14  (0.79,1.63) | | 5 | | 0.93  (0.82,1.07) | 4 | N/A | N/A | |  |
| ST (reference) | 1.00 | 7 | 1.00 | | 4 | | 1.00 | 5 | 1.00 | 3 | |  |
| Global inconsistency chi^2^ (P value) | 2.2  (P=0.5314) |  | 1.76  (P=0.4147) | |  | | 1.4  (P=0.2367) |  | 3.05  (P=0.0809) |  | |  |
| Number of studies | 37 |  | 15 | |  | | 13 |  | 3 |  | |  |

^a^Adapted from 2013 ACC/AHA,^69^ High intensity: atorvastatin (≥40 mg), rosuvastatin (≥20 mg), simvastatin (≥80 mg); Moderate intensity: atorvastatin (10-20 mg), rosuvastatin (5-10 mg), simvastatin (20-40 mg), pravastatin (40-80 mg), lovastatin (≥40 mg), fluvastatin (80 mg), pitavastatin (2-4 mg); Low intensity: atorvastatin (<10 mg), rosuvastatin (<5 mg), simvastatin (<20 mg), pravastatin (<40 mg), lovastatin (<40 mg), fluvastatin (<80 mg) pitavastatin (<2 mg)

**^b^**Significant results are in bold

eTable 12.2.5 The risk of any stroke

The following table shows the effect sizes (risk ratio) and the rank order (SUCRA ranks) compared stain monotherapy before (standard analysis) and after subgroup analyses.

| Treatment | Standard analysis | SUCRA rank | | Moderate/high^a^ | SUCRA rank | Moderate^a^ | | SUCRA rank | Low/moderate^a^ | | SUCRA rank | |
| --- | --- | --- | --- | --- | --- | --- | --- | --- | --- | --- | --- | --- |
| NIA+EZT/ST | 0.12  (0.005,2.90) | 1 | N/A | | N/A | | 0.12  (0.00,2.90) | 1 | N/A | N/A | |  |
| PCSK/ST | **0.74**  **(0.65,0.85)^b^** | 2 | **0.74**  **(0.65,0.85)^b^** | | 2 | | N/A | N/A | N/A | N/A | |  |
| EZT/ST | 0.88  (0.76,1.02) | 3 | 0.83  (0.11,6.56) | | 3 | | 0.88  (0.76,1.02) | 4 | N/A | N/A | |  |
| CETP/ST | 1.00  (0.91,1.10) | 4 | 0.98  (0.88,1.09) | | 4 | | 0.40  (0.10,1.62) | 3 | N/A | N/A | |  |
| ST (reference) | 1.00 | 5 | 1.00 | | 5 | | 1.00 | 6 | 1.00 | 1 | |  |
| OMG3/ST | 1.01  (0.81,1.25) | 6 | N/A | | N/A | | 0.31  (0.05,2.06) | 2 | 1.02  (0.13,8.21) | 2 | |  |
| FBT/ST | 1.03  (0.70,1.52) | 7 | 0.20  (0.01,4.17) | | 1 | | 1.06  (0.72,1.56) | 7 | N/A | N/A | |  |
| NIA/ST | 1.01  (0.90,1.14) | 8 | 1.78  (0.95,3.33) | | 6 | | 0.99  (0.88,1.12) | 5 | N/A | N/A | |  |
| Global inconsistency chi^2^ (P value) | 0.63  (P=0.8904) |  | 0.62  (P=0.7321) | |  | | 1.46  (P=0.2276) |  | 0.00  (P=0.9822) |  | |  |
| Number of studies | 41 |  | 17 | |  | | 18 |  | 1 |  | |  |

^a^Adapted from 2013 ACC/AHA,^69^ High intensity: atorvastatin (≥40 mg), rosuvastatin (≥20 mg), simvastatin (≥80 mg); Moderate intensity: atorvastatin (10-20 mg), rosuvastatin (5-10 mg), simvastatin (20-40 mg), pravastatin (40-80 mg), lovastatin (≥40 mg), fluvastatin (80 mg), pitavastatin (2-4 mg); Low intensity: atorvastatin (<10 mg), rosuvastatin (<5 mg), simvastatin (<20 mg), pravastatin (<40 mg), lovastatin (<40 mg), fluvastatin (<80 mg) pitavastatin (<2 mg)

^b^Significant results are in bold

eTable 12.2.6 The risk of coronary revascularization

The following table shows the effect sizes (risk ratio) and the rank order (SUCRA ranks) compared stain monotherapy before (standard analysis) and after subgroup analyses.

| Treatment | Standard analysis | SUCRA rank | Moderate/high^a^ | SUCRA rank | | Moderate^a^ | SUCRA rank | Low/moderate^a^ | | SUCRA rank |
| --- | --- | --- | --- | --- | --- | --- | --- | --- | --- | --- |
| PCSK/ST | **0.84**  **(0.75,0.94)^b^** | 1 | **0.83**  **(0.76,0.91)^b^** | | 3 | N/A | N/A | | N/A | N/A |
| NIA/ST | 0.88  (0.72,1.08) | 2 | 0.06  (0.003,1.35) | | 1 | **0.89**  **(0.80,0.99)^b^** | 1 | | N/A | N/A |
| OMG3/ST | 0.91  (0.78,1.06) | 3 | N/A | | N/A | 0.89  (0.57,1.40) | 2 | | 0.86  (0.71,1.04) | 1 |
| EZT/ST | 0.92  (0.80,1.05) | 4 | 0.44  (0.17,1.19) | | 2 | **0.93**  **(0.88,0.99)^b^** | 3 | | 0.97  (0.57,1.65) | 2 |
| ST (reference) | 1.00 | 5 | 1.00 | | 5 | 1.00 | 4 | | 1.00 | 3 |
| CETP/ST | 1.02  (0.93,1.12) | 6 | 0.94  (0.85,1.04) | | 4 | 1.21  (0.95,1.55) | 5 | | N/A | N/A |
| Global inconsistency chi^2^ (P value) | 4.57  (P=0.3347) |  | 0.84  (P=0.6578) | |  | 0.26  (P=0.6087) |  | | 2.57  ((P=0.1089) |  |
| Number of studies | 36 |  | 17 | |  | 11 |  | | 3 |  |

^a^Adapted from ACC/AHA 2013,^69^ High intensity: atorvastatin (≥40 mg), rosuvastatin (≥20 mg), simvastatin (≥80 mg); Moderate intensity: atorvastatin (10-20 mg), rosuvastatin (5-10 mg), simvastatin (20-40 mg), pravastatin (40-80 mg), lovastatin (≥40 mg), fluvastatin (80 mg), pitavastatin (2-4 mg); Low intensity: atorvastatin (<10 mg), rosuvastatin (<5 mg), simvastatin (<20 mg), pravastatin (<40 mg), lovastatin (<40 mg), fluvastatin (<80 mg) pitavastatin (<2 mg)

**^b^**Significant results in bold

12.3 Subgroup analyses of requirement of statin prior to starting non-statin lipid-lowering agent(s) with treatment options

eTable 12.3.1 The risk of cardiovascular mortality

The following table shows the effect sizes (risk ratio) and the rank order (SUCRA ranks) compared stain monotherapy before (standard analysis) and after subgroup analyses.

| Treatment | Standard analysis | SUCRA rank | Optimal LDL-C target /Maximally tolerated dose | SUCRA rank | No optimal LDL-C target/maximally tolerated dose | SUCRA rank |
| --- | --- | --- | --- | --- | --- | --- |
| FBT/ST | 0.86  (0.66,1.11) | 1 | 0.20  (0.01,4.18) | 1 | 0.86  (0.66,1.13) | 2 |
| NIA+EZT/ST | 0.41  (0.01,20.53) | 2 | N/A | N/A | 0.41  (0.01,20.41) | 3 |
| CETP/ST | 0.93  (0.84,1.03) | 3 | 0.93  (0.83,1.03) | 4 | 1.64  (0.34,7.92) | 7 |
| PCSK/ST | 0.96  (0.86,1.08) | 4 | 0.97  (0.86,1.09) | 5 | 0.59  (0.17,2.02) | 1 |
| OMG3/ST | 0.95  (0.71,1.28) | 5 | 0.32  (0.01,7.61) | 2 | 0.96  (0.71,1.29) | 4 |
| ST (reference) | 1.00 | 6 | 1.00 | 6 | 1.00 | 5 |
| EZT/ST | 1.01  (0.90,1.13) | 7 | 3.34  (0.04,288.61) | 7 | 1.00  (0.90,1.13) | 6 |
| NIA/ST | 1.09  (0.96,1.23) | 8 | 0.63  (0.01,31.48) | 3 | 1.10  (0.97,1.24) | 8 |
| Global inconsistency chi^2^ (P value) | 4.28  (P=0.2326) |  | 0.3  (P=0.5852) |  | 1.33  (P=0.5148) |  |
| Number of studies | 44 |  | 19 |  | 25 |  |

eTable 12.3.2 The risk of all-cause mortality

The following table shows the effect sizes (risk ratio) and the rank order (SUCRA ranks) compared stain monotherapy before (standard analysis) and after subgroup analyses.

| Treatment | Standard analysis | SUCRA rank | | Optimal LDL-C target /Maximally tolerated dose | SUCRA rank | No optimal LDL-C target/maximally tolerated dose | | SUCRA rank |
| --- | --- | --- | --- | --- | --- | --- | --- | --- |
| FBT/ST | 0.90  (0.68,1.18) | 1 | 0.25  (0.03,2.28) | | 2 | | 0.91  (0.76,1.10) | 2 |
| PCSK/ST | 0.93  (0.79,1.08) | 2 | 0.93  (0.77,1.13) | | 4 | | 0.32  (0.10,1.03) | 1 |
| EZT/ST | 0.97  (0.80,1.17) | 3 | 1.56  (0.03,72.12) | | 7 | | 0.98  (0.91,1.05) | 3 |
| NIA+EZT/ST | 0.92  (0.26,3.29) | 4 | N/A | | N/A | | 0.93  (0.26,3.25) | 5 |
| ST (reference) | 1.00 | 5 | 1.00 | | 5 | | 1.00 | 4 |
| CETP/ST | 1.00  (0.87,1.15) | 6 | 1.01  (0.85,1.21) | | 6 | | 1.19  (0.54,2.60) | 6 |
| NIA/ST | 1.08  (0.89,1.30) | 7 | 0.21  (0.01,5.17) | | 1 | | 1.09  (1.00,1.20) | 8 |
| OMG3/ST | 1.08  (0.92,1.28) | 8 | 0.32  (0.01,7.71) | | 3 | | 1.09  (0.97,1.22) | 7 |
| Global inconsistency chi^2^ (P value) | 6.61  (P=0.1579) |  | 0.53  (P=0.4677) | |  | | 1.91  (P=0.5912) |  |
| Number of studies | 50 |  | | 22 |  | 28 | |  |

eTable 12.3.3 The risk of coronary heart disease mortality

The following table shows the effect sizes (risk ratio) and the rank order (SUCRA ranks) compared stain monotherapy before (standard analysis) and after subgroup analyses.

| Treatment | Standard analysis | SUCRA rank | | Optimal LDL-C target /Maximally tolerated dose | SUCRA rank | No optimal LDL-C target/maximally tolerated dose | | SUCRA rank |
| --- | --- | --- | --- | --- | --- | --- | --- | --- |
| FBT/ST | 0.34  (0.01,8.20) | 1 | 0.34  (0.01,8.20) | | 2 | | N/A | N/A |
| NIA+EZT/ST | 0.39  (0.01,19.54) | 2 | N/A | | N/A | | 0.39  (0.01,19.48) | 1 |
| PCSK/ST | 0.89  (0.74,1.07) | 3 | 0.90  (0.75,1.08) | | 3 | | 0.64  (0.07,6.28) | 3 |
| CETP/ST | 0.94  (0.84,1.06) | 4 | 0.94  (0.84,1.06) | | 5 | | 0.64  (0.07,6.09) | 2 |
| OMG3/ST | 0.96  (0.73,1.26) | 5 | 0.32  (0.01,7.61) | | 1 | | 0.96  (0.73,1.27) | 5 |
| EZT/ST | 0.96  (0.85,1.09) | 6 | 1.78  (0.25,12.69) | | 7 | | 0.96  (0.85,1.09) | 4 |
| ST (reference) | 1.00 | 7 | 1.00 | | 6 | | 1.00 | 6 |
| NIA/ST | 1.04  (0.90,1.21) | 8 | 0.63  (0.01,31.49) | | 4 | | 1.04  (0.90,1.21) | 7 |
| Global inconsistency chi^2^ (P value) | 1.06  (P=0.787) |  | 1.33  (P=0.248) | |  | | 0.6  (P=0.44) |  |
| Number of studies | 43 |  | | 20 |  | 23 | |  |

eTable 12.3.4 The risk of non-fatal myocardial infarction

The following table shows the effect sizes (risk ratio) and the rank order (SUCRA ranks) compared stain monotherapy before (standard analysis) and after subgroup analyses.

| Treatment | Standard analysis | SUCRA rank | | Optimal LDL-C target /Maximally tolerated dose | SUCRA rank | No optimal LDL-C target/maximally tolerated dose | | SUCRA rank |
| --- | --- | --- | --- | --- | --- | --- | --- | --- |
| PCSK/ST | **0.82**  **(0.72,0.93)^a^** | 1 | **0.81**  **(0.71,0.94)^a^** | | 2 | | 0.45  (0.06,3.51) | 1 |
| OMG3/ST | 0.80  (0.55,1.16) | 2 | N/A | | N/A | | 0.79  (0.56,1.11) | 3 |
| EZT/ST | 0.87  (0.71,1.07) | 3 | 0.40  (0.11,1.46) | | 1 | | 0.88  (0.76,1.02) | 4 |
| FBT/ST | 0.94  (0.71,1.25) | 4 | 3.02  (0.31,29.18) | | 5 | | 0.93  (0.74,1.16) | 5 |
| CETP/ST | 0.97  (0.84,1.12) | 5 | 0.98  (0.84,1.15) | | 3 | | 0.62  (0.24,1.60) | 2 |
| NIA/ST | 0.99  (0.81,1.22) | 6 | N/A | | N/A | | 0.98  (0.80,1.19) | 6 |
| ST (reference) | 1.00 | 7 | 1.00 | | 4 | | 1.00 | 7 |
| Global inconsistency chi^2^ (P value) | 2.2  (P=0.5314) |  | 8.12  (P=0.0044) | |  | | 0.58  (P=0.7472) |  |
| Number of studies | 37 |  | 16 | |  | | 21 |  |

^a^Significant results are in bold

eTable 12.3.5 The risk of any stroke

The following table shows the effect sizes (risk ratio) and the rank order (SUCRA ranks) compared stain monotherapy before (standard analysis) and after subgroup analyses.

| Treatment | Standard analysis | SUCRA rank | | Optimal LDL-C target /Maximally tolerated dose | SUCRA rank | No optimal LDL-C target/maximally tolerated dose | | SUCRA rank |
| --- | --- | --- | --- | --- | --- | --- | --- | --- |
| NIA+EZT/ST | 0.12  (0.005,2.90) | 1 | N/A | | N/A | | 0.12  (0.00,3.03) | 1 |
| PCSK/ST | **0.74**  **(0.65,0.85)^a^** | 2 | **0.75**  **(0.65,0.85)^a^** | | 3 | | 0.35  (0.07,1.68) | 2 |
| EZT/ST | 0.88  (0.76,1.02) | 3 | 1.49  (0.09,23.73) | | 6 | | 0.90  (0.68,1.20) | 4 |
| CETP/ST | 1.00  (0.91,1.10) | 4 | 1.00  (0.91,1.10) | | 5 | | 0.53  (0.06,4.77) | 3 |
| ST (reference) | 1.00 | 5 | 1.00 | | 4 | | 1.00 | 6 |
| OMG3/ST | 1.01  (0.81,1.25) | 6 | N/A | | N/A | | 0.99  (0.72,1.37) | 5 |
| FBT/ST | 1.03  (0.70,1.52) | 7 | 0.20  (0.01,4.17) | | 1 | | 1.06  (0.68,1.65) | 7 |
| NIA/ST | 1.01  (0.90,1.14) | 8 | 0.21  (0.01,5.10) | | 2 | | 1.06  (0.74,1.53) | 8 |
| Global inconsistency chi^2^ (P value) | 0.63  (P=0.8904) |  | 18.5  (P=0.000) | |  | | 0.39  (P=0.8209) |  |
| Number of studies | 41 |  | 20 | |  | | 21 |  |

^a^Significant results in bold

eTable 12.3.6 The risk of coronary revascularization

The following table shows the effect sizes (risk ratio) and the rank order (SUCRA ranks) compared stain monotherapy before (standard analysis) and after subgroup analyses.

| Treatment | Standard analysis | SUCRA rank | | Optimal LDL-C target /Maximally tolerated dose | SUCRA rank | No optimal LDL-C target/maximally tolerated dose | | SUCRA rank |
| --- | --- | --- | --- | --- | --- | --- | --- | --- |
| PCSK/ST | **0.84**  **(0.75,0.94)^a^** | 1 | **0.85**  **(0.75,0.96)^a^** | | 3 | | 0.55  (0.29,1.03) | 2 |
| NIA/ST | 0.88  (0.72,1.08) | 2 | 0.06  (0.00,1.34) | | 1 | | **0.89**  **(0.80,0.99)^a^** | 3 |
| OMG3/ST | 0.91  (0.78,1.06) | 3 | N/A | | N/A | | 0.93  (0.84,1.02) | 4 |
| EZT/ST | 0.92  (0.80,1.05) | 4 | 0.42  (0.14,1.28) | | 2 | | **0.93**  **(0.88,0.99)^a^** | 5 |
| ST (reference) | 1.00 | 5 | 1.00 | | 4 | | 1.00 | 6 |
| CETP/ST | 1.02  (0.93,1.12) | 6 | 1.04  (0.94,1.16) | | 5 | | **0.29**  **(0.13,0.62)^a^** | 1 |
| Global inconsistency chi^2^ (P value) | 4.57  (P=0.3347) |  | 6.78  (P=0.0092) | |  | | 2.02  0.3649 |  |
| Number of studies | 36 |  | 17 | |  | | 19 |  |

^a^Significant results in bold

12.4 Subgroup analyses of cardiovascular risk (CV) stratification with treatment options

eTable 12.4.1 The risk of cardiovascular mortality

The following table shows the effect sizes (risk ratio) and the rank order (SUCRA ranks) compared stain monotherapy before (standard analysis) and after subgroup analyses.

| Treatment | Standard analysis | SUCRA rank | | Non-high CV risk^a^ | | SUCRA rank | High CV risk^a^ | SUCRA rank | |
| --- | --- | --- | --- | --- | --- | --- | --- | --- | --- |
| FBT/ST | 0.86  (0.66,1.11) | 1 | N/A | | N/A | | 0.86  (0.66,1.11) | | 1 |
| NIA+EZT/ST | 0.41  (0.01,20.53) | 2 | 0.44  (0.00,111.11) | | 1 | | N/A | | N/A |
| CETP/ST | 0.93  (0.84,1.03) | 3 | 0.71  (0.04,11.31) | | 3 | | 0.93  (0.84,1.03) | | 2 |
| PCSK/ST | 0.96  (0.86,1.08) | 4 | 1.13  (0.39,3.24) | | 6 | | 0.96  (0.86,1.08) | | 4 |
| OMG3/ST | 0.95  (0.71,1.28) | 5 | 1.90  (0.04,94.65) | | 7 | | 0.95  (0.70,1.28) | | 3 |
| ST (reference) | 1.00 | 6 | 1.00 | | 4 | | 1.00 | | 5 |
| EZT/ST | 1.01  (0.90,1.13) | 7 | 1.10  (0.02,54.06) | | 5 | | 1.01  (0.90,1.13) | | 6 |
| NIA/ST | 1.09  (0.96,1.23) | 8 | 0.63  (0.01,31.49) | | 2 | | 1.09  (0.96,1.23) | | 7 |
| Global inconsistency chi^2^ (P value) | 4.28  (P=0.2326) |  | 0.05  (P=0.819) | |  | | 4.28  (P=0.2326) | |  |
| Number of studies | 44 |  | 9 | |  | | 35 | |  |

^a^Adapted from 2016 ESC/EAS guideline^70^

eTable 12.4.2 The risk of all-cause mortality

The following table shows the effect sizes (risk ratio) and the rank order (SUCRA ranks) compared stain monotherapy before (standard analysis) and after subgroup analyses.

| Treatment | Standard analysis | SUCRA rank | | Non-high CV risk^a^ | | SUCRA rank | High CV risk^a^ | SUCRA rank |
| --- | --- | --- | --- | --- | --- | --- | --- | --- |
| FBT/ST | 0.90  (0.68,1.18) | 1 | N/A | | N/A | | 0.89  (0.66,1.20) | 1 |
| PCSK/ST | 0.93  (0.79,1.08) | 2 | N/A | | N/A | | 0.93  (0.79,1.11) | 2 |
| EZT/ST | 0.97  (0.80,1.17) | 3 | N/A | | N/A | | 0.97  (0.79,1.20) | 3 |
| NIA+EZT/ST | 0.92  (0.26,3.29) | 4 | N/A | | N/A | | 1.02(0.27,3.93) | 4 |
| ST (reference) | 1.00 | 5 | N/A | | N/A | | 1.00 | 5 |
| CETP/ST | 1.00  (0.87,1.15) | 6 | N/A | | N/A | | 1.00  (0.86,1.17) | 6 |
| NIA/ST | 1.08  (0.89,1.30) | 7 | N/A | | N/A | | 1.06  (0.84,1.34) | 7 |
| OMG3/ST | 1.08  (0.92,1.28) | 8 | N/A | | N/A | | 1.08  (0.87,1.33) | 8 |
| Global inconsistency chi^2^ (P value) | 6.61  (P=0.1579) |  | N/A | |  | | 6.4  (P=0.0935) |  |
| Number of studies | 50 |  | 5 | |  | | 39 |  |

^a^Adapted from 2016 ESC/EAS guideline^70^

eTable 12.4.3 The risk of coronary heart disease mortality

The following table shows the effect sizes (risk ratio) and the rank order (SUCRA ranks) compared stain monotherapy before (standard analysis) and after subgroup analyses.

| Treatment | Standard analysis | SUCRA rank | Non-high CV risk^a^ | SUCRA rank | High CV risk^a^ | SUCRA rank |
| --- | --- | --- | --- | --- | --- | --- |
| FBT/ST | 0.34  (0.01,8.20) | 1 | N/A | N/A | 0.34  (0.01,8.20) | 1 |
| NIA+EZT/ST | 0.39  (0.01,19.54) | 2 | N/A | N/A | N/A | N/A |
| PCSK/ST | 0.89  (0.74,1.07) | 3 | N/A | N/A | 0.89  (0.74,1.07) | 2 |
| CETP/ST | 0.94  (0.84,1.06) | 4 | N/A | N/A | 0.94  (0.84,1.06) | 3 |
| OMG3/ST | 0.96  (0.73,1.26) | 5 | N/A | N/A | 0.96  (0.70,1.33) | 5 |
| EZT/ST | 0.96  (0.85,1.09) | 6 | N/A | N/A | 0.96  (0.85,1.09) | 4 |
| ST (reference) | 1.00 | 7 | N/A | N/A | 1.00 | 6 |
| NIA/ST | 1.04  (0.90,1.21) | 8 | N/A | N/A | 1.04  (0.90,1.21) | 7 |
| Global inconsistency chi^2^ (P value) | 1.06  (P=0.787) |  | N/A |  | 1.06  (P=0.7877) |  |
| Number of studies | 43 |  | 6 |  | 35 |  |

^a^Adapted from 2016 ESC/EAS guideline^70^

eTable 12.4.4 The risk of non-fatal myocardial infarction

The following table shows the effect sizes (risk ratio) and the rank order (SUCRA ranks) compared stain monotherapy before (standard analysis) and after subgroup analyses.

| Treatment | Standard analysis | SUCRA rank | | Non-high CV risk^a^ | SUCRA rank | High CV risk^a^ | SUCRA rank |
| --- | --- | --- | --- | --- | --- | --- | --- |
| PCSK/ST | **0.82**  **(0.72,0.93)^b^** | 1 | N/A | | N/A | **0.81**  **(0.70,0.93)^b^** | 1 |
| OMG3/ST | 0.80  (0.55,1.16) | 2 | N/A | | N/A | 2.39  (0.45,12.64) | 7 |
| EZT/ST | 0.87  (0.71,1.07) | 3 | N/A | | N/A | 0.87  (0.70,1.08) | 2 |
| FBT/ST | 0.94  (0.71,1.25) | 4 | N/A | | N/A | 0.94  (0.71,1.26) | 3 |
| CETP/ST | 0.97  (0.84,1.12) | 5 | N/A | | N/A | 0.98  (0.84,1.14) | 4 |
| NIA/ST | 0.99  (0.81,1.22) | 6 | N/A | | N/A | 0.99  (0.81,1.22) | 5 |
| ST (reference) | 1.00 | 7 | N/A | | N/A | 1.00 | 6 |
| Global inconsistency chi^2^ (P value) | 2.2  (P=0.5314) |  | N/A | |  | 2.25  (P=0.5218) |  |
| Number of studies | 37 |  | 4 | |  | 30 |  |

^a^Adapted from 2016 ESC/EAS guideline^70^

^b^Significant results are in bold

eTable 12.4.5 The risk of any stroke

The following table shows the effect sizes (risk ratio) and the rank order (SUCRA ranks) compared stain monotherapy before (standard analysis) and after subgroup analyses.

| Treatment | Standard analysis | SUCRA rank | Non-high CV risk^a^ | SUCRA rank | High CV risk^a^ | | SUCRA rank |  |
| --- | --- | --- | --- | --- | --- | --- | --- | --- |
| NIA+EZT/ST | 0.12  (0.005,2.90) | 1 | N/A | N/A | | N/A | N/A | |
| PCSK/ST | **0.74**  **(0.65,0.85)^b^** | 2 | N/A | N/A | | **0.75**  **(0.66,0.86)^b^** | 2 | |
| EZT/ST | 0.88  (0.76,1.02) | 3 | N/A | N/A | | 0.88  (0.76,1.02) | 3 | |
| CETP/ST | 1.00  (0.91,1.10) | 4 | N/A | N/A | | 1.00  (0.91,1.11) | 6 | |
| ST (reference) | 1.00 | 5 | N/A | N/A | | 1.00 |  | |
| OMG3/ST | 1.01  (0.81,1.25) | 6 | N/A | N/A | | 0.18  (0.02,1.56) | 1 | |
| FBT/ST | 1.03  (0.70,1.52) | 7 | N/A | N/A | | 1.03  (0.70,1.52) | 5 | |
| NIA/ST | 1.01  (0.90,1.14) | 8 | N/A |  | | 1.02  (0.90,1.15) | 7 | |
| Global inconsistency chi^2^ (P value) | 0.63  (P=0.8904) |  |  |  | | 0.64  (P=0.8882) |  | |
| Number of studies | 41 |  | 7 |  | | 31 |  | |

^a^Adapted from 2016 ESC/EAS guideline^70^

^b^Significant results in bold

eTable 12.4.6 The risk of coronary revascularization

The following table shows the effect sizes (risk ratio) and the rank order (SUCRA ranks) compared stain monotherapy before (standard analysis) and after subgroup analyses.

| Treatment | Standard analysis | SUCRA rank | | Non-high CV risk^a^ | SUCRA rank | High CV risk^a^ | SUCRA rank |
| --- | --- | --- | --- | --- | --- | --- | --- |
| PCSK/ST | **0.84**  **(0.75,0.94)^b^** | 1 | 0.67  (0.33,1.37) | | 1 | **0.87**  **(0.76,0.996)^b^** | 1 |
| NIA/ST | 0.88  (0.72,1.08) | 2 | N/A | | N/A | 0.88  (0.71,1.10) | 2 |
| OMG3/ST | 0.91  (0.78,1.06) | 3 | N/A | | N/A | 0.93  (0.77,1.14) | 4 |
| EZT/ST | 0.92  (0.80,1.05) | 4 | N/A | | N/A | 0.92  (0.79,1.06) | 3 |
| ST (reference) | 1.00 | 5 | 1.00 | | 2 | 1.00 | 6 |
| CETP/ST | 1.02  (0.93,1.12) | 6 | N/A | | N/A | 1.00  (0.90,1.12) | 5 |
| Global inconsistency chi^2^ (P value) | 4.57  (P=0.3347) |  | 1.21  (P=0.2711) | |  | 4.38  (P=0.3576) |  |
| Number of studies | 36 |  | 2 | |  | 31 |  |

^a^Adapted from 2016 ESC/EAS guideline^70^

^b^Significant results in bold

12.5 Subgroup analyses of average age group with treatment options

eTable 12.5.1 The risk of cardiovascular mortality

The following table shows the effect sizes (risk ratio) and the rank order (SUCRA ranks) compared stain monotherapy before (standard analysis) and after subgroup analyses.

| Treatment | Standard analysis | SUCRA rank | | Age ­< 65 years | SUCRA rank | Age > 65 years | | SUCRA rank |
| --- | --- | --- | --- | --- | --- | --- | --- | --- |
| FBT/ST | 0.86  (0.66,1.11) | 1 | 0.85  (0.65,1.13) | | 1 | | N/A | N/A |
| NIA+EZT/ST | 0.41  (0.01,20.53) | 2 | 0.41  (0.01,20.67) | | 3 | | N/A | N/A |
| CETP/ST | 0.93  (0.84,1.03) | 3 | 1.31  (0.87,1.99) | | 8 | | 0.91  (0.82,1.01) | 1 |
| PCSK/ST | 0.96  (0.86,1.08) | 4 | 0.96  (0.85,1.09) | | 4 | | N/A | N/A |
| OMG3/ST | 0.95  (0.71,1.28) | 5 | 0.50  (0.04,5.73) | | 2 | | 0.96  (0.71,1.30) | 2 |
| ST (reference) | 1.00 | 6 | 1.00 | | 5 | | 1.00 | 3 |
| EZT/ST | 1.01  (0.90,1.13) | 7 | 1.02  (0.87,1.19) | | 6 | | 1.22  (0.31,4.80) | 4 |
| NIA/ST | 1.09  (0.96,1.23) | 8 | 1.15  (0.75,1.77) | | 7 | | 1.08  (0.95,1.24) | 5 |
| Global inconsistency chi^2^ (P value) | 4.28  (P=0.2326) |  | 1.69  (P=0.4305) | |  | | 3.41  (P=0.0648) |  |
| Number of studies | 44 |  | 33 | |  | | 11 |  |

eTable 12.5.2 The risk of All-cause mortality

The following table shows the effect sizes (risk ratio) and the rank order (SUCRA ranks) compared stain monotherapy before (standard analysis) and after subgroup analyses.

| Treatment | Standard analysis | SUCRA rank | | Age ­< 65 years | SUCRA rank | Age > 65 years | | SUCRA rank |
| --- | --- | --- | --- | --- | --- | --- | --- | --- |
| FBT/ST | 0.90  (0.68,1.18) | 1 | 0.89  (0.64,1.23) | | 2 | | N/A | N/A |
| PCSK/ST | 0.93  (0.79,1.08) | 2 | 0.91  (0.75,1.10) | | 1 | | N/A | N/A |
| EZT/ST | 0.97  (0.80,1.17) | 3 | 1.05  (0.78,1.41) | | 5 | | 0.70  (0.47,1.06) | 1 |
| NIA+EZT/ST | 0.92  (0.26,3.29) | 4 | 0.93  (0.26,3.34) | | 4 | | 1.08  (0.90,1.31) | 5 |
| ST (reference) | 1.00 | 5 | 1.00 | | 3 | | 1.00 | 4 |
| CETP/ST | 1.00  (0.87,1.15) | 6 | 1.17  (0.92,1.49) | | 8 | | 0.92  (0.79,1.07) | 2 |
| NIA/ST | 1.08  (0.89,1.30) | 7 | 1.12  (0.76,1.65) | | 6 | | N/A | N/A |
| OMG3/ST | 1.08  (0.92,1.28) | 8 | 1.15  (0.90,1.48) | | 7 | | 0.99  (0.77,1.28) | 3 |
| Global inconsistency chi^2^ (P value) | 6.61  (P=0.1579) |  | 2.37  (P=0.499) | |  | | 5  (P=0.0253) |  |
| Number of studies | 50 |  | 39 | |  | | 11 |  |

eTable 12.5.3 The risk of coronary heart disease mortality

The following table shows the effect sizes (risk ratio) and the rank order (SUCRA ranks) compared stain monotherapy before (standard analysis) and after subgroup analyses.

| Treatment | Standard analysis | SUCRA rank | | Age ­< 65 years | SUCRA rank | Age > 65 years | | SUCRA rank |
| --- | --- | --- | --- | --- | --- | --- | --- | --- |
| FBT/ST | 0.34  (0.01,8.20) | 1 | 0.34  (0.01,8.20) | | 1 | | N/A | N/A |
| NIA+EZT/ST | 0.39  (0.01,19.54) | 2 | 0.39  (0.01,19.56) | | 2 | | N/A | N/A |
| PCSK/ST | 0.89  (0.74,1.07) | 3 | 0.89  (0.74,1.07) | | 3 | | N/A | N/A |
| CETP/ST | 0.94  (0.84,1.06) | 4 | 0.98  (0.79,1.22) | | 6 | | 0.92  (0.81,1.06) | 1 |
| OMG3/ST | 0.96  (0.73,1.26) | 5 | 0.91  (0.56,1.49) | | 4 | | 0.98  (0.71,1.35) | 3 |
| EZT/ST | 0.96  (0.85,1.09) | 6 | 0.96  (0.85,1.09) | | 5 | | 0.91  (0.34,2.45) | 2 |
| ST (reference) | 1.00 | 7 | 1.00 | | 7 | | 1.00 | 4 |
| NIA/ST | 1.04  (0.90,1.21) | 8 | 1.09  (0.69,1.70) | | 8 | | 1.04  (0.88,1.22) | 5 |
| Global inconsistency chi^2^ (P value) | 1.06  (P=0.787) |  | 1.06  (P=0.7876) | |  | | 0.02  (P=0.8934) |  |
| Number of studies | 43 |  | 33 | |  | | 10 |  |

eTable 12.5.4 The risk of non-fatal myocardial infarction

The following table shows the effect sizes (risk ratio) and the rank order (SUCRA ranks) compared stain monotherapy before (standard analysis) and after subgroup analyses.

| Treatment | Standard analysis | SUCRA rank | | Age ­< 65 years | SUCRA rank | Age > 65 years | | SUCRA rank |
| --- | --- | --- | --- | --- | --- | --- | --- | --- |
| PCSK/ST | **0.82**  **(0.72,0.93)^a^** | 1 | **0.82**  **(0.72,0.93)^a^** | | 2 | | N/A | N/A |
| OMG3/ST | 0.80  (0.55,1.16) | 2 | 0.75  (0.52,1.08) | | 1 | | 2.73  (0.52,14.41) | 5 |
| EZT/ST | 0.87  (0.71,1.07) | 3 | 0.86  (0.71,1.05) | | 3 | | 1.02  (0.48,2.17) | 3 |
| FBT/ST | 0.94  (0.71,1.25) | 4 | 0.94  (0.72,1.23) | | 4 | | N/A | N/A |
| CETP/ST | 0.97  (0.84,1.12) | 5 | 1.05  (0.88,1.24) | | 6 | | 0.87  (0.78,0.97) | 1 |
| NIA/ST | 0.99  (0.81,1.22) | 6 | 1.14  (0.81,1.60) | | 7 | | 0.93  (0.82,1.07) | 2 |
| ST (reference) | 1.00 | 7 | 1.00 | | 5 | | 1.00 | 4 |
| Global inconsistency chi^2^ (P value) | 2.2  (P=0.5314) |  | 2.19  (P=0.5345) | |  | | 1.4  (P=0.2367) |  |
| Number of studies | 37 |  | 27 | |  | | 10 |  |

^a^Significant results are in bold

eTable 12.5.5 The risk of any stroke

The following table shows the effect sizes (risk ratio) and the rank order (SUCRA ranks) compared stain monotherapy before (standard analysis) and after subgroup analyses.

| Treatment | Standard analysis | SUCRA rank | | Age ­< 65 years | SUCRA rank | Age > 65 years | | SUCRA rank |
| --- | --- | --- | --- | --- | --- | --- | --- | --- |
| NIA+EZT/ST | 0.12  (0.005,2.90) | 1 | 0.12  (0.00,2.86) | | 1 | | N/A | N/A |
| PCSK/ST | **0.74**  **(0.65,0.85)^a^** | 2 | **0.74**  **(0.65,0.85)^a^** | | 2 | | N/A | N/A |
| EZT/ST | 0.88  (0.76,1.02) | 3 | 0.87  (0.75,1.01) | | 3 | | 1.01  (0.62,1.65) | 4 |
| CETP/ST | 1.00  (0.91,1.10) | 4 | 1.13  (0.89,1.44) | | 7 | | 0.98  (0.88,1.09) | 2 |
| ST (reference) | 1.00 | 5 | 1.00 | | 4 | | 1.00 | 5 |
| OMG3/ST | 1.01  (0.81,1.25) | 6 | 1.02  (0.83,1.27) | | 6 | | 0.31  (0.05,2.06) | 1 |
| FBT/ST | 1.03  (0.70,1.52) | 7 | 1.03  (0.70,1.52) | | 5 | | N/A | N/A |
| NIA/ST | 1.01  (0.90,1.14) | 8 | 1.64  (0.89,3.04) | | 8 | | 1.00  (0.88,1.12) | 3 |
| Global inconsistency chi^2^ (P value) | 0.63  (P=0.8904) |  | 0.63  (P=0.8906) | |  | | 1.46  (P=0.2276) |  |
| Number of studies | 41 |  | 30 | |  | | 11 |  |

^a^Significant results in bold

eTable 12.5.6 The risk of coronary revascularization

The following table shows the effect sizes (risk ratio) and the rank order (SUCRA ranks) compared stain monotherapy before (standard analysis) and after subgroup analyses.

| Treatment | Standard analysis | SUCRA rank | | Age ­< 65 years | SUCRA rank | Age > 65 years | | SUCRA rank |
| --- | --- | --- | --- | --- | --- | --- | --- | --- |
| PCSK/ST | **0.84**  **(0.75,0.94)^a^** | 1 | **0.84**  **(0.75,0.94)^a^** | | 1 | | N/A | N/A |
| NIA/ST | 0.88  (0.72,1.08) | 2 | N/A | | N/A | | 0.88  (0.76,1.03) | 1 |
| OMG3/ST | 0.91  (0.78,1.06) | 3 | 0.91  (0.78,1.07) | | 2 | | 0.89  (0.56,1.40) | 3 |
| EZT/ST | 0.92  (0.80,1.05) | 4 | 0.92  (0.77,1.09) | | 3 | | 0.90  (0.75,1.08) | 2 |
| ST (reference) | 1.00 | 5 | 1.00 | | 4 | | 1.00 | 5 |
| CETP/ST | 1.02  (0.93,1.12) | 6 | 1.09  (0.96,1.24) | | 5 | | 0.94  (0.85,1.05) | 4 |
| Global inconsistency chi^2^ (P value) | 4.57  (P=0.3347) |  | 2.93  (P=0.4032) | |  | | 1.74  (P=0.187) |  |
| Number of studies | 36 |  | 25 | |  | | 11 |  |

^a^Significant results are in bold

12.6 Subgroup analyses of Familial hypercholesterolemia (FH) groups with treatment options

eTable 12.6.1 The risk of cardiovascular mortality

The following table shows the effect sizes (risk ratio) and the rank order (SUCRA ranks) compared stain monotherapy before (standard analysis) and after subgroup analyses.

| Treatment | Standard analysis | SUCRA rank | | FH population | SUCRA rank | Non-FH population | SUCRA rank | |
| --- | --- | --- | --- | --- | --- | --- | --- | --- |
| FBT/ST | 0.86  (0.66,1.11) | 1 | N/A | | N/A | 0.86  (0.66,1.11) | | 1 |
| NIA+EZT/ST | 0.41  (0.01,20.53) | 2 | N/A | | N/A | 0.41  (0.01,20.50) | | 2 |
| CETP/ST | 0.93  (0.84,1.03) | 3 | 0.39  (0.03,4.65) | | 1 | 0.93  (0.84,1.03) | | 3 |
| PCSK/ST | 0.96  (0.86,1.08) | 4 | 0.49  (0.01,24.35) | | 2 | 0.96  (0.86,1.08) | | 4 |
| OMG3/ST | 0.95  (0.71,1.28) | 5 | N/A | | N/A | 0.95  (0.71,1.28) | | 5 |
| ST (reference) | 1.00 | 6 | 1.00 | | 3 | 1.00 | | 6 |
| EZT/ST | 1.01  (0.90,1.13) | 7 | 2.03  (0.19,22.33) | | 4 | 1.01  (0.90,1.13) | | 7 |
| NIA/ST | 1.09  (0.96,1.23) | 8 | N/A | | N/A | 1.09  (0.96,1.23) | | 8 |
| Global inconsistency chi^2^ (P value) | 4.28  (P=0.2326) |  | 0.13  (P=0.7224) | |  | 4.29  (P=0.2316) | |  |
| Number of studies | 44 |  | 4 | |  | 40 | |  |

eTable 12.6.2 The risk of All-cause mortality

The following table shows the effect sizes (risk ratio) and the rank order (SUCRA ranks) compared stain monotherapy before (standard analysis) and after subgroup analyses.

| Treatment | Standard analysis | SUCRA rank | | FH population | SUCRA rank | Non-FH population | SUCRA rank | |
| --- | --- | --- | --- | --- | --- | --- | --- | --- |
| FBT/ST | 0.90  (0.68,1.18) | 1 | N/A | | N/A | 0.90  (0.68,1.18) | | 1 |
| PCSK/ST | 0.93  (0.79,1.08) | 2 | 0.49  (0.00,57.81) | | 2 | 0.93  (0.79,1.08) | | 2 |
| EZT/ST | 0.97  (0.80,1.17) | 3 | N/A | | N/A | 0.97  (0.80,1.17) | | 3 |
| NIA+EZT/ST | 0.92  (0.26,3.29) | 4 | N/A | | N/A | 0.92  (0.26,3.29) | | 4 |
| ST (reference) | 1.00 | 5 | 1.00 | | 3 | 1.00 | | 5 |
| CETP/ST | 1.00  (0.87,1.15) | 6 | 0.50  (0.03,8.60) | | 1 | 1.00  (0.87,1.16) | | 6 |
| NIA/ST | 1.08  (0.89,1.30) | 7 | N/A | | N/A | 1.08  (0.89,1.30) | | 7 |
| OMG3/ST | 1.08  (0.92,1.28) | 8 | N/A | | N/A | 1.08  (0.92,1.28) | | 8 |
| Global inconsistency chi^2^ (P value) | 6.61  (P=0.1579) |  | 0.08  (P=0.7712) | |  | 6.61  (P=0.1579) | |  |
| Number of studies | 50 |  | 2 | |  | 48 | |  |

eTable 12.6.3 The risk of coronary heart disease mortality

The following table shows the effect sizes (risk ratio) and the rank order (SUCRA ranks) compared stain monotherapy before (standard analysis) and after subgroup analyses.

| Treatment | Standard analysis | SUCRA rank | | FH population | SUCRA rank | Non-FH population | SUCRA rank | |
| --- | --- | --- | --- | --- | --- | --- | --- | --- |
| FBT/ST | 0.34  (0.01,8.20) | 1 | N/A | | N/A | 0.34  (0.01,8.20) | | 1 |
| NIA+EZT/ST | 0.39  (0.01,19.54) | 2 | N/A | | N/A | 0.39  (0.01,19.54) | | 2 |
| PCSK/ST | 0.89  (0.74,1.07) | 3 | 1.71  (0.16,18.09) | | 3 | 0.89  (0.74,1.07) | | 3 |
| CETP/ST | 0.94  (0.84,1.06) | 4 | 0.50  (0.01,25.14) | | 1 | 0.94  (0.84,1.06) | | 4 |
| OMG3/ST | 0.96  (0.73,1.26) | 5 | N/A | | N/A | 0.96  (0.73,1.26) | | 5 |
| EZT/ST | 0.96  (0.85,1.09) | 6 | N/A | | N/A | 0.96  (0.85,1.09) | | 6 |
| ST (reference) | 1.00 | 7 | 1.00 | | 2 | 1.00 | | 7 |
| NIA/ST | 1.04  (0.90,1.21) | 8 | N/A | | N/A | 1.04  (0.90,1.21) | | 8 |
| Global inconsistency chi^2^ (P value) | 1.06  (P=0.787) |  | 0.2  (P=0.6547) | |  | 1.05  (P=0.7887) | |  |
| Number of studies | 43 |  | 3 | |  | 40 | |  |

eTable 12.6.4 The risk of non-fatal myocardial infarction

The following table shows the effect sizes (risk ratio) and the rank order (SUCRA ranks) compared stain monotherapy before (standard analysis) and after subgroup analyses.

| Treatment | Standard analysis | SUCRA rank | | FH population | | SUCRA rank | | Non-FH population | SUCRA rank |
| --- | --- | --- | --- | --- | --- | --- | --- | --- | --- |
| PCSK/ST | **0.82**  **(0.72,0.93)^a^** | 1 | 0.93  (0.06,15.82) | | 1 | | **0.82**  **(0.72,0.94)^a^** | | 1 |
| OMG3/ST | 0.80  (0.55,1.16) | 2 | N/A | | N/A | | 0.80  (0.55,1.16) | | 2 |
| EZT/ST | 0.87  (0.71,1.07) | 3 | N/A | | N/A | | 0.87  (0.71,1.07) | | 3 |
| FBT/ST | 0.94  (0.71,1.25) | 4 | N/A | | N/A | | 0.94  (0.71,1.25) | | 4 |
| CETP/ST | 0.97  (0.84,1.12) | 5 | N/A | | N/A | | 0.97  (0.84,1.12) | | 5 |
| NIA/ST | 0.99  (0.81,1.22) | 6 | N/A | | N/A | | 0.99  (0.81,1.22) | | 6 |
| ST (reference) | 1.00 | 7 | 1.00 | | 2 | | 1.00 | | 7 |
| Global inconsistency chi^2^ (P value) | 2.2  (P=0.5314) |  | 0.0  (P=0.9628) | |  | | 2.2  (P=0.5315) | |  |
| Number of studies | 37 |  | 2 | |  | | 35 | |  |

^a^Significant results are in bold

eTable 12.6.5 The risk of any stroke

The following table shows the effect sizes (risk ratio) and the rank order (SUCRA ranks) compared stain monotherapy before (standard analysis) and after subgroup analyses.

| Treatment | Standard analysis | SUCRA rank | | FH population | SUCRA rank | Non-FH population | SUCRA rank | |
| --- | --- | --- | --- | --- | --- | --- | --- | --- |
| NIA+EZT/ST | 0.12  (0.005,2.90) | 1 | N/A | | N/A | 0.12  (0.005,2.90) | | 1 |
| PCSK/ST | **0.74**  **(0.65,0.85)^a^** | 2 | N/A | | N/A | **0.74**  **(0.65,0.85)^a^** | | 2 |
| EZT/ST | 0.88  (0.76,1.02) | 3 | N/A | | N/A | 0.88  (0.76,1.02) | | 3 |
| CETP/ST | 1.00  (0.91,1.10) | 4 | N/A | | N/A | 1.00  (0.91,1.10) | | 4 |
| ST (reference) | 1.00 | 5 | N/A | | N/A | 1.00 | | 5 |
| OMG3/ST | 1.01  (0.81,1.25) | 6 | N/A | | N/A | 1.01  (0.81,1.25) | | 6 |
| FBT/ST | 1.03  (0.70,1.52) | 7 | N/A | | N/A | 1.03  (0.70,1.52) | | 7 |
| NIA/ST | 1.01  (0.90,1.14) | 8 | N/A | | N/A | 1.01  (0.90,1.14) | | 8 |
| Global inconsistency chi^2^ (P value) | 0.63  (P=0.8904) |  | N/A | |  | 0.63  (P=0.8906) | |  |
| Number of studies | 41 |  | 2 | |  | 39 | |  |

^a^Significant results in bold

eTable 12.6.6 The risk of coronary revascularization

The following table shows the effect sizes (risk ratio) and the rank order (SUCRA ranks) compared stain monotherapy before (standard analysis) and after subgroup analyses.

| Treatment | Standard analysis | SUCRA rank | | FH population | | SUCRA rank | | Non-FH population | SUCRA rank |
| --- | --- | --- | --- | --- | --- | --- | --- | --- | --- |
| PCSK/ST | **0.84**  **(0.75,0.94)^a^** | 1 | 1.31 (0.15,11.59) | | 2 | | **0.84**  **(0.75,0.94)^a^** | | 1 |
| NIA/ST | 0.88  (0.72,1.08) | 2 | N/A | | N/A | | 0.88  (0.72,1.08) | | 2 |
| OMG3/ST | 0.91  (0.78,1.06) | 3 | N/A | | N/A | | 0.91  (0.78,1.06) | | 3 |
| EZT/ST | 0.92  (0.80,1.05) | 4 | N/A | | N/A | | 0.92  (0.80,1.05) | | 4 |
| ST (reference) | 1.00 | 5 | 1.00 | | 1 | | 1.00 | | 5 |
| CETP/ST | 1.02  (0.93,1.12) | 6 |  | |  | | 1.02  (0.93,1.12) | | 6 |
| Global inconsistency chi^2^ (P value) | 4.57  (P=0.3347) |  | 0.06  (P=0.8083) | |  | | 4.58  (P=0.3335) | |  |
| Number of studies | 36 |  | 2 | |  | | 34 | |  |

^a^Significant results in bold

12.7 Subgroup analyses of baseline lipid level (LDL-C and Non-HDL-C level)

eTable 12.7.1 The risk of cardiovascular mortality

The following table shows the effect sizes (risk ratio) and the rank order (SUCRA ranks) compared stain monotherapy before (standard analysis) and after subgroup analyses.

| Treatment | Standard analysis | SUCRA rank | LDL-C < 100 mg/dl | SUCRA rank | LDL-C ≥ 100 mg/dl | SUCRA rank | Non-HDL-C  < 130 mg/dl | SUCRA rank | Non-HDL-C  ≥ 130 mg/dl | SUCRA rank |
| --- | --- | --- | --- | --- | --- | --- | --- | --- | --- | --- |
| FBT/ST | 0.86  (0.66,1.11) | 1 | 0.20  (0.01,4.18) | 1 | 0.86  (0.66,1.13) | 4 | 0.20  (0.01,4.18) | 1 | 0.86  (0.66,1.13) | 5 |
| NIA+EZT/ST | 0.41  (0.01,20.53) | 2 | N/A | N/A | 0.63  (0.01,35.49) | 5 |  |  | 0.63  (0.01,35.37) | 4 |
| CETP/ST | 0.93  (0.84,1.03) | 3 | 0.93  (0.83,1.05) | 2 | 0.51  (0.08,3.23) | 1 | 0.93  (0.83,1.05) | 2 | 0.51  (0.08,3.23) | 1 |
| PCSK/ST | 0.96  (0.86,1.08) | 4 | 0.98  (0.86,1.11) | 3 | 0.83  (0.54,1.28) | 3 | 0.98  (0.86,1.11) | 3 | 0.81  (0.52,1.26) | 2 |
| OMG3/ST | 0.95  (0.71,1.28) | 5 | N/A | N/A | 0.95  (0.70,1.28) | 6 |  |  | 0.95  (0.70,1.28) | 6 |
| ST (reference) | 1.00 | 6 | 1.00 | 4 | 1.00 | 7 | 1.00 | 4 | 1.00 | 7 |
| EZT/ST | 1.01  (0.90,1.13) | 7 | 1.01  (0.88,1.15) | 5 | 1.56  (0.59,4.09) | 8 | 1.01  (0.88,1.15) | 5 | 1.55  (0.59,4.07) | 8 |
| NIA/ST | 1.09  (0.96,1.23) | 8 | 1.09  (0.95,1.25) | 6 | 0.59  (0.04,9.45) | 2 | 1.09  (0.95,1.25) | 6 | 0.59  (0.04,9.45) | 3 |
| Global inconsistency chi^2^ (P value) | 4.28  (P=0.2326) |  | 2.59  (P=0.1072) |  | 0.82  (P=0.6629) |  | 2.59  (P=0.1072) |  | 0.81  (P=0.6664) |  |
| Number of studies | 44 |  | 16 |  | 28 |  | 17 |  | 27 |  |

eTable 12.7.2 The risk of All-cause mortality

The following table shows the effect sizes (risk ratio) and the rank order (SUCRA ranks) compared stain monotherapy before (standard analysis) and after subgroup analyses.

| Treatment | Standard analysis | SUCRA rank | LDL-C < 100 mg/dl | SUCRA rank | LDL-C ≥ 100 mg/dl | | SUCRA rank | | Non-HDL-C  < 130 mg/dl | | SUCRA rank | Non-HDL-C  ≥ 130 mg/dl | SUCRA rank |
| --- | --- | --- | --- | --- | --- | --- | --- | --- | --- | --- | --- | --- | --- |
| FBT/ST | 0.90  (0.68,1.18) | 1 | 0.25  (0.03,2.27) | 1 | 0.91  (0.52,1.62) | 5 | | 0.25  (0.03,2.27) | | 1 | | 0.91  (0.45,1.86) | 5 |
| PCSK/ST | 0.93  (0.79,1.08) | 2 | 0.98  (0.81,1.17) | 2 | 0.65  (0.34,1.24) | 2 | | 0.98  (0.81,1.18) | | 2 | | 0.60  (0.31,1.14) | 2 |
| EZT/ST | 0.97  (0.80,1.17) | 3 | 1.02  (0.78,1.34) | 6 | 0.96  (0.42,2.19) | 6 | | 1.02  (0.78,1.34) | | 6 | | 1.03  (0.47,2.24) | 7 |
| NIA+EZT/ST | 0.92  (0.26,3.29) | 4 | 1.02  (0.26,3.94) | 4 | 0.39  (0.01,22.03) | 3 | | 1.02  (0.26,3.94) | | 4 | | 0.41  (0.01,23.86) | 3 |
| ST (reference) | 1.00 | 5 | 1.00 | 3 | 1.00 | 8 | | 1.00 | | 3 | | 1.00 | 8 |
| CETP/ST | 1.00  (0.87,1.15) | 6 | 1.02  (0.86,1.20) | 5 | 0.71  (0.10,5.14) | 4 | | 1.02  (0.86,1.20) | | 5 | | 0.71  (0.10,5.21) | 4 |
| NIA/ST | 1.08  (0.89,1.30) | 7 | 1.08  (0.86,1.35) | 7 | 0.31  (0.03,3.81) | 1 | | 1.08  (0.86,1.35) | | 7 | | 0.31  (0.03,3.88) | 1 |
| OMG3/ST | 1.08  (0.92,1.28) | 8 | N/A | N/A | 0.98  (0.60,1.59) | 7 | | 1.95  (0.18,21.52) | | 8 | | 0.92  (0.52,1.62) | 6 |
| Global inconsistency chi^2^ (P value) | 6.61  (P=0.1579) |  | 3.81  (P=0.051) |  | 2.73  (P=0.2553) |  | | 3.81  (P=0.051) | |  | | 2.72  (0.2571) |  |
| Number of studies | 50 |  | 22 |  | 28 |  | | 24 | |  | | 26 |  |

eTable 12.7.3 The risk of coronary heart disease mortality

The following table shows the effect sizes (risk ratio) and the rank order (SUCRA ranks) compared stain monotherapy before (standard analysis) and after subgroup analyses.

| Treatment | Standard analysis | SUCRA rank | LDL-C < 100 mg/dl | SUCRA rank | LDL-C ≥ 100 mg/dl | SUCRA rank | Non-HDL-C  < 130 mg/dl | SUCRA rank | Non-HDL-C  ≥ 130 mg/dl | SUCRA rank |
| --- | --- | --- | --- | --- | --- | --- | --- | --- | --- | --- |
| FBT/ST | 0.34  (0.01,8.20) | 1 | 0.34  (0.01,8.20) | 1 | N/A | N/A | 0.34  (0.01,8.20) | 1 | N/A | N/A |
| NIA+EZT/ST | 0.39  (0.01,19.54) | 2 | N/A | N/A | 0.44  (0.01,25.28) | 2 | N/A | N/A | 0.43  (0.01,24.61) | 2 |
| PCSK/ST | 0.89  (0.74,1.07) | 3 | 0.92  (0.76,1.11) | 2 | 0.48  (0.21,1.14) | 1 | 0.93  (0.77,1.12) | 2 | 0.45  (0.19,1.05) | 1 |
| CETP/ST | 0.94  (0.84,1.06) | 4 | 0.94  (0.84,1.06) | 3 | 0.63  (0.07,6.06) | 4 | 0.94  (0.84,1.06) | 3 | 0.63  (0.07,6.06) | 4 |
| OMG3/ST | 0.96  (0.73,1.26) | 5 | N/A | N/A | 0.95  (0.73,1.25) | 5 | 4.88  (0.24,100.55) | 7 | 0.94  (0.72,1.24) | 5 |
| EZT/ST | 0.96  (0.85,1.09) | 6 | 0.96  (0.84,1.09) | 4 | 1.10  (0.40,3.01) | 7 | 0.96  (0.84,1.09) | 4 | 1.07  (0.39,2.92) | 7 |
| ST (reference) | 1.00 | 7 | 1.00 | 5 | 1.00 | 6 | 1.00 | 5 | 1.00 | 6 |
| NIA/ST | 1.04  (0.90,1.21) | 8 | 1.04  (0.90,1.21) | 6 | 0.59  (0.04,9.45) | 3 | 1.04  (0.90,1.21) | 6 | 0.59  (0.04,9.45) | 3 |
| Global inconsistency chi^2^ (P value) | 1.06  (P=0.787) |  | 0.74  (P=0.3898) |  | 0.62  (P=0.8918) |  | 0.66  (P=0.416) |  | 0.71  (P=0.8717) |  |
| Number of studies | 43 |  | 16 |  | 26 |  | 16 |  | 25 |  |

eTable 12.7.4 The risk of non-fatal myocardial infarction

The following table shows the effect sizes (risk ratio) and the rank order (SUCRA ranks) compared stain monotherapy before (standard analysis) and after subgroup analyses.

| Treatment | Standard analysis | SUCRA rank | LDL-C < 100 mg/dl | SUCRA rank | LDL-C ≥ 100 mg/dl | SUCRA rank | Non-HDL-C  < 130 mg/dl | SUCRA rank | Non-HDL-C  ≥ 130 mg/dl | SUCRA rank |
| --- | --- | --- | --- | --- | --- | --- | --- | --- | --- | --- |
| PCSK/ST | **0.82**  **(0.72,0.93)^a^** | 1 | **0.84**  **(0.71,0.99)^a^** | 1 | 0.80  (0.51,1.24) | 2 | 0.84  (0.71,1.00) | 1 | 0.79  (0.52,1.19) | 1 |
| OMG3/ST | 0.80  (0.55,1.16) | 2 | N/A | N/A | 0.89  (0.42,1.88) | 4 | 2.93  (0.30,28.12) | 6 | 0.80  (0.41,1.56) | 3 |
| EZT/ST | 0.87  (0.71,1.07) | 3 | 0.87  (0.67,1.13) | 2 | 0.85  (0.41,1.76) | 3 | 0.87  (0.67,1.13) | 2 | 0.84  (0.41,1.73) | 4 |
| FBT/ST | 0.94  (0.71,1.25) | 4 | 3.02  (0.31,29.26) | 6 | 0.93  (0.47,1.81) | 5 | 3.02  (0.31,29.26) | 7 | 0.93  (0.50,1.72) | 5 |
| CETP/ST | 0.97  (0.84,1.12) | 5 | 0.97  (0.83,1.15) | 3 | 0.51  (0.01,26.79) | 1 | 0.97  (0.83,1.15) | 3 | 0.51  (0.01,26.56) | 2 |
| NIA/ST | 0.99  (0.81,1.22) | 6 | 1.00  (0.80,1.26) | 4 | N/A | N/A | 1.00  (0.79,1.26) | 4 | N/A | N/A |
| ST (reference) | 1.00 | 7 | 1.00 | 5 | 1.00 | 6 | 1.00 | 5 | 1.00 | 6 |
| Global inconsistency chi^2^ (P value) | 2.2  (P=0.5314) |  | 3.94  (P=0.0473) |  | 2.09  (P=0.5534) |  | 3.8  (P=0.0513) |  | 2.18  (P=0.5364) |  |
| Number of studies | 37 |  | 16 |  | 21 |  | 16 |  | 21 |  |

^a^Significant results are in bold

eTable 12.7.5 The risk of any stroke

The following table shows the effect sizes (risk ratio) and the rank order (SUCRA ranks) compared stain monotherapy before (standard analysis) and after subgroup analyses.

| Treatment | Standard analysis | SUCRA rank | LDL-C < 100 mg/dl | SUCRA rank | LDL-C ≥ 100 mg/dl | SUCRA rank | Non-HDL-C  < 130 mg/dl | SUCRA rank | Non-HDL-C  ≥ 130 mg/dl | SUCRA rank |
| --- | --- | --- | --- | --- | --- | --- | --- | --- | --- | --- |
| NIA+EZT/ST | 0.12  (0.005,2.90) | 1 | N/A | N/A | 0.13  (0.01,3.39) | 1 | N/A | N/A | 0.13  (0.01,3.40) | 1 |
| PCSK/ST | **0.74**  **(0.65,0.85)^a^** | 2 | **0.75**  **(0.65,0.86)^a^** | 2 | 0.69  (0.46,1.03) | 3 | **0.75**  **(0.65,0.86)^a^** | 2 | 0.70  (0.47,1.05) | 3 |
| EZT/ST | 0.88  (0.76,1.02) | 3 | 0.87  (0.75,1.01) | 3 | 0.99  (0.60,1.64) | 4 | 0.86  (0.74,1.00) | 4 | 0.99  (0.60,1.65) | 4 |
| CETP/ST | 1.00  (0.91,1.10) | 4 | 1.00  (0.91,1.10) | 4 | 1.57  (0.27,9.03) | 8 | 1.00  (0.91,1.10) | 5 | 1.57  (0.27,9.03) | 8 |
| ST (reference) | 1.00 | 5 | 1.00 | 5 | 1.00 | 5 | 1.00 | 6 | 1.00 | 5 |
| OMG3/ST | 1.01  (0.81,1.25) | 6 | N/A | N/A | 1.01  (0.81,1.25) | 6 | 0.33  (0.01,7.90) | 3 | 1.01  (0.82,1.25) | 6 |
| FBT/ST | 1.03  (0.70,1.52) | 7 | 0.20  (0.01,4.17) | 1 | 1.06  (0.72,1.56) | 7 | 0.20  (0.01,4.17) | 1 | 1.06  (0.72,1.56) | 7 |
| NIA/ST | 1.01  (0.90,1.14) | 8 | 1.02  (0.90,1.15) | 6 | 0.21  (0.01,5.10) | 2 | 1.02  (0.90,1.15) | 7 | 0.21  (0.01,5.10) | 2 |
| Global inconsistency chi^2^ (P value) | 0.63  (P=0.8904) |  | 16.28  (P=0.0001) |  | 0.6  (P=0.8972) |  | 16.54  0.000 |  | 0.61  (P=0.8933) |  |
| Number of studies | 41 |  | 19 |  | 22 |  | 18 |  | 22 |  |

^a^Significant results in bold

eTable 12.7.6 The risk of coronary revascularization

The following table shows the effect sizes (risk ratio) and the rank order (SUCRA ranks) compared stain monotherapy before (standard analysis) and after subgroup analyses.

| Treatment | Standard analysis | SUCRA rank | LDL-C < 100 mg/dl | SUCRA rank | LDL-C ≥ 100 mg/dl | | SUCRA rank | | Non-HDL-C  < 130 mg/dl | | SUCRA rank | | Non-HDL-C  ≥ 130 mg/dl | | SUCRA rank | |
| --- | --- | --- | --- | --- | --- | --- | --- | --- | --- | --- | --- | --- | --- | --- | --- | --- |
| PCSK/ST | **0.84**  **(0.75,0.94)^a^** | 1 | **0.82 (0.71,0.96)^a^** | 1 | 0.91 (0.72,1.15) | 3 | | **0.82 (0.71,0.96)^a^** | | 1 | | 0.92 (0.73,1.15) | | 3 | |  |
| NIA/ST | 0.88  (0.72,1.08) | 2 | 0.88 (0.68,1.13) | 2 | N/A | N/A | | 0.88 (0.69,1.12) | | 2 | | N/A | | N/A | |  |
| OMG3/ST | 0.91  (0.78,1.06) | 3 | N/A | N/A | 0.86 (0.72,1.03) | 1 | | 1.07 (0.59,1.94) | | 5 | | 0.84 (0.70,1.01) | | 1 | |  |
| EZT/ST | 0.92  (0.80,1.05) | 4 | 0.98 (0.78,1.24) | 3 | **0.86 (0.75,0.99)^a^** | 2 | | 0.95 (0.76,1.20) | | 3 | | **0.86 (0.75,0.99)^a^** | | 2 | |  |
| ST (reference) | 1.00 | 5 | 1.00 | 4 | 1.00 | 4 | | 1.00 | | 4 | | 1.00 | | 4 | |  |
| CETP/ST | 1.02  (0.93,1.12) | 6 | 1.02 (0.91,1.15) | 5 | N/A | N/A | | 1.02 (0.91,1.15) | | 6 | | N/A | | N/A | |  |
| Global inconsistency chi^2^ (P value) | 4.57  (P=0.3347) |  | 1.59  (P=0.2076) |  | 2.2  (P=0.531) |  | | 1.64  (P=0.2008) | |  | | 2.17  (P=0.5378) | |  | |  |
| Number of studies | 36 |  | 16 |  | 20 |  | | 15 | |  | | 20 | |  | |  |

^a^Significant results in bold

12.8 Subgroup analyses of baseline lipid level (HDL-C and Triglyceride level)

eTable 12.8.1 the risk of cardiovascular mortality

The following table shows the effect sizes (risk ratio) and the rank order (SUCRA ranks) compared stain monotherapy before (standard analysis) and after subgroup analyses.

| Treatment | Standard analysis | SUCRA rank | HDL-C  < 40 mg/dl | SUCRA rank | HDL-C  > 40 mg/dl | SUCRA rank | TG  < 150 mg/dl | SUCRA rank | TG  ≥ 150 mg/dl | SUCRA rank | HDL-C  ≤ 40 mg/dl and TG  ≥ 150 mg/dl | SUCRA rank |
| --- | --- | --- | --- | --- | --- | --- | --- | --- | --- | --- | --- | --- |
| FBT/ST | 0.86  (0.66,1.11) | 1 | 0.86  (0.66,1.11) | 1 | N/A | N/A | N/A | N/A | 0.86  (0.66,1.11) | 4 | 0.86  (0.66,1.11) | 1 |
| NIA+EZT/ST | 0.41  (0.01,20.53) | 2 | N/A | N/A | 0.42  (0.01,21.24) | 1 | N/A | N/A | 0.42  (0.01,26.81) | 2 | N/A | N/A |
| CETP/ST | 0.93  (0.84,1.03) | 3 | 0.92  (0.82,1.04) | 2 | 0.98  (0.75,1.28) | 4 | 0.94  (0.81,1.08) | 1 | 0.72  (0.05,11.43) | 5 | N/A | N/A |
| PCSK/ST | 0.96  (0.86,1.08) | 4 | N/A | N/A | 0.96  (0.82,1.14) | 3 | 0.98  (0.85,1.12) | 3 | 0.82  (0.50,1.36) | 3 | N/A | N/A |
| OMG3/ST | 0.95  (0.71,1.28) | 5 | N/A | N/A | 0.93  (0.65,1.34) | 2 | 0.95  (0.69,1.31) | 2 | 0.50  (0.04,5.72) | 1 | N/A | N/A |
| ST (reference) | 1.00 | 6 | 1.00 | 3 | 1.00 | 5 | 1.00 | 4 | 1.00 | 7 | 1.00 | 2 |
| EZT/ST | 1.01  (0.90,1.13) | 7 | 4.95  (0.24,101.87) | 5 | 1.03  (0.81,1.32) | 6 | 1.02  (0.85,1.23) | 5 | 1.05  (0.27,4.12) | 6 | 4.95  (0.24,101.87) | 4 |
| NIA/ST | 1.09  (0.96,1.23) | 8 | 1.17  (0.76,1.79) | 4 | 1.06  (0.83,1.36) | 7 | 1.08  (0.90,1.28) | 6 | 1.16  (0.76,1.77) | 8 | 1.17  (0.76,1.79) | 3 |
| Global inconsistency chi^2^ (P value) | 4.28  (P=0.2326) |  | 0.52  (P=0.473) |  | 4.17  (P=0.2433) |  | 4.25  (P=0.2361) |  | 0.59  (P=0.4441) |  | 0.52  (P=0.473) |  |
| Number of studies | 44 |  | 5 |  | 38 |  | 29 |  | 15 |  | 4 |  |

eTable 12.8.2 The risk of all-cause mortality

The following table shows the effect sizes (risk ratio) and the rank order (SUCRA ranks) compared stain monotherapy before (standard analysis) and after subgroup analyses.

| Treatment | Standard analysis | SUCRA rank | | HDL-C  < 40 mg/dl | SUCRA rank | HDL-C  > 40 mg/dl | SUCRA rank | TG  < 150 mg/dl | SUCRA rank | TG  ≥ 150 mg/dl | SUCRA rank | HDL-C  ≤ 40 mg/dl and TG  ≥ 150 mg/dl | SUCRA rank |
| --- | --- | --- | --- | --- | --- | --- | --- | --- | --- | --- | --- | --- | --- |
| FBT/ST | 0.90  (0.68,1.18) | 1 | 0.91  (0.76,1.09) | | 1 | N/A | N/A | N/A | N/A | 0.91  (0.76,1.09) | 1 | 0.91  (0.76,1.09) | 1 |
| PCSK/ST | 0.93  (0.79,1.08) | 2 | 1.49  (0.06,36.45) | | 5 | 0.89  (0.71,1.12) | 1 | 0.90  (0.71,1.14) | 1 | 0.92  (0.64,1.33) | 2 | 1.49  (0.06,36.45) | 3 |
| EZT/ST | 0.97  (0.80,1.17) | 3 | 6.93  (0.36,132.51) | | 7 | 0.96  (0.73,1.28) | 3 | 0.97  (0.73,1.30) | 3 | 1.69  (0.20,14.65) | 8 | 6.93  (0.36,132.51) | 5 |
| NIA+EZT/ST | 0.92  (0.26,3.29) | 4 | 1.02  (0.27,3.85) | | 2 | 0.39  (0.01,20.04) | 2 | 1.02  (0.26,4.03) | 4 | 0.68  (0.01,59.75) | 3 | N/A | N/A |
| ST (reference) | 1.00 | 5 | 1.00 | | 4 | 1.00 | 5 | 1.00 | 5 | 1.00 | 4 | 1.00 | 2 |
| CETP/ST | 1.00  (0.87,1.15) | 6 | 0.97  (0.90,1.05) | | 2 | 1.05  (0.84,1.32) | 7 | 1.03  (0.84,1.26) | 7 | 1.21  (0.20,7.39) | 5 | N/A | N/A |
| NIA/ST | 1.08  (0.89,1.30) | 7 | 1.14  (0.86,1.52) | | 6 | 1.00  (0.69,1.46) | 4 | 1.02  (0.70,1.49) | 6 | 1.13  (0.85,1.49) | 6 | 1.14  (0.86,1.52) | 4 |
| OMG3/ST | 1.08  (0.92,1.28) | 8 | N/A | | N/A | 1.02  (0.77,1.34) | 6 | 0.95  (0.63,1.41) |  | 1.11  (0.94,1.31) | 7 | N/A | N/A |
| Global inconsistency chi^2^ (P value) | 6.61  (P=0.1579) |  | 0.06  (P=0.8052) | |  | 6.26  (P=0.0998) |  | 6.27  (P=0.0991) |  | 0.2  (P=0.6567) |  | 0.06  (P=0.8052) |  |
| Number of studies | 50 |  | | 8 |  | 41 |  | 34 |  | 16 |  | 6 |  |

eTable 12.8.3 The risk of coronary heart disease mortality

The following table shows the effect sizes (risk ratio) and the rank order (SUCRA ranks) compared stain monotherapy before (standard analysis) and after subgroup analyses.

| **Treatment** | **Standard analysis** | **SUCRA rank** | HDL-C  < 40 mg/dl | SUCRA rank | HDL-C  > 40 mg/dl | SUCRA rank | TG  < 150 mg/dl | SUCRA rank | TG  ≥ 150 mg/dl | SUCRA rank | HDL-C  ≤ 40 mg/dl and TG  ≥ 150 mg/dl | SUCRA rank |
| --- | --- | --- | --- | --- | --- | --- | --- | --- | --- | --- | --- | --- |
| FBT/ST | 0.34  (0.01,8.20) | 1 | 0.34  (0.01,8.20) | 1 | N/A | N/A | N/A | N/A | 0.34  (0.01,8.20) | 3 | N/A | N/A |
| NIA+EZT/ST | 0.39  (0.01,19.54) | 2 | N/A | N/A | 0.39  (0.01,19.54) | 2 | N/A | N/A | 0.17  (0.00,13.54) | 1 | N/A | N/A |
| PCSK/ST | 0.89  (0.74,1.07) | 3 | N/A | N/A | 0.89  (0.74,1.07) | 1 | 0.89  (0.74,1.07) | 1 | N/A | N/A | N/A | N/A |
| CETP/ST | 0.94  (0.84,1.06) | 4 | 0.92  (0.80,1.06) | 2 | 0.99  (0.80,1.23) | 5 | 0.94  (0.84,1.06) | 2 | 0.52  (0.06,4.18) | 4 | N/A | N/A |
| OMG3/ST | 0.96  (0.73,1.26) | 5 | N/A | N/A | 0.95  (0.73,1.25) | 3 | 0.97  (0.70,1.35) | 4 | 0.91  (0.56,1.49) | 5 | N/A | N/A |
| EZT/ST | 0.96  (0.85,1.09) | 6 | N/A | N/A | 0.96  (0.85,1.09) | 4 | 0.97  (0.85,1.09) | 3 | 0.41  (0.05,3.08) | 2 | N/A | N/A |
| ST (reference) | 1.00 | 7 | 1.00 | 3 | 1.00 | 6 | 1.00 | 5 | 1.00 | 6 | N/A | N/A |
| NIA/ST | 1.04  (0.90,1.21) | 8 | 1.10  (0.70,1.74) | 4 | 1.04  (0.88,1.21) | 7 | 1.04  (0.88,1.22) | 6 | 1.09  (0.69,1.73) | 7 | N/A | N/A |
| Global inconsistency chi^2^ (P value) | 1.06  (P=0.787) |  | 0.18  (P=0.6737) |  | 1.06  (P=0.787) |  | 1.05  (p=0.7892) |  | 0.14  (P=0.7115) |  | N/A |  |
| Number of studies | 43 |  | 4 |  | 38 |  | 29 |  | 13 |  | 3 |  |

eTable 12.8.4 The risk of non-fatal myocardial infarction

The following table shows the effect sizes (risk ratio) and the rank order (SUCRA ranks) compared stain monotherapy before (standard analysis) and after subgroup analyses.

| **Treatment** | **Standard analysis** | **SUCRA rank** | HDL-C  < 40 mg/dl | SUCRA rank | HDL-C  > 40 mg/dl | SUCRA rank | TG  < 150 mg/dl | SUCRA rank | TG  ≥ 150 mg/dl | SUCRA rank | HDL-C  ≤ 40 mg/dl and TG  ≥ 150 mg/dl | SUCRA rank |
| --- | --- | --- | --- | --- | --- | --- | --- | --- | --- | --- | --- | --- |
| PCSK/ST | **0.82**  **(0.72,0.93)^a^** | 1 | 0.96  (0.19,4.70) | 2 | **0.82**  **(0.73,0.92)^a^** | 1 | **0.84**  **(0.71,0.99)^a^** | 1 | 0.77  (0.59,1.01) | 2 | N/A | N/A |
| OMG3/ST | 0.80  (0.55,1.16) | 2 | N/A | N/A | 0.79  (0.55,1.13) | 2 | 2.96  (0.47,18.80) | 6 | 0.75  (0.54,1.04) | 1 | N/A | N/A |
| EZT/ST | 0.87  (0.71,1.07) | 3 | N/A | N/A | 0.87  (0.73,1.05) | 3 | 0.88  (0.68,1.14) | 2 | 0.66  (0.08,5.35) | 3 | N/A | N/A |
| FBT/ST | 0.94  (0.71,1.25) | 4 | N/A | N/A | 0.92  (0.02,44.89) | 4 | N/A | N/A | 0.93  (0.77,1.14) | 4 | 0.96  (0.19,4.70) | 2 |
| CETP/ST | 0.97  (0.84,1.12) | 5 | 0.87  (0.61,1.23) | 1 | 1.05  (0.88,1.24) | 7 | 0.97  (0.82,1.16) | 4 | N/A | N/A | N/A | N/A |
| NIA/ST | 0.99  (0.81,1.22) | 6 | 1.14  (0.73,1.77) | 4 | 0.93  (0.75,1.16) | 5 | 0.93  (0.69,1.26) | 3 | 1.14  (0.85,1.52) | 6 | 1.14  (0.73,1.77) | 3 |
| ST (reference) | 1.00 | 7 | 1.00 | 3 | 1.00 | 6 | 1.00 | 5 | 1.00 | 5 | 1.00 | 1 |
| Global inconsistency chi^2^ (P value) | 2.2  (P=0.5314) |  | 0.32  (P=0.5741) |  | 2.21  (P=0.53) |  | 2.11  (P=0.549) |  | 3.7  (P=0.0545) |  | 0.32  (P=0.5741) |  |
| Number of studies | 37 |  | 4 |  | 32 |  | 27 |  | 10 |  | 3 |  |

^a^Significant results are in bold

eTable 12.8.5 The risk of any stroke

The following table shows the effect sizes (risk ratio) and the rank order (SUCRA ranks) compared stain monotherapy before (standard analysis) and after subgroup analyses.

| **Treatment** | **Standard analysis** | **SUCRA rank** | HDL-C  < 40 mg/dl | SUCRA rank | HDL-C  > 40 mg/dl | SUCRA rank | TG  < 150 mg/dl | SUCRA rank | TG  ≥ 150 mg/dl | SUCRA rank | HDL-C  ≤ 40 mg/dl and TG  ≥ 150 mg/dl | SUCRA rank |
| --- | --- | --- | --- | --- | --- | --- | --- | --- | --- | --- | --- | --- |
| NIA+EZT/ST | 0.12  (0.005,2.90) | 1 | N/A | N/A | 0.12  (0.00,2.90) | 1 | N/A | N/A | 0.16  (0.00,5.67) | 1 | N/A | N/A |
| PCSK/ST | **0.74**  **(0.65,0.85)^a^** | 2 | N/A | N/A | **0.74**  **(0.65,0.85)^a^** | 2 | **0.75**  **(0.65,0.86)^a^** | 2 | 0.67  (0.24,1.85) | 2 | N/A | N/A |
| EZT/ST | 0.88  (0.76,1.02) | 3 | N/A | N/A | 0.88  (0.76,1.02) | 3 | 0.88  (0.76,1.01) | 3 | 1.16  (0.30,4.56) | 6 | N/A | N/A |
| CETP/ST | 1.00  (0.91,1.10) | 4 | 0.98  (0.87,1.10) | 1 | 1.06  (0.88,1.27) | 7 | 1.00  (0.91,1.10) | 5 | 1.91  (0.15,24.67) | 8 | 0.99  (0.02,53.92) | 3 |
| ST (reference) | 1.00 | 5 | 1.00 | 2 | 1.00 | 6 | 1.00 | 6 | 1.00 | 4 | 1.00 | 2 |
| OMG3/ST | 1.01  (0.81,1.25) | 6 | N/A | N/A | 1.01  (0.81,1.25) | 5 | 0.18  (0.02,1.56) | 1 | 1.02  (0.41,2.57) | 5 | N/A | N/A |
| FBT/ST | 1.03  (0.70,1.52) | 7 | 1.03  (0.70,1.52) | 3 | N/A | N/A | N/A | N/A | 0.92  (0.26,3.18) | 3 | 0.94  (0.17,5.07) | 1 |
| NIA/ST | 1.01  (0.90,1.14) | 8 | 1.66  (0.90,3.08) | 4 | 1.00  (0.88,1.12) | 4 | 1.00  (0.88,1.13) | 4 | 1.25  (0.23,7.00) | 7 | 1.53  (0.29,8.00) | 4 |
| Global inconsistency chi^2^ (P value) | 0.63  (P=0.8904) |  | 2.63  (P=0.105) |  | 0.63  (P=0.8904) |  | 0.63  (P=0.8884) |  | 0.61  (P=0.4361) |  | 0.25  (P=0.6174) |  |
| Number of studies | 41 |  | 6 |  | 34 |  | 29 |  | 12 |  | 5 |  |

^a^Significant results in bold

eTable 12.8.6 The risk of coronary revascularization

The following table shows the effect sizes (risk ratio) and the rank order (SUCRA ranks) compared stain monotherapy before (standard analysis) and after subgroup analyses.

| Treatment | Standard analysis | SUCRA rank | HDL-C  < 40 mg/dl | SUCRA rank | HDL-C  > 40 mg/dl | SUCRA rank | TG  < 150 mg/dl | SUCRA rank | TG  ≥ 150 mg/dl | SUCRA rank | HDL-C  ≤ 40 mg/dl and TG  ≥ 150 mg/dl | SUCRA rank |
| --- | --- | --- | --- | --- | --- | --- | --- | --- | --- | --- | --- | --- |
| PCSK/ST | **0.84**  **(0.75,0.94)^a^** | 1 | N/A | N/A | **0.84 (0.76,0.93)^a^** | 1 | **0.84 (0.74,0.95)^a^** | 1 | 0.95 (0.62,1.47) | 3 | N/A | N/A |
| NIA/ST | 0.88  (0.72,1.08) | 2 | N/A | N/A | 0.88 (0.74,1.05) | 3 | 0.88 (0.71,1.09) | 2 | N/A | N/A | N/A | N/A |
| OMG3/ST | 0.91  (0.78,1.06) | 3 | N/A | N/A | 0.86 (0.70,1.06) | 2 | 0.88 (0.55,1.42) | 3 | 0.86 (0.71,1.04) | 1 | N/A | N/A |
| EZT/ST | 0.92  (0.80,1.05) | 4 | N/A | N/A | 0.92 (0.82,1.03) | 4 | 0.92 (0.80,1.06) | 4 | 0.67 (0.11,3.87) | 2 | N/A | N/A |
| ST (reference) | 1.00 | 5 | N/A | N/A | 1.00 | 5 | 1.00 | 5 | 1.00 | 4 | N/A | N/A |
| CETP/ST | 1.02  (0.93,1.12) | 6 | N/A | N/A | 1.07 (0.97,1.18) | 6 | 1.02 (0.92,1.13) | 6 | N/A | N/A | N/A | N/A |
| Global inconsistency chi^2^ (P value) | 4.57  (P=0.3347) |  | N/A |  | 0.63  (P=0.8904) |  | 4.61  (P=0.3297) |  | 0.05  (P=0.8226) |  | N/A |  |
| Number of studies | 36 |  | 1 |  | 34 |  | 32 |  | 4 |  | 0 |  |

^a^Significant results in bold

Appendix 13 Sensitivity analyses

eTable 13.1 Sensitivity analyses for the risk of cardiovascular mortality with treatment options

The following table shows the effect sizes (risk ratio) and the rank order (SUCRA ranks) compared stain monotherapy before (standard analysis) and after subgroup analyses.

| Treatment | Standard analysis | SUCRA rank | Excluding study with high-risk of bias | SUCRA rank | Excluding studies with non-adjudicated cardiovascular events | SUCRA rank | Excluding studies with follow-up duration < 1 year | SUCRA rank | Excluding studies with sample size  < 25 percentile | SUCRA rank |
| --- | --- | --- | --- | --- | --- | --- | --- | --- | --- | --- |
| FBT/ST | 0.86  (0.66,1.11) | 1 | 0.85  (0.65,1.12) | 1 | 0.86  (0.66,1.11) | 1 | 0.86  (0.66,1.11) | 1 | 0.86  (0.66,1.11) | 1 |
| NIA+EZT/ST | 0.41  (0.01,20.53) | 2 | N/A | N/A | N/A | N/A | N/A | N/A | 0.41  (0.01,20.50) | 2 |
| CETP/ST | 0.93  (0.84,1.03) | 3 | 0.93  (0.83,1.04) | 2 | 0.93  (0.84,1.03) | 2 | 0.93  (0.84,1.03) | 2 | 0.93  (0.84,1.03) | 3 |
| PCSK/ST | 0.96  (0.86,1.08) | 4 | 0.96  (0.86,1.09) | 4 | 0.97  (0.86,1.08) | 3 | 0.95  (0.85,1.07) | 3 | 0.96  (0.86,1.08) | 4 |
| OMG3/ST | 0.95  (0.71,1.28) | 5 | 0.95  (0.70,1.28) | 3 | 0.96  (0.71,1.30) | 4 | 0.96  (0.71,1.29) | 4 | 0.96  (0.71,1.29) | 5 |
| ST (reference) | 1.00 | 6 | 1.00 | 5 | 1.00 | 5 | 1.00 | 6 | 1.00 | 6 |
| EZT/ST | 1.01  (0.90,1.13) | 7 | 1.02  (0.88,1.18) | 6 | 1.01  (0.90,1.13) | 6 | 1.00  (0.89,1.13) | 5 | 1.01  (0.90,1.13) | 7 |
| NIA/ST | 1.09  (0.96,1.23) | 8 | 1.08  (0.94,1.25) | 8 | 1.09  (0.96,1.24) | 7 | 1.09  (0.96,1.24) | 7 | 1.09  (0.96,1.23) | 8 |
| Global inconsistency chi^2^ (P value) | 4.28  (P=0.2326) |  | 4.25  (P=0.2356) |  | 4.3  (P=0.231) |  | 2.6  (P=0.1067) |  | 4.29  (P=0.2317) |  |
| Number of studies | 44 |  | 30 |  | 27 |  | 29 |  | 38 |  |

eTable 13.2 Sensitivity analyses for the risk of all-cause mortality with treatment options

The following table shows the effect sizes (risk ratio) and the rank order (SUCRA ranks) compared stain monotherapy before (standard analysis) and after subgroup analyses.

| Treatment | Standard analysis | SUCRA rank | Excluding study with high-risk of bias | SUCRA rank | Excluding studies with  non-adjudicated cardiovascular events | SUCRA rank | Excluding studies with follow-up duration < 1 year | SUCRA rank | Excluding studies with sample size  < 25 percentile | SUCRA rank |
| --- | --- | --- | --- | --- | --- | --- | --- | --- | --- | --- |
| FBT/ST | 0.90  (0.68,1.18) | 1 | 0.89  (0.65,1.22) | 2 | 0.90  (0.68,1.18) | 1 | 0.90  (0.76,1.07) | 1 | 0.90  (0.68,1.18) | 1 |
| PCSK/ST | 0.93  (0.79,1.08) | 2 | 0.92  (0.76,1.10) | 1 | 0.93  (0.79,1.08) | 2 | 0.89  (0.67,1.19) | 2 | 0.92  (0.79,1.08) | 2 |
| EZT/ST | 0.97  (0.80,1.17) | 3 | 1.07  (0.79,1.43) | 6 | 0.95  (0.78,1.15) | 3 | 0.92  (0.74,1.15) | 3 | 0.96  (0.79,1.17) | 4 |
| NIA+EZT/ST | 0.92  (0.26,3.29) | 4 | N/A | N/A | N/A | N/A | 1.02  (0.27,3.92) | 4 | 0.39  (0.01,19.63) | 3 |
| ST (reference) | 1.00 | 5 | 1.00 | 1 | 1.00 | 4 | 1.00 | 5 | 1.00 | 5 |
| CETP/ST | 1.00  (0.87,1.15) | 6 | 1.01  (0.86,1.19) | 4 | 1.00  (0.87,1.16) | 5 | 1.01  (0.87,1.17) | 6 | 1.01  (0.87,1.17) | 6 |
| NIA/ST | 1.08  (0.89,1.30) | 7 | 1.04  (0.79,1.38) | 5 | 1.09  (0.90,1.32) | 6 | 1.10  (0.90,1.35) | 8 | 1.08  (0.89,1.31) | 7 |
| OMG3/ST | 1.08  (0.92,1.28) | 8 | 1.08  (0.89,1.31) | 7 | 1.09  (0.92,1.29) | 7 | 1.08  (0.91,1.29) | 7 | 1.09  (0.92,1.29) | 8 |
| Global inconsistency chi^2^ (P value) | 6.61  (P=0.1579) |  | 5.85  (P=0.1193) |  | 6.59  (P=0.0863) |  | 4.09  (P=0.0432) |  | 6.48  (P=0.0906) |  |
| Number of studies | 50 |  | 36 |  | 34 |  | 32 |  | 38 |  |

eTable 13.3 Sensitivity analyses for the risk of coronary heart disease mortality with treatment options

The following table shows the effect sizes (risk ratio) and the rank order (SUCRA ranks) compared stain monotherapy before (standard analysis) and after subgroup analyses.

| Treatment | Standard analysis | SUCRA rank | Excluding study with high-risk of bias | SUCRA rank | Excluding studies with non-adjudicated cardiovascular events | SUCRA rank | Excluding studies with follow-up duration < 1 year | SUCRA rank | Excluding studies with sample size  < 25 percentile | SUCRA rank |
| --- | --- | --- | --- | --- | --- | --- | --- | --- | --- | --- |
| FBT/ST | 0.34  (0.01,8.20) | 1 | 0.34  (0.01,8.20) | 1 | 0.34  (0.01,8.20) | 1 | 0.34  (0.01,8.20) | 1 | 0.34  (0.01,8.20) | 1 |
| NIA+EZT/ST | 0.39  (0.01,19.54) | 2 | N/A | N/A | N/A | N/A | N/A | N/A | 0.39  (0.01,19.47) | 2 |
| PCSK/ST | 0.89  (0.74,1.07) | 3 | 0.89  (0.74,1.07) | 2 | 0.90  (0.75,1.07) | 2 | 0.90  (0.75,1.08) | 2 | 0.89  (0.74,1.07) | 3 |
| CETP/ST | 0.94  (0.84,1.06) | 4 | 0.94  (0.84,1.06) | 3 | 0.94  (0.84,1.06) | 3 | 0.94  (0.84,1.06) | 3 | 0.94  (0.84,1.06) | 4 |
| OMG3/ST | 0.96  (0.73,1.26) | 5 | 0.95  (0.73,1.25) | 4 | 0.97  (0.73,1.27) | 5 | 0.95  (0.72,1.25) | 5 | 0.96  (0.73,1.27) | 6 |
| EZT/ST | 0.96  (0.85,1.09) | 6 | 0.96  (0.85,1.09) | 5 | 0.96  (0.85,1.09) | 4 | 0.96  (0.84,1.08) | 4 | 0.96  (0.85,1.09) | 5 |
| ST (reference) | 1.00 | 7 | 1.00 | 6 | 1.00 | 6 | 1.00 | 6 | 1.00 | 7 |
| NIA/ST | 1.04  (0.90,1.21) | 8 | 1.04  (0.88,1.22) | 7 | 1.04  (0.90,1.21) | 7 | 1.04  (0.90,1.21) | 7 | 1.04  (0.90,1.21) | 8 |
| Global inconsistency chi^2^ (P value) | 1.06  (P=0.787) |  | 1.06  (P=0.7859) |  | 1.07  (P=0.784) |  | 0.4  (P=0.5288) |  | 1.07  (P=0.784) |  |
| Number of studies | 43 |  | 28 |  | 27 |  | 26 |  | 32 |  |

eTable 13.4 Sensitivity analyses for the risk of non-fatal myocardial infarction with treatment options

The following table shows the effect sizes (risk ratio) and the rank order (SUCRA ranks) compared stain monotherapy before (standard analysis) and after subgroup analyses.

| Treatment | Standard analysis | SUCRA rank | Excluding study with high-risk of bias | SUCRA rank | Excluding studies with non-adjudicated cardiovascular events | SUCRA rank | Excluding studies with follow-up duration < 1 year | SUCRA rank | Excluding studies with sample size  < 25 percentile | SUCRA rank |
| --- | --- | --- | --- | --- | --- | --- | --- | --- | --- | --- |
| PCSK/ST | **0.82**  **(0.72,0.93)^a^** | 1 | **0.82**  **(0.71,0.94)^a^** | 1 | **0.82**  **(0.72,0.94)^a^** | 1 | **0.79**  **(0.69,0.90)^a^** | 1 | **0.82**  **(0.72,0.93)^a^** | 1 |
| OMG3/ST | 0.80  (0.55,1.16) | 2 | 0.79  (0.54,1.17) | 2 | 0.80  (0.55,1.16) | 2 | 0.77  (0.53,1.12) | 2 | 0.79  (0.54,1.16) | 2 |
| EZT/ST | 0.87  (0.71,1.07) | 3 | 0.86  (0.69,1.08) | 3 | 0.87  (0.71,1.08) | 3 | 0.86  (0.71,1.06) | 3 | 0.87  (0.71,1.07) | 3 |
| FBT/ST | 0.94  (0.71,1.25) | 4 | 0.94  (0.70,1.27) | 5 | 0.94  (0.71,1.25) | 4 | 0.94  (0.72,1.24) | 4 | 0.94  (0.71,1.25) | 4 |
| CETP/ST | 0.97  (0.84,1.12) | 5 | 0.98  (0.84,1.14) | 6 | 0.97  (0.84,1.12) | 5 | 0.98  (0.85,1.12) | 5 | 0.97  (0.84,1.12) | 5 |
| NIA/ST | 0.99  (0.81,1.22) | 6 | 0.93  (0.72,1.20) | 4 | 0.99  (0.81,1.22) | 6 | 0.99  (0.81,1.21) | 6 | 0.99  (0.81,1.22) | 6 |
| ST (reference) | 1.00 | 7 | 1.00 | 7 | 1.00 | 7 | 1.00 | 7 | 1.00 | 7 |
| Global inconsistency chi^2^ (P value) | 2.2  (P=0.5314) |  | 2.18  (P=0.5351) |  | 2.21  (P=0.5308) |  | 1.53  (P=0.2166) |  | 2.2  (P=0.5315) |  |
| Number of studies | 37 |  | 28 |  | 30 |  | 30 |  | 28 |  |

^a^Significant results are in bold

eTable 13.5 Sensitivity analyses for the risk of any stoke with treatment options

The following table shows the effect sizes (risk ratio) and the rank order (SUCRA ranks) compared stain monotherapy before (standard analysis) and after subgroup analyses.

| Treatment | Standard analysis | SUCRA rank | Excluding study with high-risk of bias | SUCRA rank | Excluding studies with non-adjudicated cardiovascular events | SUCRA rank | Excluding studies with follow-up duration < 1 year | SUCRA rank | Excluding studies with sample size  < 25 percentile | SUCRA rank |
| --- | --- | --- | --- | --- | --- | --- | --- | --- | --- | --- |
| NIA+EZT/ST | 0.12  (0.005,2.90) | 1 | N/A | N/A | N/A | N/A | N/A | N/A | 0.12  (0.00,2.85) | 1 |
| PCSK/ST | **0.74**  **(0.65,0.85)^a^** | 2 | **0.74**  **(0.65,0.85)^a^** | 1 | **0.74**  **(0.65,0.85)^a^** | 1 | **0.76**  **(0.66,0.87)^a^** | 1 | **0.74**  **(0.65,0.85)^a^** | 2 |
| EZT/ST | 0.88  (0.76,1.02) | 3 | 0.87  (0.75,1.01) | 2 | 0.86  (0.74,1.00) | 2 | 0.88  (0.76,1.02) | 2 | 0.86  (0.74,1.00) | 3 |
| CETP/ST | 1.00  (0.91,1.10) | 4 | 1.00  (0.91,1.10) | 4 | 1.00  (0.91,1.10) | 3 | 1.00  (0.91,1.10) | 4 | 1.00  (0.91,1.10) | 4 |
| ST (reference) | 1.00 | 5 | 1.00 | 6 | 1.00 | 4 | 1.00 | 3 | 1.00 | 5 |
| OMG3/ST | 1.01  (0.81,1.25) | 6 | 1.01  (0.81,1.25) | 5 | 1.01  (0.81,1.25) | 5 | 1.01  (0.82,1.26) | 6 | 1.01  (0.81,1.25) | 6 |
| FBT/ST | 1.03  (0.70,1.52) | 7 | 1.03  (0.70,1.52) | 7 | 1.03  (0.70,1.52) | 6 | 1.03  (0.70,1.52) | 5 | 1.03  (0.70,1.52) | 7 |
| NIA/ST | 1.01  (0.90,1.14) | 8 | 1.00  (0.88,1.12) | 3 | 1.02  (0.90,1.15) | 7 | 1.02  (0.90,1.15) | 7 | 1.02  (0.90,1.15) | 8 |
| Global inconsistency chi^2^ (P value) | 0.63  (P=0.8904) |  | 0.63  (P=0.8906) |  | 0.62  (P=0.8907) |  | 0.15  (P=0.7028) |  | 0.63  (P=0.8904) |  |
| Number of studies | 41 |  | 31 |  | 30 |  | 31 |  | 32 |  |

^a^Significant results in bold

eTable 13.6 Sensitivity analyses for the risk of coronary revascularization with treatment options

The following table shows the effect sizes (risk ratio) and the rank order (SUCRA ranks) compared stain monotherapy before (standard analysis) and after subgroup analyses.

| Treatment | Standard analysis | SUCRA rank | Excluding study with high-risk of bias | SUCRA rank | Excluding studies with non-adjudicated cardiovascular events | SUCRA rank | Excluding studies with follow-up duration < 1 year | SUCRA rank | Excluding studies with sample size  < 25 percentile | SUCRA rank |
| --- | --- | --- | --- | --- | --- | --- | --- | --- | --- | --- |
| PCSK/ST | **0.84**  **(0.75,0.94)^a^** | 1 | **0.84**  **(0.75,0.94)^a^** | 1 | **0.84**  **(0.75,0.95)^a^** | 1 | **0.84**  **(0.75,0.95)^a^** | 1 | **0.84**  **(0.75,0.95)^a^** | 1 |
| NIA/ST | 0.88  (0.72,1.08) | 2 | 0.88  (0.72,1.08) | 2 | 0.88  (0.72,1.08) | 2 | 0.88  (0.71,1.09) | 2 | 0.88  (0.71,1.09) | 2 |
| OMG3/ST | 0.91  (0.78,1.06) | 3 | 0.91  (0.78,1.06) | 3 | 0.91  (0.78,1.06) | 4 | 0.90  (0.76,1.06) | 3 | 0.91  (0.78,1.08) | 4 |
| EZT/ST | 0.92  (0.80,1.05) | 4 | 0.93  (0.78,1.10) | 4 | 0.91  (0.78,1.05) | 3 | 0.92  (0.79,1.06) | 4 | 0.90  (0.78,1.05) | 3 |
| ST (reference) | 1.00 | 5 | 1.00 | 5 | 1.00 | 5 | 1.00 | 5 | 1.00 | 5 |
| CETP/ST | 1.02  (0.93,1.12) | 6 | 1.04  (0.94,1.15) | 6 | 1.02  (0.93,1.13) | 6 | 1.02  (0.92,1.13) | 6 | 1.02  (0.93,1.13) | 6 |
| Global inconsistency chi^2^ (P value) | 4.57  (P=0.3347) |  | 4.65  (P=0.3251) |  | 4.52  (P=0.3401) |  | 3.69  (P=0.1583) |  | 4.54  (P=0.3382) |  |
| Number of studies | 36 |  | 28 |  | 29 |  | 28 |  | 24 |  |

^a^Significant results in bold

Appendix 14 Comparison-adjusted funnel plot for each outcome form the network meta-analyses

eFigure 14.1 Comparison-adjusted funnel plot for the network of cardiovascular mortality

**Abbreviation:** CETP/ST=cholesteryl ester transfer protein inhibitor + statin; EZT/ST=ezetimibe + statin; FBT/ST=fibrate + statin; NIA/ST=niacin + statin; OMG3/ST=omega-3 fatty acids + statin; PCSK/ST=proprotein convertase subtilisin/kexin type 9 inhibitor + statin; NIA+EZT/ST= niacin + ezetimibe + statin; ST=statin monotherapy.

eFigure 14.2 Comparison-adjusted funnel plot for the network of all-cause mortality

**Abbreviation:** CETP/ST=cholesteryl ester transfer protein inhibitor + statin; EZT/ST=ezetimibe + statin; FBT/ST=fibrate + statin; NIA/ST=niacin + statin; OMG3/ST=omega-3 fatty acids + statin; PCSK/ST=proprotein convertase subtilisin/kexin type 9 inhibitor + statin; NIA+EZT/ST= niacin + ezetimibe + statin; ST=statin monotherapy.

eFigure 14.3 Comparison-adjusted funnel plot for the network of coronary heart disease mortality

**Abbreviation:** CETP/ST=cholesteryl ester transfer protein inhibitor + statin; EZT/ST=ezetimibe + statin; FBT/ST=fibrate + statin; NIA/ST=niacin + statin; OMG3/ST=omega-3 fatty acids + statin; PCSK/ST=proprotein convertase subtilisin/kexin type 9 inhibitor + statin; NIA+EZT/ST= niacin + ezetimibe + statin; ST=statin monotherapy.

eFigure 14.4 Comparison-adjusted funnel plot for the network of non-fatal myocardial infarction

**Abbreviation:** CETP/ST=cholesteryl ester transfer protein inhibitor + statin; EZT/ST=ezetimibe + statin; FBT/ST=fibrate + statin; NIA/ST=niacin + statin; OMG3/ST=omega-3 fatty acids + statin; PCSK/ST=proprotein convertase subtilisin/kexin type 9 inhibitor + statin; ST=statin monotherapy.

eFigure 14.5 Comparison-adjusted funnel plot for the network of any stroke

**Abbreviation:** CETP/ST=cholesteryl ester transfer protein inhibitor + statin; EZT/ST=ezetimibe + statin; FBT/ST=fibrate + statin; NIA/ST=niacin + statin; OMG3/ST=omega-3 fatty acids + statin; PCSK/ST=proprotein convertase subtilisin/kexin type 9 inhibitor + statin; NIA+EZT/ST= niacin + ezetimibe + statin; ST=statin monotherapy.

eFigure 14.6 Comparison-adjusted funnel plot for the network of coronary revascularization

**Abbreviation:** CETP/ST=cholesteryl ester transfer protein inhibitor + statin; EZT/ST=ezetimibe + statin; NIA/ST=niacin + statin; OMG3/ST=omega-3 fatty acids + statin; PCSK/ST=proprotein convertase subtilisin/kexin type 9 inhibitor + statin; ST=statin monotherapy.

eFigure 14.7 Comparison-adjusted funnel plot for the network of discontinuation due to any cause

**Abbreviation:** CETP/ST=cholesteryl ester transfer protein inhibitor + statin; EZT/ST=ezetimibe + statin; FBT/ST=fibrate + statin; NIA/ST=niacin + statin; OMG3/ST=omega-3 fatty acids + statin; PCSK/ST=proprotein convertase subtilisin/kexin type 9 inhibitor + statin; NIA+EZT/ST= niacin + ezetimibe + statin; ST=statin monotherapy.

eFigure 14.8 Comparison-adjusted funnel plot for the network of discontinuation due to adverse events

**Abbreviation:** CETP/ST=cholesteryl ester transfer protein inhibitor + statin; EZT/ST=ezetimibe + statin; FBT/ST=fibrate + statin; NIA/ST=niacin + statin; OMG3/ST=omega-3 fatty acids + statin; PCSK/ST=proprotein convertase subtilisin/kexin type 9 inhibitor + statin; NIA+EZT/ST= niacin + ezetimibe + statin; ST=statin monotherapy.

Appendix 15 Unpublished studies registered in ClinicalTrials.gov

| **No.** | **ClinicalTrials.gov Identifier** | **Intervention** | **Comparator** | **Number of participants** | **Status** | **Date** |
| --- | --- | --- | --- | --- | --- | --- |
| 1 | NCT00307307 | NIA/ST | ST | 69 | Complete | May 22, 2006 |
| 2 | NCT00349375 | FBT/ST | ST | 1,040 | Complete | July 8, 2009 |
| 3 | NCT00352183 | FBT/ST | ST | 450 | Complete | July 8, 2009 |
| 4 | NCT00362206 | FBT/ST | ST | 423 | Complete | July 8, 2009 |
| 5 | NCT00353522 | CETP/ST | ST | 135 | Complete | November 2, 2016 |
| 6 | NCT00400439 | CETP/ST | ST | 77 | Complete | July 29, 2016 |
| 7 | NCT03192579 | OMG3/ST | ST | 50 | Complete | January 9, 2018 |

**Abbreviation:** CETP/ST=cholesteryl ester transfer protein inhibitor + statin; FBT/ST=fibrate + statin; NIA/ST=niacin + statin; OMG3/ST=omega-3 fatty acids + statin; ST=statin monotherapy.

Appendix 16 Grading

| Comparison | Direct evidence | | Indirect evidence | | Network meta-analysis | |
| --- | --- | --- | --- | --- | --- | --- |
|  | Risk ratio  (95% confidence interval) | Quality of evidence | Risk ratio  (95% confidence interval) | Quality of evidence | Risk ratio  (95% confidence interval) | Quality of evidence |
| **Cardiovascular mortality** | | | | | | |
| CETP/ST vs. ST | 0.93  (0.84,1.03) | ⨁⨁⨁◯^a^ MODERATE | Not estimable | Not estimable | 0.93  (0.84,1.03) | ⨁⨁⨁◯ MODERATE |
| EZT/ST vs. ST | 1.00  (0.89,1.13) | ⨁⨁◯◯^a,b^ LOW | 5.56  (0.81,38.03) | ⨁⨁◯◯^e,d^ LOW | 1.01  (0.90,1.13) | ⨁⨁◯◯ LOW |
| FBT/ST vs. ST | 0.86  (0.66,1.11) | ⨁⨁⨁⨁  HIGH | Not estimable | Not estimable | 0.86  (0.66,1.11) | ⨁⨁⨁⨁  HIGH |
| NIA/ST vs. ST | 1.10  (0.97,1.24) | ⨁⨁⨁◯^a^ MODERATE | 0.19  (0.02,1.61) | ⨁◯◯◯^c,d^  VERY LOW | 1.09  (0.96,1.23) | ⨁⨁⨁◯ MODERATE |
| OMG3/ST vs. ST | 0.95  (0.71,1.28) | ⨁⨁⨁◯^a^ MODERATE | Not estimable | Not estimable | 0.95  (0.71,1.28) | ⨁⨁⨁◯ MODERATE |
| PCSK/ST vs. ST | 0.97  (0.86,1.08) | ⨁⨁⨁◯^a^ MODERATE | 0.09  (0.003,3.25) | ⨁◯◯◯^c,d^ VERY LOW | 0.96  (0.86,1.08) | ⨁⨁⨁◯ MODERATE |
| NIA+EZT/ST vs. ST | N/A | N/A | 0.41  (0.01,20.53) | ⨁◯◯◯^c,d^ VERY LOW | 0.41  (0.01,20.53) | ⨁◯◯◯ VERY LOW |
| EZT/ST vs. CETP/ST | N/A | N/A | 1.09  (0.93,1.27) | ⨁◯◯◯^c,b^ VERY LOW | 1.09  (0.93,1.27) | ⨁◯◯◯ VERY LOW |
| FBT/ST vs. CETP/ST | N/A | N/A | 0.92  (0.69,1.22) | ⨁⨁◯◯^e,b^ LOW | 0.92  (0.69,1.22) | ⨁⨁◯◯ LOW |
| NIA/ST vs. CETP/ST | N/A | N/A | 1.17  (1.00,1.37) | ⨁⨁◯◯^e,b^ LOW | 1.17  (1.00,1.37) | ⨁⨁◯◯ LOW |
| OMG3/ST vs. CETP/ST | N/A | N/A | 1.02  (0.75,1.40) | ⨁⨁◯◯^e,b^ LOW | 1.02  (0.75,1.40) | ⨁⨁◯◯ LOW |
| PCSK/ST vs. CETP/ST | N/A | N/A | 1.04  (0.89,1.21) | ⨁⨁⨁◯^e^  MODERATE | 1.04  (0.89,1.21) | ⨁⨁⨁◯ MODERATE |
| NIA+EZT/ST vs. CETP/ST | N/A | N/A | 0.44  (0.01,22.11) | ⨁◯◯◯^c,d^ VERY LOW | 0.44  (0.01,22.11) | ⨁◯◯◯ VERY LOW |
| FBT/ST vs. EZT/ST | N/A | N/A | 0.85  (0.63,1.13) | ⨁◯◯◯^c,b^ VERY LOW | 0.85  (0.63,1.13) | ⨁◯◯◯ VERY LOW |
| NIA/ST vs. EZT/ST | 0.19  (0.02,1.60) | ⨁⨁⨁◯^d^ MODERATE | 1.09  (0.92,1.29) | ⨁◯◯◯^c,b^ VERY LOW | 1.08  (0.91,1.28) | ⨁⨁⨁◯ MODERATE |
| OMG3/ST vs. EZT/ST | N/A | N/A | 0.94  (0.68,1.29) | ⨁◯◯◯^c,b^ VERY LOW | 0.94  (0.68,1.29) | ⨁◯◯◯ VERY LOW |
| PCSK/ST vs. EZT/ST | 0.27  (0.03,2.41) | ⨁⨁⨁◯^d^ MODERATE | 0.96  (0.82,1.13) | ⨁◯◯◯^c,b^ VERY LOW | 0.95  (0.81,1.12) | ⨁⨁⨁◯ MODERATE |
| NIA+EZT/ST vs. EZT/ST | 0.40  (0.01,20.27) | ⨁◯◯◯^a,d^ VERY LOW | Not estimable | Not estimable | 0.40  (0.01,20.27) | ⨁◯◯◯ VERY LOW |
| NIA/ST vs. FBT/ST | N/A | N/A | 1.27  (0.95,1.70) | ⨁⨁⨁◯^e^  MODERATE | 1.27  (0.95,1.70) | ⨁⨁⨁◯ MODERATE |
| OMG3/ST vs. FBT/ST | N/A | N/A | 1.11  (0.75,1.65) | ⨁⨁⨁◯^e^  MODERATE | 1.11  (0.75,1.65) | ⨁⨁⨁◯  MODERATE |
| PCSK/ST vs. FBT/ST | N/A | N/A | 1.13  (0.85,1.50) | ⨁⨁◯◯^e,b^ LOW | 1.13  (0.85,1.50) | ⨁⨁◯◯ LOW |
| NIA+EZT/ST vs. FBT/ST | N/A | N/A | 0.48  (0.01,24.22) | ⨁◯◯◯^c,d^ VERY LOW | 0.48  (0.01,24.22) | ⨁◯◯◯ VERY LOW |
| OMG3/ST vs. NIA/ST | N/A | N/A | 0.87  (0.63,1.21) | ⨁⨁⨁◯^e^  MODERATE | 0.87  (0.63,1.21) | ⨁⨁⨁◯  MODERATE |
| PCSK/ST vs. NIA/ST | N/A | N/A | 0.89  (0.75,1.05) | ⨁⨁◯◯^e,b^ LOW | 0.89  (0.75,1.05) | ⨁⨁◯◯ LOW |
| NIA+EZT/ST vs. NIA/ST | N/A | N/A | 0.37  (0.01,18.90) | ⨁◯◯◯^c,d^ VERY LOW | 0.37  (0.01,18.90) | ⨁◯◯◯ VERY LOW |
| PCSK/ST vs. OMG3/ST | N/A | N/A | 1.01  (0.74,1.39) | ⨁⨁◯◯^e,b^ LOW | 1.01  (0.74,1.39) | ⨁⨁◯◯ LOW |
| NIA+EZT/ST vs. OMG3/ST | N/A | N/A | 0.43  (0.01,21.82) | ⨁◯◯◯^c,d^ VERY LOW | 0.43  (0.01,21.82) | ⨁◯◯◯ VERY LOW |
| NIA+EZT/ST vs. PCSK/ST | N/A | N/A | 0.42  (0.01,21.34) | ⨁◯◯◯^c,d^ VERY LOW | 0.42  (0.01,21.34) | ⨁◯◯◯ VERY LOW |
| **All-cause mortality** | | | | | | |
| CETP/ST vs. ST | 1.00  (0.87,1.15) | ⨁⨁⨁◯^a^ MODERATE | Not estimable | Not estimable | 1.00  (0.87,1.15) | ⨁⨁⨁◯  MODERATE |
| EZT/ST vs. ST | 0.95  (0.78,1.15) | ⨁⨁◯◯^a,b^ LOW | 6.78  (1.13,40.61) | ⨁◯◯◯^c,d^ VERY LOW | 0.97  (0.80,1.17) | ⨁⨁◯◯ LOW |
| FBT/ST vs. ST | 0.90  (0.68,1.18) | ⨁⨁⨁⨁  HIGH | Not estimable | Not estimable | 0.90  (0.68,1.18) | ⨁⨁⨁⨁  HIGH |
| NIA/ST vs. ST | 1.09  (0.91,1.32) | ⨁⨁⨁◯^a^ MODERATE | 0.13  (0.02,1.05) | ⨁◯◯◯^c,d^ VERY LOW | 1.08  (0.89,1.30) | ⨁⨁⨁◯ MODERATE |
| OMG3/ST vs. ST | 1.08  (0.92,1.28) | ⨁⨁⨁◯^a^ MODERATE | Not estimable | Not estimable | 1.08  (0.92,1.28) | ⨁⨁⨁◯ MODERATE |
| PCSK/ST vs. ST | 0.93  (0.80,1.08) | ⨁⨁⨁◯^a^ MODERATE | 0.02  (0.0002,2.57) | ⨁◯◯◯^c,d^ VERY LOW | 0.93  (0.79,1.08) | ⨁⨁⨁◯ MODERATE |
| NIA+EZT/ST vs. ST | 1.02  (0.27,3.91) | ⨁⨁◯◯^a^ LOW | 0.39  (0.01,19.79) | ⨁◯◯◯^c,d^ VERY LOW | 0.92  (0.26,3.29) | ⨁⨁◯◯ LOW |
| EZT/ST vs. CETP/ST | N/A | N/A | 0.89  (0.67,1.19) | ⨁◯◯◯^c,b^ VERY LOW | 0.97  (0.77,1.22) | ⨁◯◯◯ VERY LOW |
| FBT/ST vs. CETP/ST | N/A | N/A | 0.78  (0.54,1.12) | ⨁⨁◯◯^e,b^  LOW | 0.93  (0.67,1.29) | ⨁⨁◯◯ LOW |
| NIA/ST vs. CETP/ST | N/A | N/A | 0.93  (0.68,1.28) | ⨁⨁◯◯^e,b^  LOW | 1.07  (0.84,1.37) | ⨁⨁◯◯ LOW |
| OMG3/ST vs. CETP/ST | N/A | N/A | 0.93  (0.69,1.26) | ⨁⨁◯◯^e,b^  LOW | 1.08  (0.87,1.35) | ⨁⨁◯◯ LOW |
| PCSK/ST vs. CETP/ST | N/A | N/A | 0.84  (0.57,1.23) | ⨁⨁⨁◯^e^  MODERATE | 0.92  (0.74,1.15) | ⨁⨁⨁◯ MODERATE |
| NIA+EZT/ST vs. CETP/ST | N/A | N/A | 0.80  (0.22,2.92) | ⨁◯◯◯^c,b^ VERY LOW | 0.92  (0.26,3.30) | ⨁◯◯◯ VERY LOW |
| FBT/ST vs. EZT/ST | N/A | N/A | 0.87  (0.59,1.28) | ⨁◯◯◯^c,b^ VERY LOW | 0.93  (0.67,1.29) | ⨁◯◯◯ VERY LOW |
| NIA/ST vs. EZT/ST | 0.13  (0.02,1.09) | ⨁⨁⨁◯^d^ MODERATE | 1.15  (0.88,1.50) | ⨁◯◯◯^c,b^ VERY LOW | 1.11  (0.85,1.45) | ⨁⨁⨁◯ MODERATE |
| OMG3/ST vs. EZT/ST | N/A | N/A | 1.05  (0.76,1.44) | ⨁◯◯◯ VERY LOW | 1.12  (0.87,1.44) | ⨁◯◯◯ VERY LOW |
| PCSK/ST vs. EZT/ST | 0.13  (0.01,2.37) | ⨁⨁⨁◯^d^ MODERATE | 0.97  (0.76,1.23) | ⨁◯◯◯^c,b^ VERY LOW | 0.95  (0.75,1.22) | ⨁⨁⨁◯ MODERATE |
| NIA+EZT/ST vs. EZT/ST | 0.40  (0.01,20.38) | ⨁◯◯◯^a,d^ VERY LOW | 1.06  (0.27,4.09) | ⨁◯◯◯^c,b^ VERY LOW | 0.95  (0.27,3.43) | ⨁◯◯◯ VERY LOW |
| NIA/ST vs. FBT/ST | N/A | N/A | 1.20  (0.88,1.65) | ⨁⨁⨁◯^e^  MODERATE | 1.20  (0.87,1.66) | ⨁⨁⨁◯ MODERATE |
| OMG3/ST vs. FBT/ST | N/A | N/A | 1.20  (0.88,1.64) | ⨁⨁⨁◯^e^  MODERATE | 1.21  (0.88,1.66) | ⨁⨁⨁◯ MODERATE |
| PCSK/ST vs. FBT/ST | N/A | N/A | 1.08  (0.78,1.51) | ⨁⨁◯◯^e , b^  LOW | 1.03  (0.76,1.40) | ⨁⨁◯◯ LOW |
| NIA+EZT/ST vs. FBT/ST | N/A | N/A | 1.04  (0.28,3.78) | ⨁⨁◯◯^c^ LOW | 1.03  (0.28,3.77) | ⨁⨁◯◯ LOW |
| OMG3/ST vs. NIA/ST | N/A | N/A | 1.00  (0.79,1.28) | ⨁⨁⨁◯^e^  MODERATE | 1.01  (0.78,1.29) | ⨁⨁⨁◯ MODERATE |
| PCSK/ST vs. NIA/ST | N/A | N/A | 0.90  (0.69,1.18) | ⨁⨁◯◯^e,b^  LOW | 0.86  (0.68,1.09) | ⨁⨁◯◯ LOW |
| NIA+EZT/ST vs. NIA/ST | N/A | N/A | 0.86  (0.24,3.11) | ⨁⨁◯◯^c^ LOW | 0.86  (0.24,3.10) | ⨁⨁◯◯ LOW |
| PCSK/ST vs. OMG3/ST | N/A | N/A | 0.90  (0.69,1.18) | ⨁⨁◯◯^e,b^  LOW | 0.85  (0.68,1.07) | ⨁⨁◯◯ LOW |
| NIA+EZT/ST vs. OMG3/ST | N/A | N/A | 0.86  (0.24,3.09) | ⨁⨁◯◯^c^ LOW | 0.85  (0.24,3.07) | ⨁⨁◯◯ LOW |
| NIA+EZT/ST vs. PCSK/ST | N/A | N/A | 0.96  (0.26,3.47) | ⨁◯◯◯^c,b^ VERY LOW | 1.00  (0.28,3.58) | ⨁◯◯◯ VERY LOW |
| **Coronary heart disease mortality** | | | | | | |
| CETP/ST vs. ST | 0.94  (0.84,1.06) | ⨁⨁⨁◯^a^ MODERATE | Not estimable | Not estimable | 0.94  (0.84,1.06) | ⨁⨁⨁◯ MODERATE |
| EZT/ST vs. ST | 0.96  (0.85,1.09) | ⨁⨁⨁◯^a^ MODERATE | 1.80  (0.29,10.98) | ⨁⨁◯◯^e,d^  LOW | 0.96  (0.85,1.09) | ⨁⨁⨁◯ MODERATE |
| FBT/ST vs. ST | 0.34  (0.01,8.20) | ⨁⨁⨁◯^d^ MODERATE | Not estimable | Not estimable | 0.34  (0.01,8.20) | ⨁⨁⨁◯ MODERATE |
| NIA/ST vs. ST | 1.04  (0.90,1.21) | ⨁⨁⨁◯^a^ MODERATE | Not estimable | Not estimable | 1.04  (0.90,1.21) | ⨁⨁⨁◯ MODERATE |
| OMG3/ST vs. ST | 0.96  (0.73,1.26) | ⨁⨁⨁◯^a^ MODERATE | Not estimable | Not estimable | 0.96  (0.73,1.26) | ⨁⨁⨁◯ MODERATE |
| PCSK/ST vs. ST | 0.90  (0.75,1.08) | ⨁⨁⨁◯^a^ MODERATE | 0.40  (0.07,2.32) | ⨁⨁◯◯^e,d^ LOW | 0.89  (0.74,1.07) | ⨁⨁⨁◯ MODERATE |
| NIA+EZT/ST vs. ST | N/A | N/A | 0.39  (0.01,19.54) | ⨁◯◯◯^c,d^ VERY LOW | 0.39  (0.01,19.54) | ⨁◯◯◯ VERY LOW |
| EZT/ST vs. CETP/ST | N/A | N/A | 1.01  (0.74,1.36) | ⨁⨁⨁◯^e^  MODERATE | 1.01  (0.74,1.36) | ⨁⨁⨁◯ MODERATE |
| FBT/ST vs. CETP/ST | N/A | N/A | 0.36  (0.01,8.74) | ⨁◯◯◯^e,b,d^ VERY LOW | 0.36  (0.01,8.74) | ⨁◯◯◯ VERY LOW |
| NIA/ST vs. CETP/ST | N/A | N/A | 1.11  (0.92,1.34) | ⨁⨁◯◯^e,b^  LOW | 1.11  (0.92,1.34) | ⨁⨁◯◯ LOW |
| OMG3/ST vs. CETP/ST | N/A | N/A | 1.02  (0.76,1.37) | ⨁⨁◯◯^e,b^  LOW | 1.02  (0.76,1.37) | ⨁⨁◯◯ LOW |
| PCSK/ST vs. CETP/ST | N/A | N/A | 0.95  (0.76,1.18) | ⨁⨁⨁◯^e^  MODERATE | 0.95  (0.76,1.18) | ⨁⨁⨁◯ MODERATE |
| NIA+EZT/ST vs. CETP/ST | N/A | N/A | 0.41  (0.01,20.82) | ⨁◯◯◯^c,b,d^ VERY LOW | 0.41  (0.01,20.82) | ⨁◯◯◯ VERY LOW |
| FBT/ST vs. EZT/ST | N/A | N/A | 0.35  (0.01,8.55) | ⨁⨁◯◯^e,d^ LOW | 0.35  (0.01,8.55) | ⨁⨁◯◯ LOW |
| NIA/ST vs. EZT/ST | N/A | N/A | 1.08  (0.89,1.32) | ⨁⨁⨁◯^e^  MODERATE | 1.08  (0.89,1.32) | ⨁⨁⨁◯ MODERATE |
| OMG3/ST vs. EZT/ST | N/A | N/A | 0.99  (0.74,1.34) | ⨁⨁⨁◯^e^  MODERATE | 0.99  (0.74,1.34) | ⨁⨁⨁◯ MODERATE |
| PCSK/ST vs. EZT/ST | 0.47  (0.10,2.16) | ⨁⨁⨁◯^d^ MODERATE | 0.94  (0.75,1.17) | ⨁⨁⨁◯^e^  MODERATE | 0.93  (0.74,1.15) | ⨁⨁⨁◯ MODERATE |
| NIA+EZT/ST vs. EZT/ST | 0.40  (0.01,20.27) | ⨁◯◯◯^a,d^ VERY LOW | Not estimable | Not estimable | 0.40  (0.01,20.27) | ⨁◯◯◯ VERY LOW |
| NIA/ST vs. FBT/ST | N/A | N/A | 3.11  (0.13,76.34) | ⨁⨁◯◯^e,d^ LOW | 3.11  (0.13,76.34) | ⨁⨁◯◯ LOW |
| OMG3/ST vs. FBT/ST | N/A | N/A | 2.85  (0.12,70.64) | ⨁⨁◯◯^e,d^ LOW | 2.85  (0.12,70.64) | ⨁⨁◯◯ LOW |
| PCSK/ST vs. FBT/ST | N/A | N/A | 2.66  (0.11,65.32) | ⨁◯◯◯^e,b,d^ VERY LOW | 2.66  (0.11,65.32) | ⨁◯◯◯ VERY LOW |
| NIA+EZT/ST vs. FBT/ST | N/A | N/A | 1.16  (0.01,181.96) | ⨁◯◯◯^c,d^ VERY LOW | 1.16  (0.01,181.96) | ⨁◯◯◯ VERY LOW |
| OMG3/ST vs. NIA/ST | N/A | N/A | 0.92  (0.67,1.25) | ⨁⨁⨁◯^e^  MODERATE | 0.92  (0.67,1.25) | ⨁⨁⨁◯ MODERATE |
| PCSK/ST vs. NIA/ST | N/A | N/A | 0.85  (0.67,1.08) | ⨁⨁◯◯^e,b^  LOW | 0.85  (0.67,1.08) | ⨁⨁◯◯ LOW |
| NIA+EZT/ST vs. NIA/ST | N/A | N/A | 0.37  (0.01,18.79) | ⨁◯◯◯^c,d^ VERY LOW | 0.37  (0.01,18.79) | ⨁◯◯◯ VERY LOW |
| PCSK/ST vs. OMG3/ST | N/A | N/A | 0.93  (0.67,1.29) | ⨁⨁◯◯^e,b^  LOW | 0.93  (0.67,1.29) | ⨁⨁◯◯ LOW |
| NIA+EZT/ST vs. OMG3/ST | N/A | N/A | 0.41  (0.01,20.61) | ⨁◯◯◯^c,d^ VERY LOW | 0.41  (0.01,20.61) | ⨁◯◯◯ VERY LOW |
| NIA+EZT/ST vs. PCSK/ST | N/A | N/A | 0.44  (0.01,22.03) | ⨁◯◯◯^c,d^ VERY LOW | 0.44  (0.01,22.03) | ⨁◯◯◯ VERY LOW |
| **Non-fatal myocardial infarction** | | | | | | |
| CETP/ST vs. ST | 0.97  (0.84,1.12) | ⨁⨁⨁◯^a^ MODERATE | Not estimable | Not estimable | 0.97  (0.84,1.12) | ⨁⨁⨁◯ MODERATE |
| EZT/ST vs. ST | 0.89  (0.72,1.10) | ⨁⨁⨁◯^a^ MODERATE | 0.44  (0.13,1.55) | ⨁◯◯◯^c,d^ VERY LOW | 0.87  (0.71,1.07) | ⨁⨁⨁◯ MODERATE |
| FBT/ST vs. ST | 0.94  (0.71,1.25) | ⨁⨁⨁⨁  HIGH | Not estimable | Not estimable | 0.94  (0.71,1.25) | ⨁⨁⨁⨁  HIGH |
| NIA/ST vs. ST | 0.99  (0.81,1.22) | ⨁⨁⨁◯^a^ MODERATE | Not estimable | Not estimable | 0.99  (0.81,1.22) | ⨁⨁⨁◯ MODERATE |
| OMG3/ST vs. ST | 0.80  (0.55,1.16) | ⨁⨁⨁◯^a^ MODERATE | Not estimable | Not estimable | 0.80  (0.55,1.16) | ⨁⨁⨁◯ MODERATE |
| PCSK/ST vs. ST | 0.81  (0.71,0.93) | ⨁⨁⨁◯^a^ MODERATE | 1.49  (0.43,5.23) | ⨁⨁◯◯^e,d^ LOW | 0.82  (0.72,0.93) | ⨁⨁⨁◯ MODERATE |
| EZT/ST vs. CETP/ST | N/A | N/A | 0.90  (0.70,1.15) | ⨁⨁⨁◯^e^  MODERATE | 0.90  (0.70,1.15) | ⨁⨁⨁◯ MODERATE |
| FBT/ST vs. CETP/ST | N/A | N/A | 0.97  (0.71,1.33) | ⨁⨁⨁◯^e^  MODERATE | 0.97  (0.71,1.33) | ⨁⨁⨁◯ MODERATE |
| NIA/ST vs. CETP/ST | N/A | N/A | 1.02  (0.80,1.31) | ⨁⨁⨁◯^e^  MODERATE | 1.02  (0.80,1.31) | ⨁⨁⨁◯ MODERATE |
| OMG3/ST vs. CETP/ST | N/A | N/A | 0.82  (0.55,1.23) | ⨁⨁⨁◯^e^  MODERATE | 0.82  (0.55,1.23) | ⨁⨁⨁◯ MODERATE |
| PCSK/ST vs. CETP/ST | N/A | N/A | 0.84  (0.69,1.02) | ⨁⨁⨁◯^e^  MODERATE | 0.84  (0.69,1.02) | ⨁⨁⨁◯ MODERATE |
| FBT/ST vs. EZT/ST | N/A | N/A | 1.08  (0.76,1.53) | ⨁⨁⨁◯^e^  MODERATE | 1.08  (0.76,1.53) | ⨁⨁⨁◯ MODERATE |
| NIA/ST vs. EZT/ST | N/A | N/A | 1.14  (0.85,1.52) | ⨁⨁⨁◯^e^  MODERATE | 1.14  (0.85,1.52) | ⨁⨁⨁◯ MODERATE |
| OMG3/ST vs. EZT/ST | N/A | N/A | 0.92  (0.60,1.40) | ⨁⨁⨁◯^e^  MODERATE | 0.92  (0.60,1.40) | ⨁⨁⨁◯ MODERATE |
| PCSK/ST vs. EZT/ST | 0.66  (0.20,2.15) | ⨁⨁⨁⨁  HIGH | 0.92  (0.71,1.18) | ⨁⨁⨁◯^e^  MODERATE | 0.94  (0.74,1.20) | ⨁⨁⨁⨁  HIGH |
| NIA/ST vs. FBT/ST | N/A | N/A | 1.05  (0.74,1.49) | ⨁⨁⨁◯^e^  MODERATE | 1.05  (0.74,1.49) | ⨁⨁⨁◯ MODERATE |
| OMG3/ST vs. FBT/ST | N/A | N/A | 0.85  (0.53,1.35) | ⨁⨁⨁◯^e^  MODERATE | 0.85  (0.53,1.35) | ⨁⨁⨁◯ MODERATE |
| PCSK/ST vs. FBT/ST | N/A | N/A | 0.87  (0.64,1.19) | ⨁◯◯◯^c,b^ VERY LOW | 0.87  (0.64,1.19) | ⨁◯◯◯ VERY LOW |
| OMG3/ST vs. NIA/ST | N/A | N/A | 0.80  (0.53,1.23) | ⨁⨁⨁◯^e^  MODERATE | 0.80  (0.53,1.23) | ⨁⨁⨁◯ MODERATE |
| PCSK/ST vs. NIA/ST | N/A | N/A | 0.83  (0.65,1.05) | ⨁⨁◯◯^e,b^  LOW | 0.83  (0.65,1.05) | ⨁⨁◯◯ LOW |
| PCSK/ST vs. OMG3/ST | N/A | N/A | 1.03  (0.69,1.53) | ⨁⨁◯◯^e,b^  LOW | 1.03  (0.69,1.53) | ⨁⨁◯◯ LOW |
| **Any stroke** | | | | | | |
| CETP/ST vs. ST | 1.00  (0.91,1.10) | ⨁⨁⨁◯^a^ MODERATE | Not estimable | Not estimable | 1.00  (0.91,1.10) | ⨁⨁◯◯ LOW |
| EZT/ST vs. ST | 0.88  (0.76,1.01) | ⨁⨁⨁◯^a^ MODERATE | 1.65  (0.13,20.68) | ⨁⨁⨁◯e  MODERATE | 0.88  (0.76,1.02) | ⨁⨁⨁◯ MODERATE |
| FBT/ST vs. ST | 1.03  (0.70,1.52) | ⨁⨁⨁⨁  HIGH | Not estimable | Not estimable | 1.03  (0.70,1.52) | ⨁⨁⨁⨁  HIGH |
| NIA/ST vs. ST | 1.01  (0.90,1.14) | ⨁⨁⨁◯^a^ MODERATE | Not estimable | Not estimable | 1.01  (0.90,1.14) | ⨁⨁⨁◯ MODERATE |
| OMG3/ST vs. ST | 1.01  (0.81,1.25) | ⨁⨁⨁◯^a^ MODERATE | Not estimable | Not estimable | 1.01  (0.81,1.25) | ⨁⨁⨁◯ MODERATE |
| PCSK/ST vs. ST | 0.74  (0.65,0.85) | ⨁⨁⨁⨁  HIGH | 0.62  (0.05,7.83) | ⨁⨁◯◯^e,d^ LOW | 0.74  (0.65,0.85) | ⨁⨁⨁⨁  HIGH |
| NIA+EZT/ST vs. ST | N/A | N/A | 0.12  (0.00,2.90) | ⨁◯◯◯^c,d^ VERY LOW | 0.12  (0.00,2.90) | ⨁◯◯◯ VERY LOW |
| EZT/ST vs. CETP/ST | N/A | N/A | 0.88  (0.74,1.05) | ⨁⨁⨁◯^e^  MODERATE | 0.88  (0.74,1.05) | ⨁⨁⨁◯ MODERATE |
| FBT/ST vs. CETP/ST | N/A | N/A | 1.03  (0.69,1.54) | ⨁⨁◯◯^e,b^  LOW | 1.03  (0.69,1.54) | ⨁⨁◯◯ LOW |
| NIA/ST vs. CETP/ST | N/A | N/A | 1.01  (0.87,1.18) | ⨁◯◯◯^c,b^ VERY LOW | 1.01  (0.87,1.18) | ⨁◯◯◯ VERY LOW |
| OMG3/ST vs. CETP/ST | N/A | N/A | 1.01  (0.80,1.27) | ⨁◯◯◯^c,b^ VERY LOW | 1.01  (0.80,1.27) | ⨁◯◯◯ VERY LOW |
| PCSK/ST vs. CETP/ST | N/A | N/A | 0.74  (0.63,0.88) | ⨁⨁⨁◯^e^  MODERATE | 0.74  (0.63,0.88) | ⨁⨁⨁◯ MODERATE |
| NIA+EZT/ST vs. CETP/ST | N/A | N/A | 0.12  (0.00,2.91) | ⨁◯◯◯^c,b,d^ VERY LOW | 0.12  (0.00,2.91) | ⨁◯◯◯ VERY LOW |
| FBT/ST vs. EZT/ST | N/A | N/A | 1.17  (0.77,1.77) | ⨁⨁⨁◯^e^  MODERATE | 1.17  (0.77,1.77) | ⨁⨁⨁◯ MODERATE |
| NIA/ST vs. EZT/ST | N/A | N/A | 1.15  (0.96,1.39) | ⨁⨁⨁◯^e^  MODERATE | 1.15  (0.96,1.39) | ⨁⨁⨁◯ MODERATE |
| OMG3/ST vs. EZT/ST | N/A | N/A | 1.15  (0.89,1.48) | ⨁⨁⨁◯^e^  MODERATE | 1.15  (0.89,1.48) | ⨁⨁⨁◯ MODERATE |
| PCSK/ST vs. EZT/ST | 0.63  (0.07,6.02) | ⨁⨁⨁◯^d^ MODERATE | 0.85  (0.70,1.03) | ⨁⨁⨁◯^e^  MODERATE | 0.84  (0.69,1.03) | ⨁⨁⨁◯ MODERATE |
| NIA+EZT/ST vs. EZT/ST | 0.13  (0.01,3.29) | ⨁◯◯◯^a,d^ VERY LOW | Not estimable | Not estimable | 0.13  (0.01,3.29) | ⨁◯◯◯ VERY LOW |
| NIA/ST vs. FBT/ST | N/A | N/A | 0.99  (0.66,1.48) | ⨁⨁⨁◯^e^  MODERATE | 0.99  (0.66,1.48) | ⨁⨁⨁◯ MODERATE |
| OMG3/ST vs. FBT/ST | N/A | N/A | 0.98  (0.63,1.52) | ⨁⨁⨁◯^e^  MODERATE | 0.98  (0.63,1.52) | ⨁⨁⨁◯ MODERATE |
| PCSK/ST vs. FBT/ST | N/A | N/A | 0.72  (0.48,1.09) | ⨁⨁⨁◯^f ,b^ MODERATE | 0.72  (0.48,1.09) | ⨁⨁⨁◯ MODERATE |
| NIA+EZT/ST vs. FBT/ST | N/A | N/A | 0.11  (0.00,2.89) | ⨁◯◯◯^c,d^ VERY LOW | 0.11  (0.00,2.89) | ⨁◯◯◯ VERY LOW |
| OMG3/ST vs. NIA/ST | N/A | N/A | 0.99  (0.78,1.27) | ⨁⨁⨁◯^e^  MODERATE | 0.99  (0.78,1.27) | ⨁⨁⨁◯ MODERATE |
| PCSK/ST vs. NIA/ST | N/A | N/A | 0.73  (0.61,0.87) | ⨁⨁◯◯^e,b^ Low | 0.73  (0.61,0.87) | ⨁⨁◯◯ LOW |
| NIA+EZT/ST vs. NIA/ST | N/A | N/A | 0.12  (0.00,2.87) | ⨁◯◯◯^c^ VERY LOW | 0.12  (0.00,2.87) | ⨁◯◯◯ VERY LOW |
| PCSK/ST vs. OMG3/ST | N/A | N/A | 0.74  (0.57,0.95) | ⨁⨁◯◯^e,b^ Low | 0.74  (0.57,0.95) | ⨁⨁◯◯ LOW |
| NIA+EZT/ST vs. OMG3/ST | N/A | N/A | 0.12  (0.00,2.90) | ⨁◯◯◯^c^ VERY LOW | 0.12  (0.00,2.90) | ⨁◯◯◯ VERY LOW |
| NIA+EZT/ST vs. PCSK/ST | N/A | N/A | 0.16  (0.01,3.92) | ⨁◯◯◯^c,d^ VERY LOW | 0.16  (0.01,3.92) | ⨁◯◯◯ VERY LOW |
| **Coronary revascularization** | | | | | | |
| CETP/ST vs. ST | 1.02  (0.93,1.12) | ⨁⨁◯◯^a,g^ Low | Not estimable | Not estimable | 1.02  (0.93,1.12) | ⨁⨁◯◯ Low |
| EZT/ST vs. ST | 0.92  (0.81,1.06) | ⨁⨁⨁◯^a^ MODERATE | 0.53  (0.20,1.45) | ⨁⨁⨁◯^e^  MODERATE | 0.92  (0.80,1.05) | ⨁⨁⨁◯ MODERATE |
| NIA/ST vs. ST | 0.89  (0.73,1.09) | ⨁⨁⨁⨁  HIGH | 0.12  (0.01,2.38) | ⨁⨁◯◯^e,d^ LOW | 0.88  (0.72,1.08) | ⨁⨁⨁⨁  HIGH |
| OMG3/ST vs. ST | 0.91  (0.78,1.06) | ⨁⨁⨁◯^a^ MODERATE | Not estimable | Not estimable | 0.91  (0.78,1.06) | ⨁⨁⨁◯ MODERATE |
| PCSK/ST vs. ST | 0.84  (0.75,0.94) | ⨁⨁⨁◯^a^ MODERATE | 1.87  (0.64,5.50) | ⨁⨁◯◯^e,d^ LOW | 0.84  (0.75,0.94) | ⨁⨁⨁◯  MODERATE |
| EZT/ST vs. CETP/ST | N/A | N/A | 0.90  (0.76,1.06) | ⨁⨁◯◯^c^ LOW | 0.90  (0.76,1.06) | ⨁⨁◯◯ LOW |
| NIA/ST vs. CETP/ST | N/A | N/A | 0.86  (0.69,1.08) | ⨁⨁◯◯^c^ LOW | 0.86  (0.69,1.08) | ⨁⨁◯◯ LOW |
| OMG3/ST vs. CETP/ST | N/A | N/A | 0.89  (0.74,1.06) | ⨁⨁◯◯^c^ LOW | 0.89  (0.74,1.06) | ⨁⨁◯◯ LOW |
| PCSK/ST vs. CETP/ST | N/A | N/A | 0.83  (0.71,0.96) | ⨁⨁◯◯c  LOW | 0.83  (0.71,0.96) | ⨁⨁◯◯  LOW |
| NIA/ST vs. EZT/ST | 0.13  (0.01,2.60) | ⨁⨁⨁◯^d^ MODERATE | 0.98  (0.76,1.25) | ⨁⨁⨁◯^e^  MODERATE | 0.96  (0.76,1.23) | ⨁⨁⨁◯ MODERATE |
| OMG3/ST vs. EZT/ST | N/A | N/A | 0.99  (0.81,1.21) | ⨁⨁⨁◯^e^  MODERATE | 0.99  (0.81,1.21) | ⨁⨁⨁◯ MODERATE |
| PCSK/ST vs. EZT/ST | 2.05  (0.76,5.58) | ⨁⨁⨁◯^d^ MODERATE | 0.90  (0.75,1.07) | ⨁⨁⨁◯^e^  MODERATE | 0.92  (0.77,1.10) | ⨁⨁⨁◯ MODERATE |
| OMG3/ST vs. NIA/ST | N/A | N/A | 1.03  (0.80,1.33) | ⨁⨁⨁◯^e^  MODERATE | 1.03  (0.80,1.33) | ⨁⨁⨁◯ MODERATE |
| PCSK/ST vs. NIA/ST | N/A | N/A | 0.96  (0.76,1.21) | ⨁⨁⨁◯^e^  MODERATE | 0.96  (0.76,1.21) | ⨁⨁⨁◯  MODERATE |
| PCSK/ST vs. OMG3/ST | N/A | N/A | 0.93  (0.77,1.12) | ⨁⨁⨁◯^e^  MODERATE | 0.93  (0.77,1.12) | ⨁⨁⨁◯  MODERATE |
| **Discontinuation due to any cause** | | | | | | |
| CETP/ST vs. ST | 1.07  (0.95,1.21) | ⨁⨁◯◯^a,g^ LOW | Not estimable | Not estimable | 1.07  (0.95,1.21) | ⨁⨁◯◯ LOW |
| EZT/ST vs. ST | 1.13  (0.94,1.36) | ⨁⨁⨁◯^a^ MODERATE | 0.97  (0.67,1.41) | ⨁⨁⨁◯^e^  MODERATE | 1.09  (0.93,1.29) | ⨁⨁⨁◯ MODERATE |
| FBT/ST vs. ST | 1.14  (0.86,1.50) | ⨁⨁⨁⨁  HIGH | Not estimable | Not estimable | 1.14  (0.86,1.50) | ⨁⨁⨁⨁  HIGH |
| NIA/ST vs. ST | 1.35  (1.12,1.63) | ⨁⨁◯◯^a,g^ LOW | 1.84  (0.93,3.61) | ⨁⨁⨁◯^e^  MODERATE | 1.38  (1.15,1.65) | ⨁⨁⨁◯ MODERATE |
| OMG3/ST vs. ST | 1.17  (0.97,1.40) | ⨁⨁◯◯^a,g^ LOW | Not estimable | Not estimable | 1.17  (0.97,1.40) | ⨁⨁◯◯ LOW |
| PCSK/ST vs. ST | 1.05  (0.93,1.18) | ⨁⨁⨁◯^a^ MODERATE | 0.93  (0.58,1.51) | ⨁⨁⨁◯^e^  MODERATE | 1.04  (0.93,1.17) | ⨁⨁⨁◯ MODERATE |
| NIA+EZT/ST vs. ST | N/A | N/A | 2.07  (1.40,3.06) | ⨁⨁◯◯^c^ LOW | 2.13  (1.40,3.24) | ⨁⨁◯◯ LOW |
| EZT/ST vs. CETP/ST | N/A | N/A | 0.90  (0.73,1.11) | ⨁⨁◯◯^c^ LOW | 1.02  (0.83,1.26) | ⨁⨁◯◯ LOW |
| FBT/ST vs. CETP/ST | N/A | N/A | 0.96  (0.72,1.29) | ⨁⨁◯◯^c^ LOW | 1.06  (0.79,1.44) | ⨁⨁◯◯ LOW |
| NIA/ST vs. CETP/ST | N/A | N/A | 1.17  (0.94,1.45) | ⨁⨁◯◯^c^ LOW | 1.29  (1.04,1.60) | ⨁⨁◯◯ LOW |
| OMG3/ST vs. CETP/ST | N/A | N/A | 1.02  (0.82,1.27) | ⨁⨁◯◯^c^ LOW | 1.09  (0.88,1.35) | ⨁⨁◯◯ LOW |
| PCSK/ST vs. CETP/ST | N/A | N/A | 0.87  (0.73,1.05) | ⨁⨁◯◯^c^ LOW | 0.97  (0.82,1.15) | ⨁⨁◯◯ LOW |
| NIA+EZT/ST vs. CETP/ST | N/A | N/A | 1.75  (1.16,2.66) | ⨁⨁◯◯ LOW | 1.99  (1.28,3.08) | ⨁⨁◯◯ LOW |
| FBT/ST vs. EZT/ST | N/A | N/A | 1.07  (0.79,1.44) | ⨁⨁◯◯^c^ LOW | 1.04  (0.75,1.44) | ⨁⨁◯◯ LOW |
| NIA/ST vs. EZT/ST | 1.65  (0.86,3.17) | ⨁⨁⨁⨁ HIGH | 1.21  (0.94,1.56) | ⨁⨁◯◯^c^ LOW | 1.26  (1.00,1.59) | ⨁⨁⨁⨁ HIGH |
| OMG3/ST vs. EZT/ST | N/A | N/A | 1.13  (0.90,1.42) | ⨁⨁◯◯^c^ LOW | 1.07  (0.83,1.36) | ⨁⨁◯◯ LOW |
| PCSK/ST vs. EZT/ST | 0.95  (0.69,1.31) | ⨁⨁⨁⨁ HIGH | 0.95  (0.76,1.20) | ⨁⨁⨁◯^e^  MODERATE | 0.95  (0.79,1.14) | ⨁⨁⨁⨁ HIGH |
| NIA+EZT/ST vs. EZT/ST | 1.94  (1.32,2.86) | ⨁⨁◯◯^a^ LOW | Not estimable | Not estimable | 1.94  (1.32,2.86) | ⨁⨁◯◯ LOW |
| NIA/ST vs. FBT/ST | N/A | N/A | 1.21  (0.89,1.65) | ⨁⨁◯◯^c^ LOW | 1.21  (0.87,1.69) | ⨁⨁◯◯ LOW |
| OMG3/ST vs. FBT/ST | N/A | N/A | 1.06  (0.77,1.44) | ⨁⨁◯◯^c^ LOW | 1.03  (0.74,1.43) | ⨁⨁◯◯ LOW |
| PCSK/ST vs. FBT/ST | N/A | N/A | 0.91  (0.68,1.20) | ⨁⨁⨁◯^e^  MODERATE | 0.92  (0.68,1.24) | ⨁⨁⨁◯ MODERATE |
| NIA+EZT/ST vs. FBT/ST | N/A | N/A | 1.82  (1.14,2.91) | ⨁⨁◯◯^c^ LOW | 1.87  (1.13,3.10) | ⨁⨁◯◯ LOW |
| OMG3/ST vs. NIA/ST | N/A | N/A | 0.87  (0.69,1.10) | ⨁⨁◯◯^c^ LOW | 0.85  (0.66,1.09) | ⨁⨁◯◯ LOW |
| PCSK/ST vs. NIA/ST | N/A | N/A | 0.75  (0.61,0.91) | ⨁⨁◯◯^c^ LOW | 0.76  (0.61,0.93) | ⨁⨁◯◯ LOW |
| NIA+EZT/ST vs. NIA/ST | N/A | N/A | 1.50  (0.99,2.28) | ⨁⨁◯◯^c^ LOW | 1.54  (0.98,2.43) | ⨁⨁◯◯ LOW |
| PCSK/ST vs. OMG3/ST | N/A | N/A | 0.86  (0.70,1.06) | ⨁⨁◯◯^c^ LOW | 0.89  (0.72,1.11) | ⨁⨁◯◯ LOW |
| NIA+EZT/ST vs. OMG3/ST | N/A | N/A | 1.72  (1.12,2.64) | ⨁⨁◯◯^c^ LOW | 1.82  (1.15,2.88) | ⨁⨁◯◯ LOW |
| NIA+EZT/ST vs. PCSK/ST | N/A | N/A | 2.01  (1.35,2.99) | ⨁⨁◯◯^c^ LOW | 2.04  (1.33,3.14) | ⨁⨁◯◯ LOW |
| **Discontinuation due to adverse events** | | | | | | |
| CETP/ST vs. ST | 1.15  (1.00,1.33) | ⨁⨁◯◯^a,g^ LOW | Not estimable | Not estimable | 1.15  (1.00,1.33) | ⨁⨁◯◯  LOW |
| EZT/ST vs. ST | 0.94  (0.76,1.17) | ⨁⨁⨁◯^a^ MODERATE | 0.68  (0.38,1.21) | ⨁⨁⨁◯^e^  MODERATE | 0.90  (0.74,1.11) | ⨁⨁⨁◯ MODERATE |
| FBT/ST vs. ST | 2.00  (1.16,3.46) | ⨁⨁⨁⨁  HIGH | Not estimable | Not estimable | 2.00  (1.16,3.46) | ⨁⨁⨁⨁  HIGH |
| NIA/ST vs. ST | 1.83  (1.47,2.26) | ⨁⨁◯◯^a,g^ LOW | 4.95  (1.40,17.47) | ⨁⨁⨁◯^e^  MODERATE | 1.88  (1.52,2.32) | ⨁⨁⨁◯ MODERATE |
| OMG3/ST vs. ST | 1.47  (1.14,1.91) | ⨁⨁⨁◯^a^ MODERATE | Not estimable | Not estimable | 1.47  (1.14,1.91) | ⨁⨁⨁◯ MODERATE |
| PCSK/ST vs. ST | 1.07  (0.90,1.28) | ⨁⨁⨁◯^a^ MODERATE | 1.12  (0.57,2.20) | ⨁⨁⨁◯^e^  MODERATE | 1.08  (0.91,1.28) | ⨁⨁⨁◯ MODERATE |
| NIA+EZT/ST vs. ST | N/A | N/A | 2.27  (1.33,3.88) | ⨁⨁◯◯^c^ LOW | 2.27  (1.33,3.88) | ⨁⨁◯◯ LOW |
| EZT/ST vs. CETP/ST | N/A | N/A | 0.75  (0.56,0.98) | ⨁⨁◯◯c  LOW | 0.78  (0.61,1.00) | ⨁⨁◯◯  LOW |
| FBT/ST vs. CETP/ST | N/A | N/A | 1.58  (0.90,2.78) | ⨁⨁◯◯c  LOW | 1.74  (0.99,3.05) | ⨁⨁◯◯  LOW |
| NIA/ST vs. CETP/ST | N/A | N/A | 1.48  (1.12,1.95) | ⨁⨁◯◯^c^ LOW | 1.63  (1.26,2.11) | ⨁⨁◯◯ LOW |
| OMG3/ST vs. CETP/ST | N/A | N/A | 1.16  (0.85,1.56) | ⨁⨁◯◯^c^  LOW | 1.28  (0.95,1.71) | ⨁⨁◯◯  LOW |
| PCSK/ST vs. CETP/ST | N/A | N/A | 0.83  (0.65,1.07) | ⨁⨁◯◯^c^  LOW | 0.93  (0.75,1.16) | ⨁⨁◯◯  LOW |
| NIA+EZT/ST vs. CETP/ST | N/A | N/A | 1.87  (1.07,3.27) | ⨁⨁◯◯^c^ LOW | 1.97  (1.13,3.43) | ⨁⨁◯◯ LOW |
| FBT/ST vs. EZT/ST | N/A | N/A | 2.12  (1.19,3.77) | ⨁⨁⨁◯^e^  MODERATE | 2.22  (1.24,3.96) | ⨁⨁⨁◯ MODERATE |
| NIA/ST vs. EZT/ST | 5.33  (1.53,18.53) | ⨁⨁⨁◯^d^ MODERATE | 1.97  (1.47,2.65) | ⨁⨁◯◯^c^ LOW | 2.08  (1.55,2.78) | ⨁⨁⨁◯ MODERATE |
| OMG3/ST vs. EZT/ST | N/A | N/A | 1.55  (1.12,2.14) | ⨁⨁⨁◯^e^  MODERATE | 1.63  (1.17,2.26) | ⨁⨁⨁◯ MODERATE |
| PCSK/ST vs. EZT/ST | 1.20  (0.71,2.03) | ⨁⨁⨁⨁  HIGH | 1.19  (0.89,1.59) | ⨁⨁⨁◯^e^  MODERATE | 1.19  (0.93,1.53) | ⨁⨁⨁⨁  HIGH |
| NIA+EZT/ST vs. EZT/ST | 2.51  (1.53,4.13) | ⨁⨁◯◯^a^ LOW | Not estimable | Not estimable | 2.51  (1.53,4.13) | ⨁⨁◯◯ LOW |
| NIA/ST vs. FBT/ST | N/A | N/A | 0.93  (0.53,1.65) | ⨁⨁◯◯^c^ LOW | 0.94  (0.52,1.68) | ⨁⨁◯◯ LOW |
| OMG3/ST vs. FBT/ST | N/A | N/A | 0.73  (0.41,1.32) | ⨁⨁⨁◯^e^  MODERATE | 0.73  (0.40,1.34) | ⨁⨁⨁◯ MODERATE |
| PCSK/ST vs. FBT/ST | N/A | N/A | 0.53  (0.30,0.92) | ⨁⨁⨁◯^e^  MODERATE | 0.54  (0.30,0.95) | ⨁⨁⨁◯ MODERATE |
| NIA+EZT/ST vs. FBT/ST | N/A | N/A | 1.18  (0.56,2.51) | ⨁⨁◯◯^c^ LOW | 1.13  (0.53,2.43) | ⨁⨁◯◯ LOW |
| OMG3/ST vs. NIA/ST | N/A | N/A | 0.78  (0.57,1.08) | ⨁⨁◯◯^c^ LOW | 0.78  (0.56,1.10) | ⨁⨁◯◯ LOW |
| PCSK/ST vs. NIA/ST | N/A | N/A | 0.56  (0.44,0.73) | ⨁⨁◯◯^c^ LOW | 0.57  (0.44,0.75) | ⨁⨁◯◯ LOW |
| NIA+EZT/ST vs. NIA/ST | N/A | N/A | 1.27  (0.72,2.23) | ⨁⨁◯◯^c^ LOW | 1.21  (0.68,2.15) | ⨁⨁◯◯ LOW |
| PCSK/ST vs. OMG3/ST | N/A | N/A | 0.72  (0.54,0.97) | ⨁⨁⨁◯^e^  MODERATE | 0.73  (0.54,1.00) | ⨁⨁⨁◯ MODERATE |
| NIA+EZT/ST vs. OMG3/ST | N/A | N/A | 1.62  (0.91,2.90) | ⨁⨁◯◯^c^ LOW | 1.54  (0.85,2.80) | ⨁⨁◯◯ LOW |
| NIA+EZT/ST vs. PCSK/ST | N/A | N/A | 2.24  (1.30,3.88) | ⨁⨁◯◯^c^ LOW | 2.11  (1.21,3.68) | ⨁⨁◯◯ LOW |

^a^Limitation (risk of bias); ^b^indirectness (different in population); ^c^contributing direct evidence of low or very low quality; ^d^wide confidence interval; ^e^contributing direct evidence of moderate quality;  ^f^contributing direct evidence of high quality; ^g^heterogeneity.

Abbreviation: CETP/ST=cholesteryl ester transfer protein inhibitor + statin; EZT/ST=ezetimibe + statin; FBT/ST=fibrate + statin; NIA/ST=niacin + statin; OMG3/ST=omega-3 fatty acids + statin; PCSK/ST=proprotein convertase subtilisin/kexin type 9 inhibitor + statin; N/A=not available; NIA+EZT/ST= niacin + ezetimibe + statin; PCSK+EZT/ST= proprotein convertase subtilisin/kexin type 9 inhibitor + ezetimibe + statin; ST=statin monotherapy.

Appendix 17 eReferences

1. Cannon, C.P., Blazing, M.A., Giugliano, R.P., Mccagg, A., White, J.A., Theroux, P., et al. (2015a). Ezetimibe Added to Statin Therapy after Acute Coronary Syndromes. *N Engl J Med* 372**,** 2387-2397.
2. Kastelein, J.J., Akdim, F., Stroes, E.S., Zwinderman, A.H., Bots, M.L., Stalenhoef, A.F., et al. (2008). Simvastatin with or without ezetimibe in familial hypercholesterolemia. *N Engl J Med* 358**,** 1431-1443.
3. Taylor, A.J., Sullenberger, L.E., Lee, H.J., Lee, J.K., and Grace, K.A. (2004). Arterial Biology for the Investigation of the Treatment Effects of Reducing Cholesterol (ARBITER) 2: a double-blind, placebo-controlled study of extended-release niacin on atherosclerosis progression in secondary prevention patients treated with statins. *Circulation* 110**,** 3512-3517.
4. Boden, W.E., Probstfield, J.L., Anderson, T., Chaitman, B.R., Desvignes-Nickens, P., Koprowicz, K., et al. (2011). Niacin in patients with low HDL cholesterol levels receiving intensive statin therapy. *N Engl J Med* 365**,** 2255-2267.
5. Landray, M.J., Haynes, R., Hopewell, J.C., Parish, S., Aung, T., Tomson, J., et al. (2014). Effects of extended-release niacin with laropiprant in high-risk patients. *N Engl J Med* 371**,** 203-212.
6. Ginsberg, H.N., Elam, M.B., Lovato, L.C., Crouse, J.R., 3rd, Leiter, L.A., Linz, P., et al. (2010). Effects of combination lipid therapy in type 2 diabetes mellitus. *N Engl J Med* 362**,** 1563-1574.
7. Yokoyama, M., Origasa, H., Matsuzaki, M., Matsuzawa, Y., Saito, Y., Ishikawa, Y., et al. (2007). Effects of eicosapentaenoic acid on major coronary events in hypercholesterolaemic patients (JELIS): a randomised open-label, blinded endpoint analysis. *Lancet* 369**,** 1090-1098.
8. Nissen, S.E., Tardif, J.C., Nicholls, S.J., Revkin, J.H., Shear, C.L., Duggan, W.T., et al. (2007). Effect of torcetrapib on the progression of coronary atherosclerosis. *N Engl J Med* 356**,** 1304-1316.
9. Barter, P.J., Caulfield, M., Eriksson, M., Grundy, S.M., Kastelein, J.J., Komajda, M., et al. (2007b). Effects of torcetrapib in patients at high risk for coronary events. *N Engl J Med* 357**,** 2109-2122.
10. Schwartz, G.G., Olsson, A.G., Abt, M., Ballantyne, C.M., Barter, P.J., Brumm, J., et al. (2012). Effects of dalcetrapib in patients with a recent acute coronary syndrome. *N Engl J Med* 367**,** 2089-2099.
11. Robinson, J.G., Farnier, M., Krempf, M., Bergeron, J., Luc, G., Averna, M., et al. (2015). Efficacy and safety of alirocumab in reducing lipids and cardiovascular events. *N Engl J Med* 372**,** 1489-1499.
12. Sabatine, M.S., Giugliano, R.P., Wiviott, S.D., Raal, F.J., Blom, D.J., Robinson, J., et al. (2015). Efficacy and safety of evolocumab in reducing lipids and cardiovascular events. *N Engl J Med* 372**,** 1500-1509.
13. Taylor, A.J., Villines, T.C., Stanek, E.J., Devine, P.J., Griffen, L., Miller, M., et al. (2009). Extended-release niacin or ezetimibe and carotid intima-media thickness. *N Engl J Med* 361**,** 2113-2122.
14. Guyton, J.R., Brown, B.G., Fazio, S., Polis, A., Tomassini, J.E., and Tershakovec, A.M. (2008). Lipid-altering efficacy and safety of ezetimibe/simvastatin coadministered with extended-release niacin in patients with type IIa or type IIb hyperlipidemia. *J Am Coll Cardiol* 51**,** 1564-1572.
15. Brunner, G., Yang, E.Y., Kumar, A., Sun, W., Virani, S.S., Negi, S.I., et al. (2013). The Effect of Lipid Modification on Peripheral Artery Disease after Endovascular Intervention Trial (ELIMIT). *Atherosclerosis* 231**,** 371-377.
16. Ballantyne, C.M., Davidson, M.H., Mckenney, J., Keller, L.H., Bajorunas, D.R., and Karas, R.H. (2008a). Comparison of the safety and efficacy of a combination tablet of niacin extended release and simvastatin vs simvastatin monotherapy in patients with increased non-HDL cholesterol (from the SEACOAST I study). *Am J Cardiol* 101**,** 1428-1436.
17. Wang, X., Zhao, X., Li, L., Yao, H., Jiang, Y., and Zhang, J. (2016). Effects of Combination of Ezetimibe and Rosuvastatin on Coronary Artery Plaque in Patients with Coronary Heart Disease. *Heart Lung Circ* 25**,** 459-465.
18. Tsujita, K., Sugiyama, S., Sumida, H., Shimomura, H., Yamashita, T., Yamanaga, K., et al. (2015). Impact of Dual Lipid-Lowering Strategy With Ezetimibe and Atorvastatin on Coronary Plaque Regression in Patients With Percutaneous Coronary Intervention: The Multicenter Randomized Controlled PRECISE-IVUS Trial. *J Am Coll Cardiol* 66**,** 495-507.
19. Masuda, J., Tanigawa, T., Yamada, T., Nishimura, Y., Sasou, T., Nakata, T., et al. (2015). Effect of combination therapy of ezetimibe and rosuvastatin on regression of coronary atherosclerosis in patients with coronary artery disease. *Int Heart J* 56**,** 278-285.
20. Luo, P., Li, L., Wang, L.X., Zhu, H.H., Du, S., Wu, S.L., et al. (2014). Effects of atorvastatin in combination with ezetimibe on carotid atherosclerosis in elderly patients with hypercholesterolemia. *Genet Mol Res* 13**,** 2377-2384.
21. Rauch, B., Schiele, R., Schneider, S., Diller, F., Victor, N., Gohlke, H., et al. (2010). OMEGA, a randomized, placebo-controlled trial to test the effect of highly purified omega-3 fatty acids on top of modern guideline-adjusted therapy after myocardial infarction. *Circulation* 122**,** 2152-2159.
22. Farnier, M., Jones, P., Severance, R., Averna, M., Steinhagen-Thiessen, E., Colhoun, H.M., et al. (2016). Efficacy and safety of adding alirocumab to rosuvastatin versus adding ezetimibe or doubling the rosuvastatin dose in high cardiovascular-risk patients: The ODYSSEY OPTIONS II randomized trial. *Atherosclerosis* 244**,** 138-146.
23. Cannon, C.P., Cariou, B., Blom, D., Mckenney, J.M., Lorenzato, C., Pordy, R., et al. (2015b). Efficacy and safety of alirocumab in high cardiovascular risk patients with inadequately controlled hypercholesterolaemia on maximally tolerated doses of statins: the ODYSSEY COMBO II randomized controlled trial. *Eur Heart J* 36**,** 1186-1194.
24. Blom, D.J., Hala, T., Bolognese, M., Lillestol, M.J., Toth, P.D., Burgess, L., et al. (2014). A 52-week placebo-controlled trial of evolocumab in hyperlipidemia. *N Engl J Med* 370**,** 1809-1819.
25. West, A.M., Anderson, J.D., Meyer, C.H., Epstein, F.H., Wang, H., Hagspiel, K.D., et al. (2011). The effect of ezetimibe on peripheral arterial atherosclerosis depends upon statin use at baseline. *Atherosclerosis* 218**,** 156-162.
26. Arimura, T., Miura, S., Ike, A., Sugihara, M., Iwata, A., Nishikawa, H., et al. (2012). Comparison of the efficacy and safety of statin and statin/ezetimibe therapy after coronary stent implantation in patients with stable angina. *J Cardiol* 60**,** 111-118.
27. Bots, M.L., Visseren, F.L., Evans, G.W., Riley, W.A., Revkin, J.H., Tegeler, C.H., et al. (2007). Torcetrapib and carotid intima-media thickness in mixed dyslipidaemia (RADIANCE 2 study): a randomised, double-blind trial. *Lancet* 370**,** 153-160.
28. Kastelein, J.J., Besseling, J., Shah, S., Bergeron, J., Langslet, G., Hovingh, G.K., et al. (2015a). Anacetrapib as lipid-modifying therapy in patients with heterozygous familial hypercholesterolaemia (REALIZE): a randomised, double-blind, placebo-controlled, phase 3 study. *Lancet* 385**,** 2153-2161.
29. Cannon, C.P., Shah, S., Dansky, H.M., Davidson, M., Brinton, E.A., Gotto, A.M., et al. (2010). Safety of anacetrapib in patients with or at high risk for coronary heart disease. *N Engl J Med* 363**,** 2406-2415.
30. Davidson, M.H., Rosenson, R.S., Maki, K.C., Nicholls, S.J., Ballantyne, C.M., Mazzone, T., et al. (2014). Effects of fenofibric acid on carotid intima-media thickness in patients with mixed dyslipidemia on atorvastatin therapy: randomized, placebo-controlled study (FIRST). *Arterioscler Thromb Vasc Biol* 34**,** 1298-1306.
31. Kastelein, J.J., Van Leuven, S.I., Burgess, L., Evans, G.W., Kuivenhoven, J.A., Barter, P.J., et al. (2007). Effect of torcetrapib on carotid atherosclerosis in familial hypercholesterolemia. *N Engl J Med* 356**,** 1620-1630.
32. Derosa, G., Cicero, A.E., Bertone, G., Piccinni, M.N., Ciccarelli, L., and Roggeri, D.E. (2004). Comparison of fluvastatin + fenofibrate combination therapy and fluvastatin monotherapy in the treatment of combined hyperlipidemia, type 2 diabetes mellitus, and coronary heart disease: a 12-month, randomized, double-blind, controlled trial. *Clin Ther* 26**,** 1599-1607.
33. Durrington, P.N., Bhatnagar, D., Mackness, M.I., Morgan, J., Julier, K., Khan, M.A., et al. (2001). An omega-3 polyunsaturated fatty acid concentrate administered for one year decreased triglycerides in simvastatin treated patients with coronary heart disease and persisting hypertriglyceridaemia. *Heart* 85**,** 544-548.
34. Kastelein, J.J., Ginsberg, H.N., Langslet, G., Hovingh, G.K., Ceska, R., Dufour, R., et al. (2015b). ODYSSEY FH I and FH II: 78 week results with alirocumab treatment in 735 patients with heterozygous familial hypercholesterolaemia. *Eur Heart J* 36**,** 2996-3003.
35. Kereiakes, D.J., Robinson, J.G., Cannon, C.P., Lorenzato, C., Pordy, R., Chaudhari, U., et al. (2015). Efficacy and safety of the proprotein convertase subtilisin/kexin type 9 inhibitor alirocumab among high cardiovascular risk patients on maximally tolerated statin therapy: The ODYSSEY COMBO I study. *Am Heart J* 169**,** 906-915.e913.
36. Nishio, R., Shinke, T., Otake, H., Nakagawa, M., Nagoshi, R., Inoue, T., et al. (2014). Stabilizing effect of combined eicosapentaenoic acid and statin therapy on coronary thin-cap fibroatheroma. *Atherosclerosis* 234**,** 114-119.
37. Teramoto, T., Kobayashi, M., Tasaki, H., Yagyu, H., Higashikata, T., Takagi, Y., et al. (2016). Efficacy and Safety of Alirocumab in Japanese Patients With Heterozygous Familial Hypercholesterolemia or at High Cardiovascular Risk With Hypercholesterolemia Not Adequately Controlled With Statins- ODYSSEY JAPAN Randomized Controlled Trial. *Circ J* 80**,** 1980-1987.
38. Stein, E.A., Roth, E.M., Rhyne, J.M., Burgess, T., Kallend, D., and Robinson, J.G. (2010). Safety and tolerability of dalcetrapib (RO4607381/JTT-705): results from a 48-week trial. *Eur Heart J* 31**,** 480-488.
39. Bays, H., Gaudet, D., Weiss, R., Ruiz, J.L., Watts, G.F., Gouni-Berthold, I., et al. (2015). Alirocumab as Add-On to Atorvastatin Versus Other Lipid Treatment Strategies: ODYSSEY OPTIONS I Randomized Trial. *J Clin Endocrinol Metab* 100**,** 3140-3148.
40. Fayad, Z.A., Mani, V., Woodward, M., Kallend, D., Abt, M., Burgess, T., et al. (2011). Safety and efficacy of dalcetrapib on atherosclerotic disease using novel non-invasive multimodality imaging (dal-PLAQUE): a randomised clinical trial. *Lancet* 378**,** 1547-1559.
41. Luscher, T.F., Taddei, S., Kaski, J.C., Jukema, J.W., Kallend, D., Munzel, T., et al. (2012). Vascular effects and safety of dalcetrapib in patients with or at risk of coronary heart disease: the dal-VESSEL randomized clinical trial. *Eur Heart J* 33**,** 857-865.
42. Ballantyne, C.M., Davidson, M.H., Mckenney, J.M., Keller, L.H., Bajorunas, D.R., and Karas, R.H. (2008b). Comparison of the efficacy and safety of a combination tablet of niacin extended-release and simvastatin with simvastatin 80 mg monotherapy: the SEACOAST II (high-dose) study. *J Clin Lipidol* 2**,** 79-90.
43. Nicholls, S.J., Puri, R., Anderson, T., Ballantyne, C.M., Cho, L., Kastelein, J.J., et al. (2016). Effect of Evolocumab on Progression of Coronary Disease in Statin-Treated Patients: The GLAGOV Randomized Clinical Trial. *Jama* 316**,** 2373-2384.
44. Landray, M., Baigent, C., Leaper, C., Adu, D., Altmann, P., Armitage, J., et al. (2006). The second United Kingdom Heart and Renal Protection (UK-HARP-II) Study: a randomized controlled study of the biochemical safety and efficacy of adding ezetimibe to simvastatin as initial therapy among patients with CKD. *Am J Kidney Dis* 47**,** 385-395.
45. Shaw, S.M., Chaggar, P., Ritchie, J., Shah, M.K., Baynes, A.C., O'neill, N., et al. (2009). The efficacy and tolerability of ezetimibe in cardiac transplant recipients taking cyclosporin. *Transplantation* 87**,** 771-775.
46. Kouvelos, G.N., Arnaoutoglou, E.M., Matsagkas, M.I., Kostara, C., Gartzonika, C., Bairaktari, E.T., et al. (2013). Effects of rosuvastatin with or without ezetimibe on clinical outcomes in patients undergoing elective vascular surgery: results of a pilot study. *J Cardiovasc Pharmacol Ther* 18**,** 5-12.
47. Ginsberg, H.N., Rader, D.J., Raal, F.J., Guyton, J.R., Baccara-Dinet, M.T., Lorenzato, C., et al. (2016). Efficacy and Safety of Alirocumab in Patients with Heterozygous Familial Hypercholesterolemia and LDL-C of 160 mg/dl or Higher. *Cardiovasc Drugs Ther* 30**,** 473-483.
48. Ballantyne, C.M., Shah, S., Kher, U., Hunter, J.A., Gill, G.G., Cressman, M.D., et al. (2017a). Lipid-Modifying Efficacy and Tolerability of Anacetrapib Added to Ongoing Statin Therapy in Patients with Hypercholesterolemia or Low High-Density Lipoprotein Cholesterol. *Am J Cardiol* 119**,** 388-396.
49. Kromhout, D., Giltay, E.J., and Geleijnse, J.M. (2010). n-3 fatty acids and cardiovascular events after myocardial infarction. *N Engl J Med* 363**,** 2015-2026.
50. Sabatine, M.S., Giugliano, R.P., Keech, A.C., Honarpour, N., Wiviott, S.D., Murphy, S.A., et al. (2017). Evolocumab and Clinical Outcomes in Patients with Cardiovascular Disease. *N Engl J Med* 376**,** 1713-1722.
51. Ridker, P.M., Revkin, J., Amarenco, P., Brunell, R., Curto, M., Civeira, F., et al. (2017a). Cardiovascular Efficacy and Safety of Bococizumab in High-Risk Patients. *N Engl J Med* 376**,** 1527-1539.
52. Ridker, P.M., Tardif, J.C., Amarenco, P., Duggan, W., Glynn, R.J., Jukema, J.W., et al. (2017b). Lipid-Reduction Variability and Antidrug-Antibody Formation with Bococizumab. *N Engl J Med* 376**,** 1517-1526.
53. Luo, P., Wang, L., Zhu, H., Du, S., Wang, G., and Ding, S. (2016). Impact of atorvastatin combined with ezetimibe for the treatment of carotid atherosclerosis in patients with coronary heart disease. *Acta Cardiologica Sinica* 32**,** 578-585.
54. Liu, Z., Hao, H., Yin, C., Chu, Y., Li, J., and Xu, D. (2017). Therapeutic effects of atorvastatin and ezetimibe compared with double-dose atorvastatin in very elderly patients with acute coronary syndrome. *Oncotarget* 8**,** 41582-41589.
55. Nosaka, K., Miyoshi, T., Iwamoto, M., Kajiya, M., Okawa, K., Tsukuda, S., et al. (2017). Early initiation of eicosapentaenoic acid and statin treatment is associated with better clinical outcomes than statin alone in patients with acute coronary syndromes: 1-year outcomes of a randomized controlled study. *Int J Cardiol* 228**,** 173-179.
56. Lincoff, A.M., Nicholls, S.J., Riesmeyer, J.S., Barter, P.J., Brewer, H.B., Fox, K.a.A., et al. (2017). Evacetrapib and Cardiovascular Outcomes in High-Risk Vascular Disease. *N Engl J Med* 376**,** 1933-1942.
57. Bowman, L., Hopewell, J.C., Chen, F., Wallendszus, K., Stevens, W., Collins, R., et al. (2017). Effects of Anacetrapib in Patients with Atherosclerotic Vascular Disease. *N Engl J Med* 377**,** 1217-1227.
58. Hagiwara, N., Kawada-Watanabe, E., Koyanagi, R., Arashi, H., Yamaguchi, J., Nakao, K., et al. (2017). Low-density lipoprotein cholesterol targeting with pitavastatin + ezetimibe for patients with acute coronary syndrome and dyslipidaemia: the HIJ-PROPER study, a prospective, open-label, randomized trial. *Eur Heart J* 38**,** 2264-2276.
59. Hibi, K., Sonoda, S., Kawasaki, M., Otsuji, Y., Murohara, T., Ishii, H., et al. (2018). Effects of Ezetimibe-Statin Combination Therapy on Coronary Atherosclerosis in Acute Coronary Syndrome. *Circ J* 82**,** 757-766.
60. Miyoshi, T., Kohno, K., Asonuma, H., Sakuragi, S., Nakahama, M., Kawai, Y., et al. (2018). Effect of Intensive and Standard Pitavastatin Treatment With or Without Eicosapentaenoic Acid on Progression of Coronary Artery Calcification Over 12 Months- Prospective Multicenter Study. *Circ J* 82**,** 532-540.
61. Watanabe, T., Ando, K., Daidoji, H., Otaki, Y., Sugawara, S., Matsui, M., et al. (2017). A randomized controlled trial of eicosapentaenoic acid in patients with coronary heart disease on statins. *J Cardiol* 70**,** 537-544.
62. Koh, K.K., Nam, C.W., Chao, T.H., Liu, M.E., Wu, C.J., Kim, D.S., et al. (2018). A randomized trial evaluating the efficacy and safety of alirocumab in South Korea and Taiwan (ODYSSEY KT). *J Clin Lipidol* 12**,** 162-172.e166.
63. Leiter, L.A., Cariou, B., Muller-Wieland, D., Colhoun, H.M., Del Prato, S., Tinahones, F.J., et al. (2017). Efficacy and safety of alirocumab in insulin-treated individuals with type 1 or type 2 diabetes and high cardiovascular risk: The ODYSSEY DM-INSULIN randomized trial. *Diabetes Obes Metab* 19**,** 1781-1792.
64. Teramoto, T., Daida, H., Ikewaki, K., Arai, H., Maeda, Y., Nakagomi, M., et al. (2017). Lipid-modifying efficacy and tolerability of anacetrapib added to ongoing statin therapy in Japanese patients with dyslipidemia. *Atherosclerosis* 261**,** 69-77.
65. Ballantyne, C.M., Shah, S., Sapre, A., Ashraf, T.B., Tobias, S.C., Sahin, T., et al. (2017b). A Multiregional, Randomized Evaluation of the Lipid-Modifying Efficacy and Tolerability of Anacetrapib Added to Ongoing Statin Therapy in Patients With Hypercholesterolemia or Low High-Density Lipoprotein Cholesterol. *Am J Cardiol* 120**,** 569-576.
66. Ray, K.K., Leiter, L.A., Muller-Wieland, D., Cariou, B., Colhoun, H.M., Henry, R.R., et al. (2018). Alirocumab vs usual lipid-lowering care as add-on to statin therapy in individuals with type 2 diabetes and mixed dyslipidaemia: The ODYSSEY DM-DYSLIPIDEMIA randomized trial. *Diabetes Obes Metab* 20**,** 1479-1489.
67. Schwartz, G.G., Steg, P.G., Szarek, M., Bhatt, D.L., Bittner, V.A., Diaz, R., et al. (2018). Alirocumab and Cardiovascular Outcomes after Acute Coronary Syndrome. *N Engl J Med* 379**,** 2097-2107.
68. Sang, Z.C., Wang, F., Zhou, Q., Li, Y.H., Li, Y.G., Wang, H.P., et al. (2009). Combined use of extended-release niacin and atorvastatin: safety and effects on lipid modification. *Chin Med J (Engl)* 122**,** 1615-1620.
69. Stone, N.J., Robinson, J.G., Lichtenstein, A.H., Bairey Merz, C.N., Blum, C.B., Eckel, R.H., et al. (2014). 2013 ACC/AHA guideline on the treatment of blood cholesterol to reduce atherosclerotic cardiovascular risk in adults: a report of the American College of Cardiology/American Heart Association Task Force on Practice Guidelines. *J Am Coll Cardiol* 63**,** 2889-2934.
70. Catapano, A.L., Graham, I., De Backer, G., Wiklund, O., Chapman, M.J., Drexel, H., et al. (2016). 2016 ESC/EAS Guidelines for the Management of Dyslipidaemias. *Eur Heart J* 37**,** 2999-3058.
